# Validity of Food insecurity experience scale (FIES) for use in rural Bangladesh and prevalence and determinants of household food insecurity: An analysis of data from Bangladesh integrated household survey (BIHS) 2018-2019

**DOI:** 10.1016/j.heliyon.2023.e17378

**Published:** 2023-06-17

**Authors:** Ahmed Jubayer, Saiful Islam, Abira Nowar, Md. Moniruzzaman Nayan, Md. Hafizul Islam

**Affiliations:** a Institute of Nutrition and Food Science, University of Dhaka, Dhaka, 1000, Bangladesh; b Bangladesh Institute of Social Research (BISR) Trust, Dhaka, 1207, Bangladesh

**Keywords:** Food insecurity, Rasch modeling, FIES, Rural Bangladesh

## Abstract

“Access” dimension of Food insecurity (FI) is directly measured by the Food Insecurity Experience Scale (FIES). The current study assessed the appropriateness of the FIES for measuring FI in rural Bangladesh, followed by an assessment of FI prevalence and its correlates utilizing Bangladesh Integrated Household Survey (BIHS) data. The internal validity of the FIES and the prevalence of FI were investigated using the Rasch modeling approach. We utilized equating procedure to calibrate the study’s result to the global FIES reference scale and determined FI prevalence rates that were comparable across countries. The external validity of the FIES was evaluated by examining its association with other FI measures using Spearman’s rho correlation analysis. With an overall Rasch reliability of 0.84, the FIES met the Rasch model assumptions of conditional independence and equal discrimination, and as well as the fit statistics standards for all eight items. Infit statistics were within the allowed limit for all FIES items indicating good internal validity. However, we noted a high outfit (>2) for the “unable to eat healthy and nutritious food” item indicating the presence of some unusual response patterns. Our analysis found no significant (>0.4) correlation between FIES items. We also found a significant correlation between FIES and other FI proxies, e.g., the Household hunger scale (HHS), Food consumption score (FCS), and Household dietary diversity score (HDDS). Overall, the prevalence of moderate or severe FI was 18.92% in rural Bangladesh. Geographic areas, access to electricity, household ownership, access to sanitation, livestock ownership, family size, education level, and monthly per capita food expenditure significantly explained the variation in FI. Our analyses suggest that the FIES is internally and externally valid for FI measurement in rural Bangladesh. However, FIES questions may need to be reordered to more accurately evaluate lower levels of FI, and the item “unable to eat healthy and nutritious food” may need cognitive testing.

## 1 Introduction

Food security refers to a state in which “all people, at all times, have physical, social, and economic access to sufficient, safe, and nutritious food that meets their dietary needs and food preferences for an active and healthy life” [1]. Under Sustainable Development Goal (SDG) - 2, all member states of the United Nations have committed to ending hunger in all its forms by 2030 [2]. Household Food insecurity (FI) is one of the crucial determinants of maternal and child malnutrition and mental health issues in adults [3]. FI is also associated with the child’s sub-optimal physical and physiological development, negatively impacting their health and ability to reach their full potential [4].

More than 820 million people still go to bed hungry every night, and about 2.37 billion have moderate or severe food insecure [5]. Asia is home to about half of the world’s food-insecure individuals (1.2 billion) [5]. Moreover, 928 million people worldwide are severely food insecure, which has turned into a grave concern in the Africa (21.5%) and Southern Asian regions (14.4%) [5]. Bangladesh has achieved great strides over the past three decades in its fight against poverty and FI, with the poverty rate dropping from 40% in 2005 to 20.5% in 2018 [6]. However, the International Food Policy Research Institute (IFPRI) and Cornell University conducted a telephone survey in 2088 rural households in Bangladesh between December 29, 2020, and January 27, 2021, and found that 16% of those households had moderate FI and 2% had severe FI [7].

Food insecurity is a multi-dimensional problem resulting from diverse issues of individual, household, social, political, economic, and environmental factors. As shown by Chowdhury et al. 2016 [8], a low level of education and poor socioeconomic status are significantly associated with household FI in Bangladesh. Similarly, other studies suggest that monthly household income, age of household head, land ownership, access to media, and wealth index are associated with household FI [9,10].

“Access to adequate food,” a vital aspect of human welfare, is one of the crucial aspects of food security. Food insecurity experience scale (FIES) was developed by the Voices of the Hungry initiative of the United Nations Food and Agriculture Organization (FAO-VoH) to minimize complexity and to address the demand for a simple, universally acceptable, and comparable FI evaluation tool [11]. The FIES provides an internationally comparable estimate of the severity of FI experience and is the specified measure for the SDG indicator 2.1.2 ‘prevalence of moderate or severe FI in the population” [2]. This experience-based FI measure is similar to other FI experimental measures, such as US Household food security survey module, Household food insecurity access scale, etc. [12].

The FIES uses an experience-based metric, modeled as a latent trait to assess FI severity at the individual or household level. It pertains to eight questions about respondents’ experiences regarding access to adequate food, difficulties in accessing food, and coping strategies due to lack of money or other resources during a reference period (i.e., 12 months). According to FIES, moderate FI exists when people face uncertainties about their access to food due to a lack of money/resources and are forced to adopt coping strategies, reducing the quality and/or quantity of food they consume. People with severe FI run out of food, experience hunger, and, at the most extreme, go days without eating [13].

The FAO-VoH evaluated FIES’s psychometric properties using data from the 2014 Gallup World Poll (GWP) survey [14]. Since the GWP adopted a global standard, the reliability of its psychometric properties in Bangladesh has not been tested. Many studies have tested the validity of the FIES in the League of Arab States [15], Sub-Saharan Africa [16], and the Bahamas [17]. However, cultural and linguistic differences in rural Bangladesh could lead to variations in FIES’s psychometric properties; hence, it is important to assess FIES’s appropriateness for application in Bangladesh. Therefore, the present study was the first intended to examine the validity of FIES in rural Bangladesh using nationally representative Bangladesh integrated household survey (BIHS) data. This study also determined the extent to which FI exists in rural Bangladesh and identified the factors associated with FI.

## 2 Methods

### 2.1 Data source

We utilized the most recent BIHS survey data, carried out between 2019 and 2020 [18]. The BIHS data have been used in evidence-based policy research addressing FI and agricultural development concerns in Bangladesh [19,20].

BIHS is the country’s most extensive household survey to date. It provides detailed data related to various nutrition and development issue such as agriculture, food insecurity, dietary consumption, women empowerment, etc. [19]. However, only rural Bangladesh and rural areas in each of the country’s seven administrative divisions are represented in the BIHS sample. This study selected 5605 rural households through a two-stage stratified sampling process.

Data Analysis and Technical Assistance Limited carried out the BIHS study with oversight from senior IFPRI researchers. Interviews were conducted with one adult male family member by a male interviewer and one adult female family member by a female interviewer.

### 2.2 The FIES questions

Unlike the previous two surveys, BIHS 2018-2019 collected comprehensive data on household FI and resilience using the FIES [21]. The FIES contained eight questions varying in difficulty from question 1’s mild FI to question 8’s extreme FI (question 8) (Table 1). Those questions ranged from “being worried about not having enough food to eat” to “going hungry for a whole day” due to lack of money or other resources. Respondents (the person in charge of the household’s food preparation) were asked if they had ever experienced different severity levels of FI in the previous 12 months.

**Table 1:** Questions of the FIES used in the BIHS survey.

Item^a^	Item description (If any member of household experiences the following situation because of lack of money or other resources)	Label^b^	Assumed severity of FI
1	Worried about not having enough food	WORRIED	Mild
2	Could not eat healthy and nutritious food	HEALTHY	Mild
3	Ate only a few kinds of foods	FEWFOODS	Mild
4	Had to skip a meal	SKIPPED	Moderate
5	Ate less than you thought you should	ATELESS	Moderate
6	household ran out of food	RUNOUT	Moderate
7	Were hungry but did not eat	HUNGRY	Severe
8	Went without eating for a whole day	WHLDAY	Severe

a The term item refers to the FIES questions in the survey module.

b Standard level according to the FIES methodology [22].

### 2.3 Analytical sample and FIES data processing

Observations with missing responses to any FIES questions were excluded from the 5605 households in the BIHS data. The final analytical sample included 5603 cases with complete responses (meaning no missing response to any FIES question) for the psychometric analysis of the FIES.

Although the BIHS survey module allowed for the recording of “don’t know” and “refused” responses to any FIES question, such responses were classified as “missing” for analytic purposes and were removed from the analysis. Then, 1s were assigned to affirmative responses and 0s to negative ones. Since the FIES consisted of eight questions, the raw score ranged from 0 to 8. We used the Voices of the Hungry project’s FIES app (a free and simple analytical tool for analyzing FIES data) to produce the parameter estimates and other outputs related to statistical validation [23]. Following the FIES data analysis methodology, cases with raw scores of 0 and 8 were considered extreme values and eliminated from the statistical validation process; however, they were included in the assessment of FI prevalence [14]. However, 1000 or more complete (meaning they had no missing response) and non-extreme (raw score between 1 and 7) cases are required to have confidence in the statistical validation of FIES data [12]; after data preprocessing, this study found 2661 eligible cases from the BIHS dataset.

### 2.4 Statistical validation of FIES data

#### 2.4.1 Rasch modeling

We analyzed the psychometric properties of the FIES by Rasch modeling based on the principles of Item response theory (IRT) [24]. The key assumptions of the Rasch model for measuring FIES’s validity and reliability were assessed as 1) The answers a person gives on each of the FIES’s eight questions are corelated to one another.; 2) There is a strong relationship between each item and the underlying FI feature (equal discrimination) and 3) There is only one latent trait being measured and response of the items are completely independent [11].

Rasch modeling output included item fit statistics (infit and outfit statistics), overall model fit statistics (Rasch reliability), and residual correlation matrix. An infit statistic between 0.7 and 1.3 was considered acceptable for adequate Rasch model building. A low infit value (below 0.7) indicates that the item is redundant with other items. In contrast, a high infit value (greater than 1.3) indicates an unusual response pattern, and items with high infit values are thought to be doing poorly [14]. An outfit with a score of greater than two was regarded as high, but because a few erratic responses influence it, it is not frequently used to eliminate items from the analysis [25].

We also calculated the residual correlation matrix for each pair of FIES items. If the residual correlation between two items was significantly high (≥0.4), the responses to items were considered redundant, implying that they represent the same conditions caused by FI [14]. Moreover, Rasch reliability was estimated to measure the discriminatory power of the scale overall, which is the scale’s ability to distinguish among respondents with varying degrees of FI. A Rasch reliability value of ≥0.7 was considered acceptable in the current analysis [21].

### 2.5 Equating

We identified the common items between the global and country scales by 1) examining equating plot of the two scale’s item parameters, 2) looking at the correlation between the common items’ adjusted item parameters, and 3) considering the absolute difference between the two scales’ item parameters for each item. Since we didn’t find any unique item, equating was performed with all eight items to the FIES global standard scale. After that, two globally-comparable indicators of the prevalence of FI at different levels of severity: moderate or severe FI (raw score = 4 to 6) and severe FI (raw score = 7 to 8), were calculated. All analyses were adjusted for sampling weights.

### 2.6 Differential item functioning (DIF)

To verify measurement invariance and comparability of prevalence rates at the sub-national level, DIF analysis was conducted to ensure that the FIES scale performs similarly in subsets of the data. Each subgroup’s item severity parameters were plotted against each other to visually evaluate of how well they aligned. Alongside, fit statistics were calculated among different subgroups to inspect whether some items were problematic only in a certain context.

### 2.7 Measuring external validity

We considered numerous FI measures and development indicators for testing the external validity of the FIES. The proxy indicators were chosen exclusively based on the literature, and FAO-VoH used majority of them during the formulation of the global FIES threshold. Our analysis included Household dietary diversity (HDDS), Food consumption score (FCS), Household hunger scale (HHS), Months of adequate household food provisioning (MAHFP), household asset ownership, total cash monthly expenditure, and percentage of total cash expenditure on food, access to improved sanitation and water facilities, and child anthropometry for external validation.

#### 2.7.1 Household dietary diversity score (HDDS)

HDDS measures the variety of food groups consumed throughout a certain time frame (24-recall) as a proxy for household food access [26]. The following set of 12 food groups was used to calculate HDDS:

**Table:** 

A. Cereal	G. Fish and sea foods
B. White roots and tubers	H. Nuts, seeds, and legumes
C. Vegetables	I. Milk and milk product
D. Fruits	J. Fats and oils
E. Meat	K. Sweets
F. Eggs	L. Miscellaneous

The respondents were asked whether anyone of the household consumed the foods from this list during the previous 24 hour. For each food group consumed, was coded as 1, otherwise coded as 0. The 12 food groups were then summed to compute HDDS, yielding a total HDDS score ranging from 0 to 12. Since there is no specific cutoff for HDDS, the present analysis treated the HDDS as a continuous variable.

#### 2.7.2 Food consumption score (FCS)

The FCS is a proxy indicator to measure caloric intake and diet quality at the household level, indicating food security status [27]. The score is based on the frequency with which various food types are consumed and are weighted accordingly. The standard food groups and standard weights used in the FCS calculation are given in supplemental table S1. The FCS score ranged from 0 to 112 and was treated as a continuous variable.

#### 2.7.3 Household hunger scale (HHS)

HHS, a household food deprivation scale, is a simple indicator for measuring household hunger in food-insecure areas. We estimated the proportion of households that fall into each of three hunger catagories: little or no hunger (HHS score 0–1), moderate hunger (HHS score 2–3), and severe hunger (HHS score 4–6) [13].

#### 2.7.4 Months of adequate household food provisioning (MAHFP)

MAHFP is used to measure household food access, a simple sum of the number of months a household reports experiencing hunger in the previous 12 months [28].

#### 2.7.5 Household asset ownership

Asset ownership is a good indicator for tracking changes in household resilience over time and monitoring progress toward increased household resilience [29]. The aggregate of positive responses to a question concerning the assets of the household was computed.

#### 2.7.6 Access to improved sanitation and water facilities

The improved water source was defined as water piped into the dwelling from a tube-well or borehole, protected dug wells, or rainwater. Water from unprotected dug wells and surface water (river, canal, dam) was considered unimproved. Households with pour-flush toilets or flush-to-piped sewer systems/septic tanks or pit latrines with slabs were considered improved sanitation facilities. In contrast, an unimproved sanitation facility was defined as a flush/pour flush to an open drain or pit latrine without a slab or hanging toilet or no toilet facility [30].

#### 2.7.7 Child anthropometry

Anthropometric data was collected using height and weight scales (the “Health O’ Meter”). WHO Anthro software (version 3.3.2) was used to determine the three anthropometric indices: Height-for-age z score (HAZ), Weight-for-age z score (WAZ), and Weight-for-height z score (WHZ). Based on the World Health Organization’s (WHO) growth standard, children were categorized as stunted (HAZ -2 SD), underweight (WAZ - 2 SD), or wasting (WHZ -2 SD) [31]. Subjects having flag data (HAZ more than +6 or less than −6, WAZ greater than +5 or less than −6, and WHZ greater than +5 or less than −5) were not included in the analysis [32].

### 2.8 Regression analysis

IBM SPSS Statistics version 25 (SPSS for windows, SPSS Inc., Chicago, IL, USA) was used for performing multiple logistic regression analysis to identify predictors of FI. The outcome variable food security was categorized as a binary variable (food secure and food insecure) based on the FIES raw score cutoff [33]. A household with a FIES score of 1 was considered to be food insecure, while those with a score of 0 indicated food security.

A set of demographic and socioeconomic factors such as geographic area, access to electricity, household ownership, access to sanitation, livestock ownership, family size, education level, and monthly per capita food expenditure were considered as explanatory variables identified a priori based on the literature review. At a significance threshold of 0.05, univariate analyses were performed for each explanatory variable. Variables whose p-values in the univariate analyses were less than 0.05 were included in the final model, which was built using the stepwise method (forward entry). The underlying assumption of the logistic regression model was investigated prior to the final model building.

The regression model’s validity was examined using Pearson goodness of fit. The degree of multicollinearity in the model was evaluated by calculating the Variance inflation factor (VIF), with a VIF larger than two indicating multicollinearity [34]. Adjusted odds ratios with 95% confidence intervals and a p value of 0.05 were used to summarize the findings.

We used the Tobit regression model to analyze the predictors of FI severity, in addition to multiple logistic regression. In the Tobit regression model, the raw FIES score (from 0 to 8) served as the dependent variable. The raw score was concentrated at 0, which led to the selection of Tobit regression as the appropriate method. Therefore, the raw score was left-censored at point 0. The model used the same covariates as a multiple logistic regression analysis.

### 2.9 Ethical approval

The Institutional Review Board at the International Food Policy Research Institute in Washington, DC, USA (Ref: 2014-44-PHND-D) granted BIHS ethical approval. The Bangladeshi government's Ministry of Agriculture approved the survey after reviewing the questionnaires. All participants gave their informed verbal agreement for participation in the study.

## 3 Result

### 3.1 General characteristics of the sampled households

A total of 5603 rural households were included in this study. The majority of the households had access to electricity, but only approximately half of them had better sanitation facilities (Supplemental table 3). Almost all rural households had their dwelling place, with more than half of them rearing livestock. In approximately half of the household'sper capita food spending was below 25 USD.

### 3.2 Severity order of FIES items

The item parameter or relative severity of the item was estimated based on the distribution of responses as a whole. Parameter values that are lower indicate less severe experiences, while those that are higher indicate more severe ones. There was a range of −4.77 to7.14 (11.91 logits) in FIES item severity parameters (Table 2). The percentage of respondents who said “yes” to items assessing more severe FI (items 6–8) was lower than the percentage who said “yes” to items measuring less severe FI (items 1–5). The item “whlday” had the highest parameter (7.14 logits) with the lowest affirmative response (0.8%), whereas the item “worried” had the lowest parameter (−4.77) and was more likely to obtain an affirmative response (44.5%). However, there was an apparent disordering of items 1–5 in measuring the severity level of FI compared to what they were supposed to measure.

**Table 2:** Proportion of affirmative responses to FIES items and item severity parameters.

Item	Number of affirmative responses n (weighted percentage)	Severity (SE)
Worried	2449 (44.5)	−4.77 (0.07)
Healthy	1954 (34.6)	−3.37 (0.06)
Fewfood	1818 (32.1)	−2.96 (0.06)
Skipped	667 (11.8)	0.21 (0.07)
Ateless	831 (14.5)	−0.35 (0.06)
Runout	233 (4.3)	2.6 (0.11)
Hungry	279 (5.5)	2.02 (0.09)
Whlday	3 (0.8)	7.14 (0.57)

SE: Standard error.

The proportion of affirmative responses declined as the severity of the items rose. Even if the severity parameters of the items assessing less severe FI (items 1–5) were inconsistent, their percentage of affirmative responses was still lower than those indicating more severe FI (items 6–8).

### 3.3 Fit statistics and overall reliability of FIES

Table 3 presents the item fit statistics and Rasch reliability value. Infit statistics were e between 0.7 and 1.3 for all FIES items, indicating a good fit for the Rasch model. These infit statistics also suggest that all items are reliably discriminating across respondents and are linked to the latent traits. Similarly, no major violation was observed for outfit statistics, although the value was slightly higher for “healthy” items than the normal value (≤2). Finally, the Rasch reliability value was 0.84, indicating a satisfactory model fit.

**Table 3:** Item fit statistics and Rasch reliability.

Item	Infit (SE of Infit)	Outfit	Rasch reliability
Worried	1.22 (0.04)	1.35	0.84
Healthy	0.97 (0.03)	3.6	
Fewfood	0.76 (0.03)	0.89	
Skipped	0.94 (0.04)	0.72	
Ateless	0.71 (0.04)	0.56	
Runout	0.78 (0.07)	0.19	
Hungry	1.18 (0.06)	0.84	
Whlday	0.99 (0.56)	0.06	

### 3.4 Conditional independence of the items

The residual correlation matrix shows the residual correlation among FIES items (Supplemental table S4). However, there was no evidence of multidimensionality in the FIES, as correlation across items was within the acceptable range of |0.4|.

### 3.5 Equating

Table 4 shows the absolute difference in severity between the items on the global and country scales. We noted that “runout” was the most discrepant in severity between the two scales. However, the differences were not too high and were close to the diagonal line (supplemental figure 1) in the equating plot. We also observed a strong correlation (98.3) between FIES items.

**Table 4:** Absolute difference between country and global standard items.

FIES item	Absolute difference	Correlation between common items
Worried	0.09	98.3
Healthy	0.09	
Fewfood	0.28	
Skipped	0.32	
Ateless	0.19	
Runout	0.48	
Hungry	0.24	
Whlday	0.12	

### 3.6 Differential item functioning

All divisions had infit statistics values in the acceptable range (0.7–1.3) for the first seven items (Supplemental table S5). A zero Infit value for “Whlday” shows that the item does not provide significant information to the measure and is relatively redundant with other items, with Chittagong and Dhaka being the exceptions.

### 3.7 External validation

Table 5 shows the association between the FIES and other FI measures. All the proxies were inversely associated with FIES except HDDS (all p < 0.001).

**Table 5:** Spearman’s rho correlations between FIES and food security proxies.

Indicators	Correlation coefficient	p-value
HDDS	0.54	<0.001
HHS	−0.16	<0.001
MAHFP	−0.21	<0.001
FCS	−0.2	<0.001
Asset ownership	−0.34	<0.001
Sanitation facilities	−0.16	<0.001
Food expenditure	−0.23	<0.001
HAZ	−0.086	<0.001
WAZ	−0.14	<0.001
WHZ	−0.09	<0.001

### 3.8 Prevalence of FI

In rural Bangladesh, 18.92% of rural households experienced moderate or severe FI, whereas only 0.84% were severely food insecure (Table 6). The prevalence of FI ranged from a high of 33.56% in the Sylhet division to a low of 11.48% in the Dhaka division. Study participants who had a rented house experienced a higher prevalence of moderate or severe FI than study participants who had ownership of a house (33.61% compared with 18.43%). Compared to households headed by men, those headed by women had a higher incidence of FI (22.78% compared with 17.98%). Moderate or severe FI was more prevalent among the participants with no access to electricity (29.41%), no access to improved sanitation (24.01%), no ownership of livestock (20.86%), those with ≤25 USD monthly per capita food expenditure (24.8%), respondents with a family size of more than 5 (19.56%), and those who had never attended school (25.02%).

**Table 6:** Prevalence of FI in rural Bangladesh (N = 5603), Bangladesh integrated household survey 2018-2019.

Background characteristics	Moderate or severe FI (weighted percentage)	Severe FI (weighted percentage)
National	18.92	0.84
Division		
Barisal	24.13	1.16
Chittagong	23.77	1.13
Dhaka	11.48	0.64
Khulna	14.24	0.86
Rajshahi	18.97	0.34
Rangpur	25.46	0.65
Sylhet	33.56	2.04
Ownership of household		
Owned	18.43	0.8
Rented	33.61	1.49
Primary adult decision-maker		
Male	17.98	0.64
Female	22.78	1.58
Access to electricity		
Yes	17.72	0.76
No	29.41	1.2
Access to sanitation		
Improved	13.61	0.7
Not improved	24.01	0.93
Livestock ownership		
Yes	17.08	0.62
No	20.86	1.03
Monthly per capita food expenditure		
≤25 USD	24.8	1.15
≥26 USD	12.66	0.47
Family size		
≤4	17.69	0.49
5+	19.56	0.99
Education level of respondent		
Never attend school	25.02	1.19
Primary or below	18.9	0.73
Secondary or higher	10.23	0.37

### 3.9 Factors influencing FI

The results of logistic regression analysis show that the odds of FI were lower in all divisions than Sylhet (Table 7). This result is supported by the Tobit regression model (Table 8). Respondents living in rented house were more likely to be food insecure than those who had their own house (AOR: 1.6, p = 0.004). Tobit regression analysis shows a similar pattern, showing that people without their own housing have a higher severity score. Considering other covariates, respondents with no access to electricity and improved sanitation were 1.5 times and 1.6 times more likely to be food insecure, respectively, than respondents with access to electricity and improved sanitation. The odds of being food insecure were 1.23 times higher among the respondents who had no ownership of livestock than those who had (AOR: 1.23, p = 0.001). Respondents with ≤25 USD monthly per capita food expenditures were 1.9 times more likely to be food insecure than the respondents with ≥26 USD monthly per capita food expenditures (AOR: 1.92, p < 0.001). The Tobit analysis also supports these results.

**Table 7:** Factors associated with household FI using multiple logistic regression (N = 5603), Bangladesh integrated household survey 2018-2019.

Parameter	AOR (95% CI)	p-value
Division		
Barisal	0.47 (0.36, 0.62)	<0.001
Chittagong	0.56 (0.46, 0.69)	<0.001
Dhaka	0.36 (0.29, 0.43)	<0.001
Khulna	0.28 (0.22, 0.36)	<0.001
Rajshahi	0.46 (0.36, 0.58)	<0.001
Rangpur	0.69 (0.54, 0.88)	0.003
Sylhet (reference)	1	
Ownership of household		0.004
Rented	1.6 (1.16, 2.2)	
Owned (reference)	1	
Access to electricity		<0.001
No	1.46 (1.23, 1.73)	
Yes (reference)	1	
Access to sanitation		<0.001
Not improved	1.6 (1.42, 1.79)	
Improved (reference)	1	
Livestock ownership		0.001
No	1.23 (1.08, 1.39)	
Yes (reference)	1	
Monthly per capita food expenditure		<0.001
≤25 USD	1.92 (1.71, 2.16)	
≥26 USD (reference)	1	
Family size		<0.001
5+	1.29 (1.14, 1.47)	
≤4 (reference)	1	
Education level of respondent		
Never attend school	2.29 (1.99, 2.64)	<0.001
Primary or below	1.68 (1.45, 1.96)	<0.001
Secondary or higher (reference)	1	

Abbreviation: AOR (adjusted odds ratio).

**Table 8:** Factors associated with household FI using Tobit regression (N = 5603), Bangladesh integrated household survey 2018-2019.

Parameter	Coefficient (95% CI)	p-value
Division		
Barisal	−1.16 (−1.53, −0.8)	<0.001
Chittagong	−0.82 (−1.08, −0.52)	<0.001
Dhaka	−1.91 (−2.17, −1.64)	<0.001
Khulna	−1.95 (−2.32, −1.58)	<0.001
Rajshahi	−1.42 (−1.74, −1.11)	<0.001
Rangpur	−0.84 (−1.16, −0.52)	<0.001
Sylhet (reference)		
Ownership of household	0.89 (0.47, 1.32)	<0.001
Rented		
Owned (reference)		
Access to electricity	0.56 (0.34, 0.78)	<0.001
No		
Yes (reference)		
Access to sanitation	0.73 (0.56, 0.9)	<0.001
Not improved		
Improved (reference)		
Livestock ownership	0.37 (0.2, 0.54)	0.001
No		
Yes (reference)		
Monthly per capita food expenditure	1.1 (0.92, 1.27)	<0.001
≤25 USD		
≥26 USD (reference)		
Family size	0.48 (0.3, 0.65)	<0.001
5+		
≤4 (reference)		
Education level of respondent		
Never attend school	1.34 (1.13, 1.54)	<0.001
Primary or below	0.87 (0.65, 1.11)	<0.001
Secondary or higher (reference)		

## 4 Discussion

Using a nationally representative large dataset, the present study sought to evaluate the internal and external validity of the FIES for measuring household FI in rural Bangladesh. We conducted a Rasch analysis to assess the performance of the FIES in the rural Bangladesh context. The statistical validity of FIES data was examined using item fit statistics, Rasch reliability, and residual correlations of items. It was found that the FIES met all of its assumptions regarding equal discrimination and conditional independence; thus, it is a valid measure of FI for Bangladesh. FIES was also significantly associated with all of the most important FI proxies. However, the prevalence of FI was estimated at 18.92% in Bangladesh. Various demographic and socioeconomic factors contributed to FI, such as geographic area, access to electricity, household ownership, access to sanitation, livestock ownership, family size, education level, and monthly per capita food expenditure.

Statistical validation is the fundamental screening tool for evaluating the quality of self-reported, experience-based FIES data and determining whether the data are compatible with the assumptions of the measurement model. It also assesses whether a given measure of FI has a strong enough statistical foundation for its intended policy and research uses. Rasch is one of the most invaluable statistical tools for developing and validating survey instruments by assessing individual items and their functionality [35]. In addition, the Rasch model serves as a statistical basis for survey-based experiential FI measurement [24].

Self-reported experiences and actions regarding food access due to a lack of money or other resources are elicited by FIES over the course of a year. One of the most important characteristics of experiential FI measures is that the FIES items vary widely in terms of the degree of FI. It is a latent feature, which means it is not directly visible. The cognitive content of the items also intuitively demonstrates the severity of the conditions detected by the items. Moreover, less often confirmed items have greater severity. However, The Rasch model provides standardized statistical methods for measuring the severity of each item by formalizing the concept of item severity ordering. By predicting their position on the same underlying continuum of severity of FI, Rasch modeling provides a theoretical basis for linking the FIES indicator items to the FIES responses [14,35]. Each respondent's item-by-item response on the scale is a logistic function of the difference between their actual FI severity and the FI severity as measured by that item [24]. The Rasch model evaluates latent qualities using raw scores, which are transformed from ordinal to continuous data using uniform interval sizes (logits) [36].

Fit statistics are chi-square type statistics [24] and were used in the current analysis to assess whether all FIES items were strongly related to the latent trait of FI. Infit is primarily used to assess the performance of the FIES items, identifying those that did not function well in the current setting. Ideally, infit statistics should be 1.0 to ensure that all items discriminate equally and that each item’s association with the underlying latent characteristic is strong and consistent. Rasch model’s assumption of equal discrimination of items can be met to an acceptable degree by infit values ranging from 0.7 to 1.3. There were no infit values in the current analysis that were high enough to justify removing the item from the scale. All FIES items with acceptable infits suggest a satisfactory fit of FIES to the Rasch model in the present analysis. Similar findings were reported using Rasch modeling in a FIES validation study in Sub-Saharan Africa [16].

Although the main statistic focuses on infit, the outfit statistic is also important. In contrast to infit, the outfit statistic is highly case sensitive; even a few cases with highly improbable response patterns (denials of the least severe items but affirmation of the most severe) can elevate outfit value. Items that cause cognitive issues or have distinctive meanings for small subpopulations can be identified using outfit statistics. A comparatively higher outfit value (3.6) for the “healthy” item in our analysis reflects that some respondents gave improbable responses. In line with our findings, “healthy” item was shown to be relatively higher (>2) in Ethiopia [16] and the Palestinian Territories [15] using Rasch analysis. One explanation for the high outfit value is that some respondents may have misunderstood the question, or the interviewers may have made a few mistakes in their comments or recordings. Cognitive testing, a qualitative research method, may be useful to improve this particular item by ensuring that the questions are being interpreted correctly and by refining the wording of the questions [37]. A high outfit value alone does not indicate a serious violation of the Rasch model because of an adequate infit value. An outfit value outside the acceptable range poses less threat to Rasch model validity since it captures the number of unexpected response patterns far from their latent trait [38].

The Rasch model also presupposes that all item-to-item correlations in the FIES should arise from items' shared association with the latent trait. Residual correlations were not high for any pair of items, indicating that the FIES was unidimensional, measuring only one construct of FI. In addition, the total Rasch dependability was within acceptable limits. Therefore, it follows that the FIES is a reliable and valid instrument for application in rural Bangladesh, with excellent psychometric qualities.

Equating procedure is necessary to compute internationally comparable prevalence rates of different levels of FI among the study population by calibrating the study’s results to the global FIES reference scale. Furthermore, this technique, make reliable comparisons of FIES results between subpopulations within a country [14].

When the severity of an item in a country is less than 0.35 points off the global standard, we classify it as “common” [11]. Even though the “runout” item showed a difference of 0.48, the correlation between the items was high enough to retain all eight items in the scale to produce an adequate measure of FI. We also conducted a DIF analysis to ensure that the FIES scale works similarly at the sub-national level. Since ‘whlday’ varies greatly throughout divisions, special care should be taken in formulating this item in future surveys to differentiate them more clearly.

We found some discrepancies in the conceptual ordering of FIES items, particularly those that measure less severe FI. The severity of FI was ranked in reverse order by the items “skipped” and “ateless”. This disarray had no effect on the overall FI measurement in this study because both items were classified as moderately FI [39]. In addition to internal validity, the present study examined the external validity of the FIES. Our analysis suggests a significant strong correlation between FIES and other FI measures, which aligns with the FAO study measuring the validity of FIES using Gallup World Poll (GWP) data [14].

The current study used FIES to investigate the prevalence of FI at the household level in rural Bangladesh. The level of moderate or severe FI (18.92%) reported in the current study is quite similar to that found in a telephone survey conducted in rural Bangladesh by IFPRI and Cornell University (16%) during the COVID-19 pandemic [7]. The IFPRI researcher also applied FIES tools, but FI was directly calculated based on the raw score. In contrast, we used a Rasch modeling and equating approach recommended by FAO-VOH to create internationally comparable indices of FI prevalence. However, using the HFIAS, a nationwide survey in Bangladesh indicated that 24% of households experienced mild FI, 5% experienced moderate FI, and 12% experienced severe FI [40]. This disparity might be due to the data collection period, method of FI measurement, and study design. It should be noted that our estimation of household-level FI is inconsistent with that found in GWP. The prevalence was lower than the 2019-20 FAO estimate of 31% for moderate or severe FI in all Bangladeshi households [5]. However, our results are not directly comparable to the FAO study due to the different sampling frames and sampling techniques.

Similar to previous studies conducted in Bangladesh [10], Latin America [41], and southern Ethiopia [42], the current study found that households with larger family sizes increased the likelihood of FI. This situation could be explained by the fact that when the number of family members increases, the cost of schooling, travel, clothes, and health care also increases. In low-income households, the budget for food is lowered when non-food expenditure increases. As family size increases, households’ access to food supplies declines, and FI increases. Contrary to our findings, studies conducted in rural areas of northern Ethiopia [43] and Kenya [44] revealed that Food security was more common in families with a large number of people living there compared to those with a small number of people living there. One possible explanation for this disparity is that as families expand, so does the number of people actively participate in the economy. Increased family size leads to greater participation in income-generating activities, which in turn leads to greater food security. Households expending ≤25 USD for monthly per capita food expenses were more likely to be food insecure than those households who expended more. A study conducted in Southwest Ethiopia found the similar results [45]. This could be due to a combination of low income and limited purchasing power. As the proportion of household expenses for food declines and households’ access to food similarly decreases, food diversity and quantity may be in limited supply.

Regarding the educational level of respondents, -those with lower educational levels (never attended school and primary school or below) were more likely to be food insecure than those with higher educational levels (secondary or higher). This finding was similar to findings of studies performed in Iran [46] and southwestern Ethiopia [42]. Regarding the relationship between FI and educational level, it may be stated that as respondents’ educational level increases, so do their understanding and behaviors regarding household nutrition. Better job opportunities and better economic situations due to higher educational levels might increase households’ access to nutritious food. Respondents’ lower literacy levels may affect all aspects of nutrition. Furthermore, households that lacked livestock were more likely to be food insecure than households that owned livestock. This finding is consistent with studies conducted in Northwest Ethiopia [47] and Bangladesh [10].

### 4.1 Strength and limitations

The study’s key strength is its use of a nationally representative large dataset, which enables us to generalize our findings to rural Bangladesh. We employed multiple logistic and Tobit regression models, which indicate the robustness of the study findings. Moreover, the results of one model are confirmed by the results of another, suggesting the independence of our study findings from the model selection for statistical analysis. However, the study is not beyond limitations. We could not rule out a certain degree of response bias due to the nature of the FIES data. Measurement of the seasonality of FI was also beyond the scope of the study due to the cross-sectional nature of the BIHS survey.

Similarly, the study design did not allow for investigating FI causality, although policy decisions at all levels of government can benefit from the information gleaned from a cross-sectional study [48]. Apart from these, the nature of BIHS data limited us in not evaluating the gender differences in food insecurity in rural Bangladesh. Women may be more likely to admit to food insecurity than men due to differences in gendered cultural expectations [49]. In rural Bangladesh, women are generally responsible for food preparation and distribution, and are more concerned about their families’ food insecurity.

### 4.2 The implication of the study

Our study analyses ensured the internal and external validity of the FIES in the context of rural Bangladesh. Future research may use the FIES to assess the state of FI as required for SDG 2.1.2, set national goals, and monitor the progress of interventions on FI at national and local levels.

## 5 Conclusion

The results of the current study suggest that the FIES is a reliable and valid tool for measuring food insecurity in rural Bangladesh. Our results also imply that the FIES questions may need to be rearranged in order to more correctly assess moderate and low FI. We recommended cognitive testing of the “healthy” item because of its high outfit value to possibly guide improvements of this item. In this study, 18.92% of study subjects experienced moderate or severe FI, and 0.84% experienced severe FI. A set of demographic and socioeconomic factors, i.e., geographic area, access to electricity, household ownership, access to sanitation, livestock ownership, family size, education level, and monthly per capita food expenditure, were significantly associated with FI.

## Funding

No public, private, or not-for-profit organization provided a grant in support of this study.

## Author contribution statement

Ahmed Jubayer, Md. Moniruzzaman Nayan: Conceived and designed the study; Analyzed and interpreted the data; Wrote the paper.

Saiful Islam: Performed the experiments; Contributed reagents, materials, analysis tools or data; Wrote the paper.

Abira Nowar: Contributed reagents, materials, analysis tools or data; Wrote the paper.

Md. Hafizul Islam: Performed the experiments; Analyzed and interpreted the data.

## Data availability statement

Data associated with this study has been deposited at Harvard Dataverse (https://dataverse.harvard.edu/dataset.xhtml?persistentId=doi:10.7910/DVN/NXKLZJ).

## Declaration of competing interest

The authors declare that they have no known competing financial interests or personal relationships that could have appeared to influence the work reported in this paper.

## Appendix A Supplementary data

bangladesh_Bihs_questionnaire_2018-2019_english bangladesh_Bihs_questionnaire_2018-2019_english
