# Supplementary material for: Validity of Food insecurity experience scale (FIES) for use in rural Bangladesh and prevalence and determinants of household food insecurity: An analysis of data from Bangladesh integrated household survey (BIHS) 2018-2019
Source: Heliyon. 2023 Jun 17;9(6):e17378. doi: 10.1016/j.heliyon.2023.e17378 (PMC10329118; doi:10.1016/j.heliyon.2023.e17378)
Supplement: bangladesh_Bihs_questionnaire_2018-2019_english [file mmc1.pdf]

Household Number: | | | | |

Bangladesh Policy Research and Strategy Support Program (PRSSP)
Bangladesh Integrated Household Survey Questionnaire: Round 3
November 2018 – May 2019
Survey designed and supervised by: International Food Policy Research Institute (IFPRI)
Survey administered by: Data Analysis and Technical Assistance Limited (DATA)
Household Questionnaire

Survey Household Type:	

FTF=1
FTF (Add'l) =2
National Rep=3
GFSS=7

Start time: Hour | | | Min | | | **Respondent ID** | |

Module A: Sample Household and Identification

Q. No.	Household Identification	Response
A01	Household Identification Number:	_ _ _ _ _ . _
A02	Census number (only for new area):	_ _ _
X	Household location/landmark:	
X1	Is this household located in the same location (i.e. village, union, upazilla)?	Yes………………1<br>No………………2
A03	Village (name and code):	_ _
A04	Union (name and code):	_ _
A05	Thana/ Upazilla(name and code):	_ _
A06	District (name and code):	_ _
A07	Division(name and code):	_ _
A08	GPS Coordinates: (If the household has relocated). **[Report degree, minute and second]**	**North:** ____°_____° __ **East:** ____°_____° _______
A09	Mobile phone number	_ _ _ _ _ _ _ _ _ _ _
A10	Name and Member ID(from Module B1) of the Primary Respondent (Household Head / primary male):	_ _ Name……...
A11	Name of the Household Head and Member ID:	_ _ Name……….

Q. No.	Household Identification	Response		
A12	Name and Member ID of Household Head's father (if Household Head is female report for Household Head's husband):	Dead ……………98<br>Alive but not HH member…99<br>Name…………………		
A13	Household Head's religion:	Muslim........ 1<br>Hindu 2	Christian.......3<br>Buddhist.......4	Other (specify) 5
A14	Primary language spoken:	Bangla .... 1<br>Urdu.............2	Hindi ....3<br>Tribal 4	Others (specify) 5
A15	Household's Ethnic group:	Bangali=1<br>Bihari=2<br>Sawtal=3<br>Khasia=4<br>Rakhain=5<br>Bowm=6<br>Chak=7<br>Chakma=8	Khumi=9<br>Kheyang=10<br>Lusai/pankho=11<br>Marma=12	Mru (murong)=13<br>Tonchonga=14<br>Tripura=15<br>Bonojogi=16<br>Others (specify)=17
A16	Date of the First visit (dd/mm/yy):	(see table below)		
A18	Name of Interviewer and code:…………..			
A19	Name of Supervisor and code:			
A20	Date of Data Verification (dd/mm/yy)	Day _ _ Month _ _ Year _ _		
A21	Signature of supervisor:			

A16 first visit:

visit	day		month		year	
1						
2						
3						
4						

Q. No.	Household Identification	Response
A22	Total number of members	
A23	Total number of female mebers of 15 to 49 years	
A24	Total number of children of less than 6 years	
A25	Main adult decision maker Male=1 Female=2	
**A26**	Outcome of the interview:	

Completed=1
Refused=2
No household member present at home=3
Household has shifted to a new place=4
Partially completed=5
Others=6

Household Number: | | | |

CONTENTS

Module B: Household Composition and Education (Male) 1

MODULE B1: HOUSEHOLD COMPOSITION (MALE) 1
MODULE B2: EDUCATION (MALE) 5

Module C: Employment (Male) 7

MODULE C1: AGRICULTURE BASED NON-AGRICULTURAL ACTIVITIES: (MALE) 9
MODULE C3: VOCATIONAL TRAINING / TECHNICAL EDUCATION (MALE) 10

Module D: Own Household Assets (Male) 12

MODULE D1: CURRENT HOUSEHOLD ASSETS (MALE) 12
MODULE D2: AGRICULTURAL IMPLEMENTS AND OTHER PRODUCTIVE ASSETS (MALE) 17

Module E: Savings (Male) 21

Module F: Loans (Male) 22

Module XXc: Questions on Child Marriage Law (Male and Female) 24

Module G: Roster of land and pond/water bodies owned or under operation (Male) 26

Module H: Agriculture (Male) 28

MODULE H1: AGRICULTURE PLOT UTILIZATION (MALE) 28
MODULE H2: IRRIGATION METHOD AND HARVEST (MALE) 31
MODULE H3: USAGE OF AGRICULTURAL CHEMICALS, FERTILIZERS AND PESTICIDES (MALE) 32
MODULE H4: RENTAL COST OF TOOLS, MACHINERY AND DRAFT ANIMAL (MALE) 33
MODULE H5: LABOR USAGE BY GENDER FOR CROP PLANTATION AND HARVESTING (MALE) 34
MODULE H6: POST HARVEST LABOR, ANIMAL AND TOOLS/ MACHINERY USAGE (MALE) 36
MODULE H7: FERTILIZER, SEED AND PESTICIDES PRICE IN DIFFERENT CROP SEASONS (MALE) 38
MODULE H7_A: LABOR COST: FOR SOWING SEED, WEED CONTROL, IRRIGATION AND HARVESTING CROPS. (MALE) 39
MODULE H8: LOCATION OF PURCHASE/RENT OF ANIMALS, TOOL/MACHINERIES, AGRICULTURAL LABOR, FERTILIZER, PESTICIDES ETC. (MALE) 39

Module I: Summary of Agriculture Production and Food grain stock (Male) 40

MODULE I1: SUMMARY OF AGRICULTURE PRODUCTION (CROPS, FRUITS AND VEGETABLES) (MALE) 40
MODULE I2: FOOD GRAIN STOCK AND STORAGE CAPACITY (MALE) 41
MODULE I2A: FOOD GRAIN STOCK AND STORAGE CAPACITY (CONTINUED) (MALE) 42
MODULE I3: NONPLOT FOOD PRODUCTION IN BOTH INSIDE AND OUTSIDE HOMESTEAD (MALE) 43
MODULE I4: SEEDLING/SEEDBED PRODUCTION COST (MALE) 44
MODULE HA: AGRICULTURAL TECHNOLOGIES - PADDY RICE (MALE) 45
MODULE I5: ACCESS TO TECHNOLOGIES (MALE) 52

Module J: Agricultural Extension Services and Subsidies (Male) 57

MODULE J1: ACCESS TO AGRICULTURE EXTENSION SERVICES (MALE) 57
MODULE J1A: ACCESS TO LIVESTOCK AND FISHERIES EXTENSION SERVICES (MALE) 63
MODULE J2A: GOVERNMENT AGRICULTURE INPUT SUBSIDY CARD RELATED INFORMATION (MALE) 64

Module K: Livestock and Poultry (Male) 67

MODULE K1: LIVESTOCK AND POULTRY (MALE) 67
MODULE ITMP1A: IMPROVED TECHNOLOGY AND MANAGEMENT PRACTICES (ITMP) (MALE) 70
MODULEITMP1B: IMPROVED LIVESTOCK HOUSING AND FEEDING MANAGEMENT PRACTICES (MALE) 71
MODULE ITMP1B: IMPROVED LIVESTOCK HEALTH MANAGEMENT PRACTICES (MALE) 72
MODULE ITMP1C: IMPROVED PRACTICES OF HANDLING, PRESERVATION, PROCESSING AND STORAGE MEAT, MILK, AND DAIRY PRODUCTS (MALE) 72
MODULE ITMP1D: PREPARING DIVERSIFIED DAIRY PRODUCTS (MALE) 74
MODULE K1A: AGRICULTURAL TECHNOLOGIES - DAIRY COWS (MILK) (MALE) 74
MODULE K2: LIVESTOCK AND POULTRY PRODUCTS (MALE) 82
MODULE K3: EXPENDITURE FOR LIVESTOCK AND POULTRY PRODUCTION (MALE) 83

Module L: Fisheries (Male) 84

MODULE L1: FISH/SHRIMP (REPORT FISH CULTIVATION IN PADDY FIELD OR OTHER CROP FIELDS AS WELL) (MALE) 84
MODULE L2: FISH/SHRIMP POND PRODUCTION AND INPUTS (MALE) 85
MODULE ITMP2B: IMPROVED HATCHERY MANAGEMENT TECHNOLOGY (MALE) 86
MODULE ITMP2C: NURSERY TECHNOLOGIES (MALE) 88
MODULE ITMP2A: TILAPIA/ SILVER CARP/KATLA AND OTHER VARIETIES OF FISH FARMING TECHNOLOGIES (MALE) 92
MODULE L2A: AGRICULTURAL TECHNOLOGIES - FISHPOND AQUACULTURE (MALE) 95
MODULE L2B: POND AREA OR DIAGRAM (THE WHOLE FARM) (MALE) 102
MODULE L2C: POND AREA MEASUREMENT (MALE) 103

Module M: Marketing of Agriculture, Livestock and Fisheries Products (Male) 105

MODULE M1: MARKETING OF PADDY, RICE, BANANA, MANGO AND POTATO (MALE) 105
MODULE M2: MARKETING OF LIVESTOCK, JUTE, WHEAT, PULSES, FISH, FRUITS, VEGETABLES, ETC. (MALE) 106

Module N: Non-agricultural Enterprises (Male) 108

Module XX: Early Marriage (Female) 111

MODULE XXA: EARLY MARRIAGE (FEMALE) 111
MODULE XXB: EARLY MARRIAGE (FEMALE) 113

Module O: Food Consumption (Female) 115

MODULE O1: PURCHASES, HOME PRODUCTION AND OTHER SOURCES (FEMALE) 115
MODULE O2: HOUSEHOLD FOOD INVENTORY ON THE DAY OF SURVEY (FEMALE) 124
MODULE O3: FOOD CONSUMPTION FROM PURCHASES, HOME PRODUCTION AND OTHER SOURCES: RECALL PERIOD 7 DAYS (FEMALE) 124

Module P: Non-food Expenditure (Male) **125**

MODULE P1: NON-FOOD EXPENDITURE MONTHLY RECALL (MALE) 125
MODULE P2: NON-FOOD EXPENDITURE ANNUAL RECALL (MALE) 128

Module Q: Housing (Male) **136**

Module R: Sanitation and Water (Male) **140**

MODULE R2: DOMESTIC ANIMALS AND POULTRY HABITATION AND HYGIENE MANAGEMENT (MALE) 147

Module S: Access to Facilities (Male) **150**

Module T: Economic Events/Shocks (Male) **152**

MODULE T1B: NEGATIVE SHOCKS (MALE) 152
MODULE T1C: SEVERE DISASTER (MALE) 155
MODULE T1C: INSURANCE (MALE) 155
MODULE T2: POSITIVE ECONOMIC EVENTS (MALE) 156

Module U: Participation in Social Safety Net Programs (Male) **157**

MODULE UA: PROGRAM PARTICIPATION IN FOOD FRIENDLY PROGRAM (KHADDO BANDHOB / TK 10/KG RICE PROGRAM) (MALE) 159

Module V: Migration, Remittances, Transfers and Other Income (Male) **164**

MODULE V1: PROFILE OF CURRENT MIGRANTS (DOMESTIC AND INTERNATIONAL) (MALE) 164
MODULE V2: REMITTANCE IN (MALE) 167
MODULE V3: REMITTANCE OUT (MALE) 169
MODULE V4: OTHER INCOME HOUSEHOLD (MALE) 170

Module W: Anthropometry, Health and Illness (Female) **171**

MODULE W1: ANTHROPOMETRY (FEMALE) 171
MODULE W2: ANTHROPOMETRY-CHILDREN (FEMALE) 174
MODULE W3: HEALTH (FEMALE) 175
MODULE W4: ILLNESS (FEMALE) 176
MODULE W5: ILLNESSES (RECORD FOR THE LAST 4 WEEKS) (FEMALE) 177
MODULE W5A: CHRONIC ILLNESS (FEMALE) 178

Module X: Household Food Consumption and Food Security (Female) **179**

MODULE X-1.1: HOUSEHOLD FOOD CONSUMPTION (RECALL-1) (FEMALE): 179
MODULE X-2.1: INTRA-HOUSEHOLD FOOD DISTRIBUTION (FEMALE) 180
MODULE X2A: CONSUMPTION OF FOOD PREPARED OUTSIDE (FEMALE) 181
MODULE X3: HOUSEHOLD FOOD HABIT (FEMALE) 182
MODULE X4: BAD TIME (KEEPING INCOME IN MIND). RECALL PERIOD: LAST 12 MONTHS (2017): (MALE AND FEMALE) 185
MODULE X5: FOOD SECURITY AND RESILIENCE (FEMALE) 186

Module Y: Nutrition Practices and Services (Female) 189

MODULE Y1: INFANT AND YOUNG CHILD FEEDING (IYCF) PRACTICES AND USE OF MICRONUTRIENTS (FEMALE) 189
MODULE Y2: NUTRITION (IYCF) KNOWLEDGE OF RESPONDENT MOTHERS (FEMALE) 203
MODULE Y3: AWARENESS-TRIAL-ADOPTION OF SENTINEL PRACTICES (FEMALE) 207
MODULE Y4: IMMUNIZATION AND HEALTH STATUS OF YOUNG CHILDREN (<2 YEARS) (FEMALE) 209
MODULE Y5: NUTRITION RELATED PRENATAL CARE DURING PREGNANCY WITH YOUNGEST CHILD (FEMALE) 216
MODULE Y7: NNP SERVICES USAGE BY CHILDREN UNDER 2 YEARS OF AGE (FEMALE) 222
MODULE Y8: EXPOSURE TO NUTRITION INFORMATION FROM HEALTH WORKERS AND MEDIA (FEMALE) 225
MODULE Y6A: ACCESS TO COMMUNITY CLINICS AND USE OF COMMUNITY CLINIC (FEMALE) 226

Module Z: Women's Status (Female) 227

MODULE Z1: WORK EARNINGS AND EXPENSES (FEMALE) 227
MODULE Z2: FREEDOM OF MOBILITY (FEMALE) 230
MODULE Z3: REPRODUCTIVE DECISIONS (FEMALE) 231
MODULE Z4: DOMESTIC VIOLENCE, ABUSE AND THREATS (FEMALE) 232
MODULE Z5: WIFE'S ASSETS THAT HAD BEEN BROUGHT TO MARRIAGE (FEMALE) 235

Module WE: Women's Empowerment in Agriculture (WEAI) Index (Male and Female) 236

MODULE WEA: INDIVIDUAL IDENTIFICATION (MALE AND FEMALE) 236
MODULE WE2: (DIMENSION 1): ROLE IN HOUSEHOLD DECISION-MAKING AROUND PRODUCTION AND INCOME GENERATION (MALE AND FEMALE) 237
MODULE WE3A: (DIMENSION 2) ACCESS TO PRODUCTIVE CAPITAL (MALE AND FEMALE) 238
MODULE WE3B: AGRICULTURAL EXTENSION (MALE AND FEMALE) 239
MODULE WE3C: (DIMENSION 3) INCOME (MALE AND FEMALE) 240
MODULE WE3D: ACCESS TO LOANS (MALE AND FEMALE) 242
MODULE WE4: INDIVIDUAL LEADERSHIP AND INFLUENCE IN THE COMMUNITY (MALE AND FEMALE) 243
MODULE WE4 CONTINUED: GROUP MEMBERSHIP (MALE AND FEMALE) 245
MODULE WE5A: DECISION MAKING (MALE AND FEMALE) 246
MODULE WE5B: MOTIVATION FOR DECISION-MAKING (MALE AND FEMALE) 247
MODULE WE5C: DECISION MAKING (MALE AND FEMALE) 250
MODULE WE6A: TIME ALLOCATION (MALE AND FEMALE) 251
MODULE WE6B: SATISFACTION WITH TIME ALLOCATION (MALE AND FEMALE) 253
MODULE WE7B: PARENT'S INFORMATION (MALE AND FEMALE) 255
MODULE WE7C: PARDA INFORMATION (MALE AND FEMALE) 257

Household Number: | | | |

DEFINITION OF RESPONDENTS

It is very important to know what is meant to be primary male decision maker and primary female decision maker since in most of the modules they will be the two main respondents.

Primary male decisionmaker/main male respondent: The primary male decisionmaker is the male member who makes more social and economic decisions concerning the household, compared to other male members, and is at least 18 years old.

Primary female decision maker/main female respondent: The primary female decisionmaker is the female member who makes more social and economic decisions concerning the household, compared to other female members, and is at least 18 years old.

The household members themselves will select their primary male and primary female respondents. Most of the responses will be given by the primary male / primary female respondents, although there are modules where other members of the household will be asked questions as well. In most of those cases, they must be more than 18 years.

CONSENT OF RESPONDENT

Good morning/afternoon. I am ________ from the Data Analysis and Technical Assistance Limited (DATA), a Bangladeshi research organization based in Dhaka. Together with the International Food Policy Research Institute (IFPRI), we are conducting a survey that will provide IFPRI with necessary information to carry out research that is designed to help promote the welfare of Bangladeshis; particularly, to improve food consumption and nutrition of the people and women's status, and to enhance agricultural development and income generation. Your household has been chosen by a random selection process.

We are inviting you to be a participant in this study. We value your opinion and there are no wrong answers to the questions we will be asking in the interview. We will use approximately 6-8 hours of your time to collect all the information. If you prefer, we can do the interview in several visits. In that case, we will fix a time with you or other respondent of your household for the next visit and come accordingly. There will be no cost to you other than your time. There will be no risk as a result of your participating in the study. Your participation in this research is completely voluntary. You are free to withdraw your consent and discontinue participation in this study at any time.

This study is conducted anonymously. You will only be identified through code numbers. Your identity will not be stored with other information we collect about you. Your responses will be assigned a code number, and the list connecting your name with this number will be kept in a locked room and will be destroyed once all the data has been collected and analyzed. Any information we obtain from you during the research will be kept strictly confidential.

Your participation will be highly appreciated. The answers you give will help provide better information to policy-makers, practitioners and program managers so that they can plan for better services that will respond to your needs.

The researcher read to me orally the consent form and explained to me its meaning. I agree to take part in this research. I understand that I am free to discontinue participation at any time if I so choose, and that the investigator will gladly answer any question that arise during the course of the research.

Household Number: | | | |

AS APPLICABLE, CHECK AND SIGN THE CONSENT BOX BELOW

Sl No.	Modules	MID of respondent	Consent of respondent	Codes
1	FOR THE ADULT RESPONDENTS FOR THE HOUSEHOLD & DWELLING CHARACTERISTICS MODULES<br>[NAME], do you agree to participate in the survey?			
	NAME ……………………………………………………..			Respondent agreed yes=1 No=2
2	FOR THE RESPONDENTS FOR THE FOOD SECURITY MODULE<br>[NAME], do you agree to participate in the survey?			
	NAME ……………………………………………………..			Respondent agreed yes=1 No=2
3	FOR THE RESPONDENTS FOR THE WOMENS NUTRITION MODULE<br>[NAME], do you agree to participate in the survey?	**MID of respondent**	**Consent of respondent**	**Codes**
	NAME ……………………………………………………..			Respondent agreed yes=1 No=2
	NAME ……………………………………………………..			
	NAME ……………………………………………………..			
	NAME ……………………………………………………..			
	NAME ……………………………………………………..			
4	FOR THE RESPONDENTS FOR PRIMARY CAREGIVERS OF CHILDREN ELIGIBLE FOR THE CHILDREN'S NUTRITION MODULE<br>[NAME], do you agree to participate in the survey?	**MID of respondent**	**Consent of respondent**	**Codes**
	NAME ……………………………………………………..			Respondent agreed yes=1 No=2
	NAME ……………………………………………………..			
	NAME ……………………………………………………..			
	NAME ……………………………………………………..			
	NAME ……………………………………………………..			
5	FOR THE FEMALE RESPONDENTS FOR THE EMPOWERMENT IN AGRICULTURE MODULES<br>[NAME], do you agree to participate in the survey?	**MID of respondent**	**Consent of respondent**	**Codes**
	NAME ……………………………………………………..			Respondent agreed yes=1 No=2
6	FOR THE MALE RESPONDENTS FOR THE EMPOWERMENT IN AGRICULTURE MODULES<br>[NAME], do you agree to participate in the survey?	**MID of respondent**	**Consent of respondent**	**Codes**
	NAME ……………………………………………………..			Respondent agreed yes=1 No=2
7	FOR THE RESPONDENTS FOR HOUSEHOLD CONSUMPTION EXPENDITURE MODULE MODULE<br>[NAME], do you agree to participate in the survey?	**MID of respondent**	**Consent of respondent**	**Codes**
	NAME ……………………………………………………..			Respondent agreed yes=1 No=2

	NAME …………………………………………………..			
	NAME …………………………………………………..			
	NAME …………………………………………………..			
	NAME …………………………………………………..			
8	FOR THE RESPONDENTS FOR SANITARY NAPKIN USE MODULE<br>[NAME], do you agree to participate in the survey?	**MID of respondent**	**Consent of respondent**	**Codes**
	NAME …………………………………………………..			Respondent agreed yes=1 No=2
	NAME …………………………………………………..			
	NAME …………………………………………………..			
	NAME …………………………………………………..			
	NAME …………………………………………………..			
9	FOR THE RESPONDENTS ELIGIBLE FOR THE EARLY MARRIAGE MODULE<br>[NAME], do you agree to participate in the survey?	**MID of respondent**	**Consent of respondent**	**Codes**
	NAME …………………………………………………..			Respondent agreed yes=1 No=2
	NAME …………………………………………………..			
	NAME …………………………………………………..			
	NAME …………………………………………………..			
	NAME …………………………………………………..			
10	FOR THE RESPONDENTS ELIGIBLE FOR THE REPRODUCTIVE DECISION MODULE<br>[NAME], do you agree to participate in the survey?	**MID of respondent**	**Consent of respondent**	**Codes**
	NAME …………………………………………………..			Respondent agreed yes=1 No=2
	NAME …………………………………………………..			
	NAME …………………………………………………..			
	NAME …………………………………………………..			
	NAME …………………………………………………..			
11	FOR THE RESPONDENTS ELIGIBLE FOR THE DOMESTIC VIOLENCE MODULE<br>[NAME], do you agree to participate in the survey?	**MID of respondent**	**Consent of respondent**	**Codes**
	NAME …………………………………………………..			Respondent agreed yes=1 No=2
	NAME …………………………………………………..			
	NAME …………………………………………………..			

Household Number: | | | | |

	NAME …………………………………………………..			
	NAME …………………………………………………..			

Contact Person:
Name of the Principal Investigator (PI): Dr. Akhter Ahmed
PRSSP/IFPRI
Address: House 10A, Road 35, Gulshan 2, Dhaka 1212
Tel: 989-8686; E-mail of PI: a.ahmed@cgiar.org

Agreed to participate ☐ Did not agree ☐

Statement of the enumerator,

I am an interviwer of the above mentioned research. I have read the consent form about which the participant is aware of. Being aware of the above mentioned description, the participant has kindly agreed to participate and put a tick mark on the box above.

Interviwer's signature_________________________ Date _____/_____/_____

DEFINITION OF HOUSEHOLD

A household is a group of people who live together and take food from the "same pot." In our survey, a household member is someone who has lived in the household at least 6 months, and at least half of the week in each week in those months.

Even those persons who are not blood relations (such as servants, lodgers, or agricultural laborers) are members of the household if they have stayed in the household at least 3 months of the past 6 months and take food from the "same pot." If someone stays in the same household but does not bear any costs for food or does not take food from the same pot, they are not considered household members. For example, if two brothers stay in the same house with their families but they do not share food costs and they cook separately, then they are considered two separate households.

Generally, if one person stays more than 3 months out of the last 6 months outside the household, they are not considered household members. We do not include them even if other household members consider them as household members.

Exceptions to these rules should be made for:

Consider as household member

- A newborn child less than 3 months old.
- Someone who has joined the household through marriage less than 3 months ago.
- Servants, lodgers, and agricultural laborers currently in the household and will be staying in the household for a longer period but arrived less than 3 months ago.
- If any household member resides outside the household for the pursuit of education, then that person's information will have to be recorded in the Household Composition module

Do not consider as household member

- A person who died very recently though stayed more than 3 months in last 6 months.
- Someone who has left the household through marriage less than 3 months ago.
- Servants, lodgers, and agricultural laborers who stayed more than 3 months in last 6 months but left permanently.

This definition of the household is very important. The criteria could be different from other studies you may be familiar with, but you should keep in mind that you should not include those people who do not meet these criteria. Please discuss any questions with your supervisor.

Household Number: | | | | |

Module B: Household Composition and Education (Male)

Module start time: Hour | | | Minu | | | **Respondent ID:** | | : | | Yes ... 1 No..... 2

Module B1: Household Composition (Male)

Note: For BIHS Round 3 households, record information of the former members first, and then add the new members of this round. For GFSS households, record in the usual way.

Start with taking information about the old members as of 2015(midline) followed by the new members in this round [For BIHS round 3 only]Member ID	Name	Status of the member in the current round [if the response code is 7 and above, do not fill out the rest of the columns]	Gender M: 1 F: 2	Age** (comp-lete years)	Date of birth			How was birth date verified?<br><br>Birth registration certificate …..1<br>National ID card …..2<br>Respondent has written the date somewhere….3<br>Recalled date from past event….4<br>Respondent guessed the date ….5	Relation of primary respondent to Household Head	Marital Status If answer is "1">>B1_14	What was your age when you got married? (If married more than once, record the age when first married)	What was your age when your first child (living or deceased) was born? No children ……99
		Code 8	Code↑	Age	Day	Month	Year	Code↑	Code 1	Code 2	Age	Age
M ID	Name	Mem_ Stat	B1_01	B1_ 02	B1_15a	B1_15b	B1_15c	B1_16	B1_ 03	B1_ 04	B1_04a	B1_04b

Household Number: | | | | |

Module B1: Household Composition (Male) (Continued)

"Did [NAME] stay here last night?<br>YES=1<br>NO=2"	Since the midline was conducted (2015), whether (name) was abroad more than 6 months<br>Yes…1<br>No…2 >>B1_07	Why did (name) return to the household from abroad?	Literacy	Education (Highest class passed)<br>If answer is 99>> B1_10	Have you participated in any educational programs?	Currently (2018) attending school/ college?<br>Yes ....1; No 2	Current main Occupation<br>if responses are code : 81-86>> B1_13a	Do you work in rural /urban area?<br>Rural 1 ;<br>Urban 2	Location of employment	What is your main source of earnings?<br><br>Record the two main sources
	Code ↑	Code 3	Code 4	Code 5	Code 9	Code ↑	Code 6	Code ↑	Code 7	Code 10
B1_14	B1_05	B1_06	B1_ 07	B1_ 08	B1_08a	B1_09	B1_ 10	B1_11	B1_ 12	B1_ 13a

Note: **Write complete years. For example if age is 18 years and 9 months, write only 18 years.

Module End Time:

Hour			Minute		

Household Number: | | | | |

Code list for Module B1:

Code 1: Relationship

Relationship with primary respondent
- Primary respondent ... 1
- Primary respondent Husband/wife ... 2
- Son/daughter ... 3
- Daughter/son -in-law ... 4
- Grandson/daughter ... 5
- Father/mother ... 6
- Brother/sister ... 7
- Niece/Nephew ... 8
- Primary respondent's cousin ... 9

Relationship with primary respondent's husband/wife
- Father-in-law/mother-in-law ... 10
- Brother/Sister-in-law ... 11
- Husband/wife's niece/nephew ... 12
- Primary respondent's husband/wife's cousin ... 13

Other relative/non relative
- Other relative ... 14
- Permanent servant ... 15
- Other Non relative/friends ... 16

Code 2: Marital status code
- Unmarried (never married) ... 1
- Married ... 2
- Widow/widower ... 3
- Divorced ... 4
- Separated/Deserted ... 5

Code3: Reason for returning from abroad
- Homesick ... 1
- Due to illness ... 2
- End of employment contract ... 3
- Job loss ... 4
- Disagreement with authorities ... 5
- Due to Economic Recession ... 6
- Other (specify) ... 7

Code 4 : Literacy
- Cannot read and write ... 1
- Can sign only ... 2
- Can read only ... 3
- Can read and write ... 4

Code 5: Education (Highest class passed)
- Never attended school ... 99
- Reads in class I ... 0
- Completed class I ... 1
- Completed class II ... 2
- Completed class III ... 3
- Completed class IV ... 4
- Completed class V ... 5
- Completed class VI ... 6
- Completed class VII ... 7
- Completed class VIII ... 8
- Completed class IX ... 9
- Completed Secondary School/Dakhil10
- Completed Higher Secondary/Alim..12
- BA/BSC pass/Fazil ... 14
- BA/BSC honors/Fazil ... 15
- MA/MSC and above/Kamil ... 16
- SSC Candidate ... 22
- HSC Candidate ... 33
- Preschool class (general) ... 66
- Preschool (mosque based) ... 67
- Medical/MBBS ... 71
- Nursing ... 72
- Engineer ... 73
- Diploma Engineer74
- Vocational/Technical Education75
- Other (specify)76

Code 6: Main Occupation

Wage Labor
- Agricultural day labor ... 1
- Earth work (govt. program) ... 2
- Earth work (other) ... 3
- Sweeper ... 4
- Scavenger ... 5
- Tea garden worker ... 6
- Construction labor ... 7
- Factory worker ... 8
- Transport worker (bus/truck helper) ... 9
- Apprentice ... 10
- Other wage labor (specify) ... 11

Salaried worker
- Government/ parastatal ... 12
- Service (private sector) ... 13
- NGO worker ... 14
- House maid ... 15
- Teacher (GoB-Primary school) ... 16
- Teacher(Non GoB Primary school) ... 17
- Teacher (GoB High school) ... 18
- Teacher (Non-GoB High school) ... 19
- Teacher (college, university) ... 20
- Other salaried worker(specify) ... 21
- Work as a political party worker ... 100

Self-employment
- Rickshaw/van pulling ... 22
- Driver of motor vehicle ... 23
- Tailor/seamstress ... 24
- Blacksmith ... 25
- Potter ... 26
- Cobbler ... 37
- Hair cutter ... 28
- Clothes washer ... 29
- Porter ... 30
- Goldsmith/silversmith ... 31
- Repairman (appliances) ... 32
- Mechanic (vehicles) ... 33
- Plumber ... 34
- Electrician ... 35

Self-employment (continued)
- Carpenter ... 36
- Mason ... 37
- Doctor ... 38
- Rural physician ... 39
- Midwife ... 40
- Herbal doctor/Kabiraj ... 41
- Engineer ... 42
- Lawyer/deed writer/Moktar ... 43
- Religious leader (Imam/Muazzem/Khadem/Purohit) ... 44
- Lodging master ... 45
- Private tutor/house tutor ... 46
- Beggar ... 47

Trader
- Small trader (roadside stand or stall) ... 50
- Medium trader (shop or small store) ... 51
- Large trader (large shop or whole sale) ... 52
- Fish Trader ... 53
- Contractor ... 54

Production
- Food Processing ... 55
- Small industry ... 56
- Handicrafts ... 57

Livestock Poultry related work/occupation
- Milk collector ... 58
- Livestock Vet medicine seller ... 59
- Livestock Feed supplier ... 60
- Commercially feed producer ... 61
- Animal Breeder ... 62
- Veterinary/paravet doctor ... 63

Farming
- Working own farm (crop) ... 64
- Share cropper/tenant ... 65
- Homestead farming ... 66
- Fisherman (using non owned/not leased water body) ... 67
- Raising fish / fish pond ... 68
- Raising poultry ... 69
- Raising livestock ... 70
- Dairy production/ dairy farming ... 71
- Other self-employed (specify) ... 72

Non-earning occupation
- Student ... 81
- Housewife ... 82
- Retired ... 83
- Child(age <12 no study/ work) ... 84
- Physically/mentally challenged ... 85
- Jobless ... 86
- Don't know ... 99

Household Number: | | | | |

Code 7: Location of employment	Code 8: Status of the member in the current round	Code 9: Educational Programs	Code 10: Main Source of Earnings
This village/ward.........................1<br>Other village/ward in this union ..2<br>Other union in this thana ....3<br>Other thana in this district ....4<br>Other district 5	Member in both previous and current round .... 0<br>New food sample and member in current round................. 66<br><br>***Not a member in the previous round but member in the current round***<br>New Member (New Born)..1<br>New Member through marriage ....2<br>New Member upon return from divorce or separation......3<br>Household merged/combined...4<br>Other reasons (Permanent) ....5<br><br>***Was a member in the previous round but no longer one in the current round***<br>Residing elsewhere for the pursuit of studies ....6<br>Death..7<br>Married and left household ....8<br>Divorced and left household...9<br>Household split ....10<br>Left household for employment ....11<br>Other reasons for leaving the household 12	Have not participated in any programs ....1<br>Food for Education (FFE) ....2<br>Tk 20 Scholarship..3<br>Anondo School Scholarship ....4<br>Tk 100 (Tk 125) Scholarship..5<br>School Feeding Program (Biscuit)..6<br>School Feeding Program (Cooked Food like Khichuri).............7<br>Secondary School Scholarship ....8<br>High School Scholarship ....9<br>Bachelor's/Master's Scholarship ………….10	Physical Labor(agri) ....1<br>Physical Labor(non-agri) ....2<br>Salaried Employee ....3<br>Self Employed (Agri) ....4<br>Self Employed (fish farming) ....5<br>Self Employed (fish capture) ....6<br>Self Employed (livestock/poultry) ....7<br>Self Employed (other non-agri) ....8<br>Land rent(cash/share) ....9<br>House Rent..10<br>Other rent(shop/productive asset) ....11<br>Business(purchase-sell) ....12<br>Business(production) ....13<br>Loan business(use of interest) ....14<br>Remittance (Country) ....15<br>Remittance (Abroad) ....16<br>Others..17<br>No source of income 18

Household Number: [ ] [ ] [ ] [ ]

Module B2: Education (Male)

Module start time: Hour [ ] [ ] Minu [ ] [ ] **Respondent ID:** [ ] Consent : [ ] yes .... 1 no 2

Report for all children/member of age 6-25 years or those attending or have attended primary/secondary school/madrasa/university.

MID	Name	Ever attend school/ Madrasa/ college/ university?<br>Yes…1>>B2_03<br>No….2	Why did you never attend school/ Madrasa/ college/ university?<br>Next child	When did you first attend school /madrasa/college/ university?	Class you were admitted to when first attending school	Type of last school/ madrasa /college/ university attended	How far is the school/ madrasa/ college/ university from your house?		Were you enrolled in school in 2017?<br>Yes…1<br>No 2	Did/do you go to school in 2018?<br>Yes…1<br>No…2>> B2_12a	What type of school?<br>Govt…. 1<br>Private …..2	Monthly Fees at the school	Does the student receive any private coaching?<br>Yes…1<br>No…2	If private coaching is availed, what amount is spent on it every month?	Currently in what type of program are you participating?	Class attending/ed in 2018	Are you repeating the class (in 2017 and 2018)<br>Yes..1No ....2<br>N/A ....9<br>>> next child	Programs participation before 2018<br>Note: report last two programs in case of multiple programs participation		Which year did the child stop attending school?<br>*not applicable ...9999	Why did the child stop attending school?
		Code ↑	Code 1	Year	Code 2	Code 3	km	min	Code ↑	Code ↑	Code ↑	Tk	Code ↑	Tk	Code 4	Code 2	Code ↑	Code 4		Year	Code 1
**MID**	**Name**	**B2_01**	**B2_02**	**B2_03**	**B2_04**	**B2_05**	**B2_06a**	**B2_06b**	**B2_07**	**B2_08**	**B2_08a**	**B2_08b**	**B2_08c**	**B2_08d**	**B2_09**	**B2_10**	**B2_11**	**B2_12a**	**B2_12b**	**B2_13**	**B2_14**

Note: * Report "9999" (not applicable) in Column B2_13, if attended school in 2011 (i.e. response in B2_10 is "2"), then Go to next row for next child.
Interviewer: Please find the code list for this module B2 in the next page.

Module end time: Hour [ ] [ ] Min [ ] [ ]

Household Number:

Code list for Module B2:

Code 1: Reason not attending/stop attending school (applicable for B2_02 and B2_14)

Age/sickness/unwillingness perspective:
Below school/madrasa age............................1
Sick/disabled child..2
Child didn't want to attend school.................3
Teachers do not teach well............................4
Parents don't want to send children to school...5
Examination not passed.................................6

Distance perspective:
No school/madrasa nearby............................7
Transport/communication problem 8

Non-ability perspective:
Inability to bear schooling expenses /inability to buy school uniform....................9
Engaged in household work..........................10
Engaged in family business/agriculture.........11
Works elsewhere for income.........................12
Does not work now but looking for work……23

Stipend perspective:
Insufficient amount of stipend money /education allowance 13
Not getting stipend, so withdrawn from school/madrasa.....................................14

Gender perspective:
Don't like to send girls to school 15
There are no female teacher in school...........16
No only boys' or only girls' school 17
Boys tease girls/don't like girls.....................18
Environment of school is not safe 19
No separate latrine for female students 20
Due to marriage...21
Other (specify) 22

Code2: Class attended (applicable for B2_04 and B2_10)

Never attended school........................99
Reads in class I..................................0
Completed class I...............................1
Completed class II.............................2
Completed class III 3
Completed class IV 4
Completed class V..............................5
Completed class VI 6
Completed class VII...........................7
Completed class VIII..........................8
Completed class IX 9
Completed Secondary School/Dakhil.10
HSC/Alim First Year..........................11
HSC/Alim Second Year…. 12
BA/BSC/Fazil First Year....................13
BA/BSC/Fazil Second Year 14
BA/BSC/Fazil Third Year..................15
BA/BSC/Fazil Fourth Year 16
MA/MSC and above/Kamil................17
SSC Candidate 22
HSC Candidate..................................33
Preschool class (general) 66
Preschool (mosque based) 77
Medical/MBBS 71
Nursing...72
Engineer..73
Diploma Engineer 74
Vocational/Technical Education.........75
Other (specify) 76

Code 3: Type of school attended/attending

Govt. aided.................................1
Private(registered)school............2
Private(non-registered)school 3

Ananda school............................4
BRAC run NGO school 5
Other NGO run school................6
Aliyah madrasa 7
Quomi madrasa 8
College/university.......................9
Nurania/hafezia Madrasa 10
Other.. 11
Kindergarten 12

Code 4: Type of program (applicable for B2_09, B2_12a and B2_12b)

Not participated/ ing in any program 1
Food for education (FFE) 2
Participated in Tk 20 stipend program.......... 3
Ananda school stipend program 4
Tk 100 (Tk 125) stipend program................. 5
School feeding program(biscuit) 6
School feeding program(cooked food for example-khichuri/singara)............................ 7
Secondary school student stipend program... 8
Higher secondary stipend program 9
Bachelor's/Master's Scholarship ………….10

Module C: Employment (Male)

Module start time: Hour [ ][ ] Minu [ ][ ] **Respondent ID:** [ ] Consent : Conse [ ] yes ....1 no......2

=> Ask about all members aged 6 years and above.

Collect information on all type of economic work performed by each HH member in the last 7 days. If any member is involved in more than one economic activity, use one row for each type of economic work.

Recall period: Last 7 days, if not mentioned otherwise.

MID	Name	What was____ employment status in the past 7 days?<br><br>if 1 or 2 >> C05<br>if 3 >> C02<br>if 4 >> C04<br><br>if 5-12 -> next row	Why did ____ not work in the last 7 days?	When will ____ return to work?<br><br>This week .... 1<br>Next week ... 2<br>After 2 weeks……...3<br>Don't know.. 9<br><br>For any of the above response Go to C05	How long have you been unemployed / looking for work?<br><br>< 1 month .... 1<br>1-<3 months............ 2<br>3-<6 months............. 3<br>6-<9 months............. 4<br>9-<12 months........... 5<br>more than 1 year .... 6<br>Looking for job for the first time .... 7<br><br>Go to next member	Information on economic activities in last 7 days									Monthly salary OR Average monthly income from this activity?
						Occupation /economic activity in last 7 days	Activity serial<br><br>Serial number	For____ (activity) on average for how many days last week did you work?	For____ (activity) on average for how many hours a day did you work?	What is the nature of the ____ work/ activity performed?<br><br>Daily/weekly wage.............1<br>Salary ....2>>C14<br>Self-employed ....3>>C14<br>Work without pay ....4>> next row	What was_______ daily wage / salary?				
											Cash<br><br>If none write "0"	Value of meals/ any other pay In-kind.<br><br>If none write "0"	Have you sold any income received in kind for wages /salaries?<br><br>Yes...1<br>No....2>>C14	How much money was obtained by selling the "kind" received wages & salaries?	
		[Code 1] ↓	[Code-2] ↓	[Code] ↑	[Code] ↑	[Code 3] ↓	No.	[days]	[hours]	[Code] ↑	[Tk]	[Tk]		[Tk]	[Tk]
**MID**	**Name**	**C01**	**C02**	**C03**	**C04**	**C05**	**C06**	**C07**	**C08**	**C09**	**C10**	**C11**	**C12**	**C13**	**C14**

Note: Interviewer: Please find the code list for this section in the next page.

Household Number: | | | |

Code list for Module C: Employment

Code 1: Employment status

Employment status	Code
Worked for pay (salary, wage, self-employed)	1
Worked without pay (apprentice, family business)	2
Did not work but have a job	3
Did not work but looked for a job	4
**Did not work because:**	
Only studied (student)	5
Too young (not student)	6
Too old/retired	7
Home/household work (includes live-in servant)	8
Disabled/invalid	9
Don’t need to	10
Other (specify)	11

Code 2: Reasons for not working in the last 7 days

Reason	Code
Sick	1
Vacation	2
Hartal/strike	3
Taking care of household matters	4
Taking care of family members	5
Other (specify)	6

Code 3: Occupation/economic activity

Occupation	Code
**Wage Labor**	
Agricultural day labor	1
Earth work (govt program)	2
Earth work (other)	3
Sweeper	4
Scavenger	5
Tea garden worker	6
Construction labor	7
Factory worker	8
Transport worker (bus/truck helper)	9
Apprentice	10
Other wage labor (specify)	11
**Salaried worker**	
Government/ parastatal	12
Service (private sector)	13
NGO worker	14
House maid	15
Teacher (GoB-Primary school)	16
Teacher (Non GoB Primary school)	17
Teacher (GoB High school)	18
Teacher (Non-GoB High school)	19
Teacher (college, university)	20
Other salaried worker (specify)	21
**Self-employment**	
Rickshaw/van pulling	22
Driver of motor vehicle	23
Tailor/seamstress	24
Blacksmith	25
Potter	26
Cobbler	27
Hair cutter	28
Clothes washer	29
Porter	30
Goldsmith/silversmith	31
Repairman (appliances)	32
Mechanic (vehicles)	33
Plumber	34
Electrician	35
Carpenter	36
Mason/Construction Rod Welder	37
Doctor	38
Rural physician	39
Midwife	40
**Self-employment (continued)**	
Herbal doctor/Kabiraj	41
Engineer	42
Lawyer/deed writer/Moktar	43
Religious leader (Imam/Muazzem/ Khadem/Purohit)	44
Lodging master	45
Private tutor/house tutor	46
Beggar	47
**Trader**	
Small trader (roadside stand or stall)	50
Medium trader (shop or small store)	51
Large trader (large shop or whole sale)	52
Fish Trader	53
Contractor	54
**Production**	
Food Processing	55
Small industry	56
Handicrafts	57
**Livestock Poultry related work/occupation**	
Milk collector	58
Livestock Vet medicine seller	59
Livestock Feed supplier	60
Commercially feed producer	61
Animal Breeder	62
Veterinary/paravet doctor	63
**Farming**	
Working own farm (crop)	64
Share cropper/tenant	65
Homestead farming	66
Fisherman (using non owned/not leased water body)	67
Raising fish / fish pond	68
Raising poultry	69
Raising livestock	70
Dairy production/ dairy farming	71
Other self-employed (specify)	72

Household Number: | | | |

Module C1: Agriculture based non-agricultural activities: (Male)

Last 12 months December 1, 2017 to November 30, 2018

C1_00: In the past 12 months, has anyone from the household worked in the sectors listed below? [ ] Yes…1 No …2

		How has any of your hh member worked mainly in the mentioned activities in last 12 months<br>Self-employed................ 1<br>Rent out equipment......... 2<br>As labor or salaried employee .... 3<br>Did not work.................. 4<br>>> next activity	1st Respondent				2nd Respondent				3rd Respondent			
			Which member of the household worked in the sectors listed below?	How many of the past 12 months did you work on this activity?	How many days of the month did you work on this activity?	Total income in last 12 months	Which member of the household worked in the sectors listed below?	How many of the past 12 months did you work on this activity?	How many days of the month did you work on this activity?	Total income in last 12 months	Which member of the household worked in the sectors listed below?	How many of the past 12 months did you work on this activity?	How many days of the month did you work on this activity?	Total income
**Agricultural Activities in a Non Agricultural Farm**	**Code**	**Code ↑**	**MID**	**Month**	**Day**	**Tk**	**MID**	**Month**	**Day**	**Tk**	**MID**	**Month**	**Day**	**Tk**
		**C1**	**C2**	**C3**	**C4**	**C5**	**C6**	**C7**	**C8**	**C9**	**C10**	**C11**	**C12**	**C13**
Rice Mill	1													
Wheat/ Maize Mill	2													
Spice Processing mill	3													
Oil mill /ghaani	4													
Sugar mill	5													
Food Processing Plant	6													
Cold storage	7													
Agricultural handling/packaging	8													
Agricultural marketing	9													
Tractor/ Power Tiller Operator	10													
Irrigation Machinery Operator	11													
Briquette Urea Applicator	12													
Sprayer Machine Operator (fertilizer/ pesticide/ insecticide)	13													
Seed Sower Machine Operator (Seeder Drills: till, plant and fertilizer simultaneously)	14													
Bed planter	15													
Reaper	16													
Thresher	17													
Irrigation equipment repair	18													
Tractor/power tiller repair	19													
Other non-farm agri equipment repair	20													

Household Number: | | | | |

		How has any of your hh member worked mainly in the mentioned activities in last 12 months<br>Self-employed................. 1<br>Rent out equipment......... 2<br>As labor or salaried employee .... 3<br>Did not work.................. 4<br>>> next activity	1st Respondent				2nd Respondent				3rd Respondent			
			Which member of the household worked in the sectors listed below?	How many of the past 12 months did you work on this activity?	How many days of the month did you work on this activity?	Total income in last 12 months	Which member of the household worked in the sectors listed below?	How many of the past 12 months did you work on this activity?	How many days of the month did you work on this activity?	Total income in last 12 months	Which member of the household worked in the sectors listed below?	How many of the past 12 months did you work on this activity?	How many days of the month did you work on this activity?	Total income
Other non-farm agri activity	21													
Seed dealer	22													
Fertilizer dealer	23													
Agricultural machinery dealer	24													

Module C3: Vocational Training / Technical Education (Male)

To be asked about both males and females who are 10 years old and above

MID	Name	Are you receiving/ have received any kind of vocational training/technical education?<br>Yes...1<br>No....2 >> Next person	What are you receiving/ have received training on?	From where are you receiving/ have received vocational training/technical education?<br><br>[If response code is 4, then go to C3_09]	If institutional training, how long is the training period?	If institutional training, did you receive any certificate/diploma/ degree in vocational training/technical education?<br>Yes…1<br>No…2 > C3_09<br>Training is not yet complete….3 >> C3_09	Is it a certificate course or diploma or Degree course?<br><br>Certificate…1<br>Diploma…2<br>Degree…3	In comparison with the general education, which grade is your qualification equivalent to?	How much did you spend overall (tuition and other fees) to obtain your certificate/diploma/degree?	What is the main reason why you decided to pursue vocational training/technical education?	Is your current job related to the training that you have acquired/are acquiring?<br><br>Yes...1<br>No....2 >> C3_12	If yes, do you use the skill that you acquired from training in your in your current job?<br><br>Yes…1<br>No…2	Has your vocational training / technical education helped you to find a suitable job/start your own business?<br><br>Yes…1<br>No….2
		Code↑	[Code 1]	[Code 2]	Months	Code↑	Code↑	[Code 3]	[TK]	[Code 4]	Code↑	Code↑	Code↑
MID	Name	C3_01	C3_02	C3_03	C3_04	C3_05	C3_06	C3_07	C3_08	C3_09	C3_10	C3_11	C3_12

Household Number: | | | |

Code 1	Code 2	Code 3	Code 4
Machinery repair/servicing…..1<br>Agricultural machinery repair/servicing… ....2<br>Electrical/Electronic Repair .... ….3<br>Automobile servicing/repair…...4<br>Textile/Garment/Knitting machine operator.......................... ….5<br>Dress-making/Tailoring/Embroidery/Stitching/Blocking.......... ….6<br>Computer Operator/IT Training… ....7<br>Beautification…..8<br>Leather Craft…...9<br>Dying/printing and finishing… ....10<br>Glass/Ceramic work… ....11<br>Carpentry…..12<br>Agriculture…..13<br>Food processing and preservation… ....14<br>Food packaging…...15<br>Fisheries/Fish Culture and Breeding… ....16<br>Livestock/Poultry rearing or farming… ....17<br>Nursing/Health Profession… ....18<br>Welding... …..19<br>Construction work / Masonry.. …..20<br>Driving…..21<br>Lathe machine operator …..22<br>Other….. 23	Government institute ....1<br>NGO...2<br>Other private institution ....3<br>Informal training 4	SSC/*Dakhil……10*<br>HSC/*Alim………12*<br>BA/BSC pass/*Fajil….14.*<br>BA/BCS honors/*Fajil..15*<br>MA/MSC and above/*Kamil……..16*<br>Medical/MBBS………71<br>Nursing…………….72<br>Engineering………73<br>Diploma engineer…74<br>Others (please mention)...75<br>Not equivalent to any class……96<br>Do not know……….98	To increase chances of finding a job....................................1<br>To start own business..2<br>To re-enter the workforce after losing previous job ....3<br>To increase income. ....4<br>To increase efficiency of the current work...........................5<br>Other...96

Module End Time: | Hour | | | Minute | | |

Household Number: [ ][ ][ ][ ]

Module D: Own Household Assets (Male)
Module D1: Current Household Assets (Male)

Module start time: Hour [ ][ ] Minu [ ][ ] Respondent ID: [ ] Consent : [ ] Yes ...1 No.....2

Description of asset	Asset code	Does your household own the item? Yes...1 No....2>>D1_12	Quantity	What portion of asset owned by household?	ID of owner Report three primary owners If household member, write MID If not household member, then use following codes: All members jointly .... 71 Male outside household .... 72 Female outside household. 73			How was the asset acquired? (report latest item if quantity >1) Purchase .... 1 Gift .... 2 Dowry .... 3 Inheritance .... 4 Own produced 5	Year of purchase / Acquisi-tion (report for latest item)	Purchase value/ price (report total value for all items owned)	Current value/ if asset sold today how much will you receive? (report total value for all items owned)	Type of asset* Consumption only .... 1 Consumption and Productive .... 2 Productive only 3	N/A for GFSS BASELINE: Quantity of asset during midline (2015) If 0>>next row	How many of the assets listed in midline (2015) were lost after midline survey? (Stolen, burnt, lost, damaged, dead etc.)	How many of the assets listed in midline (2015) were sold after midline (2015) survey? If 0 go to next row	If sold, then what was the price?	Reasons for Selling To meet household's food needs.................. 1 To meet needs other than food.................... 2 To meet children's education needs........... 3 For treatment .... 4 To purchase assets ...... 5 For emergency .... 6 Others (specify) 7		
			No.	%	MID			Code↑	Year	(Tk)	(Tk)	Code ↑	No.	No.	No.	(Tk)	Code↑		
**D1_01**	**D1_02**	**D1_03**	**D1_04**	**D1_05**	**D1_06a**	**D1_06b**	**D1_06c**	**D1_07**	**D1_08**	**D1_09**	**D1_10**	**D1_11**	**D1_12**	**D1_13**	**D1_14**	**D1_15**	**D1_16_a**	**D1_16_b**	**D1_16_c**
Trunk /Suitcase	1																		
Buckets / Pots	2																		
Stove / Gas burner	3																		
Metal cooking pots	4																		
Bed / Khat / Chowki	5																		
Armoire/Cabinet/ Alna	6																		
Table / chair	7																		
Hukka	8																		
Electric fan	9																		
Electric iron	10																		
Radio	11																		

Household Number: | | | | |

Description of asset	Asset code	Does your household own the item?<br>Yes...1<br>No....2>>D1_12	Quantity	What portion of asset owned by household?	ID of owner<br>Report three primary owners<br>If household member, write MID<br>If not household member, then use following codes:<br><br>All members jointly .... 71<br>Male outside household .... 72<br>Female outside household. 73			How was the asset acquired? (report latest item if quantity >1)<br><br>Purchase .... 1<br>Gift .... 2<br>Dowry .... 3<br>Inheritance .... 4<br>Own produced 5	Year of purchase / Acquisi-tion (report for latest item)	Purchase value/ price (report total value for all items owned)	Current value/ if asset sold today how much will you receive? (report total value for all items owned)	Type of asset*<br>Consumption only .... 1<br>Consumption and Productive .... 2<br>Productive only 3	N/A for GFSS BASELINE						
													Quantity of asset during midline (2015) If 0>>next row	How many of the assets listed in midline (2015) were lost after midline survey? (Stolen, burnt, lost, damaged, dead etc.)	How many of the assets listed in midline (2015) were sold after midline (2015) survey? If 0 go to next row	If sold, then what was the price?	Reasons for Selling<br><br>To meet household's food needs.................. 1<br>To meet needs other than food..................... 2<br>To meet children's education needs........... 3<br>For treatment .... 4<br>To purchase assets ...... 5<br>For emergency .... 6<br>Others (specify) 7		
			No.	%	MID			Code↑	Year	(Tk)	(Tk)	Code ↑	No.	No.	No.	(Tk)	Code↑		
**D1_01**	**D1_02**	**D1_03**	**D1_04**	**D1_05**	**D1_06a**	**D1_06b**	**D1_06c**	**D1_07**	**D1_08**	**D1_09**	**D1_10**	**D1_11**	**D1_12**	**D1_13**	**D1_14**	**D1_15**	**D1_16_a**	**D1_16_b**	**D1_16_c**
Audio cassette/CD player	12																		
Wall clock /watch	13																		
Wristwatch	131																		
Television (B/W)	14																		
Television (Color)	15																		
Camera/ Video Camera	50																		
Jewelry (gold)	16																		
Jewelry (silver)	161																		
Sewing machine	17																		
Bicycle	18																		
Rickshaw	19																		
Van (tricycle van)	20																		
Boat	21																		

Household Number: ☐☐☐☐

Description of asset	Asset code	Does your household own the item?<br>Yes...1<br>No....2>>D1_12	Quantity	What portion of asset owned by household?	ID of owner<br>Report three primary owners<br>If household member, write MID<br>If not household member, then use following codes:<br><br>All members jointly ....71<br>Male outside household ....72<br>Female outside household. 73			How was the asset acquired? (report latest item if quantity >1)<br><br>Purchase ....1<br>Gift ....2<br>Dowry ....3<br>Inheritance ....4<br>Own produced 5	Year of purchase / Acquisi-tion (report for latest item)	Purchase value/ price (report total value for all items owned)	Current value/ if asset sold today how much will you receive? (report total value for all items owned)	Type of asset*<br>Consumption only ....1<br>Consumption and Productive ....2<br>Productive only 3	N/A for GFSS BASELINE<br>Quantity of asset during midline (2015)<br>If 0>>next row	How many of the assets listed in midline (2015) were lost after midline survey? (Stolen, burnt, lost, damaged, dead etc.)	How many of the assets listed in midline (2015) were sold after midline (2015) survey?<br>If 0 go to next row	If sold, then what was the price?	Reasons for Selling<br><br>To meet household's food needs.................. 1<br>To meet needs other than food..................... 2<br>To meet children's education needs........... 3<br>For treatment .... 4<br>To purchase assets ...... 5<br>For emergency .... 6<br>Others (specify) 7		
			No.	%	MID			Code↑	Year	(Tk)	(Tk)	Code ↑	No.	No.	No.	(Tk)	Code↑		
**D1_01**	**D1_02**	**D1_03**	**D1_04**	**D1_05**	**D1_06a**	**D1_06b**	**D1_06c**	**D1_07**	**D1_08**	**D1_09**	**D1_10**	**D1_11**	**D1_12**	**D1_13**	**D1_14**	**D1_15**	**D1_16_a**	**D1_16_b**	**D1_16_c**
Engine boat	22																		
Motorcycle	23																		
Mobile phone set	24																		
Land phone set	25																		
Dheki	26																		
Jata	27																		
Randa	28																		
Saw	29																		
Hammer	30																		
Patkoa	31																		
Fishing net	32																		
Spade (Kodal)	33																		
Axe (Kural)	34																		
Shovel (belcha)	35																		

Description of asset	Asset code	Does your household own the item? Yes...1 No....2>>D1_12	Quantity	What portion of asset owned by household?	ID of owner Report three primary owners If household member, write MID If not household member, then use following codes: All members jointly ....7 1 Male outside household ....7 2 Female outside household. 7 3			How was the asset acquired? (report latest item if quantity >1) Purchase .... 1 Gift .... 2 Dowry .... 3 Inheritance .... 4 Own produced 5	Year of purchase / Acquisi-tion (report for latest item)	Purchase value/ price (report total value for all items owned)	Current value/ if asset sold today how much will you receive? (report total value for all items owned)	Type of asset* Consumption only .... 1 Consumption and Productive .... 2 Productive only 3	N/A for GFSS BASELINE: Quantity of asset during midline (2015) If 0>>next row	How many of the assets listed in midline (2015) were lost after midline survey? (Stolen, burnt, lost, damaged, dead etc.)	How many of the assets listed in midline (2015) were sold after midline (2015) survey? If 0 go to next row	If sold, then what was the price?	Reasons for Selling To meet household's food needs.................. 1 To meet needs other than food..................... 2 To meet children's education needs........... 3 For treatment .... 4 To purchase assets...... 5 For emergency............ 6 Others (specify) 7		
			No.	%	MID			Code↑	Year	(Tk)	(Tk)	Code ↑	No.	No.	No.	(Tk)	Code↑		
**D1_01**	**D1_02**	**D1_03**	**D1_04**	**D1_05**	**D1_06a**	**D1_06b**	**D1_06c**	**D1_07**	**D1_08**	**D1_09**	**D1_10**	**D1_11**	**D1_12**	**D1_13**	**D1_14**	**D1_15**	**D1_16_a**	**D1_16_b**	**D1_16_c**
Shabol	36																		
Daa	37																		
Horse	38																		
Mule	39																		
Donkey	40																		
Cow	401																		
Goat/ Sheep	402																		
Duck/ Hen	403																		
Other Animal (specify)	41																		
Cash in hand	42																		
Solar energy panel	43																		
Electricity Generator	44																		
IPS	45																		
Computer/ Laptop	46																		

Household Number: | | | | |

Description of asset	Asset code	Does your household own the item?<br>Yes...1<br>No....2>>D1_12	Quantity	What portion of asset owned by household?	ID of owner<br>Report three primary owners<br>If household member, write MID<br>If not household member, then use following codes:<br><br>All members jointly .... 7 1<br>Male outside household .... 7 2<br>Female outside household. 7 3			How was the asset acquired? (report latest item if quantity >1)<br><br>Purchase .... 1<br>Gift .... 2<br>Dowry .... 3<br>Inheritance .... 4<br>Own produced 5	Year of purchase / Acquisi-tion (report for latest item)	Purchase value/ price (report total value for all items owned)	Current value/ if asset sold today how much will you receive? (report total value for all items owned)	Type of asset*<br>Consumption only .... 1<br>Consumption and Productive .... 2<br>Productive only 3	N/A for GFSS BASELINE						
													Quantity of asset during midline (2015)<br>If 0>>next row	How many of the assets listed in midline (2015) were lost after midline survey? (Stolen, burnt, lost, damaged, dead etc.)	How many of the assets listed in midline (2015) were sold after midline (2015) survey?<br>If 0 go to next row	If sold, then what was the price?	Reasons for Selling<br><br>To meet household's food needs.................. 1<br>To meet needs other than food..................... 2<br>To meet children's education needs........... 3<br>For treatment .... 4<br>To purchase assets ...... 5<br>For emergency .... 6<br>Others (specify) 7		
			No.	%	MID			Code↑	Year	(Tk)	(Tk)	Code ↑	No.	No.	No.	(Tk)	Code↑		
**D1_01**	**D1_02**	**D1_03**	**D1_04**	**D1_05**	**D1_06a**	**D1_06b**	**D1_06c**	**D1_07**	**D1_08**	**D1_09**	**D1_10**	**D1_11**	**D1_12**	**D1_13**	**D1_14**	**D1_15**	**D1_16_a**	**D1_16_b**	**D1_16_c**
Flash Drive/ Memory Card	47																		
Printer	48																		
Tab	49																		
Other1 [______________]	511																		
Other2 [______________]	512																		

Note:* Consumption assets are used by household members that do not generate income. Productive assets are used for generating income.

For example, a milk cow is a consumption asset if its milk is used for only consumption for the household and not sold; but if the milk is sold then the milk cow is considered as a productive asset because it generates income for the household. If the milk is consumed by the household and also sold for income then the milk cow is considered as both consumption and productive asset.

Module D2: Agricultural Implements and Other Productive assets (Male)

												N/A for GFSS BASELINE						
Description of asset	Asset code	Does your household own the item?<br>Yes...1<br>No...2>> D2_12	Quantity	What portion of asset owned by household?	ID of owner<br>Report three primary owners<br>If household member, write MID<br>If not household member, then use following codes:<br><br>All members jointly........... 71<br>Male outside household..... 72<br>Female outside household.. 73			How was the asset acquired?<br>(report most recent item if quantity >1)<br><br>Purchase............1<br>Gift ....2<br>Dowry...............3<br>Inheritance ....4<br>Own produced...5	Year of purchase/acquisition (report for the most expensive item if quantity >1)	Purchase value/ price (report total value for all items owned)	Current value/ if asset sold today how much will you receive? (report total value for all items [illegible]	In the last round (2015), what was the quantity of assets in this household? (Number)<br>If '0' then move to the next row	What quantity of assets recorded in the last round (2015) have you lost after the last survey? (Stolen, burnt, lost, damaged, dead etc.)(Number)	What quantity of assets recorded in the last round (2015) have you sold after the last survey? (Number)<br>If '0' then move to the next row	What was the value of sales? (Tk)	Reason for selling?<br><br>To meet household's food needs ....1<br>To meet needs other than food ....2<br>To meet children's education needs ..3<br>For treatment .....4<br>To purchase assets ....5<br>For emergency....6<br>Others (specify)..7		
	Code		No.		MID			Code ↑	Year	(Tk)	(Tk)	(No)	(No)	(No)	(Tk)	Code ↑		
**D2_01**	**D2_02**	**D2_ 03**	**D2_04**	**D2_05**	**D2_06a**	**D2_06b**	**D2_06c**	**D2_07**	**D2_08**	**D2_09**	**D2_10**	**D2_12**	**D2_13**	**D2_14**	**D2_ 15**	**D2_16a**	**D2_16b**	**D2_16c**
Farming tools:																		
Manual Reaper/Sickle	1																	
Weeding tool	2																	
Harrow	3																	
Rake	4																	
Plough/ yoke	5																	
Winnowing Machine	6																	
Pesticide sprayer	7																	
Wheelbarrow	8																	
Bullock cart	9																	
Push cart	10																	
Other Light Machinery (Specify)	11																	
Machinery:																		
Tractor	12																	
Power Tiller	13																	
Trolley/Trailers	14																	
Thresher	15																	
Fodder cutting machine	16																	

Household Number:

												N/A for GFSS BASELINE						
Description of asset	Asset code	Does your household own the item? Yes...1 No...2>> D2_12	Quantity	What portion of asset owned by household?	ID of owner<br>Report three primary owners<br>If household member, write MID<br>If not household member, then use following codes:<br><br>All members jointly........... 71<br>Male outside household..... 72<br>Female outside household.. 73			How was the asset acquired?<br>(report most recent item if quantity >1)<br><br>Purchase............1<br>Gift ....2<br>Dowry...............3<br>Inheritance ....4<br>Own produced...5	Year of purchase/acquisition (report for the most expensive item if quantity >1)	Purchase value/ price (report total value for all items owned)	Current value/ if asset sold today how much will you receive? (report total value for all items	In the last round (2015), what was the quantity of assets in this household? (Number) If '0' then move to the next row	What quantity of assets recorded in the last round (2015) have you lost after the last survey? (Stolen, burnt, lost, damaged, dead etc.)(Number)	What quantity of assets recorded in the last round (2015) have you sold after the last survey? (Number) If '0' then move to the next row	What was the value of sales? (Tk)	Reason for selling?<br><br>To meet household's food needs ....1<br>To meet needs other than food ....2<br>To meet children's education needs ..3<br>For treatment .....4<br>To purchase assets ....5<br>For emergency....6<br>Others (specify) ..7		
	Code		No.		MID			Code ↑	Year	(Tk)	(Tk)	(No)	(No)	(No)	(Tk)	Code ↑		
**D2_01**	**D2_ 02**	**D2_ 03**	**D2_ 04**	**D2_ 05**	**D2_06a**	**D2_06b**	**D2_06c**	**D2_07**	**D2_08**	**D2_09**	**D2_10**	**D2_12**	**D2_13**	**D2_14**	**D2_ 15**	**D2_ 16a**	**D2_ 16b**	**D2_ 16c**
Swing basket	17																	
Don	18																	
Hand tube well	19																	
Treadle pump	20																	
Rower pump	21																	
Jumbo Pump (Axial Flow pump)	36																	
Low lift pump (LLP) for irrigation	22																	
Shallow tube well (STW)	23																	
Deep tube well (DTW)	24																	
Electric motor pump	25																	
Diesel motor pump	26																	
Spraying machines (chem./ fertilizer)	27																	
Reaper	28																	

Household Number: [ ] [ ] [ ] [ ]

												N/A for GFSS BASELINE						
Description of asset	Asset code	Does your household own the item?<br>Yes...1<br>No....2>> D2_12	Quantity	What portion of asset owned by household?	ID of owner<br>Report three primary owners<br>If household member, write MID<br>If not household member, then use following codes:<br><br>All members jointly........... 71<br>Male outside household..... 72<br>Female outside household.. 73			How was the asset acquired?<br>(report most recent item if quantity >1)<br><br>Purchase...........1<br>Gift ....2<br>Dowry...............3<br>Inheritance ....4<br>Own produced...5	Year of purchase/acquisition (report for the most expensive item if quantity >1)	Purchase value/ price (report total value for all items owned)	Current value/ if asset sold today how much will you receive? (report total value for all items	In the last round (2015), what was the quantity of assets in this household? (Number)<br>If '0' then move to the next row	What quantity of assets recorded in the last round (2015) have you lost after the last survey? (Stolen, burnt, lost, damaged, dead etc.)(Number)	What quantity of assets recorded in the last round (2015) have you sold after the last survey? (Number)<br>If '0' then move to the next row	What was the value of sales? (Tk)	Reason for selling?<br><br>To meet household's food needs ....1<br>To meet needs other than food ....2<br>To meet children's education needs ..3<br>For treatment .....4<br>To purchase assets ....5<br>For emergency....6<br>Others (specify) ..7		
	Code		No.		MID			Code ↑	Year	(Tk)	(Tk)	(No)	(No)	(No)	(Tk)	Code ↑		
**D2_01**	**D2_02**	**D2_ 03**	**D2_04**	**D2_05**	**D2_06a**	**D2_06b**	**D2_06c**	**D2_07**	**D2_08**	**D2_09**	**D2_10**	**D2_12**	**D2_13**	**D2_14**	**D2_ 15**	**D2_16a**	**D2_16b**	**D2_16c**
Seeder Drills: till, plant, fertilize simultaneously	37																	
Bed planters (forms fields into beds and furrows)	38																	
Other Heavy Machinery (Specify)	29																	
Other productive																		
Masons	30																	
Potters Chaka	31																	
Blacksmiths Hapor	32																	
Charka	33																	
Briquette Urea Applicator (Injector)	34																	
Briquette Urea Applicator (Push)	35																	
Combined harvester	39																	

Household Number: | | | |

												N/A for GFSS BASELINE						
Description of asset	Asset code	Does your household own the item?<br>Yes...1<br>No....2>> D2_12	Quantity	What portion of asset owned by household?	ID of owner<br>Report three primary owners<br>If household member, write MID<br>If not household member, then use following codes:<br>All members jointly........... 71<br>Male outside household..... 72<br>Female outside household.. 73			How was the asset acquired?<br>(report most recent item if quantity >1)<br>Purchase...........1<br>Gift ....2<br>Dowry...............3<br>Inheritance ....4<br>Own produced...5	Year of purchase/acquisition (report for the most expensive item if quantity >1)	Purchase value/ price (report total value for all items owned)	Current value/ if asset sold today how much will you receive? (report total value for all items [illegible]	In the last round (2015), what was the quantity of assets in this household? (Number) If '0' then move to the next row	What quantity of assets recorded in the last round (2015) have you lost after the last survey? (Stolen, burnt, lost, damaged, dead etc.)(Number)	What quantity of assets recorded in the last round (2015) have you sold after the last survey? (Number) If '0' then move to the next row	What was the value of sales? (Tk)	Reason for selling?<br>To meet household's food needs ....1<br>To meet needs other than food ....2<br>To meet children's education needs ..3<br>For treatment .....4<br>To purchase assets ....5<br>For emergency....6<br>Others (specify) ..7		
	Code		No.		MID			Code ↑	Year	(Tk)	(Tk)	(No)	(No)	(No)	(Tk)	Code ↑		
**D2_01**	**D2_02**	**D2_ 03**	**D2_04**	**D2_05**	**D2_06a**	**D2_06b**	**D2_06c**	**D2_07**	**D2_08**	**D2_09**	**D2_10**	**D2_12**	**D2_13**	**D2_14**	**D2_ 15**	**D2_16a**	**D2_16b**	**D2_16c**
Rice trans planter	40																	
Closed drum thresher	41																	
Open drum thresher	42																	
Corn Sheller	43																	
Sugarcane crusher	44																	
There No agriculture asset in this	99																	

Module End Time: | Hour | | | Minute | | |

Household Number: | | | | |

Module E: Savings (Male)

Module start time: Hour | | | Minu | | | Respondent ID: | | Consent : | | Yes ...1 No.....2

E01. Have any adult in the household had any savings in the past 1 year? | | Yes .... 1 No.......... 2

E02. Does any adult in the household currently have any savings? | | Yes......... 1 No.......... 2 **If NO, END MODULE**

Ask how many accounts each individual currently has and list them all. Each "account" should have a separate row. If the individual has more than one "account", put in separate rows.

Serial No.	Saver	Where do you save?	If the source of savings is the NGO, then record the code for NGO	How do you use / plan to use the savings? **Report primary use of savings**	Total amount currently saved in this savings account?	How frequently do you save?
		[Code 1]	[Code 4]	[Code 2]	(Tk)	[Code 3]
**E03**	**MID**	**E04**	**E08**	**E05**	**E06**	**E07**

Code 1: Where	Code 4:NGO Code			Code 2: Use / Intended Use	Code 3: How frequently do you save
At home .... 1<br>NGO (name of NGO) .... 2<br>Shamity (other than NGO) .... 3<br>Bank .... 4<br>Shop .... 5<br>Post office / government institution .... 6<br>Employer's provident fund..... 7<br>Insurance company .... 8<br>Relative / friend / neighbor .... 9<br>Savings collector .... 10<br>Land leased in from other household.......... 11<br>Other (specify) 12	BRAC.................................1<br>ASA...................................2<br>PROSHIKA........................3<br>Karitas Bangladesh..............4<br>Shwanirbhar Bangladesh.....5<br>TMSS.................................6<br>RDRS Bangladesh...............7<br>Bureau Tangail...................8<br>Jagoroni Chakra.................9<br>Voluntary Organization for Social Development (VOSD).............................10<br>Peoples Oriented Program Implementation (POPI)......11<br>Gono Kalyan Trust (GKT).12<br>Bachte Shekha...................13<br>PKSF.................................14<br>Bangladesh Rural Development Board(BRDB....................15	PodokhepManobik Unnyan Kendra….. 17<br>Heed Bangladesh .... ……...18<br>Bureau Bangladesh .... ……...19<br>Community Development Center (CODEC)................................. ………20<br>Gono Milon Foundation............ ………21<br>Shapla Ful .... ………22<br>Sheba Manob kolyan Kendra (SMKK) 23<br>Society for Disadvantaged Origin (SDO) .... ……...24<br>Akota Shomaj Unnyan Kendra (ASUK) 25<br>Bangladesh Development Society …...26<br>Social Organization for Voluntary Advancement (SOVA).................................. ………27<br>Society development Committee (SDC) 28<br>Faridpur Development Agency (FDA)..29	Ashar Alo Unnyan Shangstha ....30<br>Polli Progoti Sohayok Samity ....31<br>Samadhan......................................32<br>Manob Seba Sangstha ....33<br>Nobolok Parishad..........................34<br>Rural reconstruction Foundation (RRF) 35<br>Christian Civil Society (CSS) ....36<br>Uddipon ....37<br>Daak diye jai ....38<br>Shushilon ....39<br>Uttaran ....40<br>Unnyan Procheshta ....41<br>Setu Bangladesh............................42<br>Satkhira Unnyan Shangstha (SUK) 43<br>Ideal ....44<br>Manob Sompod Unnyan kendra.....45<br>Grameen bank ....46<br>HKI (Hellen Keller International).47<br>Other NGOs (specify) 48	To buy household goods ...... 1<br>To buy agricultural implements 2<br>To buy productive assets (other than agriculture) .... 3<br>To start / help business.........4<br>To buy land / house ....5<br>For education / training ....6<br>For marriage / dowry...........7<br>To build / repair house ....8<br>To get loan ....9<br>To lend to others ....10<br>To prepare for difficult times/danger .... 11<br>To send someone abroad for a job...12<br>For the future of children ... 13<br>Medical or other emergency..........................14<br>Don't know/no special reason................................ 15<br>Other (specify) 16	Daily .... 1<br>Weekly .... 2<br>Monthly .... 3<br>Quarterly............................... 4<br>Biannually (every 6 months).. 5<br>Yearly .... 6<br>Not regularly......................... 7<br><br>END TIME<br>HOUR : MINUTE

Household Number: | | | |

Module F: Loans (Male)

Module start time: Hour | | | Minu | | | Respondent ID: | | Consent : | | Yes ...1 No.....2

Report cash loans. Include both interest-bearing and non-interest-bearing cash loans.

F01. Has any member in the household ever had any loans? ☐ Yes ....1 No 2

F02. Does any member in the household currently have a loan with any individual or institution? ☐ Yes .... 1 >> Fill out the table below No 2

F02_a Have you or any other member of the household applied for loans in the last 12 months ☐ Yes......... 1 >> F02_b No .... 2 >> F02_c

F02_b If you applied for loans, what was the reason for your application being denied? ☐

Did not have collateral………………………………1
Did not have enough savings to qualify for loan…2
Did not repay previous loans (loan defaulter) ……3
Don't know…………………………………...……4
Applied for and received loan (repaid) ……..……..…5
Other (specify)… …………………………………6

} **NEXT MODULE**

F02_c If you did not apply for loans, why did you not apply? ☐

Did not need loan, and so did not apply……………………………………1
Needed loan but did not apply fearing rejection of application …………2
Needed loan but did not apply fearing not receiving the loan on time……3
Needed loan but did not apply fearing high interest rates………………4
Could not apply for not having collateral……………………………………5
Others (specify) …………………………………………………………6

} **NEXT MODULE**

Household Number: | | | |

First ask how many loans each individual currently has and list them all. Each loan should have a separate row. If an individual has more than one loan, put in separate rows.

Serial No.	Who took the loan?	What was the source of the loan?	If the source is an NGO, write the code of that NGO	What was the loan mainly used for?<br><br>Report primary 3 uses			Amount of loan	What was the interest rate?<br><br>(Report 999 if respondent does not know the interest rate)	What is the outstanding amount of the loan?	Is the outstanding amount with or without interest?<br><br>With interest............1<br>Without interest.......2	What is the maximum that you can borrow from this source?	What amount of borrowings have you applied for from this source?
		[Code 1]	[Code 2]	[Code 3]			(Tk)	(percent)	(Tk)	Code ↑	(Tk)	(Tk)
**F03**	**MID**	**F04**	**F05**	**F06_a**	**F06_b**	**F06_c**	**F07**	**F08**	**F09**	**F10**	**F11**	**F12**

Code 1: Source of loan

- Relative/friend/neighbor..............1
- Bangladesh Krishi Bank (BKB) .2
- Rajshahi Krishi Bank (RAKUB) 3
- Other Bank..................................4
- Other financial institution............5
- NGO (name of NGO______) 6
- Employer.....................................7
- Shop / Dealer / Trader 8
- Money lender 9
- Shamity (other than NGO) 10
- Leased out land to other HH......11
- Other (specify) 12

Code 2 :NGO

- BRAC 1
- ASA 2
- PROSHIKA.............................3
- Karitas Bangladesh..................4
- Shwanirbhar Bangladesh 5
- TMSS......................................6
- RDRS Bangladesh...................7
- Bureau Tangail........................8
- Jagoroni Chakra 9
- Voluntary Organization for Social Development (VOSD)..10
- Peoples Oriented Program Implementation (POPI) 11
- Gono Kalyan Trust (GKT) 12
- Bachte Shekha.........................13
- PKSF.......................................14
- Bangladesh Rural Development Board(BRDB) 15
- PodokhepManobik Unnyan Kendra 17
- Heed Bangladesh 18
- Bureau Bangladesh 19
- Community Development Center (CODEC).................................. 20
- Gono Milon Foundation............. 21
- Shapla Ful 22
- Sheba Manob kolyan Kendra (SMKK) 23
- Society for Disadvantaged Origin (SDO) 24
- Akota Shomaj Unnyan Kendra (ASUK) 25
- Bangladesh Development Society 26
- Social Organization for Voluntary Advancement (SOVA)............... 27
- Society development Committee (SDC) 28
- Faridpur Development Agency (FDA)29
- Ashar Alo Unnyan Shangstha 30
- Polli Progoti Sohayok Samity 31
- Samadhan............................... 32
- Manob Seba Sangstha.............33
- Nobolok Parishad 34
- Rural reconstruction Foundation (RRF) 35
- Christian Civil Society (CSS).36
- Uddipon..................................37
- Daak diye jai...........................38
- Shushilon................................39
- Uttaran....................................40
- Unnyan Procheshta 41
- Save Bangladesh.....................42
- Satkhira Unnyan Shangstha (SUK) 43
- Ideal..44
- Manob Sompod Unnyan Kendra 45
- Grameen bank.........................46
- HKI (Helen Keller International) 47
- Other NGOs (specify).............48

Code 3: Loan use

- Business enterprise 1
- To buy fertilizer 2
- To buy seeds 3
- To buy pesticides 4
- To buy irrigation equipment 5
- To buy other agricultural implements 6
- To buy water for irrigation..........7
- Costs of diesel/electricity for agriculture..................................8
- Labor wages for agriculture 9
- Costs of hired machines/animals for agriculture 10
- To buy productive assets for purposes other than agriculture .11
- For lease of land for agriculture (cash only) 12
- For lease of land used for purpose other than agriculture (cash only) 13
- Purchase Land.......................... 14
- To purchase cow/goat 15
- For medical treatment 16
- To meet household consumption needs 17
- Rent/purchase/improve housing 18
- Educational expenses 19
- Marriage expenditure 20
- Dowry 21
- Funeral 22
- To lend out at higher interest.... 23
- To go abroad 24
- To repay other loan 25
- Other (specify) 26

Module End Time: Hour | | | Minute | | |

Household Number: | | | | |

Module XXc: Questions on Child Marriage Law (Male and Female)

Ask to both male and female parents or guardians

Now I would like to ask you some questions regarding your knowledge of child marriage law.

Module start time: Hour | | | Minu | | | Respondent ID: | | Consent : | | Yes ...1 No.....2

SL	Questions	Answer	Answer code
XXc_01	Is there a legal minimum age of marriage for girls?"<br><br>Skip rest of the module if the answer is "No" or "Don't Know		Yes......................1<br>No ....2 > next module<br>Don't know..........3 > next module
XXc _02	What is the legal minimum age of marriage for girls		Age (year)
XXc _03	If a girl is married before the legal minimum age of marriage, what type of actions can the government take?<br><br>MARK ALL THAT APPLY<br><br>(multiple responses possible)		Marriage is void..........1<br>The bride would be jailed ....2<br>The guardian or father would be jailed or fined..........3<br>The husband would be jailed or fined..........4<br>Nothing would happen..........5<br>Other (specify)..........96<br>Don't know..........98
XXc_04	What is the legal punishment for marrying or facilitating marriage of a girl below this age (fine amount in taka)?		Fine (taka)<br>Do not know……..9888
XXc_05	What is the legal punishment for marrying or facilitating marriage of a girl below this age (jail time in months)?		Jail (months)<br>Do not know……..9888
XXc_06	When did this law come into effect? [Year]		[Year]<br>Do not know……..9888
XXc _07	Are there any exceptions to this law?		Yes......................1<br>No ....2<br>Don't know..........3
XXc _08	If yes, what are the exceptions<br><br>MARK ALL THAT APPLY<br><br>(multiple responses possible)		Special Exemption Clause .... 1<br>If Parent wants......................................2<br>If Family wants.....................................3<br>Other (specify)......................................47

Household Number: | | | | |

SL	Questions	Answer	Answer code
XXc _09	When did you first hear about the current law about the legal minimum age?		Year<br>Do not know……..9888
XXc_10	Where (i.e. from which source) did you first hear/see/watch/read about the law about the current law about the legal minimum age?		Newspaper or magazine... 1<br>Radio.. 2<br>Television .... 3<br>Poster .... 4<br>Community Programme .... 5<br>Family .... 6<br>Relatives .... 7<br>Neighbors .... 8<br>Did not hear of it………...………………………………........9<br>Other (specify) 96
XXc_11	Do you know of any case of child marriage where the current law was implemented?		Yes....................... 1<br>No 2

Module End Time: | Hour | | | Minute | | |

Household Number: | | | | |

Module G: Roster of land and pond/water bodies owned or under operation (Male)

Module start time: Hour | | | Minu | | | **Respondent ID:** | | Consent : | | Yes ...1 No.....2

List all land (all type of land & water bodies) owned or under operation in last 12 months [1st December (15th Agrahayon) 2017to 30th November (14th Agrahayon) 2018].

Plot ID	Plot Description	Plot Type	What was the area of land in the last round (midline) or in 2015 for GFSS?	Current Size/ Area	Status of the plot in the current round<br>If answer to G02 is 0 and answer to G20 is 1 to 4 then go to the next row	Distance from home<br>if next to home "0"	Usual flood depth (during monsoon/ flood season)<br>if not flooded report "0"	Soil type	Current operational status (last 12 months)	Who is the owner of the sharecropeed land?<br>Husband's relative….1<br>Wife's relative….2<br>Non-relative….3<br>If the ans to G06 is 3 to 5 or 9 or 11 to 13, then fill out these 3 columns	Where do the owner of the sharecropped land live?<br>Inside village….1<br>Different village….2<br>Town….3<br>Abroad…..4	Socio-economic status of the owner in comparison to the farmer<br>Richer…1<br>Same……2<br>Poorer…3	If the plot is rented/ leased in/out for cash, report amount received per month.<br>Note: Report only if response in G06 is "3" OR "6"	Who owns the plot? (member ID) Report 3 primary owners.<br>If HH member, write MID.<br>If outside household, use code 4.			Who owns the plot officially?<br>Report 3 primary owners MID			Current market value of the land (amount expected to spend/ pay if you want to buy)	How was this land acquired	Year of acquisition of the land
Plot ID		Code 1	Decimal	Decimal	Code 7	Meter	Feet	Code 2	Code 3	Code ↑	Code ↑	Code ↑	Tk	MID/Code 4			MID/Code 4			Tk	Code 5	Year
**Plot ID**	**Description**	**G01**	**G02_1**	**G02**	**G20**	**G03**	**G04**	**G05**	**G06**	**G21**	**G22**	**G23**	**G07**	**G08**			**G09**			**G10**	**G11**	**G12**
														**A**	**B**	**C**	**A**	**B**	**C**			
1	Homestead	1																				

Module G: Roster of land and pond/water bodies owned or under operation (continued) (Male)

List all land (all type of land & water bodies) owned or under operation during 1st December (15th Agrahayon) 2017- 30th November (14th Agrahayon) 2018.

Plot ID	Plot Description	In the last 12 months who decided to build any infrastructure(if any) on the plot?<br><br>Report 3 primary decision takers.<br>If HH member, write MID.<br>If outside household, use code 4.			How was the plot utilized in the last season?<br>If response is "3"-"8" then Go to next plot	Who worked on the plot last season?			Generally, who takes decision regarding type of crop to be planted/fish culture?			Generally, who takes decision regarding inputs? (seeds, fertilizer, irrigation etc.)			If any produce was sold from crops planted/fish cultured [PLOT] in the last growing season, who was responsible for taking the crop to market and negotiating the sale?			If any revenue was generated from on the [PLOT] in the last growing season, who decided how to spend the revenues?		
Plot ID		MID/Code4			Code 6	MID/Code4			MID/Code4			MID/Code4			MID/Code4			MID/Code4		
**Plot ID**	**Plot Description**	**G13**			**G14**	**G15**			**G16**			**G17**			**G18**			**G19**		
		**A**	**B**	**C**		**A**	**B**	**C**	**A**	**B**	**C**	**A**	**B**	**C**	**A**	**B**	**C**	**A**	**B**	**C**
1	Homestead																			

Code 1:Plot type	Code 2: Soil type	Code 3: Operation status	Code 4: Type of ownership	Code 5: How acquired	Code 6: How was the plot utilized	Code 7
Homestead ....1<br>Cultivable/arable land ....2<br>Pasture...........................3<br>Bush/forest ....4<br>Waste/non-arable land....5<br>Land in riverbed ....6<br>Other residential/commercial plot ....7<br>Cultivable Pond.............8<br>Derelict pond.................9<br>Garden (wood/Friut).......10<br>Floating plot).................11<br>Only for seed bed)..........12	Clay.............1<br>Loam ....2<br>Sandy............3<br>Clay-loam.....4<br>Sandy-loam ..5	Fallow....................................1<br>Own operated ....2<br>Rented/leased in/ for cash.......3<br>Rented/leased in/crop share ....4<br>Mortgaged in ....5<br>Rented/leased out/cash...........6<br>Rented/leased out/crop share ..7<br>Mortgage out ....8<br>Group leased in with other farmer ....9<br>Leased out to NGO.................10<br>Taken from joint owner ....11<br>Jointly with other owners........12<br>Rented in for certain amount ofcrops...................................13<br>Rented out for certain amount ofcrops...................................14<br>Free of cost…………………..15	All members jointly<br>..7<br>1<br>Male outside household<br>..7<br>2<br>Female outside household<br>..7<br>3<br>Govt / Khas land/other institutions<br>..7<br>4<br>Not applicable/Not decided<br>..9<br>8	Purchased/bought ....1<br>Inherited (wife's family) ....2<br>Inherited (husband's family) ...3<br>User right (wife's family)........4<br>User right (husband's family)..5<br>Rented/shared/leased/ Mortgaged–in ....6<br>Government Khas land/Other institution...............................7	Agriculture ....1<br>Fisheries ....2<br>Grazing for livestock ....3<br>Homestead/ house plot ....4<br>Bush ....5<br>Commercial/non-ag enterprise6<br>Fallow....................................7<br>Other (specify)........................8<br>Was not with me.....................9	Plot is the same in both rounds .... 0<br>Sold..................................... 1<br>Inherited or Household Split 2<br>Mortgage out/ Rent .... 3<br>erosion .... 4<br>New Purchase .... 5<br>New inheritance .... 6<br>New Mortgage .... 7<br>Household combined .... 8<br>Char surfaced .... 9<br>Others (specify) 10

**Module End Time:**	Hour			Minute		

Household Number: | | | | |

Module H: Agriculture (Male)

Module start time: | Hour | | | Minu | | | **Respondent ID:** | | Consent : | | Yes ...1 No.....2

Note:
- Do not include leased/rented out plots.
- If more than one crop is harvested on the same plot during the recall period, then use separate crop row for each crop.
- Collect plot level data in case of inter-cropping. For more than one crop report information using crop code.
- Crops that are not completely harvested, collect harvested quantity and collect expected harvest (production) of that left in field.
- If plot is divided (at the same time) for different crop production (e.g. intercropping &/mixed cropping), then use decimal for divided plot/sub plot.[e.g. if plot no. 5 is divided into 3 sub plots then write 5.1, 5.2 and 5.3 as plot ID.].
- Write area in decimal of sub plot in H1_03, please note that summation of all sub plots will be less than or equal to the total area of original plot mentioned in Module G.

Module H1: Agriculture Plot Utilization (Male)

Report for plot wise crop data for all Crop cultivated during 1st December (15th Agrahayon) 2017- 30th November (14th Agrahayon) 2018.
Note: BRING ALL INFORMATION ON HOMESTEAD FARMING (ALL CROPS, AS WELL AS FISH, IF FARMED ALONGSIDE RICE).
In case of intercropping, report the plantation week/month of the main crop. If the respondent responds in Bangla months for plantation/broadcast, please convert those weeks and months according to English calendar and write in designated column. **PLOT ID in Module G and Module H must match**.

Sl. no.	Plot ID	Crop season Aman (kharif2)…..1 Aus (kharif1)…..2 Boro (rabi)……3 Annual …..4	Crop Name	Crop Code In case of Intercropping report Crop code for the second crop in shaded column below		If Paddy Report Variety For all other crop report "--" in both column Variety Name and column H1_01		Crop type Local Variety .... 1 HYV .... 2 Hybrid 3	Area planted	Date planted		Source of seed (report primary source for each crop) If seedlings are created, record 11 in H1_05a and record the source in the next column		Is line sowing maintained: Broadcast…1 Line sowing ….2 Yes, straight line…3>H1_08 Yes, slightly slanted…4>H1_08 No, zigzag…5>H1_08	Cost of Seed If the code for source of seeds is 1 or 2 then record the approximate value of seeds. Note: Do not include cost of seeds used to produce seedling>>H1_09	Seedling Cost If own seedling was used, record the approximate value of seedling Note: Do not include cost of seeds used to produce seedling	If you ploanted seedling, then how old (in days) is the seedling?	When did you last purchase seed/ seedling? This year 0 One year ago 1 Two years ago…..2 In this way 3, 4 and so forth Never purchased 99	In your view, what are the two important features of the seeds		Were there any problems with the purchased seed/ seedling? If answer is not 7 then go to next plot		Reason for inadequacy	
		Code ↑		Code 1		Variety Name	Code 3	Code ↑	Decimal	Week	Month	Code 4		Code ↑	Tk	Tk	Days	Code ↑	Code 5		Code 6		Code 7	
**Sl.**	**Plot ID**	**season**	**Crop Name**	**Crop code**		**Variety Name**	**H1_ 01**	**H1_02**	**H1_03**	**H1_04a**	**H1_04b**	**H1_05a**	**H1_05b**	**H1_05c**	**H1_07**	**H1_08**	**H1_08a**	**H1_09**	**H1_10**	**H1_11**	**H1_12a**	**H1_12b**	**H1_13a**	**H1_13b**

Household Number: |__|__|__|__|

Code 1: Agriculture crop codes

Major Cereals	Pulses	Vegetables	Leafy vegetables	Fruits (continued)	Other crops (continued)
B Aus (local) ... 10	Lentil(Moshur) ... 51	Pumpkin ... 101	Pui Shak ... 201	Lemon ... 312	Tobacco ... 601
TAus (local) ... 11	Mung ... 52	Bringal (eggplant) ... 102	Palang Shak (Spinach) 202	Shaddock (pomelo) ... 313	Bettlenut ... 602
TAus (HYV) ... 12	Black gram (Mashkalai) 53	BT Brinjal 1(Bari brinjal	Lal Shak ... 203	Black berry ... 314	Bettleleaf ... 603
T Aus (hybrid) ... 13	Chickling Vetch(Khesari)54	1)…128	Kalmi Shak ... 204	Other fruits(lemon like)315	Other Tobacco like crop604
BAman (local) ... 14	Chick pea (Chhola) ... 55	BT Brinjal 2(Bari brinjal	Danta Shak ... 205	Other fruits ... 316	Cut flower ... 605
T Aman(local) ... 15	Pigeon pea (Aarohor) ... 56	2)…129	Kachu Shak ... 206	Boroi(Bitter Plum) ... 317	
T.Aman (HYV) ... 16	Field pea (Motor) ... 57	BT Brinjal 3(Bari brinjal	Lau Shak ... 207	Rose Apple ... 318	Paddy seedbed ... 701
T.Aman (hybrid) ... 17	Soybean (Gori kalai/	3)…130	Mula Shak ... 208	Wood Apple ... 319	Tomato seedbed ... 702
Boro(local) ... 18	Kali motor) ... 58	BT Brinjal 4(Bari brinjal	Khesari Shak ... 209	Ambada/Hoq Plum ... 320	Bringal seedbed ... 703
Boro (HYV) ... 19	Other Pulses ... 59	4)…131	Other green leafy vegetables	Pomegranate ... 321	Cauliflower seedbed ... 704
Boro (hybrid) ... 20		Patal ... 103	... 210	Bilimbi ... 322	Cabbage seedbed ... 705
Wheat (local) ... 21	**Oil Seeds**	Okra ... 104	Potato Leaves ... 211	Chalta ... 323	Kohlrabi seedbed ... 706
Wheat (HYV) ... 22	Sesame ... 61	Ridge gourd ... 105	Cabbage ... 212	Tamarind(pulp) ... 324	Tobacco seedbed ... 707
Maize ... 23	Linseed(tishi) ... 62	Bitter gourd ... 106	Chinese cabbage ... 213	Olive(wild) ... 325	Onion seedbed ... 708
Barley ... 24	Mustard ... 63	Arum ... 107		Coconut/Green Coconut326	Chili seedbed ... 709
Job ... 25	Ground nut/peanut ... 64	Ash gourd ... 108	**Fruits**		Other seedbed ... 710
Cheena ... 26	Soybean ... 65	Cucumber ... 109	Banana ... 301	**Other crops**	
Kaun(Italian millet) ... 27	Castor (rerri) ... 66	Carrot ... 110	Mango ... 302	Potato ... 411	
Joar(Great millet) ... 28	Others Oilseeds ... 67	Cow pea ... 111	Pineapple ... 303	Sweet potato ... 412	**By Products (Applicable for**
Bojra(Pearl millet) ... 29		Snake gourd ... 112	Jack fruit ... 304	Mulberry(Tunt) ... 413	**Module I only)**
Others ... 30	**Spices**	Danta ... 113	Papaya ... 305	Orange flesh sweet potato414	Jutestick ... 801
**Fiber Crops**	Chili ... 71	Green banana/plantain 114	Water melon ... 306		Straw ... 802
Dhonche ... 41	Onion ... 72	Cauliflower ... 115	Bangi/Phuti/Musk melon307	Sugurcan ... 501	Other byproduct ... 803
Jute ... 42	Garlic ... 73	Water gourd ... 116	Litchis ... 308	Date ... 502	
Cotton ... 43	Turmeric ... 74	Sweet gourd ... 117	Guava ... 309	Palm ... 503	Other(specify) ... 900
Bamboo ... 44	Ginger ... 75	Tomato ... 116	Ataa ... 310	Date Juice ... 504	
Other Fibre ... 45	Dhania/Coriander ... 76	Radish ... 119	Orange ... 311	Tea ... 505	
	Other spices ... 77	Turnip ... 120			
		Green Papaya ... 121			
		Kakrol ... 122			
		Yam Stem ... 123			
		Other green Vegetables124			
		DrumStick ... 125			
		Bean ... 126			
		Coriander leaf ... 127			

Household Number: | | | | |

Code 3: Paddy Rice

Variety	Code
Chandina BR-1 (Boro/Aus)	1
Mala BR-2 (Boro/Aus)	2
Biplob BR-3 (Aus/Aman)	3
Brishail BR-4* (Aman)	4
Dulavhoge BR-5* (Aman)	5
BR-6 (Boro/Aus)	6
Bribalam BR-7 (Boro/Aus)	7
Asa BR-8 (Boro/Aus)	8
Sufoza BR-9 (Boro/Aus)	9
Progoti BR-10 (Aman)	10
Mukta BR-11 (Aman)	11
Moyna BR-12 (Boro/Aus)	12
Gazi BR-14 (Boro/Aus)	13
Mohini BR-15 (Boro/aus)	14
Shahi Balam BR-16(Boro/Aus)	15
Hasi BR-17 (Boro)	16
Shahjalal BR-18 (Boro)	17
Mongal BR-19 (Boro)	18
Nizami BR-20 (Aus)	19
Niamat BR-21 (Aus)	20
Kiron BR-22* (Aman)	21
Dyshary BR-23 ((Aman)	22
Rahmat BR-24 (Aus)	23
Noya Pajam BR-25 (Aman)	24
Sraboni BR-26 (Aus)	25
Bri Dhan BR-27 (Aus)	26
Bri Dhan BR-28 (Boro)	27
Bri Dhan BR-29 (Boro)	28
Bri Dhan BR-30 (Aman)	29
Bri Dhan BR-31 (Aman)	30
Bri Dhan BR-32 (Aman)	31
Bri Dhan BR-33 (Aman)	32
Bri Dhan BR-34 (Aman)	33
Bri Dhan BR-35 (**Boro**)	34
Bri Dhan BR-36 (Boro)	35
Bri Dhan BR-37 (Aman)	36
Bri Dhan BR-38 (Aman)	37
Bri Dhan BR-39 (Aman)	38
Bri Dhan BR-40 (Aman)	39
Bri Dhan BR-41 (Aman)	40
Bri Dhan BR-42 (Aus)	41
Bri Dhan BR-43 (Aus)	42
Bri Dhan BR-44 (Aman)	43
Bri Dhan BR-45 (Boro)	44
Bri Dhan BR-46 (Aman)	45
Bri Dhan BR-47 (Boro)	46
Bri Dhan BR-48 (Aus)	47
Bri Dhan BR-49 (Aman)	48
Bri Dhan BR-50 (Banglamoti)(Boro)	49
Bri Dhan BR-51 (Aman)	50
Bri Dhan BR-52 (Aman)	51
Bri Dhan BR-53 (Aman)	52
Bri Dhan BR-54 (Aman)	53
Bri Dhan BR-55 (boro/aus)	54
Bri Dhan BR-56 (Aman)	55
Bri Dhan BR-57 (Aman)	56
Bri Dhan BR-58 (Boro)	57
Bri Dhan BR-59 (Boro)	58
Bri Dhan BR-60 (Boro)	59
Bri Dhan BR-61 (Boro)	60
Bri Dhan BR-62 Zinc enriched (Aman)	61
Bri Dhan BR-63Shorubalam (Boro)	62
Bri Dhan BR-64Zinc enriched (Boro)	63
Bri Dhan BR-65 (Aus)	64
Bri Dhan BR-66 drought tolerant(Aman)	65
Bri Dhan BR-67saline tolerant (Boro)	66
Bri Dhan BR-68 (boro	67
Bri Dhan BR-69 weed resistant(Boro)	68
Nerica(new rice for africa)	69
Haridhan	70
Asiruddin	71
Kajallata.	72
Khude kajal.	73
Miniket	74
Paijam.	75
Shapla	76
Bashmati.	77
Jamaibabu.	78
Guti/rajshahi/lalshorna	79
Bhojon(white/coarse	80
Binni dhan	81
Tepi dhan.	82
Others	888
**Hybrid**	
Alok..	85
Sonar bangla	86
Jagoron	87
Shakti 1	88
Shakti 2	89
Aloron 1	90
Aloron 2	91
Hira	92
ACI 5	93
Lal Teer	94
BINA 1	95
BINA 2	96
BINA 3	97
BINA 4	98
BINA 5	99
Bri Hybrid-1(Boro)	100
Bri Hybrid-2(Boro)	101
Bri Hybrid-3(Boro)	102
Bri Hybrid-4(Boro Aman)	103
Bina 6(Boro/aus)	104
Bina 7(Aman)	105
Bina 8(Boro/Aus)	106
Bina 10 (Boro)	107
Bina 11(aman/aus)	108
Bina 12 (aman)	109
Bina 13(aman)	110
Bina 14(boro)	111
Bina 15(aman)	112
Bina 16(aman)	113
Binashail(aman)	114
Iratom 24(Boro)	115
Taj	116
HS	117
Shonali	118
Surma	119
Padma	120
Bijoy	121
Borkot	122
Raja	123
Chitra	124
Shobujmoti	125
Kajol	126
Rajkumar	127
Robi	128
Other(specify)	999

Code 4: Source of seed

Source	Code
Own	1
Gift borrow (from neighbor/relative etc)	2
BADC(Government)	3
Bought from BRAC	4
Bought from other NGO	5
Personal shop/dealer	6
Hat/Bazar	7
Other farmer	8
Bought from farmhouse	9
Bought seedlings	10
Made seedling	11

Code 5: Significant characteristics of seeds

Characteristic	Code
Grain Yield	1
Grain Size	2
Insect/disease resistant.	3
Flood tolerant	4
Saline tolerant	5
Drought tolerant	6
Zinc enriched	7
Low labor required	8
Low input required	9
Ease of processing	10
Market demand	11
Good taste	12
Nice color	13
Good as animal feed	14
Others (specify)	15

Code 6: Did you face any problems with the seeds you purchased?

Problem	Code
No problem	1
Mixed with other seed	2
Poor germination	3
Too expensive	4
Incorrect label	5
Tampered or damaged packaging	6
Shortage of supply	7
Others (specify)	8

Code 7: Reason of inadequacy

Reason	Code
No market supply	1
Did not save seed	2
Saved seed got damaged	3
Post- flood scarcity of seedling during 2nd plantation	4
Could not get good quality seed	5
Seedling was damaged	6
Others (specify)	7

Module H2: Irrigation method and Harvest (Male)

Report for plot wise crop data for all Crop cultivated during 1st December (15th Agrahayon) 2017- 30th November (14th Agrahayon) 2018.
If the respondent responds in Bangla months for harvest, please convert those months according to English calendar and write in designated column.

Serial No.	Plot ID	Crop code In case of Intercropping report Crop code for the second crop in the shaded column below.			Irrigation								Harvest				If you have obtained the plot through sharecropping, then how much in crops have you given to the owner?	If you have obtained the plot through sharecropping, then what is the value of the crops that have you given to the owner?	If you have obtained the plot through cash rent, what is the amount that you have paid him?
				Source of water	Irrigation Method<br>If 1 thenH2_06	Energy used for Irrigation<br>Diesel .... 1<br>Electric .... 2<br>Manual 3	Number of times irrigated	Payment method for Irrigation<br>Cash 1<br>Cash & crop share 2<br>Crop share only 3→H2_05a<br>Own irrigation 4→H2_05a	Cash cost of Irrigation	What is the cost of irrigation beyond cash expenditure?	Cause of inadequacy/ insufficiency of water<br>If response is "1" →H2_08	In which month was the water insufficiency greatest (report with respect to the cultivation season)<br>January.....1<br>February...2<br>December12	Time of harvest		Quantity harvested	If partially harvested report Quantity in field			
		Code 1		Code 2 ↓	Code 3	Code ↑	No. of times	Code ↑	Tk	Tk	Code 4	Month (↑)	week	month	Kg.	Kg.	Kg.	Tk	Tk
**Sl. No.**	**ID**	**Crop Code**		**H2_01**	**H2_02**	**H2_03**	**H2_04_1**	**H2_04**	**H2_05**	**H2_05a**	**H2_06**	**H2_07**	**H2_ 08**	**H2_ 09**	**H2_10**	**H2_11**	**H2_12**	**H2_13**	**H2_14**

Code 2: Source of water	Code 3:Irrigation Method	Code 4: Cause of inadequacy & insufficiency of water
Rainfed....................1	Rainfed.................... 1	Adequate water 1
River 2	Swing basket 2	Lack of rainfall.................... 2
Canal....................3	Don.................... 3	Lack of water in river.................... 3
Pond....................4	Dugwell.................... 4	Lack of water in dam.................... 4
Hoar/Beel (Lake)....................5	Hand tubewell 5	Fall in groundwater level.................... 5
Canalirrigation 6	Treddle pump 6	Water too saline 6
Groundwater 7	Rower pump.................... 7	More arsenic in groundwater.................... 7
Tidal water 8	Shallow tubewell.................... 8	Electric failure.................... 8
	Deep tubewell.................... 9	Lack of diesel.................... 9
	Low lift pump.................... 10	Dispute with irrigation organization/supplier 10
	Canal irrigation.................... 11	Irrigation machine disfunction 11
	Axial Flow Pump 12	Other(specify) 12
	Other 13	

Household Number: | | | | |

Module H3: Usage of Agricultural Chemicals, Fertilizers and Pesticides (Male)

Recall period: 1st December (15th Agrahayon) 2017- 30th November (14th Agrahayon) 2018.

If Chemicals/Fertilizers/Pesticides etc. is not applied, report “0” relevant column headings against the Plot ID and crop code ID.

Sl.	Plot ID	Crop code<br><br>In case of Intercropping report Crop code for the second crop in the shaded column below.		Urea	TSP	SSP	DAP	MAP	MP	Zinc	Ammonia	Gypsum	NPKS	Calcium/Lime	Manure/Compost	Have you used vermicompost fertilizer?<br>Yes .....1<br>No ......2> H3_11a	Vermicompost fertilizer was<br><br>Produced .... 1<br>Purchased 2	If purchased, what was the price?	Pesticides/	insecticide	herbicide	Vitamin
		Code 1		Amt (kg)	Amt (kg)		Amt (kg)		Amt (kg)	Amt (kg)	Amt (kg)	Amt (kg)	Amt (kg)	Amt (kg)	Total Value (Tk)	Code ↑	Code ↑	Total Value (Tk)	Total Value (Tk)	Total Value (Tk)	Total Value (Tk)	Total Value (Tk)
**Sl.**	**Plot ID**	**Crop Code**		**H3_01**	**H3_02**	**H3_02b**	**H3_03**	**H3_03b**	**H3_04**	**H3_05**	**H3_06**	**H3_07**	**H3_08**	**H3_09**	**H3_10**	**H3_10a**	**H3_10b**	**H3_10c**	**H3_11a**	**H3_11b**	**H3_11c**	**H3_12**

Household Number: | | | |

Module H4: Rental Cost of Tools, Machinery and Draft Animal (Male)

Recall period: 1st December (15th Agrahayon) 2017- 30th November (14th Agrahayon) 2018.

Report rental cost if tools/machinery are rented solely. If tools/machinery is rented along with labor/operator(s) report total rental cost.

Sl. No.	Plot ID	Crop Code<br><br>In case of Intercropping report Crop code for the second crop in the shaded column below.	For land preparation							Tools/machinery used									
			Animal Used			Machinery Used				For planting		For fertilizer application		For pesticide application		For weeding		For harvesting	
			Have animals been used?	Total bullock days	Cost	Has machinery been used?<br><br>If code2 = 3>>H4_06n	Power tiller=1 Tractor=2 Plough=3 Manual= 4	Rental Cost	Fuel cost	Has machinery been used?<br><br>If code2 = 3>>H4_07n	Cost	Has machinery been used?<br><br>If code2 = 3>>H4_08n	Cost	Has machinery been used?<br><br>If code2 = 3>>H4_09n	Cost	Has machinery been used?<br><br>If code2 = 3>>H4_010n	Cost	Has machinery been used?<br><br>If code2 = 3>>next crop	Cost
		Code 1	Code 2	days	(Tk/ day)	Code 2	Code	(Tk)	(Tk)	Code 2	(Tk)	Code 2	(Tk)	Code 2	(Tk)	Code 2	(Tk)	Code 2	(Tk)
**Sl.**	**Plot ID**	**Crop Code**	**H4_01n**	**H4_01**	**H4_02**	**H4_03n**	**H4_03**	**H4_04**	**H4_05**	**H4_06n**	**H4_06**	**H4_07n**	**H4_07**	**H4_08n**	**H4_08**	**H4_09n**	**H4_09**	**H4_10n**	**H4_10**

**Code 2:**
Yes, I used my own/ I used someone else's free of charge.... 1
Yes, I rented it and then used it 2
No, I have not used it.. 3

Household Number: | | | |

Module H5: Labor Usage by Gender for Crop Plantation and Harvesting (Male)

Recall period: 1st December (15th Agrahayon) 2017- 30th November (14th Agrahayon) 2018.

M refers to male labor; F refers to female labor. Include adult and child labor. Round hours to the whole number. Report “0” only if no labor has been used. For e.g. if plot is weeded for 15 min report 1 hour and not “0”.

Sl. No.	Plot ID	Crop Code In case of Intercropping report Crop code for the second crop in the shaded column below.		Land preparation (Ploughing, Harrowing, Leveling etc.)								Planting (Seeding/Transplanting)								Other Fertilizer (including prilled urea) application							
				**Family**		**Hired**						**Family**		**Hired**						**Family**		**Hired**					
				Hours		Hours	Where were labors hired from	Total cost (Tk)	Hours	Where were labors hired from	Total cost (Tk)	Hours		Hours	Where were labors hired from	Total cost (Tk)	Hours	Where were labors hired from	Total cost (Tk)	Hours		Hours	Where were labors hired from	Total cost (Tk)	Hours	Where were labors hired from	Total cost (Tk)
		Code 1		M	F	M	M	M	F	F	F	M	F	M	M	M	F	F	F	M	F	M	M	M	F	F	F
**Sl.**	**ID**	**Crop Code**		**H5_01**	**H5_02**	**H5_03**	**H5_03a**	**H5_04**	**H5_05**	**H5_05a**	**H5_06**	**H5_07**	**H5_08**	**H5_09**	**H5_09a**	**H5_10**	**H5_11**	**H5_11a**	**H5_12**	**H5_19**	**H5_20**	**H5_21**	**H5_21a**	**H5_22**	**H5_23**	**H5_23a**	**H5_24**

Household Number: | | | |

Module H5: Labor Usage by Gender for Crop Plantation and Harvesting (continued) (Male)

Recall period: 1st December (15th Agrahayon) 2017- 30th November (14th Agrahayon) 2018.
M refers to male labor; F refers to female labor. Include adult and child labor. Round hours to the whole number. Report "0" only if no labor has been used. For e.g. if plot is weeded for 15 min report 1 hour and not "0".

Plot ID	Crop Code*	Pesticide application								Weeding								Irrigation (channel maintenance etc.)								Harvest							
		**Family**		**Hired**						**Family**		**Hired**						**Family**		**Hired**						**Family**		**Hired**					
		Hr		Hours	Where were labors hired from	Total cost (Tk)	Hours	Where were labors hired from	Total cost (Tk)	Hr		Hours	Where were labors hired from	Total cost (Tk)	Hours	Where were labors hired from	Total cost (Tk)	Hr		Hours	Where were labors hired from	Total cost (Tk)	Hours	Where were labors hired from	Total cost (Tk)	Hr		Hours	Where were labors hired from	Total cost (Tk)	Hours	Where were labors hired from	Total cost (Tk)
	Code 1	M	F	M	M	M	F	F	F	M	F	M	M	M	F	F	F	M	F	M	M	M	F	F	F	M	F	M	M	M	F	F	F
**Plot**	**Crop Code**	**H5_25**	**H5_26**	**H5_27**	**H5_27a**	**H5_28**	**H5_29**	**H5_29a**	**H5_30**	**H5_31**	**H5_32**	**H5_33**	**H5_33a**	**H5_34**	**H5_35**	**H5_35a**	**H5_36**	**H5_37**	**H5_38**	**H5_39**	**H5_39a**	**H5_40**	**H5_41**	**H5_41a**	**H5_42**	**H5_43**	**H5_44**	**H5_45**	**H5_45a**	**H5_46**	**H5_47**	**H5_47a**	**H5_48**

Household Number: | | | |

Module H6: Post Harvest Labor, Animal and Tools/ Machinery Usage (Male)

Report for all Crop cultivated during 1st December (15th Agrahayon) 2017- 30th November (14th Agrahayon) 2018.

The questions in this section are at crop level and NOT plot level.

M refers to male labor; F refers to female labor. Include adult and child labor. Round hours to the whole number only.Report “0” only if no labor has been used. For e.g. if plot is weeded for 15 min report 1 hour and not “0”.

Crop	Carrying from farm to home						Animal used for threshing		Tools/ machinery used for threshing		Threshing					
	Family		Hired				Total days	Total Cost			Family		Hired			
	Hours		Hours	Total cost (Tk)	Hours	Total cost (Tk)					Hours		Hours	Total cost (Tk)	Hours	Total cost (Tk)
Code 1	M	F	M	M	F	F	days	(Tk)	Code 2	(Tk)	M	F	M	M	F	F
**Crop Code**	**H6_01**	**H6_02**	**H6_03**	**H6_04**	**H6_05**	**H6_06**	**H6_07**	**H6_08**	**H6_09a**	**H6_09b**	**H6_10**	**H6_11**	**H6_12**	**H6_13**	**H6_14**	**H6_15**

Household Number: | | | | |

Module H6: Post Harvest Labor, Animal and Tools/ Machinery Usage (continued) (Male)

Report for all Crop cultivated during 1st December (15th Agrahayon) 2017- 30th November (14th Agrahayon) 2018.

The questions in this section are at crop level and NOT plot level.

M refers to male labor; F refers to female labor. Include adult and child labor.

Round hours to the whole number only. Report “0” only if no labor has been used. For e.g. if plot is weeded for 15 min report 1 hour and not “0”.

Crop	Drying crop						Sorting						Packaging					
	Family		Hired				Family		Hired				Family		Hired			
	Hours		Hours	Total cost (Tk)	Hours	Total cost (Tk)	Hours		Hours	Total cost (Tk)	Hours	Total cost (Tk)	Hours		Hours	Total cost (Tk)	Hours	Total cost (Tk)
Code 1	M	F	M	M	F	F	M	F	M	M	F	F	M	F	M	M	F	F
**Crop Code**	**H6_16**	**H6_17**	**H6_18**	**H6_19**	**H6_20**	**H6_21**	**H6_22**	**H6_23**	**H6_24**	**H6_25**	**H6_26**	**H6_27**	**H6_28**	**H6_29**	**H6_30**	**H6_31**	**H6_32**	**H6_33**

**Code 2:**
Yes, I used my own/ I used someone else’s free of charge................. 1
Yes, I rented it and then used it 2
No, I have not used it.. 3

Module H7: Fertilizer, Seed and Pesticides price in different crop seasons (Male)

Report for the three seasons during 1st December (15th Agrahayon) 2017- 30th November (14th Agrahayon) 2018.

Sl no	Name of Fertilizer/ Pesticide	Price of Fertilizer/Seed						Was there any inadequacy of fertilizer in the last 12 months? Yes .... 1 No...... 2>> **H7_11**	Record the code for the month in which there was an inadequacy or insufficiency of fertilizer		What do you think was the reason for this inadequacy?		Was there any problems of fertilizer?
		Rabi		Kharif 1		Kharif 2							
		Did you purchase? Yes... 1 No 2→**H7_04**	Avg price per Kg	Did you purchase? Yes .. 1 No .. 2→**H7_06**	Avg price per Kg	Did you purchase? Yes ... 1 No 2→**H7_08**	Avg price per Kg						
		Code ↑	Tk/Kg	Code ↑	Tk/Kg	Code ↑	Tk/Kg	Code ↑	Code 1		Code 2		
**H7_01**		**H7_02**	**H7_03**	**H7_04**	**H7_05**	**H7_06**	**H7_07**	**H7_08**	**H7_09a**	**H7_09b**	**H7_10a**	**H7_10b**	**H7_11**
1	Urea												
10	Briquette Urea												
21	TSP /												
22	SSP												
31	DAP												
32	/MAP												
4	MP												
5	Zinc												
6	Ammonia												
7	Gypsum												
8	NPKS												
9	Calcium /Lime												
91	Lime												
92	Calcium												
99	Without cultivation												

**Code 1: Month**	**Code 2: Cause of Inadequacy**		**Code 3: Fertilizer problem**
January.....................1<br>February...................2<br>................................<br>December.................12	Was not readily available in the market...... 1<br>Dealer hoarding... 2<br>Insufficient Supply...................................... 3<br>No dealer nearby... 4<br>Smuggling of fertilizer................................ 5	Others.. 6	No problem….1<br>Adultrated with salt…2<br>Adultrated with soil…..3<br>Adultrated with other things…4<br>Clotted….5<br>Productivity was less …..6

Household Number: | | | |

Module H7_A: Labor Cost: For sowing seed, weed control, irrigation and harvesting crops. (Male)

Report for the three seasons during 1st December (15th Agrahayon) 2017- 30th November (14th Agrahayon) 2018.

Sl. No.	Different Purposes of Labor Use	Cost of Labor (Male)									Cost of Labor (Female)								
		Rabi (Boro)			Kharif 1 (Aus)			Kharif 2 (Aman)			Rabi (Boro)			Kharif 1 (Aus)			Kharif 2 (Aman)		
		Did you hire labor?<br>Yes......1<br>No....... 2> H7a_05	Daily Rate	Daily Payment in kind (incl. food)	Did you hire labor?<br>Yes . 1<br>No. 2> H7a_08	Daily Rate	Daily Payment in kind (incl. food)	Did you hire labor?<br>Yes......1<br>No....... 2 > H7a_11	Daily Rate	Daily payment in kind (incl. food)	Did you hire labor?<br>Yes .....1<br>No....... 2> H7a_14	Daily Rate	Daily Payment in kind (incl. food)	Did you hire labor?<br>Yes......1<br>No....... 2> H7a_17	Daily Rate	Daily payment in kind (incl. food)	Did you hire labor?<br>Yes..... 1<br>No....... 2> next row	Daily Rate	Daily payment in kind (incl. food)
		Code ↑	Tk	Value in Tk	Code ↑	Tk	Value in Tk	Code ↑	Tk	Value in Tk	Code ↑	Tk	Value in Tk	Code ↑	Tk	Value in Tk	Code ↑	Tk	Value in Tk
	**H7a_01**	**H7a_02**	**H7a_03**	**H7a_04**	**H7a_05**	**H7a_06**	**H7a_07**	**H7a_08**	**H7a_09**	**H7a_10**	**H7a_11**	**H7a_12**	**H7a_13**	**H7a_14**	**H7a_15**	**H7a_16**	**H7a_17**	**H7a_18**	**H7a_19**
1	Labor needed for land preparation																		
2	Labor needed for sowing seeds																		
3	Labor needed for broadcasting seeds																		
4	Labor needed for weeding																		
5	Labor needed for manual irrigation																		
6	Labor needed for collecting crops																		
7	Labor needed for applying briquette urea fertilizer																		
8	Labor needed for applying other fertilizer																		
9	Labor needed for threshing																		

Module H8: Location of purchase/rent of Animals, Tool/Machineries, Agricultural labor, Fertilizer, Pesticides etc. (Male)

Recall period: 1st December (15th Agrahayon) 2017- 30th November (14th Agrahayon) 2018.

Sl No.	Question	Response	Response code
H8_01	Where did you rent animal used in land preparation and threshing from?		Farm gate (home)...1<br>Village market (within own village)..............2<br>Village market (outside of own village)........3<br>Town market..4<br>Other (specify)...5<br>Did not rent and did not buy 9
H8_02	Where did you rent tools and machineries used in crop cultivation from?		
H8_03	Where did you hire agricultural labor from?		
H8_04	Where did you purchase fertilizer/pesticides/insecticide/herbicide from?		

Module End Time: | Hour | | | Minute | | |

Household Number: | | | | |

Module I: Summary of Agriculture Production and Food grain stock (Male)

Module I1: Summary of Agriculture Production (crops, fruits and vegetables) (Male)

These questions are only for crops, not plots.
Crop cultivation in last 1 year, December 1 2017to November 30, 2018.

Module start time: Hour | | | Minu | | **Respondent ID:** | | Consent : | | Yes ...1 No.....2

Note: Write value of total sale proceeds in taka.
Note: Report both for cultivated and non cultivated produce. Bring information on byproduct as well. For e.g. Report the household's non-cultivated jackfruit harvest from jackfruit trees in homestead, which should be recorded in Module I3.

Crop Code **(from module H)**	Qty harvested	Qty received from leased out plot	Qty paid to land owner of leased in plot	Qty Paid for irrigation	Qty paid to labors/rent for machinary/ equipment	Qty consumed	Qty Given away	Animal feed	Wastage		Qty sold If "0" →I1_14	Where sold?	Point of sale		Total Sale Price	Amount kept for Seed for next year	Current stock (on survey day)
									Amount	Reason			How far is the place? If "1", then put "0"	Time taken to travel If "1", then put "0"			
Crop Code 1	(kg)	(kg)	(kg)	(kg)	(kg)	(kg)	(kg)	(kg)	(kg)	Code 1	(kg)	Code2	km	min	(Tk)	(kg)	(kg)
**Crop code**	**I1_01**	**I1_02**	**I1_03**	**I1_04**	**I1_05**	**I1_06**	**I1_07**	**I1_08**	**I1_09a**	**I1_09b**	**I1_10**	**I1_11**	**I1_12a**	**I1_12b**	**I1_13**	**I1_14**	**I1_15**

Household Number: | | | |

Code1: Reason for loss of output	Code 2: Point of Sale
Flood/rain 1	Farm gate (home) 1
Pest attack 2	Village market (within own village) 2
Infested by rats 3	Village Market (outside own village) ...3
Drought 4	Town Market 4
Storm/cyclone 5	Other (specify)...................................... 5
Other(specify) 6	Neither bought nor rented in..................9

Module I2: Food grain stock and Storage capacity (Male)

For last 1 year, December 1 2017to November 30, 2018

Food grain			End of Month food grain stock												Maximum amount food grain storage capacity of the household
			Dec 2017	Jan 2018	Feb 2018	Mar 2018	Apr 2018	May 2018	June 2018	July 2018	August 2018	Sep 2018	Oct 2018	Nov 2018	
			kg	kg	kg	kg	kg	kg	kg	kg	kg	kg	kg	kg	kg
**I2_01**			**I2_02**	**I2_03**	**I2_04**	**I2_05**	**I2_06**	**I2_07**	**I2_08**	**I2_09**	**I2_10**	**I2_11**	**I2_12**	**I2_13**	**I2_14**
Paddy															
Rice															
Wheat															
Have you milled paddy in past one year for your own consumption? yes=1; No=2>> next machine			Dec 2017	Jan 2018	Feb 2018	Mar 2018	Apr 2018	May 2018	June 2018	July 2018	August 2018	Sep 2018	Oct 2018	Nov 2018	
Huller mill		Amount of paddy/rice processed													
		Amount of rice received													
Husking mill		Amount of paddy/rice processed													
		Amount of rice received													
Semi-automatic mill		Amount of paddy/rice processed													
		Amount of rice received													

Household Number: | | | | |

Module I2a: Food grain stock and Storage capacity (continued) (Male)

	Kharif 2 (Aman)								Kharif 1 (Aus)								Robi (Boro)							
Crop code	First sale after how many days of production? Did not produce/sold….99>>I2_18	Qty sold?	Price of sale	Last sale after how many days of production? Did not produce/sold….99>>I2_23	Qty sold?	Price of sale	Till now, what volume/qty. of your production have you sold?	What is the current quantity of your stock? (As of Date of survey)	First sale after how many days of production? Did not produce/sold….99>>I2_26	Qty sold?	Price of sale	Last sale after how many days of production? Did not roduce/sold….99>>I2_31	Qty sold?	Price of sale	Till now, what volume/qty. of your production have you sold?	What is the currentquantity of your stock? (As of Date of survey)	First sale after how many days of production? Did not produce/sold….99>>I2_34	Qty sold?	Price of sale	Last sale after how many days of production? Did not produce/sold….99>>next crop	Qty sold?	Price of sale	Till now, what volume/qty. of your production have you sold?	What is the current quantity of your stock? (As of Date of survey)
Crop	day	kg	Tk/kg	kg	kg	Tk/kg	kg	kg	day	kg	Tk/kg	kg	kg	Tk/kg	kg	kg	day	kg	Tk/kg	kg	kg	Tk/kg	kg	kg
	**I2_15**	**I2_16**	**I2_17**	**I2_18**	**I2_19**	**I2_20**	**I2_21**	**I2_22**	**I2_23**	**I2_24**	**I2_25**	**I2_26**	**I2_27**	**I2_28**	**I2_29**	**I2_30**	**I2_31**	**I2_32**	**I2_33**	**I2_34**	**I2_35**	**I2_36**	**I2_37**	**I2_38**
Paddy																								
Rice																								
Wheat																								

Household Number: | | | |

Module I3: Nonplot food production in both inside and outside homestead (Male)

Write down the list of Nonplot food produced in both inside and outside the homestead during last 1 year (1st December, 2017 to 30th November, 2018). Report both for cultivated and non cultivated produce. For e.g. Report the household's non-cultivated jackfruit harvest from jackfruit trees in homestead.

Is this nonplot within homestead or nonplot outside homestead?<br>Nonplot inside homestead ..... 1<br>Nonplot small garden inside homestead 2<br>Nonplot outside homestead ... 3<br>Small garden beside pond/water body 4	Crop name	**Crop code**	Total quantity produced	Qty consumed	Qty sold	Total receipt from sale	Did u receive any NGO/any other organization assistance for growing this crop?<br><br>Yes .... 1<br>No.................... 2>>next crop		If Yes, what type of help?<br>Cash ....1<br>Seed ....2<br>Fertilizer ....3<br>Training ....4<br>Irrigation pump...................5<br>Others (specify) 6		
		Code 1<br>Crop code	Kg	kg	kg	Tk	Code ↑	NGO Code	Code ↑		
I3_01	I3_02	I3_03	I3_04	I3_05	I3_06	I3_07	I3_08	NGO Code	I3_09a	I3_09b	I3_09c

NGO Code:

BRAC 1
ASA 2
PROSHIKA........................ 3
Karitas Bangladesh........................ 4
Shwanirbhar Bangladesh........................ 5
TMSS........................ 6
RDRS Bangladesh........................ 7
Bureau Tangail........................ 8
Jagoroni Chakra 9
Voluntary Organization for Social Development (VOSD) 10
Peoples Oriented Program Implementation (POPI)........................ 11
Gono Kalyan Trust (GKT) 12
Bachte Shekha........................ 13
PKSF........................ 14
Bangladesh Rural Development Board(BRDB) 15
PodokhepManobik Unnyan Kendra 17
Heed Bangladesh 18
Bureau Bangladesh 19
Community Development Center (CODEC)........................ 20
Gono Milon Foundation........................ 21
Shapla Ful........................ 22
Sheba Manob kolyan Kendra (SMKK) 23
Society for Disadvantaged Origin (SDO)........................ 24
Akota Shomaj Unnyan Kendra (ASUK) 25
Bangladesh Development Society........................ 26
Social Organization for Voluntary Advancement (SOVA) 27
Society development Committee (SDC) 28
Faridpur Development Agency (FDA) 29
Ashar Alo Unnyan Shangstha 30
Polli Progoti Sohayok Samity 31
Samadhan........................ 32
Manob Seba Sangstha........................ 33
Nobolok Parishad........................ 34
Rural reconstruction Foundation (RRF)........................ 35
Christian Civil Society (CSS) 36
Uddipon 37
Daak diye jai........................ 38
Shushilon 39
Uttaran 40
Unnyan Procheshta 41
Save Bangladesh........................ 42
Satkhira Unnyan Shangstha (SUK)........................ 43
Ideal 44
Manob Sompod Unnyan kendra........................ 45
Grameen bank........................ 46
HKI (Helen Keller International) 47
Other NGOs (specify) 48

Household Number: | | | | |

Module I4: Seedling/seedbed production cost (Male)

For last 1 year, December 1 2017 to November 30, 2018

I4_1 Have you produced own seedling/seedbed for any crop last year? Yes …….1 No………2 >> Next Module

Name of each crops for which you produced seedlings					Season<br>Aman (Kharif 2)..1<br>Aus (Kharif1)....2<br>Boro (Rabi)…3<br>Annual….4	What is the area of reference for the seedbeds ?	Quantity of seed used for seedbed to produce seedling	Main source of seed<br>If produced seedling, write'11' in I4_05a, and mention seed source in the next column		Price per kg of seed used?<br>If seed source code is 1 or 2, bring approximate cost of seed	In your opinion, what are the the most important traits in this seed?		Did you face any problem with the seed/seedling you purchased?<br>If ans is 1>>next plot	Reason of inadequacy	Did you sell any seedlings or give to others?<br>Yes... 1<br>No .... 2>>I4_11	If yes, what portion of seedling you sold or gave to other?	Total area planted with own produced seedlings?	Total expenditure of other inputs used in the seed bed						
																		Chemical fertilizers		Organic fertilizers		Pesticides	Hired labor	Other
Plot ID	Crop name	Code1 CropCode	If rice, variety name	Variety Code	Code ↑	decimals	Kg	Code 2		Price/Kg	Code3		Code4	Code5	Code ↑	%	decimals	Kg	Tk	Kg	Tk	Total Tk	Tk	Tk
		I4_01		I4_02	I4_03	I4_04	I4_05	I4_06a	I4_06b	I4_07	I4_08a	I4_08b	I4_09	I4_10	I4_11	I4_12	I4_13	I4_14	I4_15	I4_16	I4_17	I4_18	I4_19	I4_20

**Code 2: Source of seed**		**Code 3: Significant characteristics of seed**		**Code 4: Did you face any problems with the seed you purchased?**	**Code 5: reason of inadequacy**
Own/Saved seeds 1	Market 7	Grain Yield 1	Low input required ... 9	No problem 1	No market supply 1
Gift from others.................2	Other farmer............ 8	Grain Size 2	Ease of processing 10	Mixed with other seed....................2	Did not save seed............................2
BADC outlet (Govt.)..........3	Bought from farm 9	Insect/disease resistant 3	Market demand.........11	Poor germination............................3	Saved seed got damaged..................3
Bought fromBRAC 4	Bought seedling........ 10	Flood tolerant.................4	Good taste 12	Too expensive 4	Post- flood scarcity of seedling during 2nd plantation......................4
Bought from other NGO 5	Produced seedling 11	Saline tolerant 5	Nice color 13	Incorrect label 5	Could not get good quality seed… ..5
personal shop/dealer...........6		Drought tolerant.............6	Good as animal feed . 14	Tampered or damaged packaging....6	New seedling was damaged.............6
		Zinc enriched 7	Others (specify)…... . 15	Shortage of supply 7	others (specify)….. 7
		Low labor required…......8		Others (specify) 8	

Consent<br>Yes=1 No=2	MID	

Household Number: | | | | |

Module ha: Agricultural Technologies - paddy rice (Male)

For last 1 year, December 1 2017 to November 30, 2018.

Interviwer: (1) If no member of any household has not cultivated paddy in last 1 year, go to the next module.

(2) Check ALL the answers of this section with module H

Q. No.	Technology Questions	Response	Response codes
HA_01	What kind of land preparation did you use for the paddy rice you planted in the past year?<br><br>[Multiple Answers possible]		None....1>>HA_06<br>ZERO TILLAGE....2<br>Ploughing....3<br>Hand weeding....4<br>Others(specify)....96
HA_02	Check HA_01: did respondent use zero tillage methods to prepare the land?		yes....1<br>no....2>>HA_04
HA_03	What kind of zero tillage system did you use on the land where you planted paddy rice?<br><br>[Multiple Answers possible]		Slash And Plant....1<br>Burn And Plant....2<br>Herbicide And Plant....3<br>Others(specify)....96
HA_04	Check HA_01: did respondent use ploughing methods to prepare the land?		yes....1<br>no....2>>HA_06
HA_05	What did you use for ploughing the land where you planted paddy rice [Multiple Answers Possible]		HAND TILLAGE (HOE)....1<br>ANIMAL TRACTION....2<br>MOTORIZED TILLER....3<br>TRACTOR....4<br>OTHER (SPECIFY)....96
HA_06	What was your main source of rice seed?		OWN SAVED SEED, SEED FROM FRIEND/<br>RELATIVE (NOT PURCHASED)....1<br>RELATIVE (NOT PURCHASED)....2<br>Prchased from market (non-Ag dealer)....3<br>Purchased from ag-dealer with cash....4<br>Purchased from ag-dealer with voucher....5<br>Aid Distribution....6<br>Subsidy card....7<br>NGO....8<br>OTHER (SPECIFY)....96

Household Number: [ ][ ][ ][ ]

Q. No.	Technology Questions	Response	Response codes
HA_07	What type of seed did you plant in the past year?<br>[Multiple answer possible]	[ ][ ][ ][ ]	Local (broadcast)..1<br>Local (transplanted) ....2<br>HYV ....3<br>Hybrid 4
HA_08	CHECK HA_07: DID RESPONDENT SELECT MORE THAN ONE TYPE OF SEED?	[ ]	Yes ....1<br>No..2>>HA_10
HA_09	Would you say that most of the rice seed you planted was traditional, local seed, HYV or hybrid?	[ ]	MOSTLY TRADITIONAL/LOCAL SEED1<br>MOSTLY HYV/hybrid ....2<br>Half was local and rest half was HYV/hybrid 3<br>Do not know...8
HA_10	When decisions are made regarding what kind of rice seed to plant, who is it that normally takes the decision?<br>[MULTIPLE RESPONSE]<br><br>**Record multiple responses only when there are more than one decision-maker**	[ ]<br>[ ]<br>[ ]	self...1<br>spouse..2<br>other household member ....3<br>other non-household member......................4
HA_11	Did you grow your rice crop: To provide food for the household?<br>To be sold or traded in the market?<br>Both for food and for the market?	[ ]	household consumption only.......................1<br>selling only...2<br>both for household consumption and selling3<br>others(specify)...96
HA_12	Did you sow rice seed directly in the paddy, did you plant seedlings in your rice paddy, or did you both sow seed directly and plant some seedlings?	[ ]	SEED SOWN DIRECTLY ONLY1>>HA_16<br>ONLY SEEDLINGS PLANTED................2<br>SOME SEED SOWN, SOME SEEDLINGS3
HA_13	Did you start your own seedlings, or did you purchase them?	[ ]	STARTED OWN SEEDLINGS..1>>HA_15<br>PURCHASED SEEDLINGS ....2<br>BOTH STARTED OWN SEEDLING AND PURCHASED..3
HA_14	Was the nursery where you purchased the rice seedlings a registered or certified nursery?	[ ]	yes...1 No.....2
HA_15	Check Ha_12: DID THE FARMER PLANT ONLY SEEDLINGS (RESPONSE 2)?	[ ]	YES, ONLY SEEDLINGS PLANTED1>>HA_17<br>NO, SOME SEEDS SOWN DIRECTLY 2
HA_16	Some farmers plant paddy rice seeds in rows and some randomly broadcast their paddy rice seeds. How did you plant paddy rice seeds?	[ ]	IN ROWS...1<br>RANDOMLY BROADCAST ....2<br>SOME IN ROWS AND SOME RANDOMLY BROADCAST 3
HA_17	Some farmers plant paddy rice alone and some plant their rice along with other crops growing in the same plot. How did you plant your rice?	[ ]	ALONE ....1>>HA_19<br>WITH OTHER CROPS 2

Household Number: [ ] [ ] [ ] [ ]

Q. No.	Technology Questions	Response			Response codes
		CROP- 1	CROP - 2	CROP- 3	
HA_18	What other crops did you plant in the same plot with the paddy rice?<br><br>SELECT THE NAME OF THE CROP(S) FROM THE DROP DOWN LIST	[ ]	[ ]	[ ]	[CROP CODE]
HA_19	Do you raise fish in your rice paddy?		[ ]		yes...1 No.....2>>HA_21
HA_20	Why do you raise fish in your rice paddy?<br>[Multiple response]	[ ]	[ ]	[ ]	For large profit .... 1<br>Fish feed is less expensive .... 2<br>Weeds of the paddy field can be used for fish feed<br>.. 3<br>Others(specify)... 96
HA_21	When decisions are made regarding whether or not to plant other crops in the same plot with your rice, who is it that normally takes the decision?<br>[MULTIPLE RESPONSE]	[ ]	[ ]	[ ]	self.. 1<br>spouse... 2<br>other hh member .... 3<br>other non-hh member 4
HA_22	Over the past two planting seasons, did you rotate your paddy rice with another crop planted in the same plot area?		[ ]		yes...1<br>No.....2>>HA_24<br>do not know................................3>>HA_24
		crop - 1	crop - 2	crop - 3	
HA_23	What was cultivated in the plot in the season before you planted the rice?<br><br>[MULTIPLE RESPONSE]	[ ]	[ ]	[ ]	[crop code]
HA_24	Did you apply fertilizer to the paddy rice in the past year?		[ ]		yes...1<br>No.....2>>HA_28
HA_25	At which times did you apply fertilizer to the paddy rice?<br><br>[MULTIPLE RESPONSE]		[ ]<br>[ ]<br>[ ]		Land preparation stage .... 1<br>planting .... 2<br>early growth stage .... 3<br>mid crop .... 4<br>others(specify).. 96
HA_26	What type of fertilizer did you use?<br>[MULTIPLE RESPONSE]		[ ]<br>[ ]<br>[ ]		SOIL BASED ORGANIC......................... . 1<br>SOIL BASED INORGANIC .... 2<br>FOLIAR FEEDS ORGANIC ……….3<br>FOLIAR FEEDS INORGANIC….4<br>Others(specify).. 96

Household Number: | | | | |

Q. No.	Technology Questions	Response	Response codes	
HA_27	What is the name of the fertilizer you mainly used for your rice in the past year?<br><br>[MULTIPLE RESPONSE]		Urea….1<br>Guti urea…2<br>TSP…3<br>SSP…4<br>DAP…..5<br>MAP…6<br>MP….7	Zinc….8<br>Ammonia….9<br>Zypsum…10<br>NPKS….11<br>Others(specify)…12<br>Donot know…13<br>Donot use chemical fertilizer…..14
HA_28	Did you apply animal manure to your rice fields in the past year?		yes...1 No.....2>>HA_31	
HA_29	How was animal manure applied to your rice fields?		Animals graze & leave dung on field…1<br>Manure applied to field by hand… 2<br>Manure applied to field with machine…3<br>Others(specify)....96	
HA_30	Where did you get the manure you applied to your rice fields?<br>[MULTIPLE RESPONSE]		Produced by own animals .... 1<br>Given by family/friends/neighbours' farm.. 2<br>Purchased from market .... 3<br>Others(specify)....96	
HA_31	When decisions are made regarding whether or not to use fertilizer for your rice, who is it that normally takes the decision?<br>[MULTIPLE RESPONSE]<br><br>**Record multiple responses only when there are more than one decision-maker**		Self .... 1<br>Spouse .... 2<br>Other hh member .... 3<br>Other non hh member 4	
HA_32	Have you been trained in how to use inorganic fertilizer for paddy rice?<br><br>Inorganic fertilizer is a man-made fertilizer that you can buy in a bag at the shop.		Yes...1 No.....2	
HA_33	Did you have any insect, rodent, or disease attacks on your paddy rice in the past year?		Yes...1 No.....2	
HA_34	Did you use chemicals to control insect, rodent, or disease attacks on the paddy rice?		Yes...1 No.....2>>Ha_36	
HA_35	Was the use of chemicals preventive, or was it in response to an insect, rodent, or disease attack?		Preventive .... 1<br>Protective .... 2<br>both preventive and protective 3	
HA_36	Have you been trained in when to use and how to apply pesticides for paddy rice?		Yes...1 No.....2	

Household Number: [ ] [ ] [ ] [ ]

Q. No.	Technology Questions	Response		Response codes
HA_37	How many times did you control weeds among your paddy rice crops in the past year?			Frequency (number)<br>None..00>>ha_39
HA_38	How did you control the weeds among your paddy rice crops?<br><br>[MULTIPLE RESPONSE]			Hoe...1<br>Herbicide..2<br>Intercropping..3<br>Slashing...4<br>Pull by hand 5
HA_39	Have you ever been trained in when to use and how to apply herbicides for paddy rice crops?			Yes...1 No.....2
HA_40	In the past year, did you use any of the following techniques to manage soil and water for your paddy rice crop?	a) Terracing		Yes...1 No.....2
		b) soil bands/trenches		Yes...1 No.....2
		c) adding lime to soil		Yes...1 No.....2
		Other (specify)_______________ _		Yes...1 No.....2
HA_41	Besides rainfall, did you use any additional irrigation methods for the paddy rice?			Yes...1 No.....2>>HA_43
HA_42	What type of irrigation did you use?<br>[MULTIPLE RESPONSE]			By hand (watering can, hose, etc.) ....1<br>Canals...2<br>Permanent hose ....3<br>Pump ....4<br>Othrs (specify)..96
HA_43	How did you harvest the paddy rice?			With a sickle ....1<br>With a scythe..2<br>With a motorized harvester ....3<br>Not yet harvested 4

Household Number: | | | | |

Q. No.	Technology Questions	Response	Response codes
HA_44	What did you dry the paddy rice on?<br>[MULTIPLE RESPONSE]		Bare ground..1<br>Ground plastered with cow dung ....2<br>Left to dry on plant in field ....3<br>Tarpaulins ....4<br>Matress made by sewing plastic bag or sack….5<br>Matress made by sewing bag or sack….6<br>Net made of nilon………7<br>Drying yard with cemented ground.............8<br>Drying racks...9<br>Solar dryers ....10<br>Mechanized dryers ....11<br>Othrs (specify)..96
HA_45	How did you thresh the rice?<br><br>[MULTIPLE RESPONSE]		Trampled by cattle/oxen….1<br>Beat with sticks…2<br>Beat with a flail….3<br>Used a treadle thresher…..4<br>Used a motorized thresher….5<br>Did not thresh….6>>ha_47<br>Othrs (specify).......................................96
HA_46	After threshing the rice, what was done with the straw?<br>[MULTIPLE RESPONSE]		Burned..1<br>Incorporated back into the soil…..2<br>Used as bedding for own livestock….3<br>Used as cooking fuel…..4<br>Left in field for grazing by animals…..5<br>Fed to own animals…..6<br>Sold to others….7<br>Gave to others ....8<br>Othrs (specify)..96
HA_47	Did you put the paddy rice in bags or other containers after harvest for storage or transport?<br>[MULTIPLE RESPONSE]		Yes, in buckets ....1>>ha_49<br>Yes, in drums ....2>>ha_49<br>Yes, in bags...3<br>No..4

Household Number: | | | | |

Q. No.	Technology Questions	Response		Response codes
HA_48	What type of storage bag did you use for the paddy rice?<br>[MULTIPLE RESPONSE]			Woven bag, single layer .... 1<br>Two- or three-layered woven bags.............. 2<br>Hermetic bag 3
HA_49	Did you use any of the following storage locations to store the paddy rice?	সংরক্ষণস্থল		
		a) Residential house?		Yes...1 No.....2
		b) Cribs?		
		c) Granaries?		
		d) Other constructed stores?		
		e) Warehouses?		
		f) Storage silos?		
		g) OTHER (SPECIFY)		
	CHECK ALL CATEGORIES FOR HA_49, IF ANY YES (CODE '1') THEN CONTINUE, OTHERWISE SKIP TO HA_51			
HA_50	Was your paddy rice attacked by insects, rodents, or disease while in storage?			Yes...1 No.....2
HA_51	What information source do you rely on the most to help you grow your rice crop well?			Friend/neighbor .... 1<br>Agro-input dealer .... 2<br>Ag extension worker .... 3<br>School .... 4<br>Radio program….5<br>Television……….6<br>Mobile phone messaging…..7<br>Internet…..8<br>Others (specify)……96

Module End Time: | Hour | | | Minute | | |

Household Number: | | | | |

Module I5: Access to Technologies (Male)

I5a: When was the household formed (year)? ______

I5b: Which year did you start rice farming? ______

I5c: Which year did you start farming? _____

Module start time: Hour | | | Minu | | | **Respondent ID:** | | Consent : | | Yes ...1 No.....2

Code	List of Technologies	Are you aware of this tech? Yes ..1 No …2	Do you use this technology now? Yes .... 1 No 2	If yes, is it rented or owned? Rented …1 Owned …..2	Have you used this technology in the past? Yes .... 1 No 2	When did you start using this technology? …..9999>>I5_06	If you have used this technology in the past, and no longer use it, when did you stop using it? Have not stopped …9999	Reason for not using now?	When was this technology first available in your village? ..... 9999>next row	Please mark the years that you have used this technology within the past 10 years? Yes... 1 No 2									
										2018	2017	2016	2015	2014	2013	2012	2011	2010	2009
			Code		Code	(year)	(year)	Code1	(year)	Code	Code	Code	Code	Code	Code	Code	Code	Code	Code
**I5_01**	**Description**	**I5_17**	**I5_02**	**I5_18**	**I5_03**	**I5_04**	**I5_05**	**I5_05b**	**I5_06**	**I5_07**	**I5_08**	**I5_09**	**I5_10**	**I5_11**	**I5_12**	**I5_13**	**I5_14**	**I5_15**	**I5_16**
**1**	Guti Urea																		
**2**	BRRI dhan BR-41 (aman)																		
**3**	BRRI dhan BR-43 (aus)																		
**4**	BRRI dhan BR-44 (aman)																		
**5**	BRRI dhan BR-46 (aman)																		
**6**	BRRI dhan BR-47 (boro)																		
**7**	BRRI dhan BR-50 (Banglar moti) (boro)																		
**8**	BRRI dhan BR-51 (aman)																		
**9**	BRRI dhan BR-52 (aman)																		

Household Number: | | | | |

Code	List of Technologies	Are you aware of this tech? Yes ..1 No …2	Do you use this technology now? Yes .... 1 No 2	If yes, is it rented or owned? Rented …1 Owned …..2	Have you used this technology in the past? Yes .... 1 No 2	When did you start using this technology? …..9999>>I5_06	If you have used this technology in the past, and no longer use it, when did you stop using it? Have not stopped …9999	Reason for not using now?	When was this technology first available in your village? ..... 9999>next row	Please mark the years that you have used this technology within the past 10 years? Yes... 1 No 2									
										2018	2017	2016	2015	2014	2013	2012	2011	2010	2009
			Code		Code	(year)	(year)	Code1	(year)	Code	Code	Code	Code	Code	Code	Code	Code	Code	Code
**I5_01**	**Description**	**I5_17**	**I5_02**	**I5_18**	**I5_03**	**I5_04**	**I5_05**	**I5_05b**	**I5_06**	**I5_07**	**I5_08**	**I5_09**	**I5_10**	**I5_11**	**I5_12**	**I5_13**	**I5_14**	**I5_15**	**I5_16**
**10**	BRRI dhan BR-53 (aman)																		
**11**	BRRI dhan BR-54 (aman)																		
**12**	BRRI dhan BR-55 (boro/aus)																		
**13**	BRRI dhan BR-56 (aman)																		
**14**	BRRI dhan BR-57 (aman)																		
**15**	BRRI dhan BR-60 (boro)																		
**16**	BRRI dhan BR-61 (boro)																		
**17**	BRRI dhan BR-62 (Zinc) (aman)																		
**18**	BRRI dhan BR-64 (Zinc) (boro)																		
**19**	BRRI dhan BR-66 (drought																		

Household Number: | | | | |

Code	List of Technologies	Are you aware of this tech? Yes ..1 No …2	Do you use this technology now? Yes .... 1 No 2	If yes, is it rented or owned? Rented …1 Owned …..2	Have you used this technology in the past? Yes .... 1 No 2	When did you start using this technology? …..9999>>I5_06	If you have used this technology in the past, and no longer use it, when did you stop using it? Have not stopped …9999	Reason for not using now?	When was this technology first available in your village? ..... 9999>next row	Please mark the years that you have used this technology within the past 10 years? Yes... 1 No 2									
										2018	2017	2016	2015	2014	2013	2012	2011	2010	2009
			Code		Code	(year)	(year)	Code1	(year)	Code	Code	Code	Code	Code	Code	Code	Code	Code	Code
**I5_01**	**Description**	**I5_17**	**I5_02**	**I5_18**	**I5_03**	**I5_04**	**I5_05**	**I5_05b**	**I5_06**	**I5_07**	**I5_08**	**I5_09**	**I5_10**	**I5_11**	**I5_12**	**I5_13**	**I5_14**	**I5_15**	**I5_16**
	tolerant) (aman)																		
**20**	BRRI dhan BR-67 (saline tolerant) (boro)																		
**21**	BRRI dhan BR-69 (weed resistant) (boro)																		
**22**	Bina - 7 (aman)																		
**23**	Bina - 8 (boro/aus)																		
**24**	Bina - 11 (aus/aman)																		
**25**	Bina - 12 (aman)																		
**26**	Four wheel Tractor																		
**27**	Two wheel tractor (Power tiller)																		
**28**	Axial flow pump/ (jumbo pump)																		

Household Number: [ ] [ ] [ ] [ ]

Code	List of Technologies	Are you aware of this tech? Yes ..1 No …2	Do you use this technology now? Yes .... 1 No 2	If yes, is it rented or owned? Rented …1 Owned …..2	Have you used this technology in the past? Yes .... 1 No 2	When did you start using this technology? …..9999>>I5_06	If you have used this technology in the past, and no longer use it, when did you stop using it? Have not stopped …9999	Reason for not using now?	When was this technology first available in your village? ..... 9999>next row	Please mark the years that you have used this technology within the past 10 years? Yes… 1 No …. 2									
										2018	2017	2016	2015	2014	2013	2012	2011	2010	2009
			Code		Code	(year)	(year)	Code1	(year)	Code	Code	Code	Code	Code	Code	Code	Code	Code	Code
**I5_01**	**Description**	**I5_17**	**I5_02**	**I5_18**	**I5_03**	**I5_04**	**I5_05**	**I5_05b**	**I5_06**	**I5_07**	**I5_08**	**I5_09**	**I5_10**	**I5_11**	**I5_12**	**I5_13**	**I5_14**	**I5_15**	**I5_16**
**29**	Irrigation pump (LLP)																		
**30**	Irrigation pump (Shallow)																		
**31**	Irrigation pump (Deep)																		
**32**	Reapers																		
**33**	Paddle thresher																		
**34**	Power thresher																		
**35**	Seeder (Seeder Drills: till, plant, and fertilize simultaneously)																		
**36**	Bed Planters (form fields into beds and furrows)																		
**37**	Fertilizer Sprayer																		

Household Number: | | | | |

Code	List of Technologies	Are you aware of this tech? Yes ..1 No …2	Do you use this technology now? Yes .... 1 No 2	If yes, is it rented or owned? Rented …1 Owned …..2	Have you used this technology in the past? Yes .... 1 No 2	When did you start using this technology? …..9999>>I5_06	If you have used this technology in the past, and no longer use it, when did you stop using it? Have not stopped …9999	Reason for not using now?	When was this technology first available in your village? ..... 9999>next row	Please mark the years that you have used this technology within the past 10 years? Yes… 1 No 2									
										2018	2017	2016	2015	2014	2013	2012	2011	2010	2009
			Code		Code	(year)	(year)	Code1	(year)	Code	Code	Code	Code	Code	Code	Code	Code	Code	Code
**I5_01**	**Description**	**I5_17**	**I5_02**	**I5_18**	**I5_03**	**I5_04**	**I5_05**	**I5_05b**	**I5_06**	**I5_07**	**I5_08**	**I5_09**	**I5_10**	**I5_11**	**I5_12**	**I5_13**	**I5_14**	**I5_15**	**I5_16**
**38**	Pesticide Sprayer																		
**41**	Combined harvester																		
**42**	Rice transplanter																		
**43**	Closed drum thresher																		
**44**	Open drum thresher																		
**45**	Corn sheller																		
**46**	Sugarcane crusher																		
**47**	Winnower																		
**39**	Others (specify)																		
**40**	Others (specify)																		

Code 1: Reason for not using:<br>Not available in the market…1<br>Do not know about the technology…..2	Replaced with new tech...3<br>It became obsolete…4<br>Technology is inappropriate….5	Expensive….6<br>Don't have the operating skill…..7<br>Not in working condition now….8	Spare parts are not available…9<br>Lack of skilled mechanic to repair….10<br>No rental market so cannot be rented…..11

Module End Time: | Hour | | | Minute | | |

Household Number: | | | |

Module J: Agricultural Extension Services and Subsidies (Male)

Hour | | | Minute | | | **Respondant ID:** | | Respondant's Consent: | | Yes...... 1 No....... 2

Module J1: Access to Agriculture Extension Services (Male)

For last 1 year, December 1 2017 to November 30 2018.

Question Number	Questions	Response	Code
**J1**	Did you grow any crop during last 12 months?		Yes .... 1<br>No.................................... 2>> **Module J1a**
**J1_01**	Did any agricultural extension agent visit your farm during the last 12 months?		Yes .... 1<br>No.................................... 2>> **J1_04**
**J1_01b**	If yes, who did the agent communicate with?		Only male..1<br>Only female…2<br>Both male and female…..3
**J1_02**	**How many times did s/he come?**		
**J1_02a**	From government extension service office		(Report frequency of visit. Report '0' if not visited)
**J1_02b**	If J1_02a is not "0", who did the agent communicate with?		Only male..1<br>Only female…2<br>Both male and female…..3
**J1_02b**	From NGO (________________________)		(Report frequency of visit. Report '0' if not visited)
**J1_02c**	From Other (________________________)		(Report frequency of visit. Report '0' if not visited)
**J1_03**	**Did you receive advice on the following?**		
**J1_03a**	Fertilizer use		Yes .... 1<br>No.................................... 2>>**J1_03c**
**J1_03b**	Was the advice given useful?		Yes, very .... 1<br>Yes, somewhat .... 2<br>No.................................... 3
**J1_03c**	Seed use		Yes .... 1<br>No.................................... 2>>**J1_03e**
**J1_03d**	Was the advice given useful?		Yes, very .... 1<br>Yes, somewhat .... 2<br>No.................................... 3
**J1_03e**	Irrigation use		Yes .... 1<br>No.................................... 2>>**J1_03g**

Question Number	Questions	Response	Code
**J1_03f**	Was the advice given useful?	[ ]	Yes, very .... 1<br>Yes, somewhat .... 2<br>No.................................... 3
**J1_03g**	Pesticide use	[ ]	Yes .... 1<br>No.................................... 2>>**J1_03i**
**J1_03h**	Was the advice given useful?	[ ]	Yes, very .... 1<br>Yes, somewhat .... 2<br>No.................................... 3
**J1_03i**	Pest and Diseases	[ ]	Yes .... 1<br>No.................................... 2>>**J1_03k**
**J1_03j**	Was the advice given useful?	[ ]	Yes, very .... 1<br>Yes, somewhat .... 2<br>No.................................... 3
**J1_03k**	Cropping practice	[ ]	Yes .... 1<br>No.................................... 2>>**J1_03m**
**J1_03l**	Was the advice given useful?	[ ]	Yes, very .... 1<br>Yes, somewhat .... 2<br>No.................................... 3
**J1_03m**	Soil type	[ ]	Yes .... 1<br>No.................................... 2>>**J1_03o**
**J1_03n**	Was the advice given useful?	[ ]	Yes, very .... 1<br>Yes, somewhat .... 2<br>No.................................... 3
**J1_03o**	Others(--)	[ ]	Yes .... 1<br>No.................................... 2>>**J1_04**
**J1_03p**	Was the advice given useful?	[ ]	Yes, very .... 1<br>Yes, somewhat .... 2<br>No.................................... 3
**J1_04**	Did you go to any extension agent or contacted over phone?	[ ]	Yes .... 1<br>No.................................... 2 >>**J1_07**
**J1_04a1**	If yes, who went or who contacted over phone?	[ ]	Only male..1<br>Only female…2<br>Both male and female…..3
**J1_05**	**How many times did you visit or contact the following?**		
**J1_5a**	Government extension service office	[ ]	(Report frequency of visit. Report ‘0’ if not visited)

Household Number:

Question Number	Questions	Response	Code
**J1_05b**	If J1_5a is not “0”, who did the agent communicate with?		Only male..1<br>Only female…2<br>Both male and female…..3
**J1_5b**	NGO (________________________)		(Report frequency of visit. Report ‘0’ if not visited)
**J1_5c**	Other (________________________)		(Report frequency of visit. Report ‘0’ if not visited)
**J1_06**	**Did you receive advice on the following?**		
**J1_06a**	Fertilizer use		Yes .... 1<br>No.................................... 2>>**J1_06c**
**J1_06b**	If yes, was the advice given useful?		Yes, very .... 1<br>Yes, somewhat .... 2<br>No.................................... 3
**J1_06c**	Seed use		Yes .... 1<br>No.................................... 2>>**J1_06e**
**J1_06d**	If yes, was the advice given useful?		Yes, very .... 1<br>Yes, somewhat .... 2<br>No.................................... 3
**J1_06e**	Irrigation use		Yes .... 1<br>No.................................... 2>>**J1_06g**
**J1_06f**	If yes, was the advice given useful?		Yes, very .... 1<br>Yes, somewhat .... 2<br>No.................................... 3
**J1_06g**	Pesticide use		Yes .... 1<br>No.................................... 2>>**J1_06i**
**J1_06h**	If yes, was the advice given useful?		Yes, very .... 1<br>Yes, somewhat .... 2<br>No.................................... 3
**J1_06i**	Pest and Diseases		Yes .... 1<br>No.................................... 2>>**J1_06k**

Household Number: | | | | |

Question Number	Questions	Response	Code
**J1_06j**	If yes, was the advice given useful?	☐	Yes, very .... 1<br>Yes, somewhat .... 2<br>No.................................... 3
**J1_06k**	Cropping practice	☐	Yes .... 1<br>No.................................... 2>>**J1_06m**
**J1_06l**	If yes, was the advice given useful?	☐	Yes, very .... 1<br>Yes, somewhat .... 2<br>No.................................... 3
**J1_06m**	Soil type	☐	Yes .... 1<br>No.................................... 2>>**J1_06o**
**J1_06n**	If yes, was the advice given useful?	☐	Yes, very .... 1<br>Yes, somewhat .... 2<br>No.................................... 3
**J1_06o**	Others(-------)	☐	Yes .... 1<br>No.................................... 2
**J1_06p**	If yes, was the advice given useful?	☐	Yes, very .... 1<br>Yes, somewhat .... 2<br>No.................................... 3
**J1_07**	If no, why? (report primary reason)<br><br>**(answer this question if answers to J1_01 and J1_04 are no)**	☐	Did not face any problems 1<br>Extension officer non-helpful 2<br>Extension officer not knowledgeable................. 3<br>Extension office too far away 4<br>Do not know of any extension service office .... 5<br>Others 6
**J1_08**	Did you attend farmers' field school in last 12 months?	☐	Yes .... 1<br>No.................................... 2 >>**J1_10**
**J1_09**	If yes, who attended?		Only male..1<br>Only female…2<br>Both male and female…..3
	Did you receive advice on the following?		

Household Number: | | | |

Question Number	Questions	Response	Code
**J1_09a**	Fertilizer use		Yes .... 1<br>No.................................... 2>> **J1_09c**
**J1_09b**	If yes, was the advice given useful?		Yes, very .... 1<br>Yes, somewhat .... 2<br>No.................................... 3
**J1_09c**	Seed use		Yes .... 1<br>No.................................... 2>>**J1_09e**
**J1_09d**	If yes, was the advice given useful?		Yes, very .... 1<br>Yes, somewhat .... 2<br>No.................................... 3
**J1_09e**	Irrigation use		Yes .... 1<br>No.................................... 2>>**J1_09g**
**J1_09f**	If yes, was the advice given useful?		Yes, very .... 1<br>Yes, somewhat .... 2<br>No.................................... 3
**J1_09g**	Pesticide use		Yes .... 1<br>No.................................... 2>>**J1_09i**
**J1_09h**	If yes, was the advice given useful?		Yes, very .... 1<br>Yes, somewhat .... 2<br>No.................................... 3
**J1_09i**	Pest and Diseases		Yes .... 1<br>No.................................... 2>>**J1_09k**
**J1_09j**	If yes, was the advice given useful?		Yes, very .... 1<br>Yes, somewhat .... 2<br>No.................................... 3
**J1_09k**	Cropping practice		Yes .... 1<br>No.................................... 2>>**J1_09m**
**J1_09L**	If yes, was the advice given useful?		Yes, very .... 1<br>Yes, somewhat .... 2<br>No.................................... 3
**J1_09m**	Soil type		Yes .... 1<br>No.................................... 2>>**J1_09o**
**J1_09n**	If yes, was the advice given useful?		Yes, very .... 1<br>Yes, somewhat 2

Question Number	Questions	Response	Code
			No.....................................3
**J1_09o**	Others(---)	[ ]	Yes ....1<br>No.....................................2>>**Next Module**
**J1_09p**	If yes, was the advice given useful?	[ ]	Yes, very ....1<br>Yes, somewhat ....2<br>No.....................................3
**J1_10**	If no, why? (report primary reason)<br><br>(answer this question if answers to J1_01 and J1_04 are no)	[ ]	Did not face any problems 1<br>Extension officer unhelpful 2<br>Extension officer not knowledgeable................. 3<br>Extension office too far away 4<br>Do not know of any extension service office .... 5<br>Others 6
**J1_11**	Did you ever have any communication with any agricultural extension agent? (including farmers' field school)	[ ]	Yes ....1<br>No..................................... 2
**J1_12**	If yes, who had a communication?	[ ]	Only male..1<br>Only female…2<br>Both male and female…..3

Household Number: | | | | |

Module J1a: Access to livestock and fisheries Extension Services (Male)

For last 1 year, December 1 2017 to November 30 2018.

Question Number	Questions	livestock	Poultry	Fish	Code
**J1a_1**	Did you produce any livestock/poultry/fish during last 12 months?				Yes...................................1<br>No .... 2>>Next Module
**J1a_01**	Did any livestock/fisheries extension agent visit your farm during the last 12 months?				Yes...................................1<br>No ....2>>J1a_04
**J1a_01b**	If yes, who communicated with the agent?				Only male..1<br>Only female…2<br>Both male and female…..3
**J1a_02**	How many times did s/he come?				
**J1a_02a**	From government livestock/fisheries extension service office				(Report frequency of visit. Report '0' if not visited)
**J1a_02b**	If J1a_02a is not '0', who communicated with the agent?				Only male..1<br>Only female…2<br>Both male and female…..3
**J1a_02b**	From NGO (________________________)				(Report frequency of visit. Report '0' if not visited)
**J1a_02c**	From Other (________________________)				(Report frequency of visit. Report '0' if not visited)
**J1a_04**	Did you go to any extension agent or contact one over phone?				Yes...................................1<br>No ....2>>**Next Module**
**J1a_04a**	If yes, who communicated with the agent contact one over phone?				Only male..1<br>Only female…2<br>Both male and female…..3
**J1a_05**	If yes, how many times did you visit the following agent/agency/organization?				
**J1a_05a**	Government livestock/fisheries extension service agent				(Report frequency of visit. Report '0' if not visited)
**J1a_05b**	If J1a_5a is not "0" who communicated with the agent?				Only male..1<br>Only female…2<br>Both male and female…..3
**J1a_05b**	NGO livestock/fisheries extension service agent (________________________)				(Report frequency of visit. Report '0' if not visited)
**J1a_05c**	Other livestock/fisheries extension service agent (________________________)				(Report frequency of visit. Report '0' if not visited)

Household Number: | | | | |

Module J2a: Government agriculture input subsidy card related information (Male)

For last 1 year, December 1 2017 to November 30 2018.

Question Number	Question	Response		Code
**J2a_01**	Have you heard about the farmer's input subsidy card or farmer's agricultural input subsidy card?			Yes .... 1<br>No.................................... 2 >>**Next Module**
**J2a_02**	Do/Did you have an agriculture input subsidy card?			Yes .... 1<br>No.................................... 2 >>**Next Module**
**J2a_03**	When did you receive the input subsidy card?	Year	Month	Month: January=1……December =12
**J2a_04**	How did you come to know about this card? (report primary source of information on subsidy card)			Response Code:<br>1. From UP Chairman<br>2. From UP member<br>3. From NGO worker<br>4. From school teacher<br>5. Courtyard meetings/ from radio<br>6. From TV<br>7. News papers<br>8. From friends/neighbors<br>9. Village Campaign<br>10. Sms from government<br>11. Recorded calls from GOB/NGO<br>12. Sub Assistant Agriculture Officer/SAAO<br>13. Village Police<br>14. Other (specify)
**J2a_05**	Did you open a bank account using agriculture input subsidy card to get assistance?			Yes .... 1<br>No.................................... 2
**J2a_06**	Do you receive or did you receive any agricultural input assistance?			Yes, get now……………………1<br>Previously got, now don't……….2>>J2a_09<br>Never received…………………..3>>J2a_10

J2a_07		If answer to "5" is yes, mention the quantity of subsidy								
	**Item**	Yes….1<br>No…...2>> next column	Cash (Taka)	Seed (kg)	Urea (kg)	TSP (kg)	DAP (kg)	MoP (kg)	Other fertilizer (kg)	Pesticide (kg)
		a	b	c	d	e	f	g	h	i
**J2a_07_01**	Wheat									
**J2a_07_02**	Maize									
**J2a_07_03**	Mustard									
**J2a_07_04**	Groundnut									
**J2a_07_05**	Sesame									
**J2a_07_06**	Summer mung									
**J2a_07_07**	Winter mung									
**J2a_07_08**	Khesari									
**J2a_07_09**	Mashkalai									
**J2a_07_10**	Phelon									
**J2a_07_12**	BT brinjal									
**J2a_07_13**	Boro									
**J2a_07_14**	Aman									
**J2a_07_15**	Aus NERICA									
**J2a_07_16**	Other Aus									
**J2a_07_17**	Other1									
**J2a_07_18**	Other 2									
**J2a_07_19**	Other 3									

Household Number: | | | | |

**J2a_08**	Did you buy any agricultural machinery at a subsidized price?			Yes .... 1 No 2>> Next Module
	If yes	Yes=1<br>No=2>> Next column	Number of farmers hhs share this machinery	What percentage of the price did you have to pay to buy it? (percentage)
		a	b	c
**J2a_08_01**	Tractor (4wheel)			
**J2a_08_02**	Shallow tubewell engine or pump			
**J2a_08_03**	LLP			
**J2a_08_04**	Power tiller			
**J2a_08_05**	Combined harvestor			
**J2a_08_06**	Reaper			
**J2a_08_07**	Power thresher			
**J2a_08_08**	Seeder			
**J2a_08_09**	Rice Trans planter			
**J2a_08_10**	Other1			
**J2a_08_11**	Other2			

**J2a_09**	If you don't get it now, what is the reason?		Do not cultivate the crop for which subsidy is given…………1<br>Amount of operating land did not qualify (decreased/increased in size) …….2<br>Do not know………………………3<br>Other reasons (specify)……………4
**J2a_10**	If never received, what is the reason?		Do not cultivate the crop for which subsidy is given…….1<br>I was not selected for this subsidy…….2<br>Amount of operating land is more than 250 decimal……….3<br>Amount of operating land is less than 5 decimal……….4<br>Do not know…………5<br>Others……………….6

Module End Time:

Hour			Minute		

Household Number: | | | | |

Module K: Livestock and Poultry (Male)
Module K1: Livestock and poultry (Male)

For last 1 year, December 1 2017 to November 30 2018.

Module Start Time: Hour | | | Minute | | | **Respondant ID:** | | Respondant's Consent: | | Yes......1 No.......2

Animal Name	Animal Code	Type of asset* Consumption only .... 1 Consumption & Productive .... 2 Productive only .... 3 No livestock 4	On December 1, 2017, how many were there and what was their value?		On November 30, 2018, how many were there and what was their value?		How many does the household own?	Total value of the animal	Who is the owner of the livestock/poultry? Report 3 primary owners. If HH member, write MID. If outside household, use code 1.			Who looks after livestock/poultry? Report 3 primary owners. If HH member, write MID. If outside household, use code 1.			In last 12 months (December 1, 2017 to November 30, 2018.)					
			No	Total value	No	Total value									How many were born?	How many were purchased? If not purchased, write "0", and move toK1_11	Where did you buy animal/bird from? Farm gate (home) .... 1 Village market (within own village) .... 2 Village market (outside of own village) .... 3 Town market .... 4 Other (specify) 5	Who decided to buy livestock and poultry? Report 3 primary owners. If HH member, write MID. If outside household, use code 1.		
	Code	Code ↑	No	Tk	No	Tk	No	Tk	MID/Code 1			MID/Code 1			No.	No.	Code ↑	MID/Code 1		
**Name**	**Livestock Code**	**K1_01**	**K1_02a**	**K1_02b**	**K1_03a**	**K1_03b**	**K1_04**	**K1_04a**	**K1_05a**	**K1_05b**	**K1_05c**	**K1_06a**	**K1_06b**	**K1_06c**	**K1_07**	**K1_08**	**K1_09**	**K1_10a**	**K1_10b**	**K1_10c**
Bullock	1																			
Milk	2																			
Buffalo	3																			
Goat	4																			
Sheep	5																			

Household Number: | | | | |

Pig	51															
Chicken (Layer)	61															
Chicken (Broiler)	62															
Chicken (Cockere	63															
Duck	8															
Other birds (specify)	9															
Others (specify)	10															

**Code 1:**
All members jointly .... 71<br>Male outside household.......... 72<br>Female outside household. ..... 73<br>Govt./Khas land/other institutions<br>.. 74<br>Not applicable/No decision taken<br>...98

Module K1: Livestock and poultry (continued) (Male)

Animal Name	Animal Code	In last 12 months (December 1 2017 to November 30 2018.), how many were							Where did you sell animal?<br><br>Farm gate (home) ....1<br>Village market (within own village).....2<br>Village market (outside of own village) 3<br>Town market ...4<br>Other (specify).5	Total value of selling	Who decided to sell?<br><br>Report 3 primary owners.<br>If HH member, write MID.<br>If outside household, use code 1.			Who controls the sales proceed of the sale of livestock products<br><br>Report 3 primary owners.<br>If HH member, write MID.<br>If outside household, use code 1.			If you raise poultry, have any of your poultry been affected by bird flu within the last 12 months?<br><br>Yes....................1<br>No.....................2<br>Not applicable...9
		Received as gift/Inherited?	Given as gift?	Received from lease	Lost (stolen/ burnt/ spoilt/ died)		Consumed by household	Sold (if not sold, write '0', and go to next row. If duck/ chicken/ bird then go to K1_21)									
					Number	Total value											
	Code	No	No	No	No	Tk	No	No	Code ↑	Tk	MID/Code1			MID/Code1			
**Name**	**Code**	**K1_ 11**	**K1_ 12**	**K1_ 13**	**K1_ 14a**	**K1_ 14b**	**K1_ 15**	**K1_16**	**K1_17**	**K1_18**	**K1_19a**	**K1_19b**	**K1_19c**	**K1_20a**	**K1_20b**	**K1_20c**	**K1_21**

Bullock	1														
Milk Cow	2														
Buffalo	3														
Goat	4														
Sheep	5														
Pig	51														
Chicken (Layer)	61														
Chicken (Broiler)	62														
Chicken (Cockerel)	63														
Duck	8														
Other birds specify)	9														
Others (specify)	10														

**Code 1:**
All members jointly 71
Male outside household.... 72
Female outside household.......73
Govt / Khas land/other institutions 74
Not applicable........................ 98

Module End Time: | Hour | | | Minute | | |

Household Number: [ ][ ][ ][ ]

Module ITMP1A: Improved Technology and Management Practices (ITMP) (Male)

Module start time: Hour [ ][ ] Minu [ ][ ] **Respondent ID:** [ ] Consent : [ ] Yes ...1 No.....2

Question Number	Question	Response	Code
**ITMP1A_01**	Do you rear livestocks?		Yes……………………………………………1<br>No……………………………………………2>>Next Module **K2**
**ITMP1A_02**	If yes, what is the main reason?		For consumption .... 1<br>To sell .... 2<br>Both for consumption and selling purpose………………………………3
**ITMP1A_03**	Do you use improved and appropriate varieties of breed management through artificial insemination?		Yes .... 1<br>No 2
**ITMP1A_04**	Where do you usually keep the livestock?		Livestock shed .... 1<br>Inside the house... 2<br>In open space in the yard............................. 3<br>Others (specify)... 4

Household Number: | | | | |

ModuleITMP1B: **Improved livestock housing and feeding management practices (Male)**

Question Number	Question	Response		Code
**ITMP1B_01**	If the livestocks are kept in the shed, observe what the floor is made of.	☐		Cement cast ....1<br>Made of brick (without welding).................2<br>Earthen floor...3<br>Others 4
**ITMP1B_02**	If the livestocks are kept in the shed, observe if the floor is dry.	☐		Yes, dry ....1<br>No, not dry (wet) 2
**ITMP1B_03**	Do you have a separate manager for watering and feeding?	☐		Yes...1<br>No..2
**ITMP1B_04**	Who mainly provides food and water for the livestocks?	☐		MID<br>Hired male labor.......................................74<br>Hired female labor....................................75
**ITMP1B_05**	Do you use concentrated feed and/or total mixed ration (TMR) and/or ready feed?	☐		Yes...1<br>No..2
**ITMP1B_06**	In the last week (7 days), did you feed any of the grass/livestock food from the right column, to your livestock:	a) Napier 1, 2 & 3	☐	Yes...1<br>No..2
		b) Pukchong	☐	
		c) German grass	☐	
		d) Sweet jumbo	☐	
		e) Jumbo gold	☐	
		f) Para	☐	
		g) Corn cob	☐	
		h) Sage grass	☐	
		i) Pea grass	☐	
		j) Others/ local grass	☐	

Household Number: | | | |

Module ITMP1B: Improved livestock health management practices (Male)

Question Number	Question	Response	Code
**ITMP1B_07**	In the last one year, you vaccinate your livestock's?		Yes .... 1<br>No 2
**ITMP1B_08**	In the last one year, you feed deworming tablets to your livestock?		Yes .... 1<br>No 2
**ITMP1B_09**	In the last one year, how many times did you get your livestock's primary health checked?<br>Note: including any visits to vets		Number<br>If not checked ever, write "0"

Module ITMP1C: Improved practices of handling, preservation, processing and storage meat, milk, and dairy products (Male)

Question Number	Question	Situation	Response	Code
**ITMP1C_01**	Who in the household mainly handles raw meat, milks the cow and prepares dairy products?			MID<br>Hired male labor........ 74<br>Hired female labor........ 75
**ITMP1C_02**	Does the person who mainly handles raw meat, milks the cow and prepares dairy products wash hands with soap, water, detergent etc. before performing the tasks mentioned <u>in the column on the right</u>?	a) After defecation		Yes .... 1<br>No........ 2<br>N/A........ 3
		b) Before eating		
		c) After managing child feces		
		d) Before feeding child		

Household Number: [ ] [ ] [ ] [ ]

Question Number	Question	Response		Code
		e) Before handling food	[ ]	
		f) Between handling raw meat/ foods and ready-to-eat foods	[ ]	
		g) Before milking cow	[ ]	
**ITMP1C_03**	Does the person who mainly handles raw meat, milks the cow and prepares dairy products wash hands with soap, water, detergent etc. before performing the tasks mentioned in the column on the right?	A) Before handling raw meat/ foods and ready-to-eat foods	[ ]	Yes .... 1<br>No........ 2<br>N/A........ 3
		B) Before milking cow	[ ]	
		C) Before preparing dairy products	[ ]	
**ITMP1C_04**	How do you preserve the items on the right column?	A) Meat	[ ]	Refrigerate........ 1<br>Store in ice box to preserve .... 2<br>Boil it .... 3<br>Do not preserve .... 4<br>Others 5
		B) Milk	[ ]	
		C) Dairy products	[ ]	
**ITMP1C_05**	Do you have to frequently boil the items on the right to store it?<br>Enumerator: Only take the answer if the person boils the items to store it	A) Meat	[ ]	Yes .... 1<br>No........ 2
		B) Milk	[ ]	
		C) Dairy products	[ ]	

Household Number: | | | | |

Module ITMP1D: Preparing diversified dairy products (Male)

Question Number	Question	Response	Code
**ITMP1D_01**	Do you prepare yoghurt (at household level)?		Yes ...... 1<br>No...... 2
**ITMP1D_02**	Do you prepare ghee (at household level)?		Yes ...... 1<br>No...... 2
**ITMP1D_0**	Do you make laban (only for processor level)?		Yes ...... 1<br>No...... 2

Module K1a: Agricultural technologies - dairy cows (milk) (Male)

Recall period: For last 1 year, December 1 2017 to November 30 2018

Serial	Question	Response	Response Code
K1a_04	How many calves under one year old do you own?	Number of calves:	Write number of calves<br><br>Do not own any...... 00
K1a_03	How many male animals (bull/bullock/buffalo), that are at least one year old, do you own?	Number of adult males:	Write number of adult male<br><br>Do not own any...... 00
K1a_02	How many dairy cows do you own that are not milking?	Number of non-milking cows:	Write number of non-milking cows<br><br>Do not own any...... 00
K1a_01	How many dairy cows do you own that are milking?	Number of milking cows:	Write number of miling cows<br><br>Do not own any...... 00
	If the answers to K1a_02 and K1a_01 are "0", i.e. if they do not keep any dairy cows, move on to the next module		
K1a_05	How do you acquire your milking cows?<br><br>[SELECT ALL THAT APPLY]		Farmer buys milking cows ...... 1<br>Farmer breeds milking cows ...... 2<br>Farmer gets cows as in-kind credit ...... 3<br>Received as gift / borga...... 4<br>Received through programs ...... 5<br>Other (specify) 96

Household Number: | | | |

Serial	Question	Response	Response Code
K1a_06	How do you breed your dairy cows?<br><br>[SELECT ALL THAT APPLY]		Natural Breeding .... 1<br>Artificial Insemination .... 2<br>Other (Specify)... 96
K1a_07	*CHECK K1a_06: DOES RESPONDENT BREED COWS USING NATURAL BREEDING (RESPONSE '1')?*		Yes .... 1<br>No.. 2> K1a_11
K1a_08	How are bull services acquired to breed your dairy cows?<br><br>[SELECT ALL THAT APPLY]		Bulls Selected from Own Herd .... 1<br>Bulls Exchanged with Other Herds .... 2<br>Bulls Purchased/Rented from Other Herds 3<br>Other (Specify)...96
K1a_09	How do you decide which bulls to choose for breeding your dairy cows and heifers?<br><br>[SELECT ALL THAT APPLY]		No selection - only one bull .... 1<br>No selection - multiple bulls in herd .... 2<br>Bull has good body size, composition......... 3<br>Bull is son of high-producing cow .... 4<br>Bull known to have good fertility .... 5<br>Other (specify) 96
K1a_10	When decisions are made regarding which bull to choose for breeding your dairy cows and heifers, who is it that normally takes the decision?<br><br>[SELECT ALL THAT APPLY]<br><br>**Record multiple responses only when there are more than one decision-maker**		Self .... 1<br>Partner/spouse .... 2<br>Other household member .... 3<br>Other non-household member..................... 4
K1a_11	*CHECK Q. K1a_06: DOES RESPONDENT USE ARTIFICIAL INSEMINATION SERVICES (RESPONSE '2')?*		Yes .... 1<br>No.. 2>>K1a_13
K1a_12	Where do you usually obtain artificial insemination services to breed your dairy cows?		Public veterinary services provider .... 1<br>Community animal health worker............... 2<br>Private services provider .... 3<br>Private vet pharmacy.................................. 4<br>Cooperatives .... 5<br>Other (specify) 96
K1a_13	Would you say that your dairy cows are mostly local, traditional breeds, or would you say that your dairy cows are mostly exotic, modern breeds?		Mostly local/traditional breeds.................... 1<br>Mostly exotic/modern breeds .... 2<br>Half local, half exotic................................. 3<br>Don't know 8
K1a_14	Which of the following is the best description of the housing for your dairy cows?		Kept in family housing............................... 1<br>No housing 2

Household Number: | | | | |

Serial	Question	Response	Response Code
			Open corral only .... 3<br>Roof only, no sides...................................... 4<br>Roof and sides, dirt floor............................. 5<br>Roof and sides, concrete floor...6
K1a_15	How do you usually supply water to your dairy cows?		Cattle drink from pond/pool........................ 1<br>Cattle drink from creek/stream.................... 2<br>Water brought to cattle by buckets............. 3<br>Water piped to cattle .... 4<br>Other (specify) 96
K1a_16	Do your dairy cows graze?		Yes ....1<br>No..2
K1a_17	In the past one year, what forages have you fed your dairy cows?<br><br>Forages are crop, cereal or legume residues, and cultivated fodders.<br><br>[SELECT ALL THAT APPLY]		None .... 1<br>Conserved rice straw .... 2<br>Conserved maize stover .... 3<br>Legume haulms/stovers .... 4<br>Forage legumes .... 5<br>Napier grass .... 6<br>Guinea grass .... 7<br>Cut fresh grass .... 8<br>Tree fodder (leaves, branches) .... 9<br>Cereal .... 10<br>Other (specify) 96
K1a_18	Where did you get this fodder?<br><br>[SELECT ALL THAT APPLY]		Gathered from Roadside .... 1<br>Weeds Pulled from Cropland .... 2<br>Grass Grew Naturally .... 3<br>Bought from Neighbor .... 4<br>Bought from Market .... 5<br>Own produce .... 6<br>Other (Specify) 96
K1a_19	In the past one year, did you feed simple crop by-products to your dairy cows?<br>A simple crop by-product is a kind of supplemental feed that is made from the parts of a plant that are left over after the main crop is harvested, like stems or seed pods. Farmers can buy simple crop by-products or make their own.		Yes .... 1<br>No..2>>K1a_23
K1a_20	How often do you feed simple crop by-products to your dairy cows?		Daily... 1<br>Weekly .... 2<br>Monthly.. 3<br>Whenever available..................................... 4

Household Number:

Serial	Question	Response	Response Code
			Other (specify) 6
K1a_21	What kind of simple crop by-products did you feed to your dairy cows?<br><br>[SELECT ALL THAT APPLY]		Maize Bran .... 1<br>Wheat Bran .... 2<br>Molasses .... 3<br>Fruit Or Vegetable Processing Waste .... 4<br>Oilseed Cake .... 5<br>Brewer's Grain .... 6<br>Cottonseed Meal/Cake .... 7<br>Rice Bran .... 8<br>Other (Specify) 96
K1a_22	Where did you get the simple crop by-products you fed to your dairy cows?<br><br>[SELECT ALL THAT APPLY]		Own Food Preparation .... 1<br>Local Brewers .... 2<br>Market .... 3<br>Dairy Cooperative .... 4<br>Other Cooperative .... 5<br>Milk Purchaser (Not Coop) .... 6<br>Local Agrovet Supplier .... 7<br>Other (Specify) 96
K1a_23	In the past one year, did you feed mixed concentrates to your dairy cows?<br><br>Mixed concentrates are special nutrient-rich feeds that are fed to dairy cows to increase milk production. They are usually a mixture of grains and cereals, and can include other nutrient-dense ingredients like brans, pomaces, or oil-seed cake. They are usually purchased.		Yes .... 1<br>No...2>>K1a_26
K1a_24	How often do you feed mixed concentrates to your dairy cows?		Daily... 1<br>Weekly .... 2<br>Monthly... 3<br>Whenever available.................................... 4<br>Other (specify) 6
K1a_25	Where did you get the mixed concentrates fed to your dairy cows?<br><br>[SELECT ALL THAT APPLY]		Own production / prepared cereal foods ..... 1<br>Market .... 2<br>Dairy Cooperative .... 3<br>Other Cooperative .... 4<br>Local Agrovet Supplier .... 5<br>Milk Purchaser (Not Coop) .... 6<br>Other (Specify) 96

Household Number: | | | | |

Serial	Question	Response	Response Code
K1a_26	In the past one year, did you feed vitamins or minerals to your dairy cows, for example, salt, vitamins, or other kinds of mineral supplements?	[ ]	Yes .... 1<br>No........ 2>>K1a_27
K1a_26a	How often do you feed vitamins or minerals to your dairy cows?	[ ]	Daily........ 1<br>Weekly .... 2<br>Monthly........ 3<br>Whenever available........ 4<br>Other (specify) 6
K1a_27	What do you do with your cows' manure?	[ ]	Yes, I collect it .... 1<br>Nothing; leave it where it falls .. 2>> K1a_28
K1a_27a	Do you dry the collected manure?	[ ]	Yes .... 1<br>No........ 2
K1a_27b	Where do you put the cows' manure after you collect it?	[ ]	Put in heap in uncovered area .... 1<br>Put in heap in covered area .... 2<br>Put in a pit/lagoon .... 3<br>Put into a tank .... 4<br>Put into a biogas-producing digester........ 5<br>Other (specify) 96
K1a_27c	What do you do with this collected manure?<br><br>[SELECT ALL THAT APPLY]	[ ]<br>[ ]<br>[ ]	Nothing .... 1<br>Household uses it for fuel .... 2<br>Household puts on field as fertilizer .... 3<br>Give it to friends/neighbors........ 4<br>Sell it to friends/neighbors .... 5<br>Sell it at market .... 6<br>Other (specify) 96
K1a_28	In the past one year, did you obtain any health services from a trained provider for your dairy cows?<br><br>Examples of health services include things like vaccinations, treatments for sick animals, and assistance with delivery of calves	[ ]	Yes .... 1<br>No........ 2>>K1a_30
K1a_29	From whom did you obtain these health services for your dairy cows?<br><br>[SELECT ALL THAT APPLY]	[ ]<br>[ ]<br>[ ]	Self .... 1<br>Community Animal Health Worker .... 2<br>Government Extension Services .... 3<br>Public Veterinarian .... 4<br>Private Veterinarian .... 5<br>Private Veterinary Pharmacy .... 6<br>Other (Specify) 96

Household Number: [ ][ ][ ][ ]

Serial	Question	Response	Response Code
K1a_30	In the past one year, have you given any medicines to your dairy cows, for example antibiotics, wormers, or external parasite treatments?	[ ]	Yes .... 1<br>No........ 2>>K1a_32
K1a_31	From where did you obtain these medicines you gave to your dairy cows?<br><br>[SELECT ALL THAT APPLY]	[ ]<br>[ ]<br>[ ]	Trader .... 1<br>Dairy Cooperative .... 2<br>Milk Purchaser (Not Coop) .... 3<br>Community Animal Health Worker .... 4<br>Local Agrovet Supplier .... 5<br>Private Veterinary Pharmacy .... 6<br>Government .... 7<br>Other (Specify) 96
K1a_32	How long does it take to obtain emergency animal health services when you need them?	Hour [ ][ ] Minute [ ][ ]	None Available 99
K1a_33	In the past one year, did you vaccinate none, some, or all of your cattle?	[ ]	No cattle vaccinated........ 1>> K1a_34<br>Some cattle vaccinated........ 2<br>All cattle vaccinated........ 3
K1a_33a	What vaccinations did you give to your dairy cows and calves in the past one year?<br><br>[SELECT ALL THAT APPLY]	[ ]<br>[ ]<br>[ ]	FMD (Foot and Mouth Disease) .... 1<br>HS .... 2<br>BQ .... 3<br>Anthrax .... 4<br>CBPP (Contagious Bovine Pleuropneumonia) 5<br>RVF (Rift Valley Fever) .... 6<br>Other (Specify)........ 96
K1a_33b	When decisions are made regarding what vaccinations to give your dairy cows, who is it that normally takes the decision?<br><br>[SELECT ALL THAT APPLY]	[ ]<br>[ ]	Self........ 1<br>Partner/spouse .... 2<br>Other household member .... 3<br>Other non-household member........ 4
K1a_34	Have you ever heard of mastitis?<br><br>Dairy cows sometimes experience a condition called mastitis, an inflammation of the cow's udder that reduces milk production and quality.	[ ]	Yes .... 1<br>No........ 2>>K1a_35
K1a_34a	Do you do anything to prevent mastitis in your dairy cows?	[ ]	Yes .... 1<br>No........ 2>>K1a_35

Serial	Question	Response	Response Code
K1a_34b	What do you do to prevent mastitis in your dairy cows?<br><br>[SELECT ALL THAT APPLY]		Wash Udder .... 1<br>Teat Dip .... 2<br>Somatic Cell Counts .... 3<br>Other (Specify) 96
K1a_35	Do you usually process some of your cows' milk into other products like cheese or yoghurt?		Yes .... 1<br>No.. 2
K1a_36	Do you sell your cows' milk?		Yes .... 1<br>No.. 2
K1a_37	Yesterday morning, how much milk in total did your dairy cows produce?		Write quantity in liters<br><br>The dairy cow did not give milk 00
K1a_38	Yesterday evening, how much milk in total did your dairy cows produce?		Write quantity in liters<br><br>The dairy cow did not give milk 00
K1a_39	ADD QUANTITIES IN ITEMS K1a_37 AND K1a_38.<br><br>Your dairy cows produced [QUANTITY] [UNITS] of milk yesterday. How many [UNITS] of that milk did you sell?		Write quantity in liters<br><br>The dairy cow did not give milk 00
K1a_40	Where do you usually sell your milk?<br><br>[SELECT ALL THAT APPLY]		Sell to Friends/Neighbors .... 1<br>Sell It at Market Myself .... 2<br>Sell to A School .... 3<br>Sell to A Milk Marketing Cooperative .... 4<br>Sell to Aggregator/Off-Taker .... 5<br>Do not Sell .... 6> K1a_46<br>Other (Specify) 96
K1a_41	At what time of the day do you usually sell milk?		Only morning milk.................................. 1<br>Only evening milk.................................... 2<br>Both morning and evening milk................. 3
K1a_42	*CHECK K1a_40: DOES FARMER SELL MILK TO A MILK MARKETING COOP (RESPONSE 4)?*		Yes .... 1<br>No.. 2>>K1a_45
K1a_43	What services does your milk marketing cooperative provide you?<br><br>[SELECT ALL THAT APPLY]		Amount Of Milk Sold .... 1<br>Fat Content Of Milk Sold .... 2<br>Acidity Of Milk Sold .... 3<br>Extension Services .... 4<br>Animal Health Services .... 5<br>Loans 6

Household Number: | | | | |

Serial	Question	Response	Response Code
			Other (Specify) 96
K1a_44	How do you receive payment for your milk from your cooperative?<br><br>[SELECT ALL THAT APPLY]		Cash ..... 1<br>Store Credit ..... 2<br>MPESA/Mobile Money ..... 3<br>Direct Deposit To Bank Account ..... 4<br>Other (Specify) 96
K1a_45	Do you keep daily written records on your dairy cows, for example how much each animal eats, what they eat, how much milk is produced, or when an animal is sold, born, or bought?		Yes ..... 1<br>No..... 2
K1a_46	How do you decide when to sell one of your dairy cows?<br><br>[SELECT ALL THAT APPLY]		When There Is A Need for Cash ..... 1<br>When Her Dam Is Poor-Performing ..... 2<br>When her milk production falls below a certain level 3<br>When She Doesn't Re-Breed ..... 4<br>When She Gets Too Old ..... 5<br>When Enough Heifers Expected to Produce More Milk Enter Herd ..... 6<br>When Herd Size Is Not Manageable ..... 7<br>When There Is Not Enough Food to Feed All of The Animals ..... 8<br>Other (Specify)..... 96
K1a_47	When decisions are made regarding selling a dairy cow, who is it that normally takes the decision?<br><br>[SELECT ALL THAT APPLY]		Self ..... 1<br>Partner/spouse ..... 2<br>Other household member ..... 3<br>Other non-household member..... 4
K1a_48	In the past year, how many of your dairy cows did you sell?		Record the number of females<br>Did not sell any 00
K1a_49	In the past year, how many male animals (bull/bullock/buffalo) did you sell?		Record the number of males<br>Did not sell any 00
K1a_50	What information source do you rely on the most to help you raise your livestock well?<br><br>**Module End Time:** Hour __ __ Minute __ __		Friend/neighbor..... 1<br>Community animal health worker..... 2<br>Local agrovet supplier..... 3<br>Private veterinary pharmacy ..... 4<br>Agricultural extension worker..... 5<br>School ..... 6<br>Radio program ..... 7<br>Television..... 8<br>Mobile phone messaging ..... 9<br>Internet ..... 10<br>Other (specify) 96

Household Number: | | | | |

Module K2: Livestock and Poultry Products (Male)
For last 1 year, December 1 2017 to November 30 2018.

Module start time: | Hour | | | Minu | | | Respondent ID: | | Consent : | | Yes ...1 No.....2

Animal/Poultry Product		Unit name	How much did you produce in the last 12 months?		How much did you consume in the last 12 months?		How much did your household give to others?	How much did your household use for reproduction?	How much was spoilt?	How much was stored/ used as stock?	How much did your household sell in the last 12 months? (if no sales >>next animal product)	Where did you sell your production?	Total value of selling	Who controls the money from the sale of livestock products?<br><br>Report 3 primary owners.<br>If HH member, write MID.<br>If outside household, use code 1.		
			Quantity	How much would you receive if you sold this product? (per unit)	Quantity	How much would you receive if you sold this product? (per unit)										
Name	Code	Unit	Qty	Taka	Qty	Taka	Qty	Qty	Qty	Qty	Qty	Code 2	Tk	MID/Code 1		
**Product Name**	**BP Code**	**K2_01**	**K2_02**	**K2_03**	**K2_04**	**K2_05**	**K2_06**	**K2_07**	**K2_08**	**K2_09**	**K2_10**	**K2_11**	**K2_12**	**K2_13a**	**K2_13b**	**K2_13c**
Milk*	1	Liter														
Eggs (chicken)*	21	Number														
Eggs (duck)*	22	Number														
Manure*	3	Kg														

Note:* Taka per unit sold.

**Code 1:**	**Code 2: Where sold**
All members jointly ....71<br>Male outside household ....72<br>Female outside household.......73<br>Govt / Khas land/other institutions 74<br>Not applicable........................ 98	Farm gate (home)............................1<br>Village market (within own village)2<br>Village market (outside own village) 3<br>Town market ....4<br>Neither sold nor rented....................9<br>Other (specify) 5

Household Number: | | | | |

Module K3: Expenditure for Livestock and Poultry Production (Male)

For last 1 year, December 1 2017 to November 30 2018.

	Animal/Poultry	Fodder /feed bought	Medicine/ treatment cost	Labor use in person days						Other expenses if purchased
				Family*		Hired*		Hired*		
				Male	Female	Male	Male	Female	Female	
Animal/Poultry	Code	(Tk)	(Tk)	(hours)	(hours)	(hours)	Total cost	(hours)	Total cost	(Tk)
**Name**	**K3_01**	**K3_02**	**K3_03**	**K3_04**	**K3_05**	**K3_06**	**K3_07**	**K3_08**	**K3_09**	**K3_10**
Cow/Bullock/Buffalo	1									
Goat/Sheep/Pig	2									
Chicken/Duck/Birds	3									
Others (please specify)	4									

Note: * Include adult and child labor.

Round hours to the whole number. Report “0” only if no labor has been used. For e.g. if plot is weeded for 15 min report 1 hour and not “0”.

Sl no	Question	Response	Response Code
**K3_11**	Where did you hire most of the laborers from?		From farm/home.................................. 1<br>Village market (within own village)..... 2<br>Village market (outside own village) ... 3<br>City market.. 4<br>Others (please specify) .... 5<br>Neither purchased nor rented................9

Module End Time: | Hour | | | Minute | | |

Household Number: | | | |

Module L: Fisheries (Male)

Module Start Time: Hour | | | Minute | | | **Respondant ID:** | | Respondant's Consent: | | Yes ......1 No 2

Module L1: Fish/Shrimp (Report fish cultivation in paddy field or other crop fields as well) (Male)

Plot/pond/water bodies utilization in last 1 year: December 1 2017 to November 30 2018.

Serial	Plot ID	Area (write '0' if fishing is done from open water body)	Name of Fish &/ Crop*	Fish and Crop Code* Write all types of fishes/crops harvested in last 1 year							Are carps and small fishes cultivated together in this	Is any crop cultivated around the bank of this	If yes, did you get any NGO assistance?	NGO name	Labor use in person days (Labor hour/ day)			Note: L1_10 -13 are applicable if ponds are used to cultivate fish only and fish & crop together. If ponds are used to cultivate crops skip L1_10-13.				How many times harvested in last 1 year	Total qty of collection/ harvest	Loss of output	
											Yes…1 No…2	Yes…1 No…2	Yes…1 No…2	If answer to L1_02c is yes, write name of the NGO	Family**	Hired**	Daily wage for hired labor	Fingerlings	Feed	Other	Total Cost			Qty	Reason
	Plot ID	Decimal	(Max. 6 types of fish and 1 crop)	Fish1	Fish2	Fish3	Fish4	Fish5	Fish6	Crop1	Code ↑	Code ↑	Code ↑	NGO	(hrs)	(hrs)	(Tk/ day)	Tk	Tk	Tk	Tk	No.	kg	Kg	Code 2
**L1_sl**	**Pond ID**	**L1_01**	**Fish and crop name**	**L1_02_01**	**L1_02_02**	**L1_02_03**	**L1_02_04**	**L1_02_05**	**L1_02_06**	**L1_02_07**	**L1_02a**	**L1_02b**	**L1_02c**	**L1_02d**	**L1_03**	**L1_04**	**L1_05**	**L1_06**	**L1_07**	**L1_08**	**L1_09**	**L1_10**	**L1_11**	**L1_12**	**L1_13**

Note:* For Fish refer the fish code (Code 1) that appears at the end of Module L2. For Crop refer to crop code (code 1) used for module H.

Household Number: | | | | |

* For each plot, write the name of all fish harvested, separated by commas. If fish is farmed in paddy field, write the name of 1 crop

*** Include adult and child labor. Round hours to the whole number. Report "0" only if no labor has been used. For e.g. if plot is weeded for 15 min report 1 hour and not "0".

Fill **separate** row for each pond. The pond reported in the Module L must be reported in the Module G. Plot ID of pond in Module G will be the Plot ID for pond in the Module L. If fish were collected from the open water, write 999 as Plot ID.

**Sl no**	Question	Response	Response Code
**L1_14**	Where did you hire most of the laborers from?		From farm/home .... 1<br>Village market (within own village)..... 2<br>Village market (outside own village) ... 3<br>City market .... 4<br>Neither purchased nor rented................ 9<br>Others (please specify) 5

Module L2: Fish/Shrimp Pond Production and Inputs (Male)

Plot/pond/water bodies utilization in last 1 year: December 1 2017 to November 30 2018.

Name of Fish	Fish Code	Unit of measure<br><br>Kg ....1<br>Nos…2	Total Production under own/share of plot/pond /any other water bodies in operation	Harvest received from the shared out (leased/contract) pond	Share of harvest given to owner (if shared pond)	Quantity consumed	Paid to the laborers	Quantity for dry fish	Given to Others	Qty sold<br><br>If "0" >>next case	Where sold?	Total value of selling
Name of Fish	[Code 1]	Code ↑	Qty	Qty	Qty	Qty	Qty	Qty	Qty	Qty	[Code 3]	Tk
**Name**	**L2_01**	**L2_02**	**L2_03**	**L2_04**	**L2_05**	**L2_06**	**L2_07**	**L2_08**	**L2_09**	**L2_10**	**L2_11**	**L2_12**

Household Number: | | | | |

Code 1: Fish Codes			Code 2: Reason of loss code	Code 3: Where sold
Silver carp .... 1<br>Grass carp......................... 2<br>Mirror carp .... 3<br>Common carp................... 4<br>Karfu .... 5<br>Rui................................... 6<br>Katla................................ 7<br>Mrigel.............................. 8<br>Kalibaus 9	Telapia/Nailotica...............10<br>Pona ....11<br>Koi ....12<br>Magur ....13<br>Shingi...............................14<br>Khalse ....15<br>Shol/Gajar/Taki ....16<br>Puti/Swarputi 17	Prawn (Golda Chingri)......18<br>Shrimp (Bagda Chingri)....19<br>Tengra/Baim ....20<br>Mola/Dhela/Kachki/Chapila 21<br>Ilish/hilsha.........................22<br>Other Large fish ....23<br>Other Small fish ....24<br>Sea fish.............................25<br>Pangash 26	Flood................................1<br>Water Toxicity..................2<br>Theft ....3<br>Due to cold ....4<br>Other (Specify) 5	Farm gate (home).............1<br>Village market (within own village) ....2<br>Village market (outside of own village) ....3<br>Town market.....................4<br>Neither sold nor rented .....5<br>Other (specify).................9

Module End Time: Hour | | | Minute | | |

Module ITMP2B: Improved Hatchery Management Technology (Male)

Module start time: Hour | | | Minu | | | **Respondent ID:** | | Consent : | | Yes ...1 No.....2

Question number	Questions	Response		Response codes
		Tilapia	Carp	
**ITMP2B_01**	Does your hh have any hatchery/ Do you produce fish fry?			Yes=1<br>No=2>> Next Module<br><br>If the respondent does not produce either Tilapia or Carp, move to the next module.
**ITMP2B_02**	What is the stocking density?			(Piece/decimal)<br>Write the number of fingerling per decimal
**ITMP2B_03**	What is the depth of water at brood ponds?			Foot
**ITMP2B_04**	What water PH level do you need to maintain?			PH Level
**ITMP2B_05**	How many times do you exchange water?			Once every month…………………..1<br>Once every two months……………..2<br>When required………………………3<br>Do not exchange water……………...4

Household Number: | | | | |

Question number	Questions	Response		Response codes
		Tilapia	Carp	
**ITMP2B_06**	What percentage of water do you exchange?			Percentage
**ITMP2B_07**	What percentage of protein is used in feed?	☐	☐	Percentage<br>Don't Know…………………..999
**ITMP2B_08**	What is the feed application rate?	☐	☐	1-1.5% of body weight………………1<br>1.5-2% of body weight………………2<br>2-3% of body weight (1-1.5% immediate before breeding)……………………………..3<br>Others………………………………...4<br>Don't know…………………………...5
**ITMP2B_09**	During the production of fish fry, do you use cow dung in your pond?	☐	☐	Use it……………………1<br>Do not use it…………..2
**ITMP2B_10**	If yes, what amount of it do you use every week?	☐	☐	(Gram/Decimal)<br>*Write the amount used every week in grams per decimal
**ITMP2B_11**	During the production of fish fry, do you use urea in your pond?	☐	☐	Use it……………………1<br>Do not use it…………..2
**ITMP2B_12**	If yes, what amount of it do you use every week?	☐	☐	(Gram/Decimal)<br>*Write the amount used every week in grams per decimal
**ITMP2B_13**	During the production of fish fry, do you use TSP in your pond?	☐	☐	Use it……………………1<br>Do not use it…………..2
**ITMP2B_14**	If yes, what amount of it do you use every week?	☐	☐	(Gram/Decimal)<br>*Write the amount used every week in grams per decimal
**ITMP2B_15**	How often do you observe netting and health conditions?	☐	☐	1-2 times each month……………………1<br>2-3 times each month……………………2<br>Don't observe…………………………….3
**ITMP2B_16**	What ratio do you maintain for Male: Female brood?	☐	☐	1:2……………………………….1<br>1:3……………………………….2<br>2:3……………………………….3<br>Others (specify)………………..4<br>Don't know……………………..5

Household Number: | | | |

Question number	Questions	Response		Response codes
		Tilapia	Carp	
**ITMP2B_17**	Does it require to take care of the aeration facilities?			Yes……………………………1<br>No……………………………..2<br>Don't know……………………3

Module End Time: | Hour | | | Minute | | |

Household Number: [ ] [ ] [ ] [ ]

Module ITMP2C: Nursery Technologies (Male)

Module start time: Hour [ ] [ ] Minu [ ] [ ]   Respondent ID: [ ]   Consent : [ ]   Yes ...1 No.....2

Question number	Questions	Response			Response Code
	Key management features	Carp	Shrimp	Prawn	
**ITMP2C_01**	Do you have a fish nursery?				Yes..1<br>No ....2>>Next Module<br><br>If the household does not have a nursery of either carp. shrimp and prawnthen move to the next module.
**ITMP2C_02**	How do you build your pond dike?				Well prepared……………………………...1<br>Not well prepared…………………………2<br>Not applicable (Prepared from before) .... 3<br>Others (Specify)... 4
**ITMP2C_03**	How does the water entrance need to be?				Deep water... 1<br>Canal water .... 2<br>Not applicable (There is water all year round) 3<br>Others (Specify)... 4
**ITMP2C_04**	Do you need to completely or partially control the aquatic weed?				Yes... 1<br>No 2
**ITMP2C_05**	Do you remove predators and non-culture fish from the farming area?				Completely removes .... 1<br>Partially removes .... 2<br>Do not remove 3
**ITMP2C_06**	What amount of lime do you use in your pond per decimal?				(kilogram/decimal)
**ITMP2C_07**	Do you put bleach in the nursery?				Yes... 1<br>No 2
**ITMP2C_08**	Do you use fencing with net?				Yes, Always use it .... 1<br>Yes, use it sometimes .... 2<br>No, never use it.. 3
**ITMP2C_09**	How do youcontrol harmful insect?				Yes, used Sumethion .... 1<br>Yes, used kerosene/diesel .... 2<br>No, do not use anything to control harmful insects3
**ITMP2C_10**	Do you use fertilizer before stocking?				Yes, use it .... 1<br>Yes, use it but not in the right quantity....... 2<br>No, never use it……………………………………..3

Household Number: | | | |

Question number	Questions	Response			Response Code
	Key management features	Carp	Shrimp	Prawn	
**ITMP2c_11**	Do you observe natural feeding in the pond?				Yes, I observe .... 1<br>Yes, but not in the right manner .... 2<br>No, I do not observe.................................... 3
**ITMP2c_12**	Do you carry out toxicity test?				Yes, carry out toxicity test .... 1<br>No, do not carry out toxicity test .... 2<br>No, can predict ifthe pond has become toxic (from the way fishes behave to toxicity/fishesfloat on the surface) 3
**ITMP2c_13**	Do you properly acclimatize the pond?				Yes... 1<br>No 2
**ITMP2c_14**	What is the stocking density?	Gram/Decimal<br><br>Piece/Decimal	Gram/Decimal<br><br>Piece/Decimal	Gram/Decimal<br><br>Piece/Decimal	Gram/Decimal<br><br>Piece/Decimal
**ITMP2c_15**	Do you test the water quality? (Different parameters)				Yes, the quality of water is tested .... 1<br>No, the quality of water is not tested 2
**ITMP2c_16**	Do you observe survivals of fishes?				Yes.. 1<br>No 2
**ITMP2c_17**	Do you maintain sampling as a routine work?				Yes.. 1<br>No 2

Household Number: | | | | |

Question number	Questions	Response			Response Code
	Key management features	Carp	Shrimp	Prawn	
**ITMP2c_18**	What is the nursery water treated with?				Treated with bleaching powder…………………1<br>Treated with lime…………………2<br>Used TINSON .... 3<br>Used Zeolite.. 4<br>Others (Specify).. 5<br>Not apllicable.. 6
**ITMP2c_19**	How is the reservoir water treated?				Water is disinfected with lime .... 1<br>Water is disinfected with bleaching powder 2<br>Others (Specify).. 3<br>Not applicable... 4
**ITMP2c_20**	Does it require in-let/Out-let system in the nursery?				Yes…………………………………...1<br>No, there was no need of one………..2<br>No, inlet/outlet………………….....….3
**ITMP2c_21**	Is WSSV negative screened PLs/spawn stocking?				Yes, screened PLs (MTT and CST gher) before stocking……………..1<br>No, screened PLs (MTT and CST gher) before stocking……………….2<br>Not applicable………..3
**ITMP2c_22**	How much supplementary feed is used?				2-7 percent of body weight……………1<br>10 percent of body weight……………2<br>Others (Specify)…..3
**ITMP2c_23**	What is the culture period?				15-20 days……….1<br>15-30 days……….2<br>30-60 days……….3<br>45-60 days……….4<br>More than 60 days………………5
**ITMP2c_24**	Is horra pulling required?				Applies regularly……………………1<br>When need…………………………..2

Module End Time: | Hour | | | Minute | | |

Household Number: | | | |

Module ITMP2A: TILAPIA/ Silver carp/Katla and Other Varieties of Fish Farming Technologies (Male)

If the household has more than one pond, then take the answers concerning majority of the ponds

Module start time: Hour | | | Minu | | | Respondent ID: | | Consent : | | Yes ...1 No.....2

Question number	Questions	Response			Response Code
	Key management features	Tilapia	Carp/Katla	Other Varieties of Fish	
**ITMP2A_01**	Are you involved in fish farming?				Yes .... 1<br>No ....2>> Next Module
**ITMP2A_02**	If yes, what is the main reason of fish farming?				For cunsumption purpose........................... 1<br>To sell .... 2<br>Both consume and sell 3
**ITMP2A_03**	How do you build your pond dike?				Moderately raised……………………….1<br>Dikes are cleaned and compacted to prevent entry of disease contaminated outside water………2<br>Don't know……………………………….3<br>Others……………………………………4
**ITMP2A_04**	Do you use fencing with net in your pond?				No Barrier……………………………...1<br>Barriers are established to prevent entering the pond……………………………………2<br>Don't know……………………………….3
**ITMP2A_05**	Do you remove predators and non-culture fish from the farming area?				Yes, completely remove…………………1<br>Yes, partially removes…………………..2<br>No, do not remove……………………..3
**ITMP2A_06**	Do you use lime in your pond?				Yes ....1<br>No .... 2>>**ITMP2A_08**
**ITMP2A_07**	If yes, how much lime do you use per decimal?				Kg/decimal
**ITMP2A_08**	Do you use fertilizer in your pond?				Very less ....1<br>Proper amount…………… .....2<br>Do not use .... …3>>**ITMP2A_13**
**ITMP2A_09**	Do you use urea?				Use it.....................................1<br>Do not use it....................... 2>>**ITMP2A_11**
**ITMP2A_10**	If yes, how much urea do you use per decimal?				Gram/decimal

Household Number: | | | | |

Question number	Questions	Response			Response Code
	Key management features	Tilapia	Carp/Katla	Other Varieties of Fish	
**ITMP2A_11**	Do you use TSP?				Use it ....1<br>Do not use it....................... 2>>**ITMP2A_13**
**ITMP2A_12**	If yes, how much TSP do you use per decimal?				Gram/decimal
**ITMP2A_13**	Do you use cow dung?				Yes ....1<br>No .... 2>>**ITMP2A_15**
**ITMP2A_14**	If yes, how much cow dung do you use per decimal?	Kg/decimal	Kg/decimal	Kg/decimal	Kg/decimal
**ITMP2A_15**	Do you carry out toxicity test?				Yes, recently…………………………1<br>Yes, but long time ago………………..2<br>No, never carried out the test…………3
**ITMP2A_16**	What is the size of the fingerlings in your pond?<br><br>(Considering most of your ponds)	Length in inches	Length in inches	Length in inches	Length in inches
**ITMP2A_17**	What is the stocking density of the fingerlings?				Piece/decimal<br>(Write the number)
**ITMP2A_18**	Do you take measures to purify fingerlings?				Yes .... 1<br>No 2
**ITMP2A_19**	Do you properly acclimatize the pond?				Not properly…………………………1<br>Absolutely maintaining………………2
**ITMP2A_20**	Do you fertilize the pond post stocking?				Yes, sometimes .... 1<br>Yes, doing it continuously .... 2<br>No 3
**ITMP2A_21**	Do you provide supplementary feed to the fish?				Provide properly and regularly .... 1<br>Occasionally... 2<br>Do not provide properly............................. 3<br>No, do not provide supplementary feed 4

Household Number: | | | | |

Question number	Questions	Response			Response Code
	Key management features	**Tilapia**	**Carp/Katla**	**Other Varieties of Fish**	
**ITMP2A_22**	Do you maintain sampling as a routine work?				Yes .... 1<br>No 2
**ITMP2A_23**	How many times do you harvest in a year?				Wirte the number of times<br>If not once, write "0"
**ITMP2A_24**	How many times do you completely harvest and empty out the pond?				Several times in a year .... 1<br>Once every year .... 2<br>Once in every two years............................. 3<br>Once in every three years........................... 4<br>Once in every four years .... 5<br>Never completely empty out the pond 6
**ITMP2A_25**	Do you have in-let/Out-let in your farming area?				Yes, currently have inlets/outlets .... 1<br>No, build it when necessary .... 2<br>No, no inlets/outlets 3
**ITMP2A_26**	How often do you exchange water in your pond?				Very few times... 1<br>Do it when needed .... 2<br>Do not exchange water in the pond............. 3
**ITMP2A_27**	Do you keep records?				Yes, accurately... 1<br>Yes, but not accurately............................... 2<br>No, don't keep records 3
**ITMP2A_28**	What is the marketing size of the fish?	Weight<br>Length	Weight<br>Length	Weight<br>Length	Weight (Gram/piece)<br>Length (Gram/Piece)
**ITMP2A_29**	How much do you produce per cycle (3-4 months)?				Kg

Household Number: | | | | |

Question number	Questions	Response			Response Code
	Key management features	**Tilapia**	**Carp/Katla**	**Other Varieties of Fish**	
**ITMP2A_30**	How much fish do you produce per decimal?	Kg/decimal	Kg/decimal	Kg/decimal	(Kg/decimal)

Module End Time: | Hour | | | Minute | | |

Module L2a: Agricultural Technologies - Fishpond Aquaculture (Male)

Recall period: For last 1 year, December 1 2017 to November 30 2018

Module start time: | Hour | | | Minu | | | **Respondent ID:** | | Consent : | | Yes ...1 No.....2

Question number	Questions	Response	Response Code
**L2a_01**	Were you or any of your family member involved in fish farming in the period December 1, 2017 to November 30, 2018)		Yes ....1<br>No...2>>Next module
**L2a_01a**	How many fish fries/ fingerlings in total have you stocked in the recall period??<br><br>Note: If the farmer was involved in **fish fry** farming, then record the **weight in gram** of the fish fries. If the farmer was involved in **fingerling** farming, then record the **number of fingerlings stocked** of the fish fries.	Gram Number	Total weight/number of fish fry/finegrlings stocked
**L2a_02a**	What was the main source of fish you stocked in the recall period?		Raised own, received from friends/relatives (Not purchased) .... 1>>**L2a**_03<br>Purchased from friends/relatives........................ 2>>**L2a**_03<br>Purchased from the market................................... 3<br>Purchased from local vendor................................ 4<br>Purchased from local hatchery .... 5<br>Purchased from non-local hatchery............................6<br>Received it as aid .... 7>>**L2a**_03

Household Number: | | | |

			Others (Specify) .... 96>>**L2a**_03
**L2a_02c**	Was the hatchery where you purchased your fish fry and fingerling a registered or certified hatchery?		Yes ....1<br>No..2<br>Don't know 8
**L2a_03**	Where do you raise your fish? In a man-made earthen pond, a raceway, a natural pond, a stream, or somewhere else?<br><br>[Multiple response allowed]		Man-made earthen pond............................. 1<br>Raceway.. 2<br>Natural pond/lake...................................... 3<br>Stream .... 4<br>Others (Specify) 96
**L2a_04**	In the last one year, did you give your fish supplemental feed?		Yes .... 1<br>No.. 2> **L2a**_06
**L2a_05**	Where did you get most of the supplemental feed you gave to your fish - did you make it yourself or did you buy it?		Mostly made own supplementary fish feed. 1<br>Mostly purchaed supplementary fish feed .. 2<br>Half made and half purchased..................... 3
**L2a_06**	In the last one year, did you use hormones to raise your fish?		Yes .... 1<br>No.. 2> **L2a**_08
**L2a_07**	At what stage of growth did you apply the hormone to the fish?<br><br>[Multiple response allowed]		Initial growth phase (First 28 days) .... 1<br>Development phase.................................... 2<br>Others (Specify) 96
**L2a_08**	Have you been formally trained in the use of hormones for fish farming?		Yes .... 1<br>No.. 2
**L2a_09**	What kind of fish did you raise in the last one year?<br><br>[Multiple response allowed]		**Use the fish code from L1**<br><br>Don't know 96
**L2a_10**	Did you observe any disease among your fish in the last one year?		Yes .... 1<br>No.. 2
**L2a_10a**	Did you do anything to control disease among your fish in the last one year?		Yes .... 1<br>No.. 2> **L2a**_11
**L2a_10b**	What did you do to control disease among your fish?<br>[Multiple response allowed]		Nothing .... 1<br>Apply salt... 2<br>Apply formalin.. 3<br>Apply malachite green .... 4<br>Apply methayl blue... 5<br>Antibiotics/Antibioic treated feed .... 6<br>Applying lime 7

			Applying Potash...8<br>Others (Specify)………………….. 96
**L2a_11**	Did you observe any parasite cases among your fish in the last one year?		Yes ....1<br>No..2
**L2a_11a**	Did you do anything to control parasites among your fish in the last one year?		Yes ....1<br>No.. 2> **L2a_**12
**L2a_11b**	What did you do to control parasites among your fish?<br><br>[Multiple response allowed]		Nothing ....1<br>Apply salt ....2<br>Apply formalin..3<br>Apply malachite green ....4<br>Apply methayl blue.......................................5<br>Antibiotics/Antibioic treated feed ....6<br>Applying lime ....7<br>Applying Potash..8<br>Others (Specify)………………….. 96
**L2a_12**	Did you monitor the water quality in your pond in the last one year?		Yes ....1<br>No.. 2> **L2a_13**
**L2a_12a**	How did you monitor the water quality in your pond in the last one year?<br><br>[Multiple response allowed]		Used hand to assess turbidity .... 1<br>Used hand to assess turbidity...2<br>PH level tested .... 3<br>Observed fish for piping behavior...................................... 4<br>Others (Specify) 96

Household Number: | | | |

**L2a_13**	Did you do anything to maintain good water quality in your pond in the last one year?	☐	Yes .... …1<br>No………………………………………………..2> **L2a_**14
**L2a_13a**	What did you do to maintain good water quality in your pond in the last one year?<br><br>[Multiple response allowed]	☐<br>☐<br>☐<br>☐	Screened the water coming into the pond .... 1<br>Cut the grass around the pond .... 2<br>Drained the pond to clean it .... 3<br>Added ash to stabilize water PH .... 4<br>Added dissolved oxygen .... 5<br>Added lime .... 6<br>Added fertilizer .... 7<br>Others (Specify) 96
**L2a_14**	In the last one year, how many times have you drained the water from your fish pond and allowed the bottom of the pond to dry?	☐	Number<br>None..00
**L2a_15**	In the last one year, have you used any techniques to improve your production of fish?	☐	Yes .... 1<br>No... 2> **L2a_**17
**L2a_16**	What kind of technique did you use in the last one year to improve your production of fish? Did you practice:	**Item / Response**<br>A) Sex separation ☐<br>B) Age separation ☐<br>C) Variation of feed ☐<br>D) An improved stocking method ☐<br>E) Others (Specify) ☐	Yes .... 1<br>No... 2
**L2a_17**	Did you add animal manure to your fish ponds in the last one year?	☐	Yes .... 1<br>No... 2> **L2a_**18a

**L2a_17a**	Where do you usually get the manure you add to your fish ponds?		Produced by own animals .... 1<br>Given by friends/family/neighbor farm....... 2<br>Purchased from market/ trader 3
**L2a_18a**	Do you usually harvest all of your fish at one time, or do you usually do partial harvests?		Harvest all fish at once .... 1<br>Partial harvest... 2
**L2a_18b**	What method do you usually use to harvest your fish?		Cast net ....1>>L2a_19a<br>Seine net...2>>L2a_19a<br>Pull cages up....................................3<br>Others (Specify) ....96>>L2a_19a
**L2a_18c**	How many cages do you have?		Number of cage<br>Don't know 998
**L2a_18d**	What is the height of your cages?		Height of the cage<br>Write it in meters<br>Don't know 998
**L2a_18e**	What is the width of your cages?		Width of the cage<br>Write it in meters<br>Don't know 998
**L2a_18f**	What is the depth of your cages?		Depth of the cage<br>Write it in meters<br>Don't know 998
**L2a_19a**	How many fish have you harvested in the last one month?		Number of fish<br>Did not harvest.......................................0>>L2a_19c<br>Don't know 9998
**L2a_19b**	What was the total weight (in kg) of the fish you harvested in the last one month?		Weight of fish<br>Kilogram<br>Don't know 9998
**L2a_19c**	How many fish have you harvested in the last one year?		Number of fish<br>Did not harvest.. 0>>L2a_20<br>Don't know 9998

Household Number: | | | |

**L2a_19d**	What was the total weight of the fish you harvested in the last one year?			Weight of fish<br>Kilogram<br>Don't know 9998
**L2a_20**	Why did you produce fish?			Raised for food only.................................. 1<br>Raised for market only............................... 2<br>Raised for both food and market................ 3
**L2a_21**	After you harvest the fish that you use to provide food for your household, do you usually:	a) Leave the fish whole round		Yes .... 1<br>No.. 2
		b) Gut the fish		
		c) Salt		
		d) Smoke		
		e) Dry		
		f) Pickle the fish		
		g) Others (Specify)		
**L2a_23**	After you harvest the fish that you sell or trade in the market, do you usually:	a) Leave the fish whole round		Yes .... 1<br>No.. 2
		b) Gut the fish		
		c) Salt		
		d) Smoke		
		e) Dry		
		f) Pickle the fish		
		g) Others (Specify)		
**L2a_23a**	After you process your fish, what do you do with the fish guts?<br><br>[Multiple response allowed]			Nothing/discard.. 1<br>Use as compost .... 2<br>Use as animal feed .... 3<br>Others (Specify) 96
**L2a_23b**	After you process your fish, what do you do with the fish skins or scales?			Nothing/discard.. 1<br>Use as compost .... 2<br>Use as animal feed .... 3<br>Sell them .... 4<br>Others (Specify) 96

Household Number: | | | | |

**L2a_24**	Do you keep regular written records on your fish, for example how much feed the fish are given, what kind of feed the fish are given, the number of fish stocked, or the number of fish harvested?	[ ]	Yes .... 1<br>No........ 2
**L2a_25**	Have you ever been trained in aquaculture, either formally or informally?	[ ]	Yes, formally trained........1<br>Yes, informally trained .... 2>>L2a_26<br>Yes, both formally and informally........3<br>Not trained .... 4>>L2a_26
**L2a_25a**	When was the last time you participated in a formal training on aquaculture?	[ ]	Within the past 1 year .... 1<br>Within the past 2 year .... 2<br>Within the past 3 year .... 3<br>More than 3 years ago........ 4
**L2a_26**	Do you have access to extension services for your fish?	[ ]	Yes .... 1<br>No........ 2
**L2a_27**	What information source do you rely on the most to help you raise your fish well?	[ ]	Family/friend/neighbor .... 1<br>AG Extension Worker........ 2<br>School .... 3<br>Radio program .... 4<br>Television........ 5<br>Mobile phone messaging .... 6<br>Internet .... 7<br>Others (Specify) 96

Module End Time: | Hour | | | Minute | | |

Household Number: | | | | |

Module L2b: Pond area or diagram (The whole farm) (Male)

Module start time: | Hour | | | Minu | | |

Respondent ID: | |

Consent : | |

Yes ...1
No.....2

MAP OUT/DRAW THE PONDS WHERE FISH ARE RAISED. INDICATE THE LOCALITY AND NUMBER EACH POND. THE PONDS IDENTIFIED IN THIS MODULE WILL BE USED WITH MODULE L2c

North

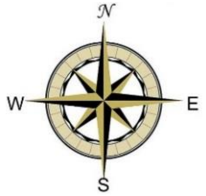

Household

East

West

South

Module End Time: | Hour | | | Minute | | |

Household Number: | | | | |

Module L2c: Pond Area Measurement (Male)

Module start time: Hour | | | Minu | | | Respondent ID: | | Consent : | | Yes ...1 No.....2

Question number	A	B	C	D	E
L2c_01	Write the plot ID of the pond from module G	Pond 1 [ ]	Pond 2 [ ]	Pond 3	Pond 4
L2c_02	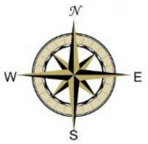<br>Draw the pond diagram =>>	Pond 1	Pond 2	Pond 3	Pond 4
**Note**L2c_03:<br><br>Walk the perimeter of the pond, write down the number of steps walked in length and width and record that in the first row of Number of steps. Then multiply the number of steps by 1.5 to convert it to meter.<br>(Length X 1.5, Width X 1.5), Finally write it in the row mentioned meter.	Number of steps<br>**Length** [ ][ ] X [ ][ ] **Width**<br><br>Meter<br>**Length** [ ][ ] X [ ][ ] **Width**	Number of steps<br>**Length** [ ][ ] X [ ][ ] **Width**<br><br>Meter<br>**Length** [ ][ ] X [ ][ ] **Width**	Number of steps<br>**Length** [ ][ ] X [ ][ ] **Width**<br><br>Meter<br>**Length** [ ][ ] X [ ][ ] **Width**	Number of steps<br>**Length** [ ][ ] X [ ][ ] **Width**<br><br>Meter<br>**Length** [ ][ ] X [ ][ ] **Width**

Household Number: [ ] [ ] [ ] [ ]

Now multiply the length and width and record the below for each pond:					
L2c_03	Walk the perimeter of the pond and enter the area in square meters<br><br>Length X Width	[ ][ ][ ][ ][.][ ]<br>(Square meter)<br><br>Not present .... 9994<br>Refused .... 9995<br>Others 9996	[ ][ ][ ][ ][.][ ]<br>(Square meter)<br><br>Not present .... 9994<br>Refused .... 9995<br>Others 9996	[ ][ ][ ][ ][.][ ]<br>(Square meter)<br><br>Not present .... 9994<br>Refused .... 9995<br>Others 9996	[ ][ ][ ][ ][.][ ]<br>(Square meter)<br><br>Not present .... 9994<br>Refused .... 9995<br>Others 9996
L2c_04	End time of the module	[ ][ ]<br>Hour<br>[ ][ ]<br>Minute	[ ][ ]<br>Hour<br>[ ][ ]<br>Minute	[ ][ ]<br>Hour<br>[ ][ ]<br>Minute	[ ][ ]<br>Hour<br>[ ][ ]<br>Minute

Module End Time: Hour [ ][ ] Minute [ ][ ]

Household Number: [ ] [ ] [ ] [ ]

Module Start Time: Hour [ ] [ ] Minute [ ] [ ] **Respondant ID:** [ ] Respondant's Consent**:** [ ] Yes ......1 No 2

Module M: Marketing of Agriculture, Livestock and Fisheries Products (Male)

Module M1: Marketing of Paddy, Rice, Banana, Mango and Potato (Male)

Information regarding the sale of paddy, rice, banana, mango and potato. Recall period is last 1 year: DECEMBER 1 2017 to NOVEMBER 30, 2018.

LIST ALL SALES; EACH LINE SHOULD BE A SEPARATE TRANSACTION (SALE), NOT AGGREGATE SALES FOR THAT PRODUCT.

Sale Serial No.	Sale Product	Month of sale Jan-1, Dec-12	Buyer	Major reason for the choice of this buyer	Total qty sold in this sale	Unit code of this sale Mond .....1 Kg..........2 Numbers 3 Sacks......4 Kandi .....5 (if unit is kg, skip M1_08)	Kgs per unit sold?	Price received per unit	Total amt received in sale	Modes of payment	% paid in cash and immediately (if 100% go to m1_14)	If buyer paid later, then after how many days?	Did you contact buyer over cell phone before sale? Yes...1 No....2>m1_16	If yes, to M14, was price agreed on cell phone? Yes...1 No...2	Sale location	Dist. and time taken to go to the place where you sold your produce?		Transport means	Transaction time on locate-on of sale	Does the buyer provide any input as advance to the farmer? Yes...1 No…2
	Code 1	No.	Code 2	Code 3	No	Code ↑	kg	Tk	Tk	Code 4	%	days			Code 5	hour	km	Code 6	Hour	
**M1_01**	**M1_02**	**M1_03**	**M1_04**	**M1_05**	**M1_06**	**M1_07**	**M1_08**	**M1_09**	**M1_10**	**M1_11**	**M1_12**	**M1_13**	**M1_14**	**M1_15**	**M1_ 16**	**M1_17**	**M1_18**	**M1_ 19**	**M1_ 20**	**M1_21**

Household Number: | | | | |

Module M2: Marketing of livestock, Jute, Wheat, Pulses, Fish, Fruits, Vegetables, etc. (Male)

Report for last transaction in the past 1 year: December 1, 2017 to November 30, 2018.

Exclude the following items: Paddy, Rice, Banana, Mango and Potato. List all sales, each line should be a separate transaction (sale), not aggregate over sales for that product.

Sale serial No.	Sale Product Code	Month of sale	Buyer	Major reason for the choice of this buyer	Total qty sold in this sale	Unit code of this sale<br><br>Mond.... 1<br>Kg .... 2<br>Numbers .... 3<br>Sacks .... 4<br>Kandi.... 5<br><br>(if unit is kg, skip M2_08)	Kgs per unit sold?	Price receivedd per unit	Total amt received in sale	Modes of payment	% paid in cash and immediately (if 100 percent go to m2_14)	If buyer paid later, then after how many days?<br><br>(put "-" if 100% is paid in cash at the time of transaction)	Did you contact buyer over cell phone before sale?<br>Yes...1<br>No....2> M2_16	If yes, to M14, was price agreed on cell phone?<br><br>Yes...1<br>No....2	Sale location	Distance and time taken to go to the place where you sold your produce?		Transport means	Transaction time on location of sale	Does the buyer provide any input as advance to the farmer?<br><br>Yes...1<br>No....2
	Code 1	No.	Code 2	Code 3	No	Code ↑	kg	Tk	Tk	Code 4	%	days			Code 5	hour	km	Code 6	Hour	
**M2_01**	**M2_02**	**M2_03**	**M2_04**	**M2_05**	**M2_06**	**M2_07**	**M2_08**	**M2_09**	**M2_10**	**M2_11**	**M2_12**	**M2_13**	**M2_14**	**M2_15**	**M2_16**	**M2_17**	**M2_18**	**M2_19**	**M2_20**	**M2_21**

Household Number: | | | |

Code list for Module M:

Code 1

Item	Code
Paddy/ rice	1
Rice	2
Wheat (a food crop)	3
Potato	4
Dal	5
Duck/Chicken	6
Cow/Buffalo/Milk cow	7
Goat/ Lamb	8
Goat/Lamb	9
Fish	10
Shrimp	11
Banana	12
Papaya	13
Jack fruit	14
Mango	15
Guava	16
Eggplant	17
Tomato	18
Gourd	19
Palang shak	20
Lal shak	21
Pui shak	22
Jute	23
Shorisha	24
Onion	25
Garlic	26
Others (specify)	27

Customer code 2

Item	Code
Village collector	1
Wholesaler	2
Cold storage owner	3
Cold storage wholesale	4
Collection center of company	5
Processing farm	6
Co-operative society	7
Farmer society	8
Retailer	9
Consumer	10
Hotel/ restaurant	11
Others (please specify)	12

Customer's choice code 3

Item	Code
Pays high/good price	1
Buys a bulk	2
Buys limited quantity	3
Makes advance Payment	4
Makes immediate Payment	5
Lives nearby	6
No other option	7

Payment code 4

Item	Code
Cash	1
Ingredients	2
Part cash & part goods	3
Cheque	4
Others (please specify)	5

Farmer's sale center code 5

Item	Code
Farmer's field / own village	1
Local retail market	2
District wholesale market	3
Other district Wholesale market	4
Dhaka wholesale market	5
Other wholesale market	6
Cold storage	7
Wholesale collection center	8
Others (please specify)	9

Transportation code 6

Item	Code
Porter/ Self carrying	1
Rickshaw	2
Van	3
Push van	4
Tractor	5
Truck	6
Motorcar	7
Bicycle	8
Motor bicycle	9
Horse cart	10
Bullock cart	11
Others (please specify)	12
From own home	13

Module End Time: Hour | | | Minute | | |

Household Number: | | | |

Module N: Non-agricultural Enterprises (Male)

Module Start Time: Hour | | | Minute | | | **Respondant ID:** | | Respondant's Consent**:** | | Yes ......1 No 2

Ask member who is most knowledgeable about household's economic activities

Question Number	MID	Question	Response	Response Code
N01		Has anyone in your household owned or operated any non-farm economic activity or business in the last 12 months?		Yes ....1<br>No ....2>> End module

Enterprise	What type of business is/was this?		Who in the household made the decision to start the business?<br><br>If household member write member ID. If not, use code 2.			Who in the household would you consider the owners of the business?<br><br>If household member write member ID. If not, use code 2.			What was your profit in the last 12 months (excluding [illegible]	For how long has this enterprise been operating?		Is the business still in operation?<br>1 = Yes (>>N10a)	Who in the household made the decision to sell/end the business activities?<br><br>If household member write member ID. If not, use code 2.			Who in the household had control over any money from the sale or closure of the business?<br><br>If household member write member ID. If not, use code 2.			Who in the household is/was the principal manager/administrator of the business (responsible for day to day operations)?<br><br>If household member write member ID. If not, use code 2.			Who in the household work/worked in the business?<br><br>If household member write member ID. If not, use code 2.		
No	Description	Business Code 1	Mid/ code 2	Mid/ code 2	Mid/ code 2	Mid /code 2	Mid /code 2	Mid/ code 2	Tk	Yr	Month		Mid / code 2	Mid/ code 2	Mid/ code 2	Mid/ code 2	Mid / code 2	Mid/ code 2	Mid/ code 2	Mid / code 2	Mid/ code 2	Mid/ code 2	Mid / code 2	Mid/ code 2
**BID**		**N02**	**N03 a**	**N03 b**	**N03 c**	**N04 a**	**N04 b**	**N04 c**	**N05**	**N06a**	**N06b**	**N07**	**N08 a**	**N08 b**	**N08 c**	**N09 a**	**N09 b**	**N09 c**	**N10 a**	**N10 b**	**N10 c**	**N11a**	**N11 b**	**N11c**
i																								
ii																								
iii																								
iv																								
v																								

Household Number: | | | |

Module N: Non-agricultural Enterprises (continued….) (Male)

Enterprise		What type of business is/was this?	Who in the household controls/controlled the money earned from the business?<br><br>If household member write member ID. If not, use code 2.			Where do you operate the enterprise?<br><br>Home…1<br>Fixed location outside home…2<br>No fixed place….3	How many months did the enterprise operate in the past 12 months?	What is your share of equity in this enterprise?	What share of profit is kept by your HH?	Who are your main customers?<br><br>Other Household/ Individuals.....1<br>Govt/other public Firm...........2<br>Private enterprise...3<br>Other(specify) 4		Where do you sell?<br>From farm/home1...1<br>Village market (within own village).........2<br>Village market (outside own village).........3<br>Town market..4<br>Others (please specify) 5	Is the enterprise registered with the govt. or local govt.?<br><br>Yes.......1<br>No........2<br>N/A......3	What was your main source of finance for setting up the business?<br><br>(write down the 2 most important)<br><br>(Code-3)		How many people did you employ over the past 12 months?	What problems if any have you had in running your business?<br><br>(write down the 2 most important)	
No		Business Code 1	Mid/ code 2	Mid/ code 2	Mid/ code 2		Month	%	%	1st	2nd			Code 3	Code 3	Number	Code 4	Code 4
**BID**		**N02**	**N12a**	**N12b**	**N12c**	**N13**	**N14**	**N15**	**N16**	**N17a**	**N17b**	**N18**	**N19**	**N20a**	**N20b**	**N21**	**N22a**	**N22b**
i																		
ii																		
iii																		
iv																		
v																		
vi																		

Household Number: [ ] [ ] [ ] [ ]

Code list for module N:

Business Code 1	Ownership/Decision-making Code 2	Source of finance Code 3	Business Problems code 4
Nursery ... 1	All members jointly ... 71	Inherited/ through gift ... 1	No major problem ... 1
Fishing ... 2	Male outside household ... 72	Own savings ... 2	Inadequate capital or credit ... 2
Mining and quarrying ... 3	Female outside household ... 73	Borrowing from relatives/ friends ... 3	Inadequate tech. knowledge ... 3
Manufacturing ... 4	Govt / Khas land/other institutions 74	Agricultural Dev. Bank ... 4	Lack of required expertise ... 4
Electricity, gas and water supply ... 5	Not applicable ... 98	Commercial bank ... 5	High-cost of running rent. ... 5
Construction ... 6		Grameen bank ... 6	Water supply problem ... 6
Wholesale and retail trade; repair of motor vehicles, motorcycles, and personal and household goods ... 7		Other financial Inst. ... 7	Power supply problem ... 7
Hotels and restaurants ... 8		NGO/ Relief agency ... 8	Problems with equipment/ spare parts ... 8
Transport, storage and communications 9		Sale of assets ... 9	Government regulations ... 9
Financial intermediation ... 10		Money lender ... 10	Lack of raw materials ... 10
Real estate and business ... 11		Other(specify) ... 11	Lack of customers ... 11
Administration, security and social safety 12		Not applicable ... 12	Transport problems ... 12
Education/Science ... 13			Business problems owing to deterioration of laws and regulations ... 13
Health and Social work ... 14			Other ... 14
Other social services ... 15			Not applicable ... 15
Own household production ... 16			
Work Out of state ... 17			
Food processing ... 21			
Garments ... 22			
Wooden furniture ... 23			
Publishing/printing ... 24			
Other industries ... 25			
Fish farming ... 26			
Forestry ... 27			
Wholesale ... 28			
Retailer ... 29			
Other business ... 30			
Transportation ... 31			
Communications ... 32			
Army/police/BDR ... 33			
Arts/culture ... 34			
Sports/tourism/leisure ... 35			
Banking/finances/loans ... 36			
Management and administration ... 37			
Non-agricultural day labourer ... 38			
Others ... 39			

Module End Time: Hour [ ] [ ] Minute [ ] [ ]

Household Number: | | | | |

Module XX: Early Marriage (Female)

Module XXa: Early Marriage (Female)

Module start time: Hour | | | Minu | | | Respondent ID: | | Consent : | | Yes ...1 No.....2

[This module applies to female respondents only].

These questions are regarding all females who are <u>married out</u> and is currently below the age of 31, and who were members of the household during BIHS Round 1 Survey in 2011. The primary female is the respondent for this module. [Information on all females who were married out and is currently below the age of 31 but who have returned to this household permanently (due to separation, divorce or death of husband) will be recorded <u>in the next module,</u> **Module XXb***].*

Serial #	ID<br>Record ID number of all applicable females starting from ID	MID<br>(MID from BIHS Round 1)	Name	What was [name's] husband's age at the time of her marriage?	What was [name's] age at menarche?	What is her current educational attainment? (record highest class passed)	What was her educational attainment at the time of her marriage? (record highest class passed)	What is her husband's current educational attainment? (record highest class passed)	What was her husband's educational attainment at the time of her marriage? (record highest class passed)	What was [name's] age at the time of her marriage?	What is [name]'s father's educational attainment?	What is [name]'s mother's educational attainment?	Was [name's] father alive at the time of [name]'s marriage?<br><br>Yes 1<br>No 2	What was [names]'s father's occupation at the time of [name's] marriage?	Was [name's] mother alive at the time of [name]'s marriage?<br><br>Yes 1<br>No 2	How many brothers did [name] have at the time of marriage?	How many sisters did [name] have at the time of marriage?	Amount of dowry paid by family?<br><br>* Does not know ….99			What amount of mahr/kabin was promised during [name's] marriage?<br><br>Note: For other religions, ask what amount of money was promised to [name] during marriage?<br><br>* Does not know ….99	What amount of mahr/kabin has been paid off during the time of the [name's] marriage?<br><br>* Does not know….99			What were the main reason why [name]'s parents got [name] married before she turned 18? (choose multiple)		
Serial no.	PID	MID	Name	years	Years	Educ code	Educ code	Educ code	Educ code	years	Educ code	Educ code	Code ↑	Occu code	Code ↑	No.	No.	Cash amount (TK):	Gold amount (TK):	In-kind amount (TK):	(TK)	Cash amount (TK):	Gold amount (TK):	In-kind amount (TK):	Code 1		
				xxa_01	xxa_02	xxa_03	xxa_04	xxa_05	xxa_06	xxa_07	xxa_08	xxa_09	xxa_10	xxa_11	xxa_12	xxa_13	xxa_14	xxa_15a	xxa_15b	xxa_15c	xxa_16	xxa_17a	xxa_17b	xxa_17c	xxa_18a	xxa_18b	xxa_18c

Household Number: | | | |

Code 1: Codes for reasons of early marriage	
She was harassed by local boys/men	1
Out of fear of harassment by boys/men	2
Thought the groom was very good and they might not get such a good choice again	3
Marriage proposals were coming for her	4
The girl herself wanted to marry	5
If the girl gets more education, it will be difficult to find her equally or more educated groom	6
If the girl gets more education, then parents might have to pay higher dowry to find her a good match	7
If the girl gets older, then parents might have to pay higher dowry to find her a good match	8
Pressure from relatives	9
Pressure from neighbors	10
Economic condition was poor to support her (food, education, clothing etc)	11
Not applicable; she was married after turning 18	12
Other (specify)	96
Do not want to respond	88

Module End Time:

Hour			Minute		

Household Number: | | | | |

Module XXb: Early Marriage (Female)

To be asked to all married females under the age of 31. [Applicable for all the daughter in laws and the females who have returned permanently in the household after separation, divorce or death of husband.]

Module start time: Hour | | | Minu | | | Respondent ID: | | Consent : | | Yes ...1 No.....2

Serial #	MID	Name	What was your husband's age at the time of your marriage?	What was your age at menarche?	What is your current educational attainment? (record highest class passed)	What was your educational attainment at the time of your marriage? (record highest class passed)	What is your husband's current educational attainment? (record highest class passed)	What was your husband's educational attainment at the time of her marriage? (record highest class passed)	What was your age at the time of your marriage?	What is your father's educational attainment?	What is your mother's educational attainment?	Was your father alive at the time of your marriage? Yes 1 No 2	What was your father's occupation at the time of your marriage?	Was your mother alive at the time of your marriage? Yes 1 No 2	How many brothers did you have at the time of marriage?	How many sisters did you have at the time of marriage?	Amount of dowry paid by family? * Does not know ….99			What amount of mahr /kabin was promised during your marriage? Note: For other religions, ask what amount of money was promised to you during marriage? * Does not know ….99	What amount of mahr/kabin has been paid off during the time of the your marriage? * Does not know….99			What were the main reason why your parents got you married before you turned 18? (choose multiple)		
Serial no.	MID	Name	years	years	Educ code	Educ code	Educ code	Educ code	years	Educ code	Educ code	Code ↑	Occu code	Code ↑	No.	No.	Cash amount (TK):	Gold amount (TK):	In-kind amount (TK):	(TK)	Cash amount (TK):	Gold amount (TK):	In-kind amount (TK):	Code 1		
			**xxb_01**	**xxb_02**	**xxb_03**	**xxb_04**	**xxb_05**	**xxb_06**	**xxb_07**	**xxb_08**	**xxb_09**	**xxb_10**	**xxb_11**	**xxb_12**	**xxb_13**	**xxb_14**	**xxb_15a**	**xxb_15b**	**xxb_15c**	**xxb_16**	**xxb_17a**	**xxb_17b**	**xxb_17c**	**xxb_18a**	**xxb_18b**	**xxb_18c**

Module End Time: Hour | | | Minute | | |

Household Number: | | | | |

Code 1: Codes for reasons of early marriage	
She was harassed by local boys/men	1
Out of fear of harassment by boys/men	2
Thought the groom was very good and they might not get such a good choice again	3
Marriage proposals were coming for her	4
The girl herself wanted to marry	5
If the girl gets more education, it will be difficult to find her equally or more educated groom	6
If the girl gets more education, then parents might have to pay higher dowry to find her a good match	7
If the girl gets older, then parents might have to pay higher dowry to find her a good match	8
Pressure from relatives	9
Pressure from neighbors	10
Economic condition was poor to support her (food, education, clothing etc)	11
Not applicable; she was married after turning 18	12
Other (specify)	96
Do not want to respond	88

Household Number: | | | |

Module O: Food Consumption (Female)

Module O1: Purchases, Home Production and Other Sources (Female)

Module start time: Hour | | | Minu | | | **Respondent ID:** | | Consent : | | Yes ...1 No.....2

Collect information for last 7 days.

Name of the item	Code	Did your household consume it in the last 7 days Yes…1 No….2	Total quantity consumed	Unit of measure? Kg............ 1 Grams...... 2 Liter......... 3 Number.... 4	If the unit of measure is number, then write the average weight in grams	Quantity consumed from purchased food				Quantity from own production	Quantity from other sources	
						Quantity consumed	Unit price	Total value	Did you purchase on credit? (note: if more than half bought on credit, write 'yes') Yes…1 No….2		Quantity	Source
				Code ↑				(Tk)				[Code1]
	**O1_01**	**O1_02**	**O1_03**	**O1_04**	**O1_05**	**O1_06**	**O1_07**	**O1_08**	**O1_09**	**O1_10**	**O1_11**	**O1_12**
**Cereals**												
Parboiled rice (coarse)	1											
Non-parboiled rice (coarse)	2											
Fine rice	3											
Rice flour	4											
Suji (cream of wheat/barley)	5											
Wheat	6											
Atta	7											
Maida (wheat flour w/o bran)	8											
Semai/noodles	9											
Chaatu	10											
Chira (flattened rice)	11											
Muri/Khoi (puffed rice)	12											
Barley	13											
Sagu	14											
Corn	15											
Cerelac	16											
Other	901											
**Pulses**												
Lentil	21											
Chick pea	22											
Anchor daal	23											
Black gram	24											
Khesari	25											
Mung	26											
Pea	27											
Shem bichi	28											
Other pulses	902											
**Edible oil**												
Soybean	31											
Mustard	32											
Dalda/banspati	33											
Ghee	34											
Palm oil	35											

Household Number: | | | | |

Name of the item	Code	Did your household consume it in the last 7 days Yes…1 No….2	Total quantity consumed	Unit of measure? Kg............ 1 Grams...... 2 Liter......... 3 Number.... 4	If the unit of measure is number, then write the average weight in grams	Quantity consumed from purchased food				Quantity from own production	Quantity from other sources	
						Quantity consumed	Unit price	Total value	Did you purchase on credit? (note: if more than half bought on credit, write 'yes') Yes…1 No….2		Quantity	Source
				Code ↑				(Tk)				[Code1]
	**O1_01**	**O1_02**	**O1_03**	**O1_04**	**O1_05**	**O1_06**	**O1_07**	**O1_08**	**O1_09**	**O1_10**	**O1_11**	**O1_12**
Sesame oil	36											
Other oil	903											
**Vegetables**												
Patal	41											
Bitter gourd	42											
Okra	43											
Eggplant	44											
BT Brinjal	441											
Tomato	45											
Pumpkin	46											
Sweet gourd	47											
Ash gourd	48											
Long bean	49											
Water gourd	50											
Sheem	51											
Carrot	52											
Radish	53											
Cauliflower	54											
Green banana	55											
Papaya	56											
Green chili	57											
Cucumber	58											
Kachu (arum)	59											
Danta (amaranth)	60											
Potato	61											
White Sweet Potato	621											
Orange Flesh Sweet Potato	622											
Green mango	63											
Onion	64											
Garlic	65											
Dhundal	66											
Shapla	67											
Kachur lati	68											
Jhinga (ribbed gourd)	69											
Green pea	70											
Fig	71											
Drum stick	72											
Snake gourd	73											
Green jackfruit	74											
Kolar mocha	75											
Mete alu	76											

Household Number: | | | | |

Name of the item	Code	Did your household consume it in the last 7 days Yes…1 No….2	Total quantity consumed	Unit of measure? Kg............ 1 Grams...... 2 Liter......... 3 Number.... 4	If the unit of measure is number, then write the average weight in grams	Quantity consumed from purchased food				Quantity from own production	Quantity from other sources	
						Quantity consumed	Unit price	Total value	Did you purchase on credit? (note: if more than half bought on credit, write 'yes') Yes…1 No….2		Quantity	Source
				Code ↑				(Tk)				[Code1]
	**O1_01**	**O1_02**	**O1_03**	**O1_04**	**O1_05**	**O1_06**	**O1_07**	**O1_08**	**O1_09**	**O1_10**	**O1_11**	**O1_12**
Beher gura	77											
Soybean bori	78											
Jack fruit seed	79											
Cabbage	80											
Kakrol	81											
Shalgom	82											
Other	904											
**Leafy vegetables**												
Pui (Indian spinach)	86											
Lal Shak (red amaranth)	87											
Bathua	88											
Bokful	89											
Cabbage	90											
Danta Shak	91											
Helencha	92											
Kalmi Shak	93											
Kachu Shak	94											
Kalo kachu Shak	95											
Katanate	96											
Lau Shak	97											
Pat Shak	98											
Dheki Shak	99											
Dhania Shak	100											
Palang Shak (spinach)	101											
Onion/garlic stalk	102											
Pea leaves	103											
Drumstick leaves	104											
Mustard leaves	105											
Radish leaves	106											
Mixed leafy vegetables	107											
Dudhali Pata	108											
Black gram leaves	109											
Shechi Shak	110											
Swett gourd leaves	111											
Khesari Shak	112											
Geema Shak	113											
Neem Shak	114											
Darkuni Shak	115											
Other leafy vegetables	905											
**Meat, eggs and milk**												
Beef/buffalo	121											

Household Number: [ ] [ ] [ ] [ ]

Name of the item	Code	Did your household consume it in the last 7 days Yes…1 No….2	Total quantity consumed	Unit of measure? Kg............ 1 Grams...... 2 Liter......... 3 Number.... 4	If the unit of measure is number, then write the average weight in grams	Quantity consumed from purchased food: Quantity consumed	Unit price	Total value	Did you purchase on credit? (note: if more than half bought on credit, write 'yes') Yes…1 No….2	Quantity from own production	Quantity from other sources: Quantity	Source
				Code ↑				(Tk)				[Code1]
	O1_01	O1_02	O1_03	O1_04	O1_05	O1_06	O1_07	O1_08	O1_09	O1_10	O1_11	O1_12
Mutton	122											
Chicken	123											
Duck	124											
Pigeon	125											
Bids/bok/gughu	126											
Pig	322											
Liver	127											
Stomach of beef/goat	128											
Dried meat	129											
Egg	130											
Fish egg	131											
Milk	132											
Powdered Milk	133											
Condensed Milk	134											
Butter	135											
Other meat	906											
**Fruits**												
Mango	141											
Banana	142											
Papaya	143											
Orange	144											
Apple	145											
Coconut	146											
Jack Fruit	147											
Litchis	148											
Black berry	149											
Bel	150											
Pomelo	151											
Grapes	152											
Amra	153											
Karambola	154											
Guava	155											
Jujube/dried jujube	156											
Olive	157											
Tamarind	158											
Dalim	159											
Lemon	160											
Dates	161											
Sugarcane	162											
Green Coconut	163											
Ata (bullock's heart)	164											

Household Number: | | | | |

Name of the item	Code	Did your household consume it in the last 7 days Yes…1 No….2	Total quantity consumed	Unit of measure? Kg............ 1 Grams...... 2 Liter......... 3 Number.... 4	If the unit of measure is number, then write the average weight in grams	Quantity consumed from purchased food				Quantity from own production	Quantity from other sources	
						Quantity consumed	Unit price	Total value	Did you purchase on credit? (note: if more than half bought on credit, write 'yes') Yes…1 No….2		Quantity	Source
				Code ↑				(Tk)				[Code1]
	**O1_01**	**O1_02**	**O1_03**	**O1_04**	**O1_05**	**O1_06**	**O1_07**	**O1_08**	**O1_09**	**O1_10**	**O1_11**	**O1_12**
Chalta	165											
Tarmuj (Watermelon)	166											
Bangi (Musk melon)	167											
Pineapple	168											
Sobeda	169											
Jaamrul	170											
Myrobalan/ Indian Gooseberry	317											
Water Caltrop	319											
Other fruit	907											
**Fish (large)**												
Rui	176											
Katla	177											
Mrigel	178											
Kalibaus	179											
Surma	180											
Chital	181											
Boal	182											
Aair	183											
Pangash	184											
Ritha	185											
Hilsa	186											
Jatka	187											
Grass Carp	188											
Mirror Carp	189											
Silver Carp	190											
Telapia	191											
Swarputi	192											
Chital	193											
Taki	194											
Mague	195											
Singi	196											
Baim	197											
Koi	198											
Meni	199											
Shapla/padda/rupsha fish	200											
Bagda Chingree	201											
Golda Chingree	202											
Tortoise meat	203											
Poona fish	204											
Dried fish	205											
Other big fish	908											

Household Number: [ ][ ][ ][ ]

Name of the item	Code	Did your household consume it in the last 7 days Yes…1 No….2	Total quantity consumed	Unit of measure? Kg............ 1 Grams...... 2 Liter......... 3 Number.... 4	If the unit of measure is number, then write the average weight in grams	Quantity consumed from purchased food: Quantity consumed	Unit price	Total value	Did you purchase on credit? (note: if more than half bought on credit, write 'yes') Yes…1 No….2	Quantity from own production	Quantity from other sources: Quantity	Source
				Code ↑				(Tk)				[Code1]
	**O1_01**	**O1_02**	**O1_03**	**O1_04**	**O1_05**	**O1_06**	**O1_07**	**O1_08**	**O1_09**	**O1_10**	**O1_11**	**O1_12**
**Fish (small)**												
Gura mach	211											
Panch mishali	212											
Puti	213											
Tengra	214											
Pabda	215											
Moa/mola	216											
Dhela	217											
Batashi	218											
Kachki	219											
Chanda	220											
Khalisa	221											
Chela	222											
Chapila	223											
Kajari	224											
Tatkeni	225											
Bata	226											
Ghutum	227											
Bele	228											
Chewa	229											
Poa	230											
Foli	231											
Bacha	232											
Baicha	233											
Kaikla	234											
Darkini	235											
Palshe	236											
Harkun	237											
Karfu fish	238											
Kakra	239											
Small prawn	240											
Dried small shrimp/prawn	241											
Dried small fish	242											
Fermented fish	243											
Other small fish	909											
**Spices**												
Dried chili	246											
Turmeric (not dried)	247											
Turmeric (dried)	248											
Jira	249											
Elachi	250											

Household Number: [ ] [ ] [ ] [ ]

Name of the item	Code	Did your household consume it in the last 7 days Yes…1 No….2	Total quantity consumed	Unit of measure? Kg............ 1 Grams...... 2 Liter......... 3 Number.... 4	If the unit of measure is number, then write the average weight in grams	Quantity consumed from purchased food: Quantity consumed	Unit price	Total value	Did you purchase on credit? (note: if more than half bought on credit, write 'yes') Yes…1 No….2	Quantity from own production	Quantity from other sources: Quantity	Source
				Code ↑				(Tk)				[Code1]
	**O1_01**	**O1_02**	**O1_03**	**O1_04**	**O1_05**	**O1_06**	**O1_07**	**O1_08**	**O1_09**	**O1_10**	**O1_11**	**O1_12**
Cinnamon	251											
Salt (Ordinary)	2521											
Salt (Iodine)	2522											
Panchforan	253											
Coriander	254											
Ginger	255											
Garam Masala	256											
Black cumin	257											
Mustard	258											
Til (sesame)	259											
Mouri	260											
Babuni/randhuni	261											
Tishi	262											
Tejpata	263											
Baking powder	264											
Raisins	318											
Chui jhaal	323											
Other	910											
**Other food**												
Sugar	266											
Gur	267											
Misri/tal misri	268											
Tea leaves	269											
Badam (ground nut)	270											
Honey	271											
**Drinks and beverages**												
Tea –prepared	272											
Coke/ Seven-up etc./Pepsi/RC/Urocola etc.	273											
Packaged Juice	274											
Horlicks/Bournvita/Tang	275											
Sugarcane/palm/date juice	276											
**Other foods prepared outside home**												
Rice/Jao	277											
Panta Bhaat	278											
Khichuri	279											
Polao/Biryani/Tehari	280											
Ruti/Parota	281											
Bonroti/paoroti	282											
Burger	283											

Household Number: | | | | |

Name of the item	Code	Did your household consume it in the last 7 days Yes…1 No….2	Total quantity consumed	Unit of measure? Kg........... 1 Grams...... 2 Liter......... 3 Number.... 4	If the unit of measure is number, then write the average weight in grams	Quantity consumed from purchased food				Quantity from own production	Quantity from other sources	
						Quantity consumed	Unit price	Total value	Did you purchase on credit? (note: if more than half bought on credit, write 'yes') Yes…1 No….2		Quantity	Source
				Code ↑				(Tk)				[Code1]
	**O1_01**	**O1_02**	**O1_03**	**O1_04**	**O1_05**	**O1_06**	**O1_07**	**O1_08**	**O1_09**	**O1_10**	**O1_11**	**O1_12**
Paes/firni/cooked firni	284											
Pitha	285											
Halua	286											
Bharta	287											
Bhaji	288											
Jhol curry	289											
Bhuna curry	290											
Daal	291											
Salad	292											
Sweets	293											
Curd	294											
Alur chap	295											
Singara	296											
Puri	297											
Piaju	298											
Chhola/ghugni/boot	299											
Achar/Chatni	300											
Chotpoti	301											
Chanachur	302											
Mowa (Puffed rice ball coated in molassces)	323											
Biscuit	303											
Cake	304											
Patties	305											
Chips	306											
Chocolate	307											
Chewing gum	308											
Gaja	309											
Murali	310											
Nimki	311											
Any fried food	312											
Any boiled food	313											
Tobacco	314											
Betel Leaf	315											
Supari	316											
Taaler shaash	320											
Ice Cream	321											

Household Number: | | | | |

Sl no	Question	Response	Response Code
**O1_13**	Where did you buy most of the food items from?		From farm/home... 1<br>Village market (within own village).................... 2<br>Village market (outside own village).................. 3<br>City market... 4<br>Others (please specify) .... 5<br>School gate……………………………………….6
**O1_14**	Who purchases most of the food for this household?		Member ID

**Code 1: Quantity from other sources**	
Loan from friend/relative ....1<br>Gift from friend/relative.....................2<br>Wages ....3<br>Collected 4	Government program..........................5<br>NGO program....................................6<br>Begged..7<br>Hunted/Fished ....8<br>Not applicable……………………… 9

Module End Time: | Hour | | | Minute | | |

Household Number: | | | |

Module O2: Household Food Inventory on the Day of Survey (Female)

(As observed and weighed by enumerator)

Module start time: Hour | | | Minu | | **Respondent ID:** | | Consent : | | Yes ...1 No.....2

Item	Item Code	Quantity	Unit<br>Kg...1<br>Gram ....2<br>Liter..3
			Code ↑
**O2_01**	**O2_02**	**O2_03**	**O2_04**
Paddy	1		
Rice	2		
Atta	3		
Cooking Oil	4		
Pulses	5		

Module End Time: Hour | | | Minute | |

Module O3: Food Consumption from Purchases, Home Production and Other Sources: Recall Period 7 Days (Female)

Module start time: Hour | | | Minu | | **Respondent ID:** | | Consent : | | Yes ...1 No.....2

Member	Code	The number of members who consumed everyday						
		Day 1 (yesterday)	Day 2 (previous day)	Day 3 (previous day)	Day 4 (previous day)	Day 5 (previous day)	Day 6 (previous day)	Day 7 (previous day)
	**O3_01**	**O3_02**	**O3_03**	**O3_04**	**O3_05**	**O3_06**	**O3_07**	**O3_08**
<10 year old male	1							
<10 year old female	2							
>10 year old male	3							
>10 year old female	4							

Module End Time: Hour | | | Minute | |

Household Number: | | | | |

Module P: Non-food Expenditure (Male)

Module Start Time: Hour | | | Minute | | **Respondant ID:** | | Respondant's Consent: | | Yes ..... 1 No 2

Module P1: Non-food Expenditure Monthly Recall (Male)

Item Name	Expenses Code	Cash expenditure (including debt)	Where do you buy most of the items from?<br><br>From farm/home.................................. 1<br>Village market (within own village)..... 2<br>Village market (outside own village) ... 3<br>City market.. 4<br>Others (please specify) 5	If not purchased	
				Value	Source:<br><br>Friend/neighbors /relatives 1<br>Own craftsmanship or stock 2<br>Collected ....3<br>Other 4
		(Tk)		(Tk)	Code ↑
	**P1_01**	**P1_02**	**P1_03**	**P1_04**	**P1_05**
**FUEL AND LIGHTING**					
Firewood	1				
Cow dung/cakes/bhushi/wood-powder	2				
Jute stick	3				
Kerosene	4				
Agri by products fuel: paddy, hag, pressed sugarcane and dried com plants, etc.	5				
Gas (natural, bio-gas)/LPG	6				
Electricity (national grid)	7				
Electricity (generator)	46				
Electricity (Solar)	47				
Pit coal, char coal, wood coal	8				
Other fuels and light (e.g. matches and candles etc.)	9				
**COSMETICS AND OTHER EXPENSES**					
Snow, cream, powder	10				
Perfume etc.	11				
Hair cutting, styling, shaving, etc.	12				
Hair oil, hair cream, combs, clips, etc.	13				
Razor, razor blades, shaving cream and lotions, etc.	14				
Lipstick, nail-polish, etc.	15				

Household Number: | | | | |

Item Name	Expenses Code	Cash expenditure (including debt)	Where do you buy most of the items from?<br><br>From farm/home.................................. 1<br>Village market (within own village)..... 2<br>Village market (outside own village) ... 3<br>City market.. 4<br>Others (please specify) 5	If not purchased	
				Value	Source:<br><br>Friend/neighbors /relatives 1<br>Own craftsmanship or stock 2<br>Collected ....3<br>Other 4
		(Tk)		(Tk)	Code ↑
	**P1_01**	**P1_02**	**P1_03**	**P1_04**	**P1_05**
Beautifying items (hair ribbon, churi, kajal, etc.)	16				
**WASHING AND CLEANING EXPENSES**					
Bath soap, shampoo, toothpaste, etc.	17				
Washing soap, powder for cloths	18				
Washing/ laundry expenses	19				
Bleaching powder, soda etc.	20				
Vim/ dish cleaning supplies	21				
Finis/ phenyl/ other household cleaning supplies	22				
Toilet papers	23				
Mosquito coil	24				
Mosquito spray	25				
Women's sanitary napkin	48				
Diaper for baby	49				
**TRANSPORT/ TRAVEL AND OTHER MISC. CHARGES**					
Bus fare	26				
Rickshaw/ van fare	27				
Taxi/ tempoo/ mishuk fare	28				
Boat/ launch fare	29				
Train fare	30				
Other transport fare (specify)	31				
Bicycle maintenance, tyres, tubes repairs etc.	32				
Motor-cycle maintenance, repairs, etc.	33				
Car maintenance, repairs, etc.	34				

Household Number: | | | | |

Item Name	Expenses Code	Cash expenditure (including debt)	Where do you buy most of the items from?<br><br>From farm/home.................................. 1<br>Village market (within own village)..... 2<br>Village market (outside own village) ... 3<br>City market.. 4<br>Others (please specify) 5	If not purchased	
				Value	Source:<br><br>Friend/neighbors /relatives 1<br>Own craftsmanship or stock 2<br>Collected ....3<br>Other 4
		(Tk)		(Tk)	Code ↑
	**P1_01**	**P1_02**	**P1_03**	**P1_04**	**P1_05**
Boat maintenance, repairs, etc.	35				
Petrol	36				
Diesel	37				
Motor oil/CNG etc.	38				
Other transport, repair and maintenance.	39				
Telephone bill/ charges/mobile	40				
Telegram, postal and courier service expenses, etc.	41				
Salaries and wages of servants	42				
Salaries and wages of drivers	43				
Salaries and wages of others including guards, gardeners, housekeepers etc.	44				
Other contingencies expenses (specify)	45				

Household Number: | | | | |

Module P2: Non-food Expenditure Annual Recall (Male)

Item Name	Expenses Code	If purchased			If not purchased		
		How many did you buy?	Cash expenditure (including debt)	Where do you buy most of the items from?<br>From farm/home.................................. 1<br>Village market (within own village) .... 2<br>Village market (outside own village) ... 3<br>City market.. 4<br>Others (please specify) 5	How many did you produce at home/receive as gift?	Value	Source:<br>Friends/Relatives/Neighbors/others…1<br>own craftsmanship or stock .... 2<br>Collected..................................... 3<br>Others.. 4
		(Number)	(Tk)		(Number|)	(Tk)	Code
	**P2_01**	**P2_02**	**P2_03**	**P2_04**	**P2_05**	**P2_06**	**P2_07**
For adult:<br>Lungi/dhuti	51						
Shirts	52						
Pant	53						
Saree	54						
Blouse/ petticoat	55						
Salwar kameez/ Orna	56						
Punjabi/ Pajamas	57						
Suit, overcoat, ashkan, etc.	58						
Sweaters, Jacket, pullovers, mufflers, etc.	59						
Underwear etc.	60						
Socks, handkerchiefs, scarves, caps, neckties etc.	61						
Other (specify)	62						
For children:							
Lungi/dhuti	63						
Full pant	64						
Half pant	65						
Shirts	66						
T-shirt	67						
Frocks, dresses, babysuit etc.	68						
Socks, handkerchiefs, scarves, caps, neckties etc.	69						
Sweaters, Jacket, pullovers, mufflers, etc.	70						
Other for children (specify)	71						
**Both**							

Household Number: | | | | |

Item Name	Expenses Code	If purchased			If not purchased		
		How many did you buy?	Cash expenditure (including debt)	Where do you buy most of the items from?<br>From farm/home.................................. 1<br>Village market (within own village) .... 2<br>Village market (outside own village) ... 3<br>City market.. 4<br>Others (please specify) 5	How many did you produce at home/receive as gift?	Value	Source:<br>Friends/Relatives/Neighbors/others…1<br>own craftsmanship or stock .... 2<br>Collected..................................... 3<br>Others... 4
		(Number)	(Tk)		(Number|)	(Tk)	Code
	**P2_01**	**P2_02**	**P2_03**	**P2_04**	**P2_05**	**P2_06**	**P2_07**
Towel, Gamcha	72						
Chador, shawl, etc.	73						
Other (specify)	74						
Clothing material and tailoring							
Clothing material and tailoring	75						
Mill-made cloth/ fabric	76						
Hand loom cloth/ fabric	77						
Drill and other cloth for trousers	78						
Woolen cloth	79						
Silk	80						
Artificial silk etc.	81						
Other artificial-yarn made cloths	82						
Tailoring expenses	83						
Other clothing related expenses	84						
Footwear							
Leather shoes	85						
Leather sandal-shoes	86						
Leather sandal	87						
Plastic shoes	88						
Plastic sandal-shoes	89						
Other sandal	90						
Canvas shoes, sports shoes, etc.	91						
Wooden sandals	92						
Shoe brush, polish, cleaning supplies, etc.	93						
Maintenance and repair expenses of foot wear	94						
Other expenses regarding footwear	95						
Household-use textiles, etc.							

Household Number: [ ] [ ] [ ] [ ]

Item Name	Expenses Code	If purchased			If not purchased		
		How many did you buy?	Cash expenditure (including debt)	Where do you buy most of the items from?<br>From farm/home.................................. 1<br>Village market (within own village) .... 2<br>Village market (outside own village) ... 3<br>City market.. 4<br>Others (please specify) 5	How many did you produce at home/receive as gift?	Value	Source:<br>Friends/Relatives/Neighbors/others…1<br>own craftsmanship or stock .... 2<br>Collected...................................... 3<br>Others.. 4
		(Number)	(Tk)		(Number|)	(Tk)	Code
	**P2_01**	**P2_02**	**P2_03**	**P2_04**	**P2_05**	**P2_06**	**P2_07**
Quilt/blanket/Katha	96						
Toshok	97						
Jajim	98						
Foam sheet	99						
Bed sheets	100						
Bed cover	101						
Pillows, cushions	102						
Pillow cover, cushion cover	103						
Table cover	104						
Curtain	105						
Mosquito netting	106						
Other (specify)	107						
Housing related expenses							
House rent (rented house)	108						
Imputed rent (owner-occupied or other)	109						
Water/ sewerage charges	110						
Home additions/ improvements	111						
Painting	112						
Disaster-related maintenance/ repair	113						
Other routine maintenance/ repair	114						
Municipal tax	115						
Other related services/ expenses	116						
Medical treatment expenses (male)							
Doctor's fees	117						
Other practitioner's fees (homeopath etc.)	118						
Medicines	119						

Household Number: [ ][ ][ ][ ]

Item Name	Expenses Code	If purchased: How many did you buy?	If purchased: Cash expenditure (including debt)	If purchased: Where do you buy most of the items from? From farm/home.................................. 1 Village market (within own village) .... 2 Village market (outside own village) ... 3 City market.. 4 Others (please specify)....... 5	If not purchased: How many did you produce at home/receive as gift?	If not purchased: Value	If not purchased: Source: Friends/Relatives/Neighbors/others…1 own craftsmanship or stock .... 2 Collected..................................... 3 Others.. 4
		(Number)	(Tk)		(Number|)	(Tk)	Code
	**P2_01**	**P2_02**	**P2_03**	**P2_04**	**P2_05**	**P2_06**	**P2_07**
Ayurvedic/ Kabiraji	120						
Medical Tests (X-ray, blood, urine etc.)	121						
Hospitalization, clinic charges, etc.	122						
Dental related expenses	123						
Spectacles	124						
Hearing aids	125						
Crutches	126						
Health-related travel/ incidental expenses	127						
Medical treatment expenses (female)							
Doctor's fees	128						
Other practitioner's fees (homeopath etc.)	129						
Medicines	130						
Ayurvedic/ Kabiraji	131						
Medical Tests (X-ray, blood, urine etc.)	132						
Hospitalization, clinic charges, etc.	133						
Dental related expenses	134						
Spectacles	135						
Hearing aids	136						
Crutches	137						
Maternity expenses	138						
Health-related travel/ incidental expenses	139						
Educational expenses (male)							
Registration fees	140						
Examination fees	141						

Household Number: | | | | |

Item Name	Expenses Code	If purchased			If not purchased		
		How many did you buy?	Cash expenditure (including debt)	Where do you buy most of the items from?<br>From farm/home.................................. 1<br>Village market (within own village) .... 2<br>Village market (outside own village) ... 3<br>City market.. 4<br>Others (please specify) 5	How many did you produce at home/receive as gift?	Value	Source:<br>Friends/Relatives/Neighbors/others…1<br>own craftsmanship or stock .... 2<br>Collected .... 3<br>Others.. 4
		(Number)	(Tk)		(Number|)	(Tk)	Code
	**P2_01**	**P2_02**	**P2_03**	**P2_04**	**P2_05**	**P2_06**	**P2_07**
Annual fees	142						
School fees	143						
Personal Teaching expenses	144						
Text book/ note books/ stationary	145						
Hostel Expenses	146						
Other	147						
Educational expenses (female)							
Registration fees	148						
Examination fees	149						
Annual fees	150						
School fees	151						
Personal Teaching expenses	152						
Text book/ note books/ stationary	153						
Hostel Expenses	154						
Other	155						
Remittances, ceremonies, gifts, etc.							
Remittances to others living separately	156						
Zakat	157						
Fitra	158						
Donation/ Sadqa	159						
Qurbani	160						
Religious functions (milad etc.)	161						
Expenditure on Hajj	162						
Expenditure on Pilgrimage	163						
Expenditure on marriage	164						
Expenditure on births	165						
Expenditure on deaths	166						
Other (specific)	167						

Household Number: | | | | |

Item Name	Expenses Code	If purchased			If not purchased		
		How many did you buy?	Cash expenditure (including debt)	Where do you buy most of the items from?<br>From farm/home.................................. 1<br>Village market (within own village) .... 2<br>Village market (outside own village) ... 3<br>City market.. 4<br>Others (please specify) 5	How many did you produce at home/receive as gift?	Value	Source:<br>Friends/Relatives/Neighbors/others…1<br>own craftsmanship or stock .... 2<br>Collected .... 3<br>Others.. 4
		(Number)	(Tk)		(Number|)	(Tk)	Code
	**P2_01**	**P2_02**	**P2_03**	**P2_04**	**P2_05**	**P2_06**	**P2_07**
Recreation & leisure, etc.							
Books, newspaper, magazines, story books	168						
Cinema	169						
Theater	170						
Variety shows, concerts, etc.	171						
Sporting expenses, club membership fees, etc.	172						
Video cassette purchases and rental etc.	173						
Audio cassette purchases etc.	174						
Photography	175						
TV/ video/ satellite license fees, etc.	176						
Other recreation, marriage day/birthday, tourism & leisure related expenses	177						
Taxes, interest, fines, etc.							
Income tax	178						
Bank interest charge, Payment of banking charge	179						
Fines	180						
Legal practitioner fees	181						
Other legal expenses	182						
Property registration fees	183						
Other (specify)	184						
Cooking equipment							
Glass/china/clay plates and dishes etc.	185						
Refrigerators	186						

Household Number: ☐☐☐☐

Item Name	Expenses Code	If purchased			If not purchased		
		How many did you buy?	Cash expenditure (including debt)	Where do you buy most of the items from?<br>From farm/home.................................. 1<br>Village market (within own village) .... 2<br>Village market (outside own village) ... 3<br>City market.. 4<br>Others (please specify) 5	How many did you produce at home/receive as gift?	Value	Source:<br>Friends/Relatives/Neighbors/others…1<br>own craftsmanship or stock .... 2<br>Collected .... 3<br>Others.. 4
		(Number)	(Tk)		(Number|)	(Tk)	Code
	**P2_01**	**P2_02**	**P2_03**	**P2_04**	**P2_05**	**P2_06**	**P2_07**
Stove (electric/ gas/ kerosene)	187						
Pots/ pans	188						
Other kitchen ware and utensils	189						
spoons/ forks/ knives etc.	190						
Micro oven/Pressure cooker	191						
Others	192						
Furniture							
Bed/Chowki	193						
Table/Chair/Dressing Table	194						
Sofa	195						
Wood/Iron Almirah/Wardrobe/Bookshelf	196						
Trunk/Suitcase	197						
Costs of Furniture Building/Repair/Polish	198						
Other furniture related cost	199						
Personal articles							
Gold Jewelry	200						
Silver Jewelry	201						
Imitation Jewelry	202						
Purses/ money bags	203						
Vanity bags	204						
Umbrella, walking stick	205						
Tie-pin, cigarette cases, lighter etc.	206						
Wrist watch/ clock etc.	207						
Other personal use items (belts, etc.>)	208						
Misc. Household durable							
Radio	210						

Household Number: | | | | |

Item Name	Expenses Code	If purchased			If not purchased		
		How many did you buy?	Cash expenditure (including debt)	Where do you buy most of the items from?<br>From farm/home.................................. 1<br>Village market (within own village) .... 2<br>Village market (outside own village) ... 3<br>City market.. 4<br>Others (please specify) 5	How many did you produce at home/receive as gift?	Value	Source:<br>Friends/Relatives/Neighbors/others…1<br>own craftsmanship or stock .... 2<br>Collected .... 3<br>Others.. 4
		(Number)	(Tk)		(Number|)	(Tk)	Code
	**P2_01**	**P2_02**	**P2_03**	**P2_04**	**P2_05**	**P2_06**	**P2_07**
Two-in-one	211						
Black & White Television	212						
Colored Television	213						
VDO game set	214						
VCD/ VCR/dish antenna/cable membership fees	215						
Washing machine, iron, etc.	216						
Guitar/ orchestra/ harmonium	217						
Typewriter, personal computer etc.	218						
Lenten/ chimney lamp etc.	219						
Electric fans, air-conditioners, coolers, etc.	220						
Cameras/ camcorders/video camera	221						
Pen drive/flash drive/ memory card	222						
Insurance expenditure							
Life insurance	223						
Health insurance	224						
General insurance	225						

Module End Time: Hour | | | Minute | | |

Household Number: | | | | |

Module Q: Housing (Male)

Ask of household head or other senior member of household

Module Start Time: Hour | | | Minute | | | **Respondant ID:** | | Respondant's Consent: | | Yes ..... 1 No....... 2

Question number	Question	Response	Response option
**Q_01**	Do you own this house, do you use it for free, or do you rent this house?		Owned..............................1<br>Free ....2<br>Rented ....3 (Go to Q03)
**Q_02**	Estimate the monthly rent you could receive if you rented this dwelling or one exactly like it to another person?		Taka<br>(if don't know…..9999)<br>Next>> move to Q_04
**Q_03**	How much monthly rent do you pay for this dwelling?		Taka
**Q_04**	How many years ago was this house built? How old is it?		Years<br>(if don't know…..9999)
**Q_05**	If this household shares space with another household, how many households live in this house?		Number<br>(Write "0" if no sharing)
**Q_06**	*OBSERVE*<br>What type of dwelling does the household live in?		No sign of damage ....1<br>Slightly damaged ....2<br>Somewhat damaged ....3<br>Very damaged...................4<br>In a very poor state 5
**Q_07**	*OBSERVE*<br>The outer walls of the main dwelling of the household are predominantly made of what material?		Concrete/Brick..................1<br>Tin/CI Sheet......................2<br>Wood ....3<br>Mud..................................4<br>Bamboo.............................5<br>Jute straw ....6<br>Plastic /Polythene..............7<br>Cardboard/paper ....8<br>Golpaata/Palm leaf............9<br>Grass/Straw.......................10<br>Other (specify) 11

Household Number: | | | | |

Question number	Question	Response	Response option
**Q_08**	*<u>OBSERVE</u>*<br>The <u>roof</u> of the main dwelling is predominantly made of what material?		Concrete/Brick.................1<br>Tin/CI Sheet.....................2<br>Wood ....3<br>Mud.................................4<br>Bamboo............................5<br>Jute straw ....6<br>Plastic /Polythene.............7<br>Cardboard/paper ....8<br>Golpaata/Palm leaf............9<br>Grass/Straw.......................10<br>Other (specify).................11
**Q_09**	*<u>OBSERVE</u>*<br>The <u>floor</u> of the main dwelling is predominantly made of what material?		Concrete/Brick.................1<br>Tin/CI Sheet.....................2<br>Wood ....3<br>Mud.................................4<br>Bamboo............................5<br>Jute straw ....6<br>Plastic /Polythene.............7<br>Cardboard/paper ....8<br>Golpaata/Palm leaf............9<br>Grass/Straw.......................10<br>Other (specify).................11
**Q_10**	How many rooms does your household occupy?		Number<br>(Exclude rooms used for business)
**Q_11**	How many rooms are used for sleeping?		Number
**Q_11b**	Is the cooking usually done in the house, in a separate building, or outdoors?		IN THE HOUSE. 1>> **Q12**<br>IN A SEPARATE BUILDING……..2<br>OUTDOORS 3
**Q_11c**	Do you have a separate room which is used as a kitchen?		YES.................... 1<br>NO 2
**Q_12**	*<u>OBSERVE</u>*<br>What is the total floor area of the dwelling in square feet? (measuring tape to be provided)		Square feet
**Q_13**	Does this household have an electricity connection?		Yes...................................1<br>No ....2>>**Q16**

Household Number: ☐☐☐☐

Question number	Question	Response	Response option
**Q_14**	How often does the electricity supply go off?	☐	Never ....1<br>Rarely................................2<br>Less than half the time ......3<br>About half the time ....4<br>More than half...................5<br>Almost always 6
**Q_15**	How much was the electricity cost last month?	☐	Taka
**Q_16**	What is your main source of cooking fuel?	☐	Electricity..........................1>>**Q18**<br>Supply gas.........................2<br>LPG..................................3<br>Kerosene ....4<br>Firewood ....5<br>Dried cow dung.................6<br>Coal..................................7<br>Rice bran/saw dust ....8<br>Dried leaves ....9<br>Other (specify) 10
**Q_17**	What was the total cost for cooking fuel in the household in the past month?	☐	Taka
**Q_18**	What is your main source of lighting fuel?	☐	Electricity..........................1>>**Q19a**<br>Private Generator ....2<br>Solar electricity ....3<br>Kerosene ....4<br>Candles ....5<br>Torch/fire skewer……………….6<br>Others…………………………...7
**Q_19**	What was the total cost for lighting fuel in the household in the past month?	☐	Taka
**Q_19a**	Has the household used biogas for any purpose?	☐	Yes, my own plant .... 1<br>Yes, renting via line .... 2<br>No .... 3>>**Q20**

Household Number: [ ] [ ] [ ] [ ]

Question number	Question	Response	Response option
**Q_19b**	If yes, for what purpose was biogas used?		Only for cooking........................ 1<br>Only for lighting .... 2<br>For both lighting and cooking.... 3<br>Business .... 4<br>Business and for lighting or cooking 5<br>Others.. 6
**Q_19c**	If yes, then from which year?		Year
**Q_20**	How many cellular telephones are there in working condition?		Number<br>(if no cell phones exist then record 0)
**Q_20a**	Do you have a personal cellular telephone? If yes, can you please give me your number?	Y/N MID<br>Mobile No.	Yes.. 1<br>No 2
**Q_20b**	Does anyone else in the household have a cellphone?	Y/N MID<br>Mobile No.	Yes.. 1<br>No 2
**Q_20c**	Does anyone else in the household have a cellphone?	Y/N MID<br>Mobile No.	Yes.. 1<br>No 2

Household Number: | | | | |

Question number	Question	Response	Response option
**Q_20d**	If yes, I transfer using	Y/N MID Mobile No.	Bkash .... 1<br>Ucash .... 2<br>Mcash... 3<br>DBBL Mobile .... 4<br>Other mobile banking(specify) . 5<br>No mobile banking available ..... 6<br>No one in the HH has a mobile phone…7
**Q_21**	What was the total cost for cell phone service for all household members last month?		Taka

Module End Time: | Hour | | | Minute | | |

Module R: Sanitation and Water (Male)

Module Start Time: | Hour | | | Minute | | | **Respondant ID:** | | Respondant's Consent: | | Yes ..... 1 No 2

Question number	Question	Response	Response Code
**R01**	What type of latrine do you use?		None (open field) ....1<br>Kutcha (fixed place) ....2<br>Pucca (unsealed)..............................3<br>Sanitary without flush (water sealed) .... 4<br>Sanitary with flush (water sealed) ...5<br>Community latrine...........................6<br>Other (specify)................................7
**R01a**	What type of latrine do you use? Can I see the latrine that is used by your household members?		***Note: Please observe and write the type of the latrine***<br>Open field......................................1<br>Latrine, slab cannot be cleaned ....2<br>Latrine, slab can be cleaned(concrete/china) 3
**R01b**	Where does the discharged material go?		Directly to the pond, canal or other water body….1<br>Sealed pit... 2
**R01c**	***Please observe:*** only if the latrine is made by slab that can be cleaned (made of concrete/china)		Flush at septic.................................1<br>Can be flushed................................2<br>Cannot be flushed............................3
**R01d**	***Please observe:*** only if the latrine is made by slab that can be cleaned (made of concrete/china)		Sanitary latrine (low pan) has curved piped outlet (broken) .. 1<br>Sanitary latrine (low pan) has curved piped outlet (not broken/in a good condition) 2

Household Number: | | | | |

Question number	Question	Response	Response Code
**R01e**	Do you share this latrine with other household		Yes ....1<br>No..2
**R01f**	If yes, how many households use it?		No. of hh…..
**R01g**	Is there any member/members in the hh who very rarely or does/do not use the latrine?		Yes ....1<br>No..2
**R01h**	If yes, who are they?	**MID** **MID** **MID**	Write MID
**R01i**	Where did your child whose age is less than 3 years defecate the last time?		Open field/space ....1<br>Inside home ....2<br>Inside the latrine used by the hh ......3<br>Latrine of some other hh ....4<br>Not applicable ....5>R01K
**R01j**	Where was the waste of your less than 3 year old child's last defecation thrown?		In a definite place of a field where we usually throw other hh waste…1<br>Latrine that we usually use…..2<br>Some other place……3
	Facilities of washing hands		
**R01k**	**Please Observe**: Is there any arrangement for hand wash near the latrine? (cannot be any further than 10 steps from the latrine)		Yes ....1<br>No..2
**R01L**	I can see that there is/ is not an arrangement to wash hands near the latrine. Is there any other hand wash area inside the household?		Dining area ....1<br>Kitchen ....2<br>Others ....3<br>No definite place for handwash……………..9
**RO1m**	**Please Observe:** Is there water in that hand wash area?		Yes ....1<br>No..2
**RO1n**	**Please Observe:** Is there any cleansing agent at the hand wash area?		Yes ....1<br>No..2

Household Number: | | | |

Question number	Question	Response	Response Code
**RO1o**	If yes, then list all the items that are there in the hand wash area.	☐	Soap..1<br>Liquid soap..2<br>Detergent powder ....3<br>Ash/earth ....4<br>Others 5
**R02**	Does the household have access to a water supply?	☐	Yes ....1<br>No...2
R02a	Where is that water source located?	☐	In own dwelling……………………1<br>In own yard/plot ....2<br>Elsewhere…………………………3
**R03**	What is the source of water used for other purposes than drinking, cooking, taking a shower?	☐	Supply Water (piped)<br>inside house.......................................1<br>Supply Water (piped), outside.........2<br>Own tube well ....3<br>Community tubewell ....4<br>Rain water ....5<br>Ring Well/ Indara ....6<br>Pond/River/ Canal ....7<br>Bottled water ....8<br>Shallow tubewell for irrigation........9<br>Deep tubewell for irrigation ....10<br>Other (specify)................................ 11<br>Other tubewell 12
**R04**	Is the source of drinking water same as the source of water used for other purposes?	☐	Yes .... 1>>R11<br>No.. 2
**R05**	Source of drinking water	☐	Supply Water (piped)<br>inside house.......................................1<br>Supply Water (piped), outside.........2<br>Own tube well ....3<br>Community tubewell ....4<br>Rain water ....5<br>Ring Well/ Indara ....6<br>Pond/River/ Canal ....7<br>Bottled water ....8<br>Shallow tubewell for irrigation........9<br>Deep tubewell for irrigation ....10<br>Other (specify).................................11<br>Other tubewell 12

Household Number: [ ][ ][ ][ ]

Question number	Question	Response	Response Code
**R11**	What is the distance of the main source of drinking water from the household?	[ ]	Meter
R11a	How long does it take to travel to the source of water, collect water and return to the household?	[ ]	Less than 5 minutes ....1<br>6-15 minutes...................................2<br>16-30 minutes.................................3<br>31-60 minutes.................................4<br>More than 1 hour 5
R11b	How many times in a day do you collect drinking water?	[ ]	Number of times
R11c	Will you please show me the container in which you preserve your water?	[ ]	Clay container ....1<br>Aluminium/metal container.............2<br>Steel container................................3<br>Other non-metallic contain(large) ..4<br>Plastic container ....5<br>Container with a plastic handle and a lid that can be used to close the top ....6<br>Glass bottle.....................................7<br>Other bottles...................................8<br>Other(specify) 9
R11c2	Is water available from this source all year round?	[ ]	Yes ....1<br>No...2<br>Don't know.....................................8

Household Number: | | | |

Question number	Question	Response	Response Code
R11c3	In the past two weeks, was water available every day from this source?		Yes ....1<br>No..2<br>Don't know.....................................8
R11d	Is there a lid for the container?		Yes ....1<br>No..2
R11e	Is there a piped outlet from the container in which you preserve water?		Yes ....1<br>No..2
R11f	If not, how do you obtain water from the container?		Tilt the container and pour the water out 1<br>Immerse another container to get the water out of the water container................2<br>Obtain water from the holder without immersing Your hands into it............................3

Household Number: | | | |

Question number	Question	Response	Response Code
**R06a**	In the past 24 hours, what steps have you taken to purify your water?		Filtered ....1<br>Boiled ....2<br>Boiled and filtered ....3<br>Chemically treated (iodine/chlorine) 4<br>Iodine/chlorine/chemically treated ..5<br>UV treated ....6<br>Filtered using cloth and sand...........7<br>Filtered using commercial filter ......8<br>Boiled and filtered using cloth ....9<br>Boiled and filtered using commercial filter 10<br>Not purified ....11<br>Straight from source ....12<br>Others (specify) 13
**R07**	If tubewell is used for drinking water, has the water been tested for arsenic contamination?		Yes ....1<br>No..2>>R9a<br>Don't know.....................................3>> R9a
**R08**	If yes, what color has the tubewell been marked?		Red ....1<br>Green...2>> R9a<br>None ....3>> R9a<br>Don't know.....................................4>> R9a
**R09**	If it has been colored red, do you still use it for drinking purposes?		Yes .... 1<br>No...2
**R09a**	Is the source of drinking water the same throughout the year?		Yes .... 1<br>No... 2
**R09b**	If no, where else do you get your water from and during which months?<br><br>For months write January …..1, February…..2, …….December….12	Other source | Month 1 | Month 2 | Month 3	Supply Water (piped)<br>inside house ....1<br>Supply Water (piped), outside.........2<br>Own tube well ....3<br>Community tubewell ....4<br>Rain water ....5<br>Ring Well/ Indara ....6<br>Pond/River/ Canal ....7<br>Bottled water ....8<br>Shallow tubewell for irrigation........9<br>Deep tubewell for irrigation ....10<br>Other (specify)................................11<br>Other tubewell 12

Household Number: | | | | |

Question number	Question	Response	Response Code
**R12**	What is the primary source of water for cooking?		Supply Water (piped) inside house ....1<br>Supply Water (piped), outside ....2<br>Own tube well ....3<br>Community tubewell ....4<br>Rain water ....5<br>Ring Well/ Indara ....6<br>Pond/River/ Canal ....7<br>Bottled water ....8<br>Shallow tubewell for irrigation........9<br>Deep tubewell for irrigation ....10<br>Other (specify)................................11<br>Other tubewell 12
**R13**	What is the primary source of water for washing clothes?		Supply Water (piped) inside house ....1<br>Supply Water (piped), outside ....2<br>Own tube well ....3<br>Community tubewell ....4<br>Rain water ....5<br>Ring Well/ Indara ....6<br>Pond/River/ Canal ....7<br>Bottled water ....8<br>Shallow tubewell for irrigation........9<br>Deep tubewell for irrigation ....10<br>Other (specify)................................11<br>Other tubewell 12
**R10**	How do you dispose garbage?		Local authority collects ....1<br>Private firm collects ....2<br>Public garbage pit/hole....................3<br>Own garbage pit/hole ....4<br>Burnt/buried ....5<br>Own garbage heap (not pit) ....6<br>Gather in open place........................7<br>Throw in pond/khaal/beel................8<br>Other (specify)................................9

Module End Time: | Hour | | | Minute | | |

Household Number: | | | | |

Module R2: Domestic animals and poultry habitation and hygiene management (Male)

Module start time: Hour | | | Minu | | | Respondent ID: | | Consent : | | Yes ...1 No.....2

Question number	Question	Response	Response Code
R2_01	If you have any children under the age of 5, have you ever seen them putting dirt/soil in their mouth?		Yes, multiple occasions………1<br>Yes, at least one occasion…..2<br>Never………………………………….3
R2_02	If a child consumes soil, do you think this is..		Healthy…………………………….…1<br>Harmless…………………………2<br>Harmful……………………………..3<br>Don't know………………………..4
R2_03	Are any livestock or pets kept inside the main household dwelling overnight in the last one year?		Yes....................................... 1<br>No.. 2>>R2_08
R2_04	Which animals are kept inside the main household dwelling overnight?<br><br>[Multiple response allowed]<br><br>Enumerator : Ask about the remaining domestic animals or pets once the respondant has answered the question		Chicken................. 1<br>Duck...................... 2<br>Sheep.................... 3<br>Goats.................... 4<br>Cattle.................... 5<br>Dogs...................... 6<br>Others................... 7<br>None......................8
R2_05	Are any of these animals kept overnight in the same room/area where your children sleep?		Yes................................. 1<br>No.................................. 2<br>No children.................... 3

Household Number: | | | |

R2_06	Which of these animals leave droppings in the household?<br><br>[Multiple response allowed]		Chicken.................1<br>Duck......................2<br>Sheep.................... 3<br>Goats.................... 4<br>Cattle.................... 5<br>Dogs......................6<br>Others.................. 7<br>None......................8
R2_07	How often do you remove/discard animal droppings?		Daily ....1<br>Several times a week .... 2<br>Occasionally.........................3<br>Never................................. 4
R2_08	If you have a child under 5, do they often come into direct physical contact with these animals?<br><br>[Multiple response allowed]		Chicken.................1<br>Duck......................2<br>Sheep.................... 3<br>Goats.................... 4<br>Cattle.................... 5<br>Dogs......................6<br>Others.................. 7<br>None......................8

Household Number: | | | |

Enumerator Observes:			
R2_09	**[OBSERVATION]: Can we observe poultry feces (chicken, ducks, bird, etc.) Around the house or in the compound?**		Yes... 1<br>No.. 2<br>Cannot observe...................... 88
R2_10	**[OBSERVATION]: Can we observe other animal feces (dog, cat, cattles, etc.) Around the house or in the compound?**		Yes... 1<br>No.. 2<br>Cannot observe...................... 88
R2_11	**[OBSERVATION]: Can you observe other garbage lying around in the house or compound?**		Yes... 1<br>No.. 2<br>Cannot observe………………..88

Module End Time: | Hour | | | Minute | | |

Household Number: | | | | |

Module S: Access to Facilities (Male)

Module Start Time: Hour | | | Minute | | | **Respondant ID:** | | Respondant's Consent: | | Yes ......1 No 2

Code	List of Facilities	Does any household member visit this facility regularly?<br><br>Yes ....1<br>No ....2>> Next row	What mode of transportation do you normally use to get to the closest facilities?<br>(multiple responses possible- list 3)<br>Foot..................................1<br>Bicycle.............................2<br>Rickshaw/Van...................3<br>Bullock cart.......................4<br>Boat..................................5<br>Engine boat.......................6<br>Motorcycle........................7<br>Tempo/Baby taxi/Nosimon 8<br>Bus....................................9<br>Train ....10<br>Other 11			Distance	How long does it normally take to get from your house to the closest facility?	
**Code**	**Facilities**	Code ↑	Code ↑			Km	**Hour**	**Minutes**
**Code**	**S_01**	**S_02**	**S03_a**	**S03_b**	**S03_c**	**S_04**	**S_05**	**S_06**
**01**	Health center/hospital							
**20**	Community Clinic							
**02**	Bus stop							
**03**	Main road							
**04**	Railway station							
**05**	Local shop/shops							
**06**	Weekly/periodic bazaar							
**07**	Nearest town							
**09**	Agricultural office							
**10**	Post office							
**11**	Bank							
**12**	BRAC							

Household Number: | | | |

**13**	Grameen Bank							
**14**	ASA							
**15**	Other NGO							
**16**	Internet access							
**17**	Kindergarten School							
**18**	Primary school for girls							
**19**	Primary school for boys							
**21**	Primary school (boys and girls)							
**22**	Secondary School (girls)							
**23**	Secondary school (boys)							
**24**	Secondary school (boys and girls)							
**8**	College							
**25**	Madrasa (female)							
**26**	Madrasa (male)							
**27**	Madrasa (both)							
**28**	Seed dealer							
**29**	Fertilizer dealer							
**30**	Pesticide dealer							

Module End Time: | Hour | | | Minute | | |

Household Number: | | | |

Module T: Economic Events/Shocks (Male)

Module T1b: Negative Shocks (Male)

Module start time: Hour | | | Minu | | Respondent ID: | | Consent : | | Yes ...1 No.....2

Recall period: Since the midline survey / For GFSS households, since 2015 & in the last 12 months

For the specified recall period, record information on negative shocks faced by the household.

Shocks (unexpected events)	Shock Code	Since midline survey/ For GFSS households, since 2015, did the event occur?<br><br>Yes.....1<br>No......2 >> Next row	In the past 12 months, did the event occur?<br><br>Yes.....1<br>No......2 >> Next row	How severe was the impact of this event on your household's economic condition?<br><br>Not Severe................ 1<br>Somewhat severe...... 2<br>Severe....................... 3<br>Extremely Severe ..... 4<br>Refused 7	How severe was the impact of this event on your household's food consumption?<br><br>Not Severe .... 1<br>Somewhat severe ...... 2<br>Severe .... 3<br>Extremely Severe...... 4<br>Refused..................... 7	What did you do to cope with this shock?<br><br>(bring up to 3 coping strategies)		
Shock	Code			Code ↑	Code ↑	Code 1		
T1b_01		T1b_02	T1b_03	T1b_04	T1b_05	T1b_06a	T1b_06b	T1b_06c
Did your household face difficult times as a result of having too much rain?	41							
Did your household face difficult times as a result of having too little rain?	42							
Did your household face difficult times as a result of land erosion?	43							
Did your household face difficult times as a result of having too much rain?	44							
Did your household face difficult times as a result of food price inflation?	45							
Did your household face difficult times as a result of someone stealing or destroying household member's belongings?	46							
Did your household face difficult times as a result of not being able to access inputs for your	47							
Did your household face difficult times as a result of disease affecting your crops ?	102							
Did your household face difficult times as a result of pest infesting your crops ?	103							
Did your household face difficult times as a result of theft of your household's crops ?	48							
Did your household face difficult times as a result of not being able to access inputs for your [illegible]	49							
Did your household face difficult times as a result of disease affecting your livestock ?	50							
Did your household face difficult times as a result of theft of your household's livestock?	13							
Did your household face difficult times as a result of not being able to sell the crops, livestock or other products of your household at a fair price?	51							
Is/Has anyone in your household experiencing/experienced a severe illness?	52							

Household Number: | | | | |

Shocks (unexpected events)	Shock Code	Since midline survey/ For GFSS households, since 2015, did the event occur?<br><br>Yes.....1<br>No......2 >> Next row	In the past 12 months, did the event occur?<br><br>Yes.....1<br>No......2 >> Next row	How severe was the impact of this event on your household's economic condition?<br><br>Not Severe................ 1<br>Somewhat severe...... 2<br>Severe....................... 3<br>Extremely Severe ..... 4<br>Refused 7	How severe was the impact of this event on your household's food consumption?<br><br>Not Severe .... 1<br>Somewhat severe ...... 2<br>Severe .... 3<br>Extremely Severe...... 4<br>Refused..................... 7	What did you do to cope with this shock?<br><br>(bring up to 3 coping strategies)		
Shock	Code			Code ↑	Code ↑	Code 1		
T1b_01		T1b_02	T1b_03	T1b_04	T1b_05	T1b_06a	T1b_06b	T1b_06c
Did your household have to bear medical expenses due to illness or injury of a household [illegible]	4							
Did your household experience loss of income due to illness or injury of a household member?	3							
Has your household experienced the death of a family member?	2							
Has your household experienced the death of the main earner of the family?	1							
Has anyone in your household experienced loss of a regular job?	5							
Did you lose your home due to a river erosion?	6							
Did you lose your home (due to any other reason besides river erosion)?	7							
Did anyone in your household become divorced/seperated?	8							
Did your household lose crops due to floods?	9							
Did your household lose crops due to a cyclone?	101							
Did your household lose crops due to any other reasons, besides flood/cyclone? (example: hailstorm, drought, etc.)	104							
Did your household lose livestock due to floods?	11							
Did your household lose livestock due to cyclone?	111							
Did your household lose livestock due to death of livestock?	12							
Did your household lose productive assets due to floods?	14							
Did your household lose productive assets due to destruction in fire?	151							
Aside from cyclone, did your household lose productive assets for other reasons (such as theft, river erosion)?	152							
Did your household lose consumption assets due to floods?	16							

Household Number: | | | |

Shocks (unexpected events)	Shock Code	Since midline survey/ For GFSS households, since 2015, did the event occur?<br><br>Yes.....1<br>No......2 >> Next row	In the past 12 months, did the event occur?<br><br>Yes.....1<br>No......2 >> Next row	How severe was the impact of this event on your household's economic condition?<br><br>Not Severe................ 1<br>Somewhat severe...... 2<br>Severe...................... 3<br>Extremely Severe ..... 4<br>Refused 7	How severe was the impact of this event on your household's food consumption?<br><br>Not Severe .... 1<br>Somewhat severe ...... 2<br>Severe .... 3<br>Extremely Severe...... 4<br>Refused..................... 7	What did you do to cope with this shock?<br><br>(bring up to 3 coping strategies)		
Shock	Code			Code ↑	Code ↑	Code 1		
T1b_01		T1b_02	T1b_03	T1b_04	T1b_05	T1b_06a	T1b_06b	T1b_06c
Did your household have to pay dowry?	18							
Did your household have to spend on other costs for a wedding?	19							
Did your household face division of father's property?	20							
Did your household face failure or bankruptcy of business?	21							
Did your household experience extorion by mastans?	22							
Was any member of your household imprisoned by the police?	23							
Was any member of your household arrested by the police?	24							
Did any one in your household have to pay a big bribe?	25							
Did your household have to cover the cost for the court case of a member?	26							
Did you experience any loss due to the court case?	27							
Did your household cover the reparations for victim of crime committed by a household [illegible]	28							
Was your household negatively affected by long duration of hartals/strikes/political unrest?	29							
Did your household experience cut-off or decrease of regular remittances to household?	30							
Did your household experience the withdrawal of NGO assistance?	31							
Did your household experience cut-off of benefits from a social safety program?	52							
Other (please specify) - 1	34							
Other (please specify) - 2	35							

Household Number: | | | | |

Code list for Module T1:

**Code 1: Coping strategies**		
None.. 1	Ate less food to reduce expenses........................ 10	Emergency receipt of remittance from migrant family member. 20
Sold land (specify homestead or agricultural) 2	Ate lower quality food to reduce expenses............ 11	Forced to change occupation.................... 21
Mortgaged/leased land (specify homestead or agricultural) 3	Took children out of school 12	Moved to less expensive housing.................. 22
Sold productive asset (specify)....................... 4	Transferred children to less expensive school 13	Sent non-working household member to work.............. 23
Mortgaged productive asset (specify)................... 5	Adult household member took job elsewhere temporarily 14	Took help from others.......................... 24
Sold consumption asset (specify) 6	Sent household member away permanently 15	Other (specify) 25
Mortgaged consumption asset (specify) 7	Sent children to be fostered by relatives.............. 16	
Took loan from NGO/institution 8	Sent children into domestic service.................. 17	
Took loan from mahajan/non-institutional source 9	Sent children to work somewhere other than domestic service . 18	
	Sent wife and children to his parental home............ 19	

Module T1c: Severe Disaster (Male)

Serial no.	Question	Response	Response Code
T1c_01	What has been the biggest disaster you faced in last 12 months that has affected your household most severely?		Insert code from T1b_01<br><br>Not Applicable...... 98
T1c_02	What has been the biggest disaster you faced in last 5 years that has affected your household most severely?		

Module T1c: Insurance (Male)

Serial no.	Question	Response	Response Code
T1c_03	Do you or any of your hh member have any insurance of any kind?		Yes.........................1<br>No..........................2
T1c_04	If yes, what type of insurance?		Life insurance………...1<br>Medical insurance…….2<br>Crop insurance………..3<br>Property insurance…….4<br>Other (please specify)…5

Household Number: | | | |

Module T2: Positive Economic Events (Male)

Recall period: Since midline. Now ask about any positive events that benefited the household financially.

Events	Event Code	Did the household experience any positive events last since midline?<br><br>Yes.........1<br>No..........2 → NEXT EVENT	The last time it happened, what year did it happen?<br><br>(year)			Rank the most important positive events<br><br>Most important......1<br>2nd most important.2<br>3rd most important.3
			What year did it happen?		What was the price of the acquired item (TK)?	
			Month	**Year**		
**T2_01**	**T2_02**	**T2_03**	**T2_04**	**T2_05**	**T2_06**	**T2_07**
New regular job for household member	**01**					
New or increased remittances	**02**					
Inheritance	**03**					
Large gift/lottery winnings	**04**					
Receipt of dowry	**05**					
Gain from business activities, specify	**06**					
Scholarship for child's education	**07**					
New NGO IGA starts	**08**					
Primary Education Stipend (100 taka)	**09**					
Secondary school stipend	**10**					
Other 1 (specify)	**11**					
Other 2 (specify)	**12**					
Other 3 (specify)	**13**					

Module End Time: | Hour | | | Minute | | |

Household Number: | | | |

Module U: Participation in Social Safety Net Programs (Male)

Collect data for last one year. Applicable for all household members. In case of participation in multiple programs report MID of all participants.

Module start time: Hour | | | Minu | | | Respondent ID: | | Consent : | | Yes ...1 No.....2

Sl. No	Description	Has s/he got any assistance? Yes ....1 No ....2 >> next row	Member ID		Cash	Rice		Wheat		Other food	Other in-kind		
											Subsidy Code	Number	Price
		Code ↑			Tk	Kg	Value (Tk/kg)	Kg	Value (Tk/kg)	Value (Tk)	Code1	Number	Value (Tk)
**SLNO**		**U01**	**MID1**	**MID2**	**U02**	**U03**	**U04**	**U05**	**U06**	**U07**	**U09**	**U010**	**U08**
**01**	Ananda School												
**02**	Stipend for Primary Students												
**03**	School Feeding Program												
**04**	Stipend for Dropout Students												
**05**	Stipend for Secondary and Higher Secondary/Female Student												
**06**	Stipend for Poor Boys in secondary school												
**07**	Stipend for Disabled Students												
**08**	Old Age Allowance												
**09**	Allowances for Distressed Cultural Personalities/ Activists												
**10**	Allowances for beneficiaries in Ctg. Hill Tract area.												
**11**	Allowances for the Widowed, Deserted and Destitute Women												
**12**	Allowances for the Financially Insolvent Disabled												
**13**	Maternity allowance program for the Poor Lactating Mothers												
**14**	Maternal Health Voucher Scheme												
**47**	Improving Maternal and Child Nutrition (IMCN)												
**15**	Honorarium for Insolvent Freedom Fighters												
**16**	Honorarium for Injured Freedom Fighters												
**49**	Ration Program for Martyr Family and Wounded Freedom Fighters												
**17**	Gratuitous Relief (Cash)												
**18**	Gratuitous Relief (GR)- Food												
**19**	General Relief Activities												
**20**	Cash For Work												
**21**	Agriculture Rehabilitation												
**22**	Subsidy for Open Market Sales												
**23**	Vulnerable Group Development (VGD)												

Household Number: [ ] [ ] [ ] [ ]

Sl. No	Description	Has s/he got any assistance?<br>Yes ....1<br>No ....2 >> next row	Member ID		Cash	Rice		Wheat		Other food	Other in-kind		
											Subsidy Code	Number	Price
**24**	VGD-UP (8 District on Monga Area)												
**25**	Vulnerable Group Feeding (VGF)												
**261**	Test Relief (TR) Food												
**262**	Test Relief (TR) Cash												
**27**	Food Assistance in CTG-Hill tracts Area												
**28**	Food For Work (FFW)												
**29**	Special fund for Employment Generation for Hard-core Poor in SIDR Area												
**30**	Fund for the Welfare of Acid Burnt and Disables												
**31**	100 days Employment Scheme / Employment Generation Program for the Poorest (EGPP)												
**32**	Rural Employment Opportunities for Protection of Public Property (REOPA)												
**33**	Rural Employment and Rural Maintenance Program (RERMP)												
**34**	Community Nutrition Program												
**35**	Char Livelihood Program (CLP)												
**36**	Shouhardo Program (CARE)												
**46**	Nabajibon Program (Save the Children)												
**45**	Proshar Program (ACDI VOCA)												
**37**	Accommodation (Poverty Alleviation & Rehabilitation) Project (Chief Advisors Office)												
**38**	Housing Support												
**39**	TUP (BRAC)												
**40**	One House one farm												
**43**	TMRI												
**48**	Pension program for retired government employees and their families												
**50**	Program for improving the living standards of tea garden workers												
**51**	Climate Rehabilitation Program (Gucchograam)												
**52**	Social Security Policy Support (SSSS) Program												
**44**	Other (please specify)												

Module End Time: Hour [ ] [ ] Minute [ ] [ ]

Household Number: | | | | |

Code1:Subsidy Code

Cow 1	Latrine..4	Others1 7
Goat.. 2	Tin 5	Others2 8
Chicken 3	Rickshaw/Cart 6	Others3 9
		Did not receive any in-kind goods..................................10

Module Ua: Program Participation in Food Friendly Program (Khaddo Bandhob / TK 10/kg Rice Program) (Male)

Module start time: Hour | | | Minu | | | Respondent ID: | | Consent : | | Yes ...1 No.....2

Question Number	Questions	Response	Code
**Ua_01**	Do you know about Khaddo Bandhob / TK 10 per kg program rice program?		Yes .... 1<br>No.. 2>> **Ua_03**
**Ua_02**	How did you know about the program?		From UP Chairman…………………………………………………..1<br>From UP member………………………………………….….…..2<br>From NGO worker…………………………………………...….3<br>From school teacher………………………………...…...……4<br>Courtyard meetings/ from radio………………………………….…5<br>From TV………………………………………………………………6<br>News papers…………………………………………………….7<br>From friends/neighbors……………………………………………8<br>Village Campaign……………………………………………….9<br>Sms from government…………………………………………….10<br>Recorded calls from GOB/NGO…………………………………..11<br>SAAO…………………………………………………………..12<br>Village Police…………………………………………………..13<br>Other (specify)……………………………………………………14
**Ua_03**	Are you/have you been a beneficiary?		Yes .... 1<br>No.. 2>> **Next Module**

Household Number: | | | | |

Question Number	Questions	Response	Code
**Ua_04**	How did you get selected into the program?		Did not apply but UP selected me……………………………………1<br>Did not apply but NGO selected me…………………………………2<br>I applied, and UP selected me ....…………………………………3<br>I applied, and NGO selected me………………………………......4<br>Does not know who selected…………………………………………5<br>Other member of the program supported me…………………………6<br>Paid money for getting selected………………………………………7<br>Husband/other family members assisted……………………………..8<br>I pursued………………………………………………………………9<br>Other (please specify)……………………………………………….10
**Ua_05**	Do you know in which months of the year the program is effective?		Yes .... 1<br>No.. ………………......2
**Ua_06**	If yes, state the months.		January…………………………………………………1<br>February………………………………………………..2<br>……………………………………………………..……..<br>December………………………………………………12
**Ua_07**	***Enumerator**: Verify the answer is correct [September, October, November, March, April]*<br>*Note: Verify if answer is different from the correct months*		Correct…………………………………………………1<br>Incorrect………………………………………………...2
**Ua_08**	When did you get enlisted in the program (month and year)?	Year Month	
**Ua_09**	When did you first avail this program (month and year)?	Year Month	

Household Number: | | | |

Question Number	Questions	Response	Code
**Ua_10**	Do you know the maximum limit on the amount of rice you can buy per month?		Yes .... 1<br>No... ………………...2
**Ua_11**	If yes, state the maximum limit on the amount of rice you can buy per month.		Kilograms/Month
**Ua_12**	Where do you buy the rice from?		Ration dealer……………………………………………………….1<br>Upazila Office…………………………………………………...2<br>UP office…………………………………………………………...3<br>School……………………………………………………………….4<br>NGO office……………………………………………………….5<br>Any other fixed place (specify)………………………………….6<br>Other (specify)…………………………………………………..7
**Ua_13**	Did you purchase rice in the months of March/April 2018?		Yes .... 1<br>No... …..……………...2 >> Ua_17
**Ua_14**	How much rice did you buy in this period?		Kilograms
**Ua_15**	How much did you pay per kilogram for the rice you bought?		TK/kg
**Ua_16**	How many times did you buy rice in that period?		Number of times
**Ua_17**	Did you purchase rice in the months of September-November 2018?		Yes .... 1<br>No... …..……………...2 >> Ua_21
**Ua_18**	How much rice did you buy in this period?		Kilograms
**Ua_19**	How much did you pay per kilogram for the rice you bought?		TK/kg
**Ua_20**	How many times did you buy rice in that period?		Number of times

Household Number:

Question Number	Questions	Response	Code
**Ua_21**	What was the quality of rice you bought?		Very Good……………………………………………………………1<br>Good…………………………………………………………………..2<br>Not so good………………………………………………………......3<br>Bad……………………………………………………………………4<br>Very bad………………………………………………………………5<br>Not suitable for human consumption…………………………………6
**Ua_22**	If answer is 4 to 6, what were the problems?		Had dust/other particles………………………………………………1<br>Pest infested…………………………………………………………..2<br>Bad smell……………………………………………………………..3<br>Other (specify)………………………………………………………..4
**Ua_23**	What type pf problems did you face when you went to purchase the rice?		Had to wait in the line for a long time………………………………1<br>Distance from home to distribution center is very far………………2<br>Had to pay bride the seller...……………………………………….....3<br>There were no problems……………………………………………4<br>Other (please specify)………………………………………………5
**Ua_24**	Did you sell any portion of the rice?		Yes .... 1<br>No…………………………………….………………......2
**Ua_25**	If yes, how much rice did you sell?		Kilograms
**Ua_26**	What was the selling price per kilogram?		Taka/kg
**Ua_27**	***Enumerator****: Ask the beneficiary to show her/his card for the program. Record outcome.*		Showed her/his card…………………………………………………1<br>Could not show…………………………………………………...2

Household Number: | | | |

Question Number	Questions	Response	Code
**Ua_28**	***Enumerator:*** *If she/he could not show the card, ask why.*		Did not receive card yet……………………………………………….1<br>Sold the card to somebody else……………………………………….2<br>UP chairman/member kept it in their custody………………………...3<br>NGO kept it…………………………………………………………...4<br>Lost the card/cannot find it……………………………………………5<br>Other (specify)………………………………………………………...6

Module End Time: | Hour | | | Minute | | |

Household Number: | | | |

Module Start Time: Hour | | | Minute | | | **Respondant ID:** | | Respondant's Consent**:** | | Yes...... 1 No....... 2

Module V: Migration, Remittances, Transfers and Other Income (Male)
Module V1: Profile of Current Migrants (Domestic and International) (Male)

**V1_01**	Has anyone, who was a member of your household in the last midline survey/ 2015, currently a migrant (living away for 6 months or more) within the country (but not in same upazilla) or abroad?		Yes ..... 1<br>No .... 2>>V2_01

Report for all current migrants of the household. Use separate rows for each person.

Person ID	Was a member of the household in midline	Name	Relation to household head	When did (name) migrate?		Age	Sex<br><br>Male …1<br>Female.2	Education	Occupation<br><br>Use Code 3 from Module C	Which country is s/he in now?<br><br>Within Bangladesh…1<br>Abroad…2>>V1_11	If in-country, write zila code.	If abroad, write country code.	Who helped in the migration process?<br>If response is 1 or 4>>V1_12	If Code8 is 2 or 3, which division is this agent located?	For what purpose did the member initially migrate?	How was the migration expenses paid?	Does the migrant send remittance home on a regularly (at least once in the last 12 months)?<br><br>Yes…1<br>No….2
	Member ID		Code 1	Year	Month	Complete year	Code ↑	Code 2	Code 3 from Module C	Code ↑	Code 4	Code 5	Code 8	Code 9	Code 6	Code 7	Code ↑
**PID**	**MID**	**Name**	**V1_02**	**V1_0**	**V1_0**	**V1_05**	**V1_0**	**V1_0**	**V1_08**	**V1_09**	**V1_10**	**V1_11**	**V1_15**	**V1_16**	**V1_12**	**V1_13**	**V1_14**
81																	
82																	
83																	
84																	
85																	
86																	

Household Number: [ ] [ ] [ ] [ ]

Code 1: Relationship	Code 2:Education	Code 4: District /Zilla		
**Relationship with primary respondent**	Never attended school............99	Dhaka.........................1	Comilla........................25	Lalmonirhat.................49
Primary respondent................1	Reads in class I 0	Gazipur........................2	Feni 26	Nilphamari 50
Primary respondent Husband/wife 2	Completed class I....................1	Manikganj 3	Lakshimpur 27	Rangpur.......................51
Son/daughter..........................3	Completed class II 2	Munshiganj 4	Noakhali......................28	Dinajpur 52
Daughter/son -in-law 4	Completed class III.................3	Narayanganj 5	Khulna.........................29	Thakurgaon 53
Grandson/daughter.................5	Completed class IV................4	Narsingdi....................6	Jessore 30	Panchagarh..................54
Father/mother.........................6	Completed class V 5	Faridpur.......................7	Jhenaidah.....................31	Sylhet 55
Brother/sister 7	Completed class VI................6	Gopalganj 8	Magura 32	Hobiganj......................56
Niece/Nephew 8	Completed class VII 7	Madaripur....................9	Narail...........................33	Moulovibazar 57
Primary respondent's cousin...9	Completed class VIII 8	Rajbari........................10	Bagerhat 34	Sunamganj...................58
	Completed class IX.................9	Shariatpur....................11	Satkhira 35	Barishal 59
**Relationship with primary respondent's**	Completed Secondary School/Dakhil	Jamalpur......................12	Chuadanga...................36	Bhola 60
**husband/wife**	10	Sherpur........................13	Kushtia 37	Jhalakathi 61
Father-in-law/mother-in-law...10	Higher secondary (1st	Kishoreganj 14	Meherpur.....................38	Pirojpur 62
Brother/Sister-in-law 11	year)…………..11	Mymensing 15	Rajshahi.......................39	Barguna 63
Husband/wife's niece/nephew 12	Completed Higher Secondary/Alim 12	Netrokona...................16	Noagaon 40	Patuakhali....................64
Primary respondent's	Degree (1st	Tangail 17	Nawabganj 41	
husband/wife's cousin 13	year)………………….…13	Chittagong...................18	Natore..........................42	
	BA/BSC pass/Fazil 14	Cox's bazar 19	Pabna...........................43	
**Other relative/non relative**	BA/BSC honors/Fazil 15	Bandarban 20	Sirajganj 44	
Other relative 14	MA/MSC and above/Kamil....16	Khagrachhari...............21	Bogra...........................45	
Household help.......................15	SSC Candidate........................22	Rangamati 22	Joypurhat.....................46	
Other Non relative/friends 16	HSC Candidate 33	Brahmanbaria..............23	Gaibanda 47	
	Preschool class (general) 66	Chandpur.....................24	Kurigram 48	
	Preschool (mosque based) 67			
	Medical/MBBS 71			
	Nursing 72			
	Engineer.................................73			
	Diploma Engineer...................74			
	Vocational/Technical Education 75			
	Other (specify)........................76			

Household Number: | | | | |

Code 5: Country			Code 6: Primary reason for migration	Code 7: How was the migration expenses paid
Australia ....1<br>Bahrain ....2<br>Brunei ....3<br>Canada ....4<br>Cyprus...5<br>Federation of Russia ....6<br>France ....7<br>Germany ....8<br>Greece...9<br>India...10<br>Iran ....11<br>Iraq ....12<br>Italy ....13<br>Japan..14	Jordan..................15<br>Kuwait ....16<br>Laos ....17<br>Liberia.................18<br>Libya ....19<br>Malaysia..............20<br>Maldives ....21<br>Mauritius.............22<br>New Zealand.......23<br>Oman ....24<br>Pakistan ....25<br>Poland ....26<br>Qatar ....27<br>South Korea 28	Saudi Arabia....... 29<br>Singapore .... 30<br>South Africa ...... 31<br>Spain .... 32<br>Sweden............... 33<br>Switzerland .... 34<br>Taiwan .... 35<br>Turkey .... 36<br>U.S.A.................. 37<br>UAE .... 38<br>U.K..................... 39<br>Yemen .... 40<br>Other(specify) 41	Employment ....1<br>Education..........................2<br>Marriage ....3<br>Health care........................4<br>Medical Treatment............5<br>Escape war/ violence ....6<br>Drought/Famine/Disease/Etc. 7<br>Business/self-appointed work 8<br>Other (specify) 9	Migrating member paid all expenses........................... 1<br>All expenses were paid from common household resources.. 2<br>Received money from friends/relatives .... 3<br>Borrowed money from friends and relatives .... 4<br>Borrowed money from commercial lender.................. 5<br>Made arrangement with employment agency/foreign employer... 6<br>Mortgaged land .... 7<br>Sold own land or other assets .... 8<br>I do not know.. 9<br>Others (Specify) 10
**Code8**	**Code9**			
Friends/family in the migrated location 1<br>Agent in Bangladesh....................2<br>Both ....3<br>Others (specify............................4	Dhaka..................1<br>Chittagong ....2<br>Rajshahi ....3<br>Khulna ....4<br>Barisal.................5<br>Sylhet..................6<br>Rangpur 7			

Household Number: | | | | |

Module V2: Remittance in (Male)

**V2_01**	During the past 12 months, have you or any member of household received any money from any person who does not live in your household?		Yes.....................1>>V2_02<br>No.......................2>>V3_01

Report for remittances received from migrants of the household and any other remitter.

Use PID 81-89 for migrants who were household members in the last 5 years. Use PID 91-99 for remitters who were never household members.

Person ID	Was a member of the household in midline	What is the relationship of the remitter to the household head?	Where does the remitter currently live?		In the past 12 months			Which expenditure, saving, and investment, would have been **CUT** had the remittance from this source not been received?			Did the person who sent the money put any condition on items the money was to be spent on?<br><br>Yes…1<br>No….2>>V2_11	What items were these?			Who was the remittance sent to?
			District (if within Bangladesh)	Country (if outside Bangladesh)	How many times have you received remittance payments?	How much money in total did your household receive?	How was the remittance sent?<br><br>(Report primary method)								
	Member ID	Code 1	Code 4	Code 5	number	Tk	Code 8	Code11			Code	Code11			Code12
**PID**	**MID**	**V2_02**	**V2_03**	**V2_04**	**V2_05**	**V2_06**	**V2_07**	**V2_08a**	**V2_08b**	**V2_08c**	**V2_09**	**V2_10a**	**V2_10b**	**V2_10c**	**V2_11**

Household Number: [ ][ ][ ][ ]

Code 10: How was the remittance sent	Code 11: Applicable for V2_08 & V2_10	Code12 :Who was the remittance sent to
Personal delivery by family, friends ...... 1<br>Remittance company (i.e. Western Union) 2<br>Transfer to your own bank account .... 3<br>Transfer to someone else's bank account 4<br>Cheque / Bank draft .... 5<br>Money order .... 6<br>TT (telephonic/telegraphic transfer) ..... 7<br>Hundi/Hawala .... 8<br>Mobile Banking .... 10<br>Other (Specify) 9	Cash savings .... 1<br>Education .... 2<br>Health (Hospital/Doctor/Medicine) .... 3<br>Consumption (food, cloths) .... 4<br>Build house/land /renovate house .... 5<br>Purchase of vehicle .... 6<br>Purchase consumer durables .... 7<br>Investment in agr. or business.............. 8<br>Purchase of gold and other jewelry........ 9<br>Livestock purchase .... 10<br>Purchase shares/stock/bonds.................. 11<br>Other (Specify) 12	For female member ....1<br>For male member ....2<br>All members of the household...............3

SL	Questions	Response	Response Code		
V2_12	In the last 12 months, what was the remittance from abroad used for?<br><br>According to the amount of money spent, record the three primary expenses	[ ]<br>[ ]<br>[ ]	Not applicable............................ 1<br>Savings .... 2<br>Repay loan .... 3<br>Buying productive assets .... 4<br>Agricultural equipment purchase 5<br>Purchase land .... 6<br>Treatment.................................. 7<br>Household commodity purchase. 8<br>Education expenses.................... 9<br>Wedding expenses 10	Traveling abroad..............................11<br>Building/purchasing house ....12<br>Repair/develop house ....13<br>House rent..14<br>Purchase furniture............................15<br>Electronics ....16<br>Vehicle purchase..............................17<br>Giving loan ....18<br>Dowry ....19<br>Non-agricultural productive asset purchase...20	Charity(except Jakat) ....21<br>Jakat.......................................22<br>For religious institutions (mosque, madrasa, orphanage ....23<br>Investment in business institutions ....24<br>Primary education for male......25<br>Primary education for female ..26<br>Secondary education for male..27<br>Secondary education for female ....28<br>Others (specify) 29
V2_13	In the last 12 months, what purpose was the remittance within Bangladesh used for?<br><br>According to the amount of money spent, record the three primary expenses	[ ]<br>[ ]<br>[ ]			

Household Number: | | | | |

Module V3: Remittance out (Male)

Report for remittances received from migrants of the household and any other remitter

**V3_01**	During the past 12 months, did you or any member of your household send money to someone who does not live in your household?		Yes .... 1<br>No .... 2>>Next Module

Sl No.	What is the relationship of the recipient to the household head?	Where does the recipient currently live?		In the past 12 months		
		District (if within Bangladesh)	Country (if outside Bangladesh	How many times have you send remittance payments?	How much money in total did your household send?	How was the remittance sent? (Report primary method)
	Code 1	Code 4	Code 5	Number	Tk	Code 13
**Sl No.**	**V3_02**	**V3_03**	**V3_04**	**V3_05**	**V3_06**	**V3_07**

Code 13: How was the remittance sent?
Personal delivery by family, friends 1
Remittance company (i.e. Western Union) 2
Transfer to your own bank account..............................3
Transfer to someone else's bank account 4
Cheque / Bank draft 5
Money order...6
TT (telephonic/telegraphic transfer) 7
Hundi/Hawala 8
Mobile Banking 10
Other (specify) 9

Household Number: | | | | |

Module V4: Other Income household (Male)

Report for the last 12 months:

Question number	Question	Response	Response code
**V4_01**	Income received from land rent		Taka/year
**V4_02**	Income received from rent of other property		Taka/year
**V4_03**	Income received from life and non-life insurance		Taka/year
**V4_04**	Profits and dividends received as partner/share holder		Taka/year
**V4_05**	Gratuity, separation payment, retirement benefit received		Taka/year
**V4_06**	Lottery, prizes, and similar income received (in cash)		Taka/year
**V4_07**	Lottery, prizes, and similar income received (in kind)		Report imputed value
**V4_08**	Charity and other assistance (in cash)		Taka/year
**V4_09**	Charity and other assistance (in kind)		Report imputed value
**V4_10**	Interests received (from bank and other sources)		Taka/year
**V4_11**	Other cash receipts (not included elsewhere)		Taka/year
**V4_12**	Other in-kind receipts (not included elsewhere)		Report imputed value

Module End Time: | Hour | | | Minute | | |

Household Number: | | | | |

Module W: Anthropometry, Health and Illness (Female)

Module W1: Anthropometry (Female)

Measure for all household members ≥ 5 years.

Module start time: Hour | | | Minu | | | Respondent ID: | | Consent : | | Yes ...1 No.....2

	Member ID	Name	Are you pregnant? Yes 1 No 2	Are you lactating? Yes 1 No 2	Weight	Height	If not measured, why?	Blood pressure measurement for all members aged **12 years** and above	
			Code ↑	Code ↑	(kg)<br><br>(Mark height and weight '-' if not measured)	(cm)<br>(Mark height and weight '-' if not measured)	Have measured……………………..1<br>Absent……………………..……..2<br>Sick.......... ……………….……..3<br>Refused to give measurement…..….4<br>Other (specify)……………………..5	Systolic/ Upper (mmHg)	Diastolic/ Lower (mmHg)
	**MID**	**Name**	**W1_01**	**W1_02**	**W1_03**	**W1_04**	**W1_05**	**W1_34**	**W1_35**
**Women**									
**Men**									

Module W1: (Continues) ***Menstrual hygiene practices and knowledge among adolescent girls and women between the ages of 10 and 50 years***

Do you have your menstruation?<br><br>Yes .... 1<br>No .... 2<br>Never menstruated…3 >>skip to next module	What was your age during your first menstruation?<br>(If the response to W1_06 is 2, then record the age and skip to W1_14)<br><br>(Age when menstruation started)	What kind of sanitary napkin/pad do you use during menstruation?<br><br>(If response is 3, skip to W1_15; If response is 6, skip to W1_16)	How often to your purchase sanitary napkins/pads?<br><br>Every month…1<br>Buy together for couple of months…2<br>Not applicable ....3>> W1_15	How much do you spend on this product every month?<br><br>Tk	What is your relationship with the person who buys sanitary napkins/pads for you?<br><br>Self…1<br>Husband…2<br>Mother…3<br>Sister…4<br>Father…5<br>Brother…6<br>Other female…7<br>Other male…8	How many times do you change pads/cloth in one day on average?<br><br>Less than 2 times…1<br>2 to 3 times…2<br>More than 3 times…3	If the answer to W1_08 is 3 and above:<br><br>Why don't you use sanitary pad/tampon?	If use reusable cloth, what do you wash it with?<br><br>Only water…1<br>Soap and water…2<br>Not applicable…3>>W1_19	Where do you dry the cloth?<br><br>Indoors…1<br>Sunlight…2<br>Not applicabl…3	How do you dispose the sanitary napkin/pad/cotton/cloth etc.?	What type of problems did you face during menstruation?		For how many days do you have blood flow during menstruation?<br><br>Codes:<br>1 to 2 days…1<br>3 to 4 days…2<br>5 to 7 days…3<br>7 to 10 days…4<br>>10days…5
											Monsoon season	Dry season	
Code ↑	years	Code 1	Code ↑	Taka	Code ↑	Code ↑	Code 2	Code ↑	Code ↑	Code 3	Code 4		Code ↑
W1_06	W1_07	W1_08	W1_09	W1_10	W1_11	W1_15	W1_16	W1_17	W1_18	W1_19	W1_12	W1_13	W1_20

Module W1: (Continues) ***Menstrual hygiene practices and knowledge among adolescent girls and women between the ages of 10 and 50 years***

Quantity of blood flow:<br><br>Normal …1<br>Excessive …2<br>Scanty…3	During menstruation do you bathe daily?<br><br>Yes…1<br>No…2	What do you wash your hands with after cleaning yourself?<br><br>With water only…1<br>With soap and	Do you regularly clean your external genitalia?<br><br>Yes 1<br>No 2	What do you clean external genital with?<br><br>With water…1<br>With soap and water…2	Applicable for all responders:<br><br>Who answered on your behalf?<br><br>Self…1<br>Husband…2<br>Mother…3<br>Sister…4<br>Father…5<br>Brother…6			**Ask to all females less than 19 years old**						Applicable for all responders:<br><br>Who answered on your behalf?<br><br>Self…1<br>Husband…2<br>Mother…3<br>Sister…4<br>Father…5<br>Brother…6
						Did you know about menstruation prior to menarche?<br>Yes 1<br>No 2	Who first told you about menstruation?	Whom do you consult about menstrual hygiene?	What do you believe is the cause of menstruation?<br><br>Physiological …1<br>Result of sin from God…2<br>Due to some disease…3<br>Do not know …4<br>Other (please specify)…5	Do you face any of the following restrictions during menstruation (allow multiple):	Do you miss school often during menstruation?<br><br>Yes…1<br>No…2>W1_33<br>Not applicable…3>W1_14	If response to W1_31 is 1, reasons for missing school:	If answer to W1_31 is 2, is there a proper washroom/ toilet facility to change pad/cloth or clean yourself?<br><br>Yes 1<br>No 2	

Household Number: | | | | |

		water…<br>2			Other female…7<br>Other male…8											Other female…7<br>Other male…8
Code ↑	Code ↑	Code ↑	Code ↑	Code ↑	Code ↑	Code ↑	Code 5	Code 5	Code ↑	Code 6			Code ↑	Code 7	Code ↑	Code ↑
W1_21	W1_22	W1_23	W1_24	W1_25	W1_14a	W1_26	W1_27	W1_28	W1_29	W1_30 a	W1_30 b	W1_30 c	W1_31	W1_32	W1_33	W1_14b

**Code 1**	**Code 2**	**Code 3**	**Code 4**
Disposable commercially sold sanitary napkin/pad/tampons…1 >>W1_09<br>Disposable sanitary napkin/pad/tampons made from cotton/gauze/soft tissue…2 >>W1_09<br>Reusable cloth after boiling or washing…3<br>Old cloth thrown away after single use …4<br>Other…5<br>Nothing…6	Never heard of it…1<br>Expensive…2<br>No one is there to buy it for her…3<br>Difficult to discard…4<br>Don't feel comfortable…5	Throw away with general waste…1<br>Burning…2<br>Dispose by burying…3<br>Not applicable…4<br>Other (please specify)…5	Itching (irritating skin sensation causing a desire to scratch)/scabies…1<br>Itching inside the vaginal canal…2<br>Fever/ feel feverish…3<br>Lower abdominal pain…4<br>White discharge…5<br>Bad odor discharge…6<br>Irregular menstruation…7<br>Jock itch/ crotch itch/ crotch rot (tinea cruris): fungal infection of the groin and upper thighs…8<br>Sweaty crotch/damp groin…9<br>No problem…10<br>Breast pain…12<br>Other (specify)…11

**Code 5**	**Code 6**	**Code 7**
Mass media…1<br>Mother/older sister/elder female in the household…2<br>School teacher/curricula…3<br>Peers/friends…4<br>Other (books, health workers, relatives outside the household)…5	Visiting religious places/practicing prayers and rituals…1<br>Routine household work…2<br>Playing…3<br>Attending school…4<br>Going outside in general…5<br>Eating certain types of food…6<br>Separated within households…7<br>Do not face any restrictions…9	Feel uncomfortable at school around boys…1<br>Remain sick…2<br>Heavy bleeding…3<br>No place to change pads/cloth…4<br>Forbidden by guardian…5

Module End Time: | Hour | | | Minute | | |

Household Number: | | | |

Module W2: Anthropometry-Children (Female)
Measure for all children less than 60 months old.

Module start time: Hour | | | Minu | | | Respondent ID: | | Consent : | | Yes ...1 No.....2

Member ID	Name	Father's ID No.*	Mother's ID No.*	Birth order*	Child's date of birth								Weight (kg)	Height (cm)	How was this child's height measured?<br><br>Lying down…1<br>Standing up…2	Reason for not being measured?<br><br>Have measured…1<br>Absent…2<br>Sick…3<br>Refused to give measurement…4<br>Other (specify)…5	Is this child still breast fed?<br><br>Yes ......1<br>No 2
					Day	If does not know day, what week of the month?	Month	Year	Age (month)	Confirmed with birth certificate or health card?<br><br>Yes ......1<br>No 2	Child's weight during birth (kg)	How was the information regarding birth weight collected?<br><br>From Health Card…66<br>From mother/ other hh member…77<br>Was not weighted/ Cannot remember…99					
**MID**	**Name**	**W2_01a**	**W2_01**	**W2_13**	**W2_02**	**W2_03**	**W2_04**	**W2_05**	**W2_14**	**W2_06**	**W2_15**	**W2_16**	**W2_07**	**W2_08**	**W2_09**	**W2_10**	**W2_11**

*Note for W2_13 (birth order): Record according to the mother's pregnancy order. Do not record miscarriages that happen prior to the completion of the first 6 months, only record those that happen after 6 months (those will be considered still-birth).

*Note: If the child's mother is not a member of the household (for example, if she is dead or lives in another household), insert 55.

Household Number: | | | | |

Module W3: Health (Female)

Health status questions to be asked about all household members over the age of 15.

Respondent ID [ ]

Member ID	Name	Can this person do the following? Code 1			Does this person have difficulties with:			Does this personhave a missing or deformed limb?	Does this person suffer from any paralysis or have part of their body that has lost its sense of touch?
		Stand up after sitting down	Walk for 5km	Walk with ½ maund rice/wheat for 20m	Hearing? (Code 2)	Speaking? (Code 3)	Eyesight? (Code 4)	If yes, which one? (Code 5)	If yes, which one? (Code 5)
MID	Name	**W3_01**	**W3_02**	**W3_03**	**W3_04**	**W3_05**	**W3_06**	**W3_07**	**W3_08**

Code 1: Daily activity
Easily 1
With a little difficulty 2
With a lot of difficulty 3
Not at all 4

Code 2: Hearing
No problems 1
Sometimes has difficulty 2
Generally poor hearing 3
Deaf in one ear 4
Deaf in both ears 5

Code 3: Speech
No problems 1
Sometimes has difficulty 2
Generally has difficulty 3
Cannot speak at all 4

Code 4: Sight
No problems 1
Difficulties seeing things close 2
Difficulty in seeing things far away 3
Generally poor eyesight 4
Cannot see at night/sees with difficulty 5
Blind in one eye 6
Blind in both eyes 7

Code 5: Which one
No / none 1
Finger 2
Hand 3
Arm 4
Toes 5
Foot 6
Leg 7
Jaw 8
Back 9
From hips down 10
From neck down 11
Left side of body 12
Right side of body 13
Part of/ whole face 14
Whole body 15
Other: Specify 16

Household Number: | | | | |

Module W4: Illness (Female)

Questions to be asked to all household members.

Respondent ID

Member ID	Name	In the last four weeks, has this person suffered: (Write 77 if condition has persisted for more than four weeks)													
		Any illness or injury in the last four weeks? Yes....... 1 No....... 2 >> next row	How many days in total?	How many days has this person been unable to perform his/her main activity at all? (Applicable only for 15 years and above. If not applicable write 99)	Significant weight loss Yes ....1 No........2	Prolonged fever? Yes ....1 No ....2 >>W4_07	How many days in total?	Diarrhea Yes....... 1 No....... 2 >>W4_09	How many days in total?	Persistent cough? Yes....... 1 No....... 2 >> W4_11	How many days in total?	Generalized skin rash? Yes....... 1 No....... 2 >> W4_13	How many days in total?	Mouth or throat infection? Yes....... 1 No .... 2>>next row	How many days in total?
		Code ↑	Days	Days	Code ↑	Code ↑	Days	Code ↑	Days	Code ↑	Days	Code ↑	Days	Code ↑	Days
**MID**	**Name**	**W4_01**	**W4_02**	**W4_03**	**W4_04**	**W4_05**	**W4_06**	**W4_07**	**W4_08**	**W4_09**	**W4_10**	**W4_11**	**W4_12**	**W4_13**	**W4_14**

Household Number: | | | |

Module W5: Illnesses (Record for the last 4 weeks) (Female)

Respondent ID [ ]

Record information for any member of the household who suffered from any illness in the last 4 weeks. For every illness, fill out a new row.

ID	Name	What are you suffering from? Code 1			How long ago did you recover? (if still sick, insert 99)	For how many days did you suffer (days)	For how many days did you have to refrain from your regular work because of the illness
		W5_01a	W5_01b	W5_01c	W5_02	W5_03	W5_04

Illness Code- 1		
Fever 1	Asthma........................... 17	Others 33
Influenza 2	Other allergies................ 18	AIDS/HIV Positive……………………………………34
Pneumonia....................... 3	Arthritis/*Gout*................. 19	Stroke / paralysis ……………………………35
Diarrhea 4	TB 20	Back pain/injury ………………………..36
Dysentery 5	Rheumatic Fever 21	Kidney problem…………………………………..37
Cholera............................ 6	Diabetes 22	Amnesia…………………………38
Malaria 7	Anemia........................... 23	Did not suffer from any illness in the last four weeks................ 99
Typhoid........................... 8	High Blood Pressure 24	
Jaundice 9	Heart Disease 25	
Mumps 10	Cancer/Tumor 26	
Small Pox........................ 11	Headache.. 27	
Measles 12	Stomach Ache 28	
Tonsillitis 13	Eye Problem... 29	
Acidity 14	Ear Problem 30	
Ulcer 15	Dental Problem 31	
Skin Disease.................... 16	Illnesses pertaining exclusively to females 32	

Household Number: | | | |

Module W5a: Chronic Illness (Female)

Respondent ID

Note: Record information on chronic illnesses of all family members

ID	Name	Age	Chronic Disease: Have you ever been diagnosed with the following? Yes…1 No…2								
			Heart disease	Diabetes	Cancer	High Blood pressure	High Cholesterol	Stroke	Measles	Asthma	HIV / AIDS
**MID**	**Name**	**(Years)**	**W5_05**	**W5_06**	**W5_07**	**W5_08**	**W5_09**	**W5_10**	**W5_11**	**W5_12**	**W5_13**

Module End Time:

Hour			Minute		

Household Number: | | | | |

Module X: Household Food Consumption and Food Security (Female)
Module X-1.1: Household Food Consumption (Recall-1) (Female):
Recall: Last 24 Hours

Module start time: Hour | | | Minu | | | Respondent ID: | | Consent : | | Yes ...1 No.....2

Note:

First ask if yesterday was a special day, like a celebration or feast day or a fast day where anyone in the HH ate special foods or where they ate more or less than usual or did not eat because they were fasting?

X1_01. Was yesterday a special day when special kinds of foods were eaten? | | Yes .... 1 No.......... 2

If yesterday was **not** a special day, then ask the respondent about the types of foods that they or anyone else in their household ate yesterday during the day and at night, whether at home or outside the home.
If yesterday **was** a special day, then ask the respondent to describe the foods (meals and snacks) consumed the **day before yesterday (or the last normal day)** during the day and night, whether at home or outside the home.

(Gather information on last 24 hours family food consumption)
Quantity of family food consumption: Female member in-charge of cooking, supervising and serving to be interviewed.

Recall Date:

Day	Month	Year

Respondent ID: | |

Source of Food:<br>Left over from previous day........1<br>Food cooked in the HH ....2<br>Purchased..................................3<br>Food received from others...........4<br>Invitation...................................5<br>Food taken at employer's place...6	Time of day<br>Morning...... 1<br>Noon .... 2<br>Night........... 3<br>Snacks......... 4	Menu	Menu codes	Ingredients	Ingredients	Total raw weight of ingredients	Cooked weight
			Code		Code	(gram)	(gram)
**X1_02**	**X1_03**	**X1_04**	**X1_05**	**X1_06**	**X1_07**	**X1_08**	**X1_09**

Household Number: | | | | |

Module End Time: | Hour | | | Minute | | |

Module X-2.1: Intra-Household Food Distribution (Female)

Household Recall (Individual Level):

Module start time: | Hour | | | Minu | | | Respondent ID: | | Consent : | | Yes ...1 No.....2

(Gather information on last 24 hours family food consumption)
Quantity of family food consumption: Female member in-charge of cooking, supervising and serving to be interviewed. If anyone has not taken any meal, then put reason code in column X2_03

Mark tick for appropriate time

Morning	Noon	Night	Snacks

Member ID Or Guest/Given food to other/Left over for next day	Name	If meal not taken, then why not? (If Code is 2 to 9, then move to next MID)	If Guest			Menu	Menu codes	Cooked Weight	If curry, then weight of meat/fish in the curry	Where meal was taken	Order of taking meal
			Sex Male...... 1 Female...2	Age	Type of Guest						
Note*		Code 1 ↓	Code↑	(Years)	Code 2		Code	(Gram)	(Gram)	Code 3 ↓	
**X2_01**	**X2_02**	**X2_03**	**X2_04**	**X2_05**	**X2_06**	**X2_07**	**X2_08**	**X2_09**	**X2_10**	**X2_11**	**X2_12**

Note: Write member ID for each household member:
Begin guest code from.................101
Food Given to animal code..........201
Food Given to others code...........301
Food left over code......................401

Code 1: Cause of not taking meal
Yes, meal taken 1
Food was not available 2
Fasting...3
Sickness...4
Unwilling to take food.........................5
Currently staying away from HH 6
Not a valid HH member 7
Breastfed child 8
Others (specify)..................................9

Code 2: Type of guest code
Relatives from outside village.....1
Relatives from same village 2
Neighbors...................................3
Friends 4
Dignitary 5
Day labor working at house.........6
Day labor working at field...........7
Other 8

Code 3: Where meal was taken code
Respondent's home 1
Employer's house 2
Invitation....................................3
In market place/hotels 4
Absent..5
School gate…………..6
Local *haat/bazar* (weekly/daily market)……….7

Module End Time: | Hour | | | Minute | | |

Household Number: | | | | |

Module X2a: Consumption of Food Prepared Outside (Female)

Household Recall (Individual Level) Period: Last 24 Hours (Bring information for up to 5 people)

X2a_01: In the past 24 hours, did any household member consume food prepared outside of home?

Module start time: Hour | | | Minu | | | Respondent ID: | | Consent : | | Yes ...1 No.....2

Member ID of the person who has eaten food prepared outside of home	Food item prepared outside of home consumed in the last 24 hours	Why did you consume this food item?<br>I work outside of home....... 1<br>School Tiffin .... 2<br>There was no food in the house .... 3<br>I like the taste of this food.. 4<br>Ate as a snack.................... 5<br>Other (Please specify) 96	Where did you get this food item from?<br>Gift .... 1<br>Purchased .... 2<br>Other (Please specify)...... 3	If purchased, where was it purchased from?<br>Street Vendor.....................1<br>Local shop ....2<br>Haat/Bazar.........................3<br>School gate ....4<br>Other (Please specify)........5	Was the food covered?<br>Yes...1<br>No…2	If covered, was it inside a sealed packet?<br>Yes...1<br>No…2
MID	Code 1	Code↑	Code↑	Code↑	Code↑	Code↑
**MID**	**X2a_01**	**X2a_02**	**X2a_03**	**X2a_04**	**X2a_05**	**X2a_06**

Module End Time: Hour | | | Minute | | |

CODE 1:
Rice...............................1
Khichuri 2
Ruti 3
Ruti 4
Parota 5
Bonroti/Paoroti..............6
Butter bun......................7
Lentil.............................8
Bharta............................9
Paes/firni/semai...........10
Bhapa pitha 11
Chitoi pitha..................12
Piaju 13
Shingara 14
Samusa 15
Alur chop 16
Dalpuri 17
Alupuri 18
Beguni 19
Patise............................20
Chhola 21
Ghugni 22
Chotputi........................23
Fuchka..........................24
Bhelpuri........................25
Jilapi.............................26
Moa (puffed rice mixed with molasses).............27
Biscuit 28
Cake 29
Chocolate/hard candy/logenze..............30

Murali 31
Goja 32
Candyfloss 33
Kotkoti.................................34
Shon papdi 35
Sesame Khaja 36
Nimki...................................37
Jhalmuri 38
Unpacked (open) chanachur .39
Sliced hogplum mixed with salt and chili 40
Sliced green mango mixed with salt and chili 41
Sliced guava mixed with salt and chili 42
Sliced elephant apple mixed with salt and chili.................43
Tamarind mixed with salt and chili......................................44
Cadabel................................45
Other sliced fruits(please specify) 46
Mango bar............................47
Open chutney/aachar 48
Sugarcane juice....................49
Palm cabbage juice 50
Date juice.............................51
Other juices (sold without packaging) 52
Non-branded ice-cream.........53
Branded ice cream (e.g. Igloo) 54
Palm cabbage nucleus...........55
Cucumber 56
Khirai...................................57

Other 1 (please specify) 58
Other 2(please specify) 59

Household Number: [ ] [ ] [ ] [ ]

Module X3: Household Food Habit (Female)

Note to Enumerators: Ask these questions to the primary female member or the person who is mainly responsible for preparing food for the household.

Module start time: Hour [ ] [ ] Minu [ ] [ ] Respondent ID: [ ] Consent : [ ] Yes ...1 No.....2

Question		Code	Response	Item	Food Item	Food frequency: *READ:* Now I would like to ask you about foods that the members of your household consumed at home. Could you please tell me how many days in the past week your household has eaten the following food items, prepared and/or consumed at home and what the source of the food was? Did you eat this in the last 24 hours Yes…1 No….2 Not applicable...99											How many days consumed in the last 7 days? If 0>>skip to next food item CODE 1	What was the main source of this food in the last 7 days? CODE 2
						< 2 year old child			< 2 year old child's mother		14 to 49 year old female					Other members of the household		
						**X3_09**			**X3_10**		**X3_11**					**X3_12**	**X3_07**	**X3_08**
						**MID**	**MID**	**MID**	**MID**	**MID**	**MID**	**MID**	**MID**	**MID**	**MID**			
**X3_01**	In the past 4 weeks was there ever no food to eat of any kind in your house because of lack of resources to get food?	Yes… 1<br>No… 1 >> X3_03		1	Cereals (maize, sorghum, millet, barley) and food made from grains, such as bread, rice, noodles, porridge, or [other local grain food]?													
**X3_02**	How often did this happen in the past 4 weeks?	Rarely (1-2 times) … 1<br>Sometimes (3-10 times) … 2<br>Often (> 10 times) …. 3		2	Pumpkin, carrots, squash, or sweet potatoes that are yellow or orange inside or [other local yellow/orange foods]?													
				3	White potatoes, white yams, cassava, [other local root crops] or any other foods made from roots?													

				4	Any plantain or green bananas?													
**X3_03**	In the past 4 weeks did you or any household member go to sleep at night hungry because there was not enough food?	Yes… 1<br>No… 1 >> X3_05		5	Any dark green leafy vegetables such as [local dark green leafy vegetables]?													
				6	Other Vitamin A rich vegetables													
				7	Any other vegetables?													
				8	Ripe mangoes, ripe papayas or [other local vitamin A-rich fruits]?													
**X3_04**	How often did this happen in the past 4 weeks?	Rarely (1-2 times) … 1<br>Sometimes (3-10 times) … 2<br>Often (> 10 times) …. 3		9	Any other fruits?													
				10	Any liver, kidney, heart, or other organ meats from domesticated animals such as cow, pig, lamb, goat, chicken, or duck?													
				11	Any meat from domesticated animals, such as cow, pig, lamb, goat, chicken, or duck?													
**X3_05**	In the past 4 weeks did you or any household member go a whole day and night without eating anything at all because there was not enough food?	Yes… 1<br>No… 2 >> X3_07		12	Any liver, kidney, heart, or other organ meats from wild animals such as [turtle, snake, wild pig, wild chicken etc.]?													
				13	Any flesh from wild animals, such as [turtle, snake, wild pig, wild chicken etc.]?													
				14	Eggs?													
				15	Fresh or dried fish, shellfish, or seafood?													

Household Number: | | | | |

				16	Any foods made from beans, peas, or lentils, such as [anchor *dal*, *shimer bichi*, *mosur dal* etc.]?													
				17	Any foods made from nuts or seeds such as [*chinabadam, til, tishi* etc.]?													
				18	Milk, cheese, yogurt, or other milk products?													
				19	Any oil, fats, or butter, or foods made with any of these?													
				20	Any sweet or sugary foods such as honey, chocolates, sweets, candies, pastries, cakes, or biscuits?													
				21	Condiments for flavor, such as *aachars*, chilies, spices, herbs, fish powder or [*chuijhal, darchini, chukaru, mouriseed etc.*]?													
**X3_06**	How often did this happen in the past 4 weeks?	Rarely (1-2 times)… 1<br>Sometimes (3-10 times)…. 2<br>Often (> 10 times)… 3		22	Grubs, snails or insects such as [*shamuk, jhinuk* etc.]?													
				23	Tobacco (and other addictive substances)													
				24	Foods made with red palm oil, red palm nut, or red palm nut pulp sauce?													
				25	Any other foods (not mentioned in the list above)													

Household Number: | | | |

Code 1: Food Frequency	Code 2: Source of Food
Not eaten 0	Own production.......................... 1
1 day...................................... 1	Hunting/gathering/fishing........... 2
2 days 2	Bought using cash 3
3 days 3	Bought on credit 4
4 days 4	Borrowed (friends/relatives)....... 5
5 days 5	Gifts (friends/relatives)............... 6
6 days 6	Begging 7
7 days 7	Received as payment 8
	Food assistance........................... 9
	Collected 10

Module X4: Bad time (keeping income in mind). Recall period: last 12 months (2017): (Male and Female)

		Month 1 (A)	Month 2(B)	Code	
X4_1	Based on your income which months are usually the worst for your household			January ..... 1<br>February ..... 2<br>March ..... 3<br>April.....4<br>May.....5<br>June.....6<br>July.....7	August.....8<br>September.....9<br>October.....10<br>November.....11<br>December.....12<br>No income shortage in any of the months.............13

ENSURE COMPLETE PRIVACY BEFORE CONTINUING WITH THIS MODULE.

Module End Time: | Hour | | | Minute | | |

Household Number: | | | |

Module X5: Food Security and Resilience (Female)

Module start time: Hour | | | Minu | | | Respondent ID: | | Consent : | | Yes ...1 No.....2

Recall period: Past 12 months

Note to Enumerators: Respondent should be the individual is responsible for preparing food for the household

Now I would like to ask you some questions about your food consumption in the past 12 months.

Serial no.	Question	Response	Response Code
**X5_01**	During the past 12 months, was there a time when you or others in your household were worried you would not have enough food to eat because of a lack of money or other resources?		Yes……………..1<br>No…………………2<br>Refused…………….3
**X5_02**	During the past 12 months, was there a time when you or others in your household were unable to eat healthy and nutritious food because of a lack of money or other resources?		Yes……………..1<br>No…………………2<br>Refused…………….3
**X5_03**	During the past 12 months, was there a time when you or others in your household ate only a few kinds of foods because of a lack of money or other resources?		Yes……………..1<br>No…………………2<br>Refused…………….3
**X5_04**	During the past 12 months, was there a time when your household did not have food because of a lack of money or other resources?		Yes……………..1<br>No…………………2<br>Refused…………….3
**X5_05**	During the past 12 months, was there a time when you or others in your household ate less than you thought you should because of a lack of money or other resources?		Yes……………..1<br>No…………………2<br>Refused…………….3
**X5_06**	During the past 12 months, was there a time when you or others in your household had to skip a meal because there was not enough money or other resources to get food?		Yes……………..1<br>No…………………2<br>Refused…………….3
**X5_07**	During the past 12 months, was there a time when you or others in your household were hungry but did not eat because there was not enough money or other resources for food?		Yes……………..1<br>No…………………2<br>Refused…………….3
**X5_08**	During the past 12 months, was there a time when you or others in your household went without eating for a whole day because of a lack of money or other resources?		Yes……………..1<br>No…………………2<br>Refused…………….3

Household Number: | | | | |

Next I will ask you some questions about other kinds of difficult times that people face in the past 12 months

Shocks (unexpected events)	Shock Code	Has this shock occurred in the past 12 months?<br>Yes .... 1<br>No........... 2 >> NEXT Row	How severely did this event affect your hh's economic condition?<br><br>Not Severe…1<br>Somewhat severe…2<br>Severe…3<br>Extremely severe…4<br>Refused to answer…5	How severely did this event affect your hh's food condition?<br><br>Not Severe…1<br>Somewhat severe…2<br>Severe…3<br>Extremely severe…4<br>Refused to answer…5	If T1_02 is "yes" how have you or your hh coped with this situation?		
					Code 1		
**X5_09a**	**code**	**X5_09b**	**X5_09c**	**X5_09d**	**X5_09e1**	**X5_09e2**	**X5_09e3**
In the past 12 months, did your household face difficult times as a result of having too much rain?	**41**						
In the past 12 months, did your household face difficult times as a result of having too little rain?	**42**						
In the past 12 months, did your household face difficult times as a result of erosion of your land?	**43**						
In the past 12 months, did your household face difficult times as a result of losing your household's land?	**44**						
In the past 12 months, did your household face difficult times as a result of sharp increases in the price of food?	**45**						
In the past 12 months, did your household face difficult times as a result of someone stealing or destroying household members' belongings?	**46**						
In the past 12 months, did your household face difficult times as a result of not being able to access inputs for your crops?	**47**						
In the past 12 months, did your household face difficult times as a result of disease affecting your crops?	**102**						
In the past 12 months, did your household face difficult times as a result of pests affecting your crops?	**103**						
In the past 12 months, did your household face difficult times as a result of someone stealing crops from your household?	**48**						
In the past 12 months, did your household face difficult times as a result of not being able to access inputs for your livestock?	**49**						
In the past 12 months, did your household face difficult times as a result of disease affecting your livestock?	**50**						
In the past 12 months, did your household face difficult times as a result of someone stealing animals from your household?	**13**						
In the past 12 months, did your household face difficult times as a result of not being able to sell the crops, livestock, or other products your household produces for a fair price?	**51**						
Has anyone in your household experienced a severe illness in the past 12 months?	**52**						
Has your household experienced the death of a family member in the past 12 months?	**2**						

Household Number: | | | | |

Code 1: Coping strategies		
None........ 1<br>Sold land (specify homestead or agricultural) .... 2<br>Mortgaged/leased land (specify homestead or agricultural) ...... 3<br>Sold productive asset (specify)........ 4<br>Mortgaged productive asset (specify)........ 5<br>Sold consumption asset (specify) .... 6<br>Mortgaged consumption asset (specify) .... 7<br>Took loan from NGO/institution .... 8<br>Took loan from mahajan/non-institutional source 9	Ate less food to reduce expenses........ 10<br>Ate lower quality food to reduce expenses........ 11<br>Took children out of school .... 12<br>Transferred children to less expensive school .... 13<br>Adult household member took job elsewhere temporarily .... 14<br>Sent household member away permanently .... 15<br>Sent children to be fostered by relatives........ 16<br>Sent children into domestic service........ 17<br>Sent children to work somewhere other than domestic service . 18<br>Sent children to his parental home 19	Emergency receipt of remittance from migrant family member. 20<br>Forced to change occupation .... 21<br>Moved to less expensive housing........ 22<br>Sent non-working household member to work .... 23<br>Took help from others .... 24<br>Other (specify)........ 25

Now I would like to ask you a few more questions concerning these difficult times

Serial no.	Question	Response			Response Code
**X5_10**	Would you say that right now, your household's ability to meet your food needs is:<br><br>Better than before these difficult times?<br>The same as before these difficult times?<br>Or worse than before these difficult times?				BETTER THAN BEFORE DIFFICULT TIMES………..1<br>SAME AS BEFORE DIFFICULT TIMES………….2<br>WORSE THAN BEFORE DIFFICULT TIMES…….3<br>HOUSEHOLD DID NOT FACE ANY NEGATIVE SHOCKS…….4<br>REFUSED…………………….7
**X5_11**	Looking ahead over the next year, do you believe your household's ability to meet your food needs will be:<br><br>Better than before these difficult times?<br>The same as before these difficult times?<br>Or worse than before these difficult times?				BETTER THAN BEFORE DIFFICULT TIMES………..1<br>SAME AS BEFORE DIFFICULT TIMES………….2<br>WORSE THAN BEFORE DIFFICULT TIMES…….3<br>HOUSEHOLD DID NOT FACE ANY NEGATIVE SHOCKS…….4<br>REFUSED…………………….7
**X5_12**	Thank you for sharing your experiences.<br><br>Now I will ask you some questions about whether your household will be able to lean on others for financial or food support during difficult times.<br><br>By difficult times I mean times when there is loss of a family member, loss of income, hunger, drought, flood, conflict or similar events.<br><br>Will your household be able to lean on:<br><br>a) Relatives living in your community?<br>b) Relatives living outside your community?<br>c) Non-relatives living in your community?<br>d) Non-relatives living outside your community?	no.	Item	Answer	
		a	Relatives living in your community		
		b	Relatives living outside your community		
		c	Non-relatives living in your community		
		d	Non-relatives living outside your community		
**X5_13**	Will the same people that you will be able to lean on during your difficult times also be able to lean on you for financial or food support during their difficult times?				
**X5_14**	Do you believe your local government will help the community cope with difficult times in the future, for example during [flood, drought, tornado, cyclone, etc. (any event will have a negative impact on all members of the community)]?				YES…….1<br>No, Will not be able to……..2<br>No, support not needed……..3

Module End Time: | Hour | | | Minute | | |

Household Number: | | | | |

Module Y: Nutrition Practices and Services (Female)
Module Y1: Infant and Young Child Feeding (IYCF) Practices and Use of Micronutrients (Female)

Module start time: Hour | | | Minu | | | **Respondent ID:** | | Consent : | | Yes ...1 No.....2

Question Number	Questions	Response	Code
**Y1_00**	Is there any child less than 2 years in this household?		Yes............................1<br>No.............................2>> **Module Y8**

Note to Enumerators: The questions below are concerning children of less than 2 years old.

Question Number	Questions	Youngest child <24 months		Code
		Child 1	Child 2	
Child's Member ID	Child's name and ID (from Module B)	Child's Name:_____________<br>Mem. ID	Child's Name:_____________<br>Mem ID	Name and Member ID
Mother's Member ID	Child's mother's name and ID<br><br>(from Module B)	Mother's Name: _______________________<br>Mem. ID		Name and Member ID<br>If individual is not a member of the household, write 55.
Father's Member ID	Child's father's name and ID<br><br>(from Module B)	Father's Name: _______________________<br>Mem. ID		Name and Member ID<br>If individual is not a member of the household, write 55.
Y1_01	Where was this child of yours <NAME> delivered?			Your home .... 1<br>Natal house.. 2<br>Other house .... 3<br>Government/private hospital, clinic, UHC .... 4<br>Other (specify) 5
Y1_02	Who was present to help you during <NAME's> delivery?<br><br>(Multiple response possible)			Govt. Hospital/Upazila Health Comple .... 1<br>Doctor .... 2<br>Midwife/nurse .... 3<br>Govt. Heath Worker (FWA/HA) .... 4<br>MA/SACMO... 5<br>BRAC SS .... 6<br>BRAC SK.. 7<br>BRAC Pushti Kormi (PK)/IYCF Promoter .... 8<br>Others NGO Worker .... 9<br>TTBA.. 10<br>TBA .... 11<br>Village Doctor.. 12<br>Homeopath Doctor... 13<br>Kabiraj/Herbal healer .... 14<br>Spiritual Healer..15<br>Pharmacy ....16<br>Husband..17<br>Mother/Mother in law ....18<br>Other Family members...19<br>Neighbors/ Friends...20<br>Private Hospital/ Clinic..21<br>Mother and Children Welfare Centre (MCWC) ....22<br>Community Clinic..23<br>Satellinte Clinic/ EPI Center ....24<br>Smiling Sun Clinic...25<br>Self...26<br>Nobody ....27<br>Other (specify) ....77<br>Do not know/ do not remember....88

Household Number: [ ] [ ] [ ] [ ]

Question Number	Questions	Youngest child <24 months		Code
		Child 1	Child 2	
Y1_03	Did anyone help you put the baby to the breast after birth?	[ ]	[ ]	Yes ....1<br>No ....2 >> Y1_03b
Y1_03a	Who helped you put the baby to the breast after birth?	[ ]	[ ]	Govt. Hospital/Upazila Health Comple .... 1<br>Doctor .... 2<br>Midwife/nurse .... 3<br>Govt. Heath Worker (FWA/HA) .... 4<br>MA/SACMO .... 5<br>BRAC SS .... 6<br>BRAC SK .... 7<br>BRAC Pushti Kormi (PK)/IYCF Promoter .... 8<br>Others NGO Worker .... 9<br>TTBA .... 10<br>TBA .... 11<br>Village Doctor .... 12<br>Homeopath Doctor .... 13<br>Kabiraj/Herbal healer .... 14<br>Spiritual Healer .... 15<br>Pharmacy .... 16<br>Husband .... 17<br>Mother/Mother in law .... 18<br>Other Family members .... 19<br>Neighbors/ Friends .... 20<br>Private Hospital/ Clinic .... 21<br>Mother and Children Welfare Centre (MCWC) .... 22<br>Community Clinic .... 23<br>Satellinte Clinic/ EPI Center .... 24<br>Smiling Sun Clinic .... 25<br>Self .... 26<br>Nobody .... 27<br>Other (specify) .... 77<br>Do not know/ do not remember.... 88
Y1_03b	How soon did you put the child to the breast after birth?<br>(Note: If respondent says that the baby was put to breast right after birth or within 1 hour of birth then write 0 hours. If the respondent mentions within 1 to 23 hours of birth then write the number of hours. If the time was 24 hours more than 24 hours then write number of days)	Hours [ ]<br>Days [ ]	Hours [ ]<br>Days [ ]	Right after birth or within less than 1 hour, write “0”<br>If less than 24 hours, write hours<br>If 24 hours or more than 24 hours, write number of days
Y1_03c	Was there anything put to the child’s mouth immediately after birth?	[ ]	[ ]	Yes .... 1<br>No .... 2>>Y1_04<br>Don’t know ....88>>Y1_04
Y1_03d	What was put to the child’s mouth immediately after birth by you or anyone else?<br>***Interviwer:*** Please verify what food was given to the child by mother and also by anyone else	[ ]<br>[ ]<br>[ ]	[ ]<br>[ ]<br>[ ]	Honey .... 1<br>Mustard oil .... 2<br>Plain water .... 3<br>Sugar/glucose water .... 4<br>Tea/Coffee .... 5<br>Cow/Goat milk .... 6<br>Breast milk .... 7<br>Other (specify) .... 77<br>Do not remember 88

Household Number:

Question Number	Questions	Youngest child <24 months		Code
		Child 1	Child 2	
Y1_03e	Who put food to the child's mouth immediately after birth?<br>(Multiple response possible)			Govt. Hospital/Upazila Health Comple .... 1<br>Doctor .... 2<br>Midwife/nurse .... 3<br>Govt. Heath Worker (FWA/HA) .... 4<br>MA/SACMO .... 5<br>BRAC SS .... 6<br>BRAC SK .... 7<br>BRAC Pushti Kormi (PK)/IYCF Promoter .... 8<br>Others NGO Worker .... 9<br>TTBA .... 10<br>TBA .... 11<br>Village Doctor .... 12<br>Homeopath Doctor .... 13<br>Kabiraj/Herbal healer .... 14<br>Spiritual Healer ....15<br>Pharmacy ....16<br>Husband ....17<br>Mother/Mother in law ....18<br>Other Family members ....19<br>Neighbors/ Friends ....20<br>Private Hospital/ Clinic ....21<br>Mother and Children Welfare Centre (MCWC) ....22<br>Community Clinic ....23<br>Satellinte Clinic/ EPI Center ....24<br>Smiling Sun Clinic ....25<br>Self ....26<br>Nobody ....27<br>Other (specify) ....77<br>Do not know/ do not remember....88
Y1_04	During the first 3 days after the baby was born, what was given to the child by you or anyone else?<br><br>(Multiple response possible			Honey……………1<br>Mustard oil……………2<br>Plain water……………3<br>Sugar/glucose water……………4<br>Tea/Coffee……………5<br>Cow/Goat milk……………6<br>Breast milk……………7<br>Other (specify)……………77<br>Do not remember……………88
Y1_05	Did you give the child colostrum?			Yes (gave to child) .... 1<br>No (did not give to child) 2
Y1_05a	From birth until now, has this child ever been given expressed breast milk (in a cup or bowl)?			Yes ....1<br>No 2
Now we would like to ask you about what the child's current eating pattern:				
Y1_06	Is the child still breastfeeding?			Yes .... 1>>Y1_09<br>No .... 2<br>Never.......... 3>> Y1_09
Y1_07	If no, at what age did you stop breastfeeding the child?			Month<br>Don't Know/cannot remember....88
Y1_08	Why did you stop breastfeeding?			Problems with breast (pain)……………1<br>Child does not suck well……………2<br>Not enough time to feed child……………3<br>Child already grown up/ No need for breast feeding……………4<br>Mother got pregnant……………5<br>New baby born……………6<br>Cracked nipples…7<br>Felt not enough breast milk………8<br>Other (specify)….9
Y1_09	At what age did you start giving the following liquids/foods to the child?<br>If mother fed her child any of the following food within the first 29 days (less than 1 months of age), this can be noted as "0" month.			
	1. Water			At "0" month of age 0

Household Number: | | | | |

Question Number	Questions	Youngest child <24 months		Code
		Child 1	Child 2	
	2. Other non breast milk liquids (sugar/glucose water, tea, fruit juice etc.)			At "1" month of age .... 1<br>At "2" months of age... 2<br>At "3" months of age... 3<br>At "12" months of age... 12<br>So on<br>Don't Know .... 88<br>Not given yet...............................99
	3. Cow/Goat milk			
	4. Sooji, rice gruel, etc.			
	5. Semi-solid foods (soft rice, khichuri, mashed potato, ripe banana, other mashed family foods etc.)			
	6. Solid foods (such as rice, wheat, puffed/ pressed rice etc.)			
	7. Fish			
	8. Meat (chicken, mutton, beef, etc., khichuri with meat)			
	9. Eggs			
	10. Legumes (pulse, peas, etc.)			
	11. Green vegetables			
	12. Snack foods (chanachur, chips)			
	13. Biscuit			
	14.Pustikona/Monimix/Mymix			
The following questions are based on previous day (last 24 hours) recall, i.e., yesterday during the day and the night.				
Y1_10	How many times did you breastfeed [NAME] yesterday, during the day or night?			Number of times<br>Stopped breast feeding/Never breast fed 99

Household Number: | | | | |

Question Number	Questions	Youngest child <24 months		Code
		Child 1	Child 2	
Y1_11	Other than breast milk, how many times did [NAME] drink other milk, formula or yogurt yesterday, during the day or night?<br><br>Do not include number of times the child was breastfed in this question. This variable is only to capture milk or milk products **other than breast milk**	☐	☐	Number of times<br>Not given yet.. 99
Y1_12	How many times did [NAME] eat solid, semi-solid or soft foods other than liquids yesterday, during the day or night?<br><br>**Semi-solid** foods such as soft rice, mashed potato, ripe banana, other mashed family foods etc. **Solid** foods such as rice, wheat, puffed/pressed rice etc.<br>Meals include both meals and snacks (other than trivial amounts)	☐	☐	Number of times<br>Not given yet...............................…………………………99
Y1_12a	Of the cooked foods that you fed the child yesterday, could you tell us about how many baati's (show the measuring cup) you offered the child to eat yesterday?<br><br>Please think of the total amount of food the child was given and convert it into milliliters. .	☐	☐	Milliliter<br>Not given yet……………………………………9999

Household Number: | | | | |

Yesterday (during the day or the night) did you give any of the following liquids to the child?

Please describe everything that ***NAME*** drank yesterday during the day or night, whether at home or outside the home.

a) Think about when ***(NAME)*** first woke up yesterday. Did ***(NAME)*** drink anything at that time? *If yes*: Please tell me everything ***(NAME)*** drank at that time. *Probe:* Anything else? U*ntil respondent says nothing else.* I*f no, continue to Question b).*

b) What did ***(NAME)*** do after that? Did ***(NAME)*** drink anything at that time?

If yes: Please tell me everything ***(NAME) drank*** at that time. *Probe:* Anything else? U*ntil respondent says nothing else*.

R*epeat* q*uestion b) above until respondent says the child went to sleep until the next day.*

Once the respondent finishes recalling foods eaten, read each food group where '1' was not circled, ask the following question and Circle *'1' if respondent says yes, '2' if no and '99' if don't know:*

Question Number	Questions	Youngest child <24 months				Code
		**Child 1**		**Child 2**		
Y1_13	Yesterday (during the day or the night) did you give any of the following liquids to the child?	Yesterday	Has the child ever been fed this liquid? (ask only if child 0-6 months old)	Yesterday	Has the child ever been fed liquid? (ask only if child 0-6 months old)	
Y1_13a	Breast milk					Yes……………….1<br>No 2
Y1_13b	Water					
Y1_13c	Baby formula (prepared food for child)					
Y1_13d	Any other kind of milk (powder, cow/goat milk etc.)					
Y1_13e	Fruit juice (made at home)					
Y1_13f	Fruit juice (purchased, packaged)					
Y1_13g	Water-based liquids, teas, sugar water, coffee					
Y1_14	Yesterday (during the day and the night), did you use a baby bottle to feed the child?					Yes……………….1<br>No 2

Did your child eat (or drink) any of the following foods yesterday (during the day or night)?
Please describe everything that (NAME) ate yesterday during the day or night, whether at home or outside the home.
a) Think about when (NAME) first woke up yesterday. Did (NAME) eat anything at that time? If yes: Please tell me everything (NAME)ate at that time. Probe: Anything else? Until respondent says nothing else. If no, continue to Question b).
b) What did (NAME) do after that? Did (NAME) eat anything at that time?
If yes: Please tell me everything (NAME) ate at that time. Probe: Anything else? Until respondent says nothing else.
Repeat question b) above until respondent says the child went to sleep until the next day.
If respondent mentions mixed dishes like a PORRIDGE, sauce or stew, probe:
c) What ingredients were in that (MIXED DISH)? Probe: Anything else? Until respondent says nothing else.
As the respondent recalls foods, underline the corresponding food and circle '1' in the column next to the food group. If the food is not listed in any of the food groups below, write the food in the box labeled 'other foods'. If foods are used in small amounts for seasoning or as a condiment, include them under the condiments food group.

Household Number: | | | | |

Once the respondent finishes recalling foods eaten, read each food group where ‘1’ was not circled, ask the following question and Circle ‘1’ if respondent says yes, ‘2’ if no and ‘8’ if don’t know:

Question Number	Questions	Youngest child <24 months				Code
		Child 1		Child 2		
Y1_15	Did your child eat (or drink) any of the following foods yesterday (during the day or night)?	Yesterday	Has the child ever been fed this liquid? (ask only if child 0-6 months old)	Yesterday	Has the child ever been fed this liquid? (ask only if child 0-6 months old)	
Y1_15a	Rice					Yes .... 1<br>No...... 2
Y1_15b	Cereals such as wheat, pressed rice, puffed rice, suji					
Y1_15c	Purchased baby cereals (NAME: such as **Cerelac**)<br>Add name of most common food that iron fortified. Country specific					
Y1_15d	Legume: daal					
Y1_15e	Green leafy vegetables					
Y1_15f	Pumpkin, orange yam, orange-red-flesh sweet potatoes, carrots, tomato (vitamin-A rich)					
Y1_15g	Any other vegetables (starchy vegetables: potatoes, yam, plantain)					
Y1_15h	Ripe papaya or mango					
Y1_15i	Other fruits such as oranges, banana, grapefruits					
Y1_15j	Any other fruits					
Y1_15k	Meat such as beef, mutton					
Y1_15l	Chicken, duck, pigeon					
Y1_15m	Liver, heart, kidneys					
Y1_15n	Fish					
Y1_15o	Eggs					
Y1_15p	Peanuts, groundnuts, other nuts					
Y1_15q	Milk (non-human milk – cow, goat or powder)					
Y1_15r	Milk products (yogurt, rice pudding etc.)					
Y1_15s	Fat (oil, butter, ghee)					
Y1_15t	Chips or chanachur					
Y1_15u	Bread or buns					
Y1_15v	Candies or chocolates					
Y1_15v1	Biscuits					
Y1_15w1	Nutrient Powder/ Vitamin Sprinkles (Micronutrients, Monimix, MyMix, Pustika etc.)					
Y1_15w	Any iron containing tablet					
Y1_15x	Spices					
Y1_15y	Others (Specify)					

Household Number: | | | |

Question Number	Questions	Youngest child <24 months		Code	
		Child 1	Child 2		
Y1_15_1	In the past 1 month, did you face any problems feeding your child?			Yes..1<br>No...2>>Y1_16	
Y1_15_2	What kind of problems do you currently face?<br><br>(Multiple response possible)<br>[bring up to 3 answers]			Problems with breast (pain)……...1<br>Child does not suck well………...2<br>Not able to breastfeed well………3<br>Not enough time to feed child…...4<br>Cracked nipples………………….5	Feel not enough breast milk…..6<br>Poor appetite………………….7<br>Child runs around too much…..8<br>Child does not like solid foods.......9<br>Child sick……………………10<br>Other (specify)………………77
Y1_15_3	Did you seek help from anyone to help address this problem?			Yes..1<br>No...2>>Y1_16	

Household Number: | | | | |

<table>
<tr><th rowspan="2">Question Number</th><th rowspan="2">Questions</th><th colspan="2">Youngest child <24 months</th><th colspan="2" rowspan="2">Code</th></tr>
<tr><th>Child 1</th><th>Child 2</th></tr>
<tr><td>Y1_15_4</td><td>Who did you seek help from?

(Multiple response possible)</td><td></td><td></td><td>Govt. Hospital/Upazila Health Complex 1
Doctor 2
Midwife/nurse 3
Govt. Heath Worker (FWA/HA) 4
MA/SACMO 5
BRAC SS 6
BRAC SK 7
BRAC Pushti Kormi (PK)/IYCF Promoter 8
Others NGO Worker 9
TTBA 10
TBA 11
Village Doctor 12
Homeopath Doctor 13
Kabiraj/Herbal healer .... 14</td><td>Spiritual Healer 15
Pharmacy 16
Husband 17
Mother/Mother in law 18
Other Family members 19
Neighbors/ Friends 20
Private Hospital/ Clinic 21
Mother and Children Welfare Centre (MCWC) 22
Community Clinic 23
Satellite Clinic/ EPI Center 24
Smiling Sun Clinic 25
Self 26
Nobody 27
Other (specify) 77
Do Not Know/ Do Not Remember 88</td></tr>
</table>

Household Number:

Y1_15_5	What feeding advice did the person give you?<br><br>(Multiple response possible)<br>[bring upto 2 answers]			Showed how to hold the baby and attach baby's mouth to breast during breast feeding .... 1<br>Give pressed breast milk when the mother stays away from the baby .... 2<br>Give the child only breast milk for the first 6 months .... 3<br>Continue breastfeeding the child up to 2 years .... 4<br>Breastfeed more often .... 5<br>Give child other milk (cow/goat/buffalo milk) .... 6<br>Give infant formula or powder milk (Nan, Cerelac, Lactogen, Horlicks) .... 7<br>Give sooji/gruels mixed with milk .... 8<br>Give Khichuri .... 9<br>Give mashed family food .... 10<br>Give animal source food (egg, fish, chicken, liver) .... 11<br>Give vitamin/supplementary food .... 12<br>Give favorite nutritious food 13	Give favorite nutritious food .... 13<br>Advise to feed frequently .... 14<br>Give an extra meal for 2 weeks after the child recovers from an illness .... 15<br>Give ORS/home prepared solution .... 16<br>Feed less than usual .... 17<br>Give syrups .... 18<br>Give traditional medicine .... 19<br>Give treated water .... 20<br>Give carrot juice or rice water .... 21<br>DO NOT give other milk or any food to the child other than breast milk until a child is 6 months old .... 22<br>DO NOT give infant formula or powder milk(Nan, Cerelac, Lactogen, Horlicks .... 23<br>DO NOT give Khichuri .... 24<br>DO NOT give mashed family food .... 25<br>DO NOT give animal source food(egg, fish, chicken, liver) 26

Household Number: | | | | |

Question Number	Questions	Youngest child <24 months		Code	
		Child 1	Child 2		
					Others (specify) 77
Y1_15_6	Did they suggest any baby food/formula?			Yes……………………………………..….1<br>No..2	

MICRONUTRIENTS POWDER RELATED QUESTIONS

Question Number	Questions	Youngest child <24 months						Code
		Child 1			Child 2			
Y1_16	Have you ever heard of a powder called Sprinkles/ (show example packets) for putting in the food of young children?							Yes……………………………..1<br>No………………………………2>>Y1_31<br>Don't remember/don't know……88>Y1_31
Y1_16a	Which powder have you heard about?	Pustikona	Monimix	Other (please specify)	Pustikona	Monimix	Other (please specify)	Yes... 1<br>No.. 2

Household Number: | | | |

Question Number	Questions	Youngest child <24 months		Code
		Child 1	Child 2	
Y1_17	Where did you hear about this?<br>(multiple answers possible)			Television/radio advertisement…………………...1<br>Pharmacy/ shop in village………………………..2<br>Doctor (MBBS/ Village)…………………………3<br>BRAC Health Worker<br>(SS/PS/SK./PK/PO)……………………………4<br>From other NGO workers……………………………………….....5<br>From neighbor or family member………………..6<br>Hospital……………………………………………7<br>Other _______ 77
Y1_18	Was (NAME) **<u>EVER</u>** given food mixed with micronutrient powder/ vitamin powder?			Yes.. 1>>Y1_18b<br>No... 2
Y1_18a	Why was (NAME) never given micronutrient powder/ vitamin powder?<br><br>(multiple answers possible)			Don't think that it is useful for the child .... 1<br>Child doesn't like Pushtikona .... 2<br>Child gets constipation/black stool .... 3<br>Child gets stomach ache.. 4<br>Not affordable/too expensive .... 5<br>Family barriers/family members discourage .... 6<br>It is medicine, only needed if child is ill .... 7<br>It is like sugar, not needed .... 8<br>Doctor/SS/Other health official did not prescribe .... 9<br>Buy another brand of MNP (Monimix, Mymix etc.) .... 10<br>Received it for free.. 11<br>The child is still less than 6 months old .... 12<br>Others (specify)... 77<br>Don't know 99
Y1_18b	Which of these micronutrients [NAME on the side] was given to child?			Pushtikona... 1<br>Monimix .... 2<br>MyMix .... 3<br>Pushtika... 4<br>Other 77
Y1_19	Was (NAME) never given micronutrient powder/ vitamin powder in the past month?			Yes.. 1<br>No... 2
Y1_20	During the past 7 days, on how many days did you mix micronutrient powder with food for (NAME)?			Days<br>Don't know/don't remember……….88
Y1_22	Did you <u>receive</u> packets of micronutrient powders from anyone or any program at any time in last six months?			Yes .... 1<br>No .... 2>>Y1_25<br>Don't remember/don't know. 88>> Y1_25

Household Number: | | | | |

Question Number	Questions	Youngest child <24 months		Code
		Child 1	Child 2	
Y1_23	Where did you receive these packets of micronutrient powders from?<br>(multiple answers possible)			BRAC SS .... 1<br>Other BRAC health workers(SK/PK/PO) .... 2<br>SMC/other health workers .... 3<br>Pharmacy .... 4<br>Local shop.................. 5<br>Doctor's Chamber.................. 6<br>Company's Medical Representative.................. 7<br>Others (specify)..................77
Y1_24	How many packets did you receive in the last six months?			Number<br>Don't remember/don't know....... 88
Y1_25	Did you purchase packets of micronutrient powders any time in last six months?			Yes .... 1<br>No .... 2<br>Don't remember/don't know.................. 88
Y1_26	Did you purchase packets of micronutrient powders any time in last one month?			Yes .... 1<br>No .... 2 >> Y1_28<br>Don't remember/don't know.................. 88 >> Y1_28
Y1_27	How many packets of micronutrient powders did you purchase in last one month?			Number<br>Don't remember/don't know....... 88
Y1_28	Where did you buy micronutrient powders from the last time you purchased micronutrient powders?			BRAC SS .... 1<br>other BRAC health workers(SK/PK/PO) .... 2<br>SMC/other health workers .... 3<br>Pharmacy .... 4<br>Local shop.................. 5<br>Doctor's chamber.................. 6<br>Company's Medical Representative.................. 7<br>Others (specify).................. 77<br>Did not purchase ....99>>Y1_30
Y1_29	What was the price of each packet of micronutrient powder?			Taka
Y1_30	Which members of your family consumed Sprinkles in the last one week?<br>[bring up to 4 answers]			Member ID<br>No family member consumed in last 1 week 98
Y1_31	Did you ever purchase any other vitamins or mineral supplements in last six months?<br><br>Give examples of locally available vitamins/minerals; note this is other than micronutrient powder			Yes .... 1<br>No .... 2<br>Don't remember/don't know.................. 88

Household Number:

Question Number	Questions	Youngest child <24 months		Code
		Child 1	Child 2	
Y1_32	Did you purchase any vitamin or mineral supplements in last one month?			Yes.. 1<br>No .... 2 >>Y1_35<br>Don't remember/don't know……………………………....88>>Y1_35
Y1_33	Can you tell us the name of the vitamin/mineral supplement you bought?			Vitamin/Mineral 1________________________<br>Vitamin/Mineral 2________________________<br>Don't know name..88<br>Did not purchase .... 9999>>Next Module
Y1_34	Was the empty bottle of vitamin/Mineral/packet checked?			Yes.. 1<br>No .... 2<br>Did not purchase in last 1 month…………………………99
Y1_35	Which members of your family consumed the vitamin/mineral supplements in the last one week? [bring up to 4 answers]			Member ID<br>None of the family member consumed in last 1 week…………………98

Household Number: ☐☐☐☐

Module Y2: Nutrition (IYCF) Knowledge of Respondent Mothers (Female)

Question Number	Questions	Response	Code
**Y2_00**	According to you, when can you start giving a young child the following foods?		
**1**	Water		<= 1 Month of age .... 0<br>At 1 Month of age .... 1<br>At 2 Month of age .... 2<br>At 3 Month of age .... 3<br>At 4 Month of age .... 4<br>At 5 Month of age .... 5<br>At 6 Month of age .... 6<br>At 7 Month of age .... 7<br>At 8 Month of age .... 8<br>At 9 Month of age .... 9<br>At 10 Month of age .... 10<br>At 11 Month of age .... 11<br>At 12 Month of age .... 12<br>Don’t know/didn’t answer .... 88<br>Should not be given 99
**2**	Rice, Bread, Pressed rice, Muri, sooji, etc.		
**3**	Legume: daal, pulse, peas		
**4**	Green leafy vegetables		
**5**	Vegetables such as pumpkin, orange yam, orange-red-flesh sweet potatoes, carrots, tomato (vitamin-A rich)		
**6**	Ripe papaya or mango		
**7**	Bananas		
**8**	Beef, mutton		
**9**	Chicken, duck, other poultry, chicken liver		
**10**	Fish (Big)		
**11**	Fish (Small)		
**12**	Eggs		
**13**	Peanuts, groundnuts, other nuts		
**14**	Milk (cow, goat or powdered)		
**15**	Purchased snack foods (chips, biscuits, chanachur, Chocolate/candies etc.)		
**16**	Other non-breast milk liquids (sugar/glucose water, tea, fruit juice etc.)		
**17**	Semi-solid foods (soft rice, khichuri, mashed potato, ripe banana, other mashed family foods etc.)		
**Y2_01**	How soon after birth should a baby start breastfeeding?		Immediately .... 1<br>Less than 1 hour after delivery .... 2<br>Some hours later but less than 24 hrs .... 3<br>1 day later .... 4<br>More than 1 day later .... 5<br>Do not think baby should be breastfed .... 6<br>Don’t know 88

Household Number: | | | | |

Question Number	Questions	Response	Code
**Y2_02**	What should a mother do with the “first milk” or colostrum?		Throw it away and start breastfeeding when the real milk comes in .... 1<br>Give it to her baby by breastfeeding soon after birth .... 2<br>Other (specify) .... 3<br>Don’t know 88
**Y2_03**	How often should a baby breastfeed?		Whenever baby wants .... 1<br>When you see the baby is hungry .... 2<br>When the baby cries .... 3<br>Other (specify) .... 4<br>Don’t know 88
**Y2_04**	If a mother thinks her baby is not getting enough breast milk, what should she do?		Breastfeed more often/more frequently .... 1<br>Give other liquids/foods .... 2<br>Mother needs to drink more water .... 3<br>Mother needs to eat more food .... 4<br>Other (specify) .... 5<br>Eat food that increases milk production .... 6<br>Feed the child with patience .... 7<br>Don’t know 88
**Y2_05**	Do you think that infants under 6 months of age should be given water if the weather is very hot?		Yes .... 1<br>No .... 2<br>Don’t know 88
**Y2_05a**	Do you think that a breastfeeding mother of an infant under 6 months of age should stop breastfeeding if she becomes pregnant?		Yes .... 1<br>No .... 2<br>Don’t know 88
**Y2_05b**	Until about what age should a baby be exclusively breastfed?		Months<br>Don’t know/Don’t remember 88
**Y2_06**	At what age should a baby first start to receive liquids (including water) other than breast milk?		Months<br>Don’t know 88
**Y2_07**	At what age should a baby first start to receive foods in addition to breast milk?		Months<br>Don’t know 88
**Y2_07a**	Until what age should a child be breastfed in addition to other food?		Months<br>Don’t know 88
**Y2_08**	Name one thing that can happen to children if they do not get enough iron (either in their diet or via iron supplements).		Impaired learning .... 1<br>Impaired development .... 2<br>Lower height .... 3<br>Weakened immune defense .... 4<br>Feel tired .... 5<br>Become anemic .... 6<br>Other (specify) .... 7<br>Don’t know 88
**Y2_09**	What seasoning (food item) is often fortified with iodine (a nutrient important for brain development)?		Salt .... 1<br>Other (specify) .... 2<br>Don’t know 88

Household Number: | | | | |

Question Number	Questions	Response	Code
**Y2_10**	For how long do children need an extra meal per day after they have been sick?<br>*(a meal in addition to the ones they are fed usually)*	Day ☐<br>Week ☐	Day/Week<br>Don't know 88
**Y2_11a**	What should a mother do in regard to child feeding when a child aged less than 6 months has diarrhea?<br>*(Multiple response possible)*		Give ORS/home-prepared solution .... 1<br>Feed less than usual .... 2<br>Continue breastfeeding .... 3<br>Breastfeed more often .... 4<br>Give syrups .... 5<br>Give traditional medicine .... 6<br>Give treated water .... 7<br>Give carrot juice or rice water .... 8<br>Other: .... 77<br>Don't know 88
**Y2_11b**	What should a mother do in regard to child feeding when a child aged more than 6 months has diarrhea?<br>*(Multiple response possible)*		Give ORS/home-prepared solution .... 1<br>Feed less than usual .... 2<br>Continue breastfeeding .... 3<br>Breastfeed more often .... 4<br>Give syrups .... 5<br>Give traditional medicine .... 6<br>Give treated water .... 7<br>Give carrot juice or rice water .... 8<br>Other: .... 77<br>Don't know .... .99
**Y2_12**	When should you wash your hands?<br>(multiple answers possible)		Before eating .... 1<br>After using the toilet .... 2<br>Before feeding the child .... 3<br>After cleaning a child who has defecated .... 4<br>Other (specify) .... 5<br>Don't know 88
**Y2_13**	What are some of the things we can do to encourage young children to eat their food?<br>(multiple answers possible)		Feed slowly and patiently .... 1<br>Talk to the child .... 2<br>Force the child .... 3<br>Reduce distractions .... 4<br>Feed other foods .... 5<br>Change flavor of the food .... 6<br>Give vitamins .... 7<br>Take the child's mind off .... 8<br>Start CF on time so child develops a habit .... 9<br>Food should be mashed .... 10<br>Maintain food consistency (concentration) .... 11<br>Offer a variety of foods. 12

Household Number: [ ][ ][ ][ ]

Question Number	Questions	Response	Code
			Offer the child his/her favorite nutritious food .... 13<br>Let the child eat with own hands (provided hands are washed) .... 14<br>Avoid junk food (chips, packet juice etc.) .... .15<br>Feed when child is hungry .... 16<br>Encourage and praise the child .... 17<br>Other (specify) .... 77<br>Don't know 88
**Y2_14**	What foods does a young child (<24 months) need in order to grow and develop their brain?<br>(multiple answers possible)	[ ][ ][ ][ ]	Gruels/bread/rice.......... 1<br>Gruel with milk.......... 2<br>Animal foods such as meat or chicken.......... 3<br>Fish .... 4<br>Eggs .... 5<br>Fruits.......... 6<br>Vegetables .... 7<br>Milk .... 8<br>Pulses (daal).......... 9<br>Other (specify) .... 10<br>Don't know 88
**Y2_14a**	Can you name some benefits of providing Pushtikona/micronutrient powder to children?<br>[multiple responses possible]	[ ][ ][ ]	Child will not be anemic. .... 1<br>Good for child's brains and intelligence/good in studies or school.......... 2<br>Child will grow well (height or weight) .... 3<br>Improved child development .... 4<br>Increases child's appetite .... 5<br>Child suffers less from illness.......... 6<br>Child cries less.......... 7<br>It has vitamins and minerals .... 8<br>Prevents diarrhea .... 9<br>Treats diarrhea .... 10<br>Improves child's immune system .... 11<br>Child is more energetic (feels less tired).......... 12<br>Others (Specify).......... 77<br>Don't know 88
**Y2_14b**	How many sachets of Pushtikona should be fed to a child in 7days?	[ ]	Number of sachets [___][___]<br>Don't know .... 88<br>Never Fed 99
**Y2_14c**	How much of the Pushtikona sachet should be mixed with the child's food at one meal?	[ ]	Full sachet.......... 1<br>Half sachet… .... 2<br>Less than half sachet .... 3<br>Others (specify) .... 4<br>Don't know 88
**Y2_14d**	Within how much time should the food after adding Pushtikona be fed to the child?	[ ]	Within 30 minutes.......... 1<br>Others [____] mins 2

Household Number: | | | | |

Question Number	Questions	Response	Code
			Don't know 88
**Y2_14e**	How hot or cold should the food be when adding Pushtikona to it?	☐	Hot (just cooked)… .... 1<br>Warm/room temperature (after cooking allow the food to cool)... 2<br>Cold .... 3<br>Others (specify) .... 4<br>Don't know 88

Module Y3: Awareness-Trial-Adoption of Sentinel Practices (Female)

Question Number	I will now ask you about a few child feeding practices. Can you tell me if you have ever heard about any of the following child feeding practices?	A. Have you heard about....?<br>yes .... 1>>B<br>No .... 2>>Next row	B. If ans is 'yes' for A<br><br>Where did you hear about this?<br><br>Family Member ..... 1<br>Friend/neighbor ..... 2<br>Health worker .... 3<br>Nurse/dispensary ... 4<br>Radio .... 5<br>TV .... 6<br>BRAC .... 7<br>Religious Leader.... 8<br>Health Center......... 9<br>Other (specify)....... 10	C. Did you ever try this?<br><br>Yes.........1>>Next Row<br>No ....2>>D	D. If ans is "No" for C, ask:<br><br>Can you tell me the main reason why you did not ever try this at home?<br><br>Next Qs.<br><br>Response	Code
**Y3_01**	Starting breast feeding immediately after delivery within 1 hour?	☐	☐	☐	☐	Code 1↓
**Y3_02**	Not giving anything except breast milk to your child for six months	☐	☐	☐	☐	Code 2 ↓
**Y3_03**	Feeding your baby mashed family foods in addition to breast feeding right at 7 months of age	☐	☐	☐	☐	Code 3↓
**Y3_04**	Feeding animal foods like fish, egg, liver, meat at least once a day? (for child more than 6 months old)	☐	☐	☐	☐	Code 4↓
**Y3_07**	Adding Pushtikonia/Monimix/MyMix/Sprinkles to the child's food	☐	☐	☐	☐	Code 5↓

Household Number: | | | | |

Code 1	Code 2	Code 3	Code 4	Code 5
Do not know................................ 1<br>Insufficient breast milk .... 2<br>Family members discouraged me 3<br>Other people discouraged me...... 4<br>Doctor forbid me......................... 5<br>Did not feel it to be necessary..... 6<br>Other (specify) 7	Do not know.......................... 1<br>Mother did not want............. 2<br>Family members told me to feed something else....................... 3<br>Others told me to feed something else .... 4<br>Insufficient breast milk .... 5<br>Other (specify) 6	Child does not like it................1<br>Not enough food at home ....2<br>Did not have required ingredients ....3<br>Ingredients too expensive ....4<br>Child not old enough ....5<br>Other (specify)..........................6<br>Did not feel it to be necessary...7	Do not know .... 1<br>Child does not like it...... 2<br>Did not have money....... 3<br>Fish, eggs, liver, meat too expensive .... 4<br>Child not old enough ..... 5<br>Other (specify).............. 6<br>Did not feel it to be necessary 7	Do not know ....1<br>Child does not like it..........2<br>Did not have money............3<br>Pushtikona too expensive ...4<br>Child not old enough ....5<br>Other (specify)....................6<br>Did not feel it to be necessary 7

Household Number: | | | | |

Module Y4: Immunization and Health Status of Young Children (<2 years) (Female)

Instructions: To be asked for all children under the age of 24 months

Important note: <u>finish asking all questions for the first child, and then ask the same set of questions for the younger child.</u>

Question number	Question	Child < 24 months				Response Code
		Child 1		Child 2		
Child's Member ID	Copy the child's Name and ID from module B	Name:____________________<br>Mem. ID		Name:____________________<br>Mem. ID		Name and Member ID
Mother's Member ID	Copy the child's mother's Name and ID from module B	Name: ____________________<br>Member ID				Name and Member ID<br>If not member of HH, then record 55
Father's Member ID	Copy the child's father's Name and ID from module B	Name: ______________________<br>Member ID				Name and Member ID<br>If not member of HH, then record 55
Y4_01	Present age of child in months					Age in months
Y4_02	Gender of the child					Boy... 1<br>Girl...............................2
Y4_03	Birth Order (number)					Write the exact number birth order of the child.
Y4_04	If the child is over 6 months old, has the child received vitamin A capsule in last 6 month?					Yes .... 1<br>No .... 2<br>N/A (age < 6 months)................ 9<br>Don't Know/ Don't Remember88
Y4_05	***Immunization status of the child***<br>*Check health card (if available) and based on the immunization information given, fill up the following questions. If no card available, then ask mother of child.*					
	Which immunizations has the target child taken so far?	Child 1		Child 2		
	List of vaccination:	**From Health Card**	**From Mother**	**From Health Card**	**From Mother**	Response Code
Y4_05a	BCG					
Y4_05b	DPT 1					
Y4_05c	DPT 2					
Y4_05d	DPT 3					
Y4_05e	Hepatitis B 1					
Y4_05f	Hepatitis B 2					
Y4_05g	Hepatitis B 3					
Y4_05h	Penta (DPT, Hep-B, HIB) 1					Yes .... 1<br>No .... 2<br>N/A 3
Y4_05i	Penta (DPT, Hep-B, HIB) 2					
Y4_05j	Penta (DPT, Hep-B, HIB) 3					
Y4_05k	OPV 0 (at birth)					
Y4_05l	OPV (Polio) 1					
Y4_05m	OPV (Polio) 2					
Y4_05n	OPV (Polio) 3					
Y4_05o	Measles					
Y4_05p	Vitamin A					

Household Number: | | | | |

Child Illness and Use of Health Services

Serial	Question	Response								Code
		Child 1				Child 2				
		Child of < 24 months				Child of < 24 months				
		A1	A2	A3	A4	A1	A2	A3	A4	
		Fever	Cough/cold	Fast breathing/ short breathing	Diarrhea	Fever	Cough/cold	Fast breathing/ short breathing	Diarrhea	
Y4_06	Has (NAME) had any of the following symptoms in the past two weeks?									Yes .... 1<br>No .... 2>Y4_10<br>Don't Know .... 88>Y4_10
Y4_07	Did you ask for advice or seek treatment when the child had (name illness/symptom)?									Yes .... 1<br>No .... 2>>Y4_09

Household Number: | | | | |

Serial	Question	Response								Code
		Child 1				Child 2				
		Child of < 24 months				Child of < 24 months				
Y4_08	WHOM did you seek advice/medical help or treatment for this illness(Please specify each illness to the respondent) (multiple responses possible)<br><br>Please ask about the FIRST point of care and enter that in top box. Then ask about SECOND point of care, and enter that in second box, and then finally, ask about THIRD point of care and enter in third box.									Govt. Hospital/Upazila Health Complex .... 1<br>Doctor .... 2<br>Midwife/nurse .... 3<br>Govt. Heath Worker (FWA/HA).......... 4<br>MA/SACMO.......... 5<br>BRAC SS .... 6<br>BRAC SK .... 7<br>BRAC Pushti Kormi (PK)/IYCF Promoter....... 8<br>Others NGO Worker .... 9<br>TTBA.......... 10<br>TBA .... 11<br>Village Doctor.......... 12<br>Homeopath Doctor.......... 13<br>Kabiraj/Herbal healer.......... 14<br>Spiritual Healer .... 15<br>Pharmacy .... 16<br>Husband .... 17<br>Mother/Mother in law .... 18<br>Other Family members.......... 19<br>Neighbors/ Friends.......... 20<br>Private Hospital/ Clinic .... 21<br>Mother and Children Welfare Centre (MCWC) 22<br>Community Clinic.......... 23<br>Satellite Clinic/ EPI Center .... 24<br>Smiling Sun Clinic.......... 25<br>Self.......... 26<br>Nobody.......... 27<br>Other (specify) .... 77<br>Do Not Know/ Do Not Remember 88

Household Number: | | | | |

Serial	Question	Response								Code
		Child 1				Child 2				
		Child of < 24 months				Child of < 24 months				
Y4_081	Where did this care provider sit?									Government hospital .... 1<br>Upazila Health Complex .... 2<br>Private clinic .... 3<br>Pharmacy .... 4<br>MCWC........ 5<br>Community Clinic........ 6<br>Satellite clinic/EPI Center .... 7<br>Smiling Sun Clinic........ 8<br>Union Health and Family Welfare Center........ 9<br>NGO facility: (specify) .... 10<br>Doctor's Chamber .... 11<br>Private doctor (MBBS) .... 12<br>Village doctor........ 13<br>Home........ 14<br>Other (specify) .... 77<br>Don't know/cannot recall........ 88<br>Not applicable 99
Y4_08b	Did the care provider take the NAME's weight when they examined him/her?									Yes........ 1<br>No........ 2
Y4_08c	Did the care provider ask how NAME was being fed when they examined him/her?									Yes........ 1<br>No........ 2
Y4_08d	Did the care provider give you any advice about child feeding when they examined NAME?									Yes........ 1<br>No........ 2>> Y4_09
Y4_08e	What advice did the care provider give about feeding?									Breastfeed more........ 1<br>Feed other foods........ 2<br>Give the child formula or tinned milk........ 3<br>Give the child tonics or vitamins........ 4<br>Other, specify........9

Household Number:

Serial	Question	Response		Code
		Child 1	Child 2	
		Child of < 24 months	Child of < 24 months	
Y4_09	What did you give the child when he/she had diarrhea? (Select all that apply)			ORS(bought) ... 1<br>Zinc tablets ... 2<br>Antibiotics ... 3<br>Homemade ORS ... 4<br>Traditional medicine ... 5<br>Other ... 6<br>Nothing ... 7
Y4_09a	I'd like to know how much NAME was given to DRINK during the diarrhea (including breastmilk)? Was he/she given less than usual to eat, about the same amount, more than usual, or nothing to drink?			Nothing to drink ... 0<br>Much less ... 1<br>Somewhat less ... 2<br>About the same ... 3<br>More ... 4<br>Don't know ... 8
Y4_09b	I'd like to know how much NAME was given to EAT during the diarrhea (including breastmilk)? Was he/she given less than usual to eat, about the same amount, more than usual, or nothing to drink?			Nothing to eat ... 0<br>Much less ... 1<br>Somewhat less ... 2<br>About the same ... 3<br>More ... 4<br>Don't know ... 8
Y4_10	Was NAME ever treated in a health center or hospital because he/she was very malnourished?			Yes ... 1<br>No ... 2
Y4_11	Was NAME ever given special foods like this (SHOW PACKET OF RUTF) to take home and eat for a few weeks because he/she was very malnourished?			Yes ... 1<br>No ... 2>>Y4_12

Household Number: [ ][ ][ ][ ]

Serial	Question	Response		Code
		Child 1	Child 2	
		Child of < 24 months	Child of < 24 months	
Y4_11a	Where did you get this special food (SHOW PACKET OF RUTF)?	[ ]	[ ]	Govt. Hospital/Upazila Health Complex .... 1<br>Doctor .... 2<br>Midwife/nurse .... 3<br>Govt. Heath Worker (FWA/HA) .... 4<br>MA/SACMO .... 5<br>BRAC SS .... 6<br>BRAC SK .... 7<br>BRAC Pushti Kormi (PK)/IYCF Promoter .... 8<br>Others NGO Worker .... 9<br>TTBA .... 10<br>TBA .... 11<br>Village Doctor .... 12<br>Homeopath Doctor .... 13<br>Kabiraj/Herbal healer .... 14<br>Spiritual Healer .... 15<br>Pharmacy .... 16<br>Husband .... 17<br>Mother/Mother in law .... 18<br>Other Family members .... 19<br>Neighbors/ Friends .... 20<br>Private Hospital/ Clinic .... 21<br>Mother and Children Welfare Centre (MCWC) .... 22<br>Community Clinic .... 23<br>Satellite Clinic/ EPI Center .... 24<br>Smiling Sun Clinic .... 25<br>Self .... 26<br>Nobody .... 27<br>Other (specify) .... 77<br>Do Not Know/ Do Not Remember 88
Y4_12	Has NAME ever been given any medicines for deworming?	[ ]	[ ]	Yes .... 1<br>No .... 2>>Next module
Y4_13	When was the last time NAME was given a medicine for deworming?	[ ]	[ ]	[___] MONTHS AGO (*write 0 if less than 1 month ago)

Household Number: | | | | |

Serial	Question	Response		Code
		Child 1	Child 2	
		Child of < 24 months	Child of < 24 months	
Y4_14	Where did you get the medicine from?			Govt. Hospital/Upazila Health Complex .... 1<br>Doctor .... 2<br>Midwife/nurse .... 3<br>Govt. Heath Worker (FWA/HA) .... 4<br>MA/SACMO .... 5<br>BRAC SS .... 6<br>BRAC SK .... 7<br>BRAC Pushti Kormi (PK)/IYCF Promoter .... 8<br>Others NGO Worker .... 9<br>TTBA .... 10<br>TBA .... 11<br>Village Doctor .... 12<br>Homeopath Doctor .... 13<br>Kabiraj/Herbal healer .... 14<br>Spiritual Healer .... 15<br>Pharmacy .... 16<br>Husband .... 17<br>Mother/Mother in law .... 18<br>Other Family members .... 19<br>Neighbors/ Friends .... 20<br>Private Hospital/ Clinic .... 21<br>Mother and Children Welfare Centre (MCWC) .... 22<br>Community Clinic .... 23<br>Satellite Clinic/ EPI Center .... 24<br>Smiling Sun Clinic .... 25<br>Self .... 26<br>Nobody .... 27<br>Other (specify) .... 77<br>Do Not Know/ Do Not Remember 88

Household Number: | | | | |

Module Y5: Nutrition related Prenatal Care during Pregnancy with Youngest Child (Female)

Instructions: To be asked for all children under the age of 24 months

Serial no.	Question	Child of < 24 months		Response Code
		Child 1	Child 2	
Child's Member ID	Copy the child's Name and ID from module B	Name:____________________ Mem. ID	Name:____________________ Mem. ID	Name and Member ID
Mother's Member ID	Copy the child's mother's Name and ID from module B	Name: ____________________ Member ID		Name and Member ID<br>If not member of HH, then record 55
Father's Member ID	Copy the child's father's Name and ID from module B	Name: ______________________ Member ID		Name and Member ID<br>If not member of HH, then record 55
Y5_ 01	Did you receive any antenatal care (ANC) when you were pregnant with this child?			Yes.......................................1<br>No .... 2>>Y5_09

Household Number: | | | | |

Serial no.	Question	Child of < 24 months		Response Code
		Child 1	Child 2	
Y5_02	Whom did you see for antenatal care during this pregnancy?<br>(MULTIPLE RESPONSES, PROBE AND WRITE ALL MENTIONED)			Govt. Hospital/Upazila Health Complex ....1<br>Doctor...2<br>Midwife/nurse ....3<br>Govt. Heath Worker (FWA/HA) ....4<br>MA/SACMO ....5<br>BRAC SS ....6<br>BRAC SK...7<br>BRAC Pushti Kormi (PK)/IYCF Promoter ....8<br>Others NGO Worker..9<br>TTBA ....10<br>TBA..11<br>Village Doctor ....12<br>Homeopath Doctor ....13<br>Kabiraj/Herbal healer ....14<br>Spiritual Healer ....15<br>Pharmacy..16<br>Husband ....17<br>Mother/Mother in law..18<br>Other Family members ....19<br>Neighbors/ Friends ....20<br>Private Hospital/ Clinic ....21<br>Mother and Children Welfare Centre (MCWC) ....22<br>Community Clinic ....23<br>Satellite Clinic/ EPI Center..24<br>Smiling Sun Clinic ....25<br>Self ....26<br>Nobody...27<br>Other (specify)...77<br>Do Not Know/ Do Not Remember 88
Y5_ 03	How many antenatal care sessions/check-ups did you attend when you were pregnant with this child?			Never..0<br>If attended, record exact number of sessions

Household Number: [ ][ ][ ][ ]

Serial no.	Question	Child of < 24 months		Response Code
		Child 1	Child 2	
Y5_ 04	Where all did you attend these sessions? Anywhere else? PROBE for all responses.<br><br>If antenatal sessions attended "0" zero time then write "99=N/A"<br><br>(Multiple Response)			Government hospital ....1<br>Upazila Health Complex ....2<br>Private clinic....3<br>Pharmacy....4<br>MCWC....5<br>Community Clinic ....6<br>Satellite clinic/EPI Center....7<br>Smiling Sun Clinic ....8<br>Union Health and Family Welfare Center ....9<br>NGO facility: (specify)....10<br>Doctor's Chamber ....11<br>Private doctor (MBBS)....12<br>Village doctor....13<br>Home....14<br>Other (specify)....77<br>Don't know/cannot recall ....88<br>Not applicable 99
Y5_05	During your pregnancy with this child, how often was your weight measured?			Never.... 0<br>Once .... 1<br>2 times.... 2<br>3 times.... 3<br>4 times.... 4<br>5 times.... 5<br>6 times.... 6<br>More than 6 times.... 7
Y5_06	Were you advised on what to eat or how to cook your food during pregnancy?			Yes, on what to eat .... 1<br>Yes, on how to cook .... 2<br>Yes, on both eat and cook.... 3<br>No, I was not advised .... 4 >> Y5_08
Y5_07	What advise were you given about eating during pregnancy?			Eat more food .... 1<br>Take more rest.... 2<br>Take iron-folic acid tablets .... 3<br>Take calcium tablets .... 4<br>Other 5
Y5_08	How many Tetanus Toxoid (TT) vaccinations did you have during your pregnancy with this child? [*A TT vaccination is an injection in your arm to prevent the baby from getting tetanus]*			None.... 0<br>One.... 1<br>Two .... 2<br>Don't know/cannot remember 8
Y5_09	Did you take iron/IFA tablets/supplements during your pregnancy with this child? SHOW IRON TABLET			Yes .... 1<br>No.... 2 >> Y5_13<br>Don't know/cannot remember .... 8 >> Y5_13

Household Number: | | | |

Serial no.	Question	Child of < 24 months		Response Code
		Child 1	Child 2	
Y5_10	Where did you get the iron/IFA tablets?<br><br>(multiple responses)			Government hospital ....1<br>Upazila Health Complex ....2<br>Private clinic....3<br>Pharmacy....4<br>MCWC....5<br>Community Clinic ....6<br>Satellite clinic/EPI Center....7<br>Smiling Sun Clinic ....8<br>Union Health and Family Welfare Center ....9<br>NGO facility: (specify)....10<br>Doctor's Chamber ....11<br>Private doctor (MBBS)....12<br>Village doctor....13<br>Home....14<br>Other (specify)....77<br>Don't know/cannot recall ....88<br>Not applicable 99
Y5_11	From which month of pregnancy did you start taking iron tablets/supplements?			Month
Y5_12	During your pregnancy for this child, for how many months did you take iron tablets or supplements so far?			Number of Months iron supplement taken<br>If less than 1 month write 1.<br>Don't know/cannot remember 88
Y5_13	Did you take any CALCIUM tablets/supplements during your pregnancy with this child? SHOW CALCIUM TABLET			Yes .... 1<br>No.... 2 >> Y5_17<br>Don't know/cannot remember .... 8 >> Y5_17
Y5_14	Where did you get the calcium tablets?<br>(multiple responses)			Government hospital ....1<br>Upazila Health Complex ....2<br>Private clinic....3<br>Pharmacy....4<br>MCWC....5<br>Community Clinic ....6<br>Satellite clinic/EPI Center....7<br>Smiling Sun Clinic ....8<br>Union Health and Family Welfare Center ....9<br>NGO facility: (specify)....10<br>Doctor's Chamber ....11<br>Private doctor (MBBS)....12<br>Village doctor....13<br>Home....14<br>Other (specify)....77<br>Don't know/cannot recall ....88<br>Not applicable 99

Household Number: | | | | |

Serial no.	Question	Child of < 24 months		Response Code
		Child 1	Child 2	
Y5_15	From which month of pregnancy did you start taking calcium tablets/supplements?			Month
Y5_16	During your pregnancy for this child, for how many months did you take calcium tablets or supplements so far			Number of Months iron supplement taken<br>If less than 1 month write 1.<br>Don't know/cannot remember 88
Y5_17	Were you given a vitamin A capsule after delivery? *This is usually given soon (within a few weeks) after delivery.*			Yes ....1<br>No..2<br>Don't know/cannot recall 88
Y5_18	Place of delivery for this child?<br><br>(in case of govt. or private facility specify where it was)			Government hospital ....1<br>Upazila Health Complex ....2<br>Private clinic....................3<br>Pharmacy....................4<br>MCWC....................5<br>Community Clinic....................6<br>Satellite clinic/EPI Center ....7<br>Smiling Sun Clinic ....8<br>Union Health and Family Welfare Center ....9<br>NGO facility: (specify)....................10<br>Doctor's Chamber ....11<br>Private doctor (MBBS)....................12<br>Village doctor....................13<br>Home....................14<br>Other (specify)....................77<br>Don't know/cannot recall ....88<br>Not applicable 99
Y5_19	Geographic place of delivery			Locally (home, facility)....................1<br>In mother's natal/family area....................2<br>Others (specify).................................. 3
Y5_20	Who helped with the delivery of this child?			Doctor/nurse/ midwife....................1<br>Govt. Heath Worker (FWA/HA) ....2<br>Trained Birth Attendant (TTBA)....................3<br>Traditional Birth Attendant (TBA)....................4<br>Village doctor....................5<br>NGO worker....................6<br>Relative(s) ....7<br>Neighbors/ Friends ....8<br>Others (specify).................................. 9
Y5_21	How soon was the baby weighed after birth?			Within 24 hours....................1<br>Between 1-3 days ....2<br>Between 4 days and 1 week....................3<br>In the second week. ....4<br>After 2 weeks ....5<br>Weight not taken .... 6 >> NEXT MODULE

Household Number:

Serial no.	Question	Child of < 24 months		Response Code
		Child 1	Child 2	
Y5_22	Who weighed the baby?			Govt. Hospital/Upazila Health Complex ....1<br>Doctor...2<br>Midwife/nurse ....3<br>Govt. Heath Worker (FWA/HA) ....4<br>MA/SACMO ....5<br>BRAC SS ....6<br>BRAC SK..7<br>BRAC Pushti Kormi (PK)/IYCF Promoter ....8<br>Others NGO Worker..9<br>TTBA ....10<br>TBA..11<br>Village Doctor ....12<br>Homeopath Doctor ....13<br>Kabiraj/Herbal healer ....14<br>Spiritual Healer ....15<br>Pharmacy...16<br>Husband ....17<br>Mother/Mother in law..18<br>Other Family members ....19<br>Neighbors/ Friends ....20<br>Private Hospital/ Clinic ....21<br>Mother and Children Welfare Centre (MCWC) ......22<br>Community Clinic ....23<br>Satellite Clinic/ EPI Center......................................24<br>Smiling Sun Clinic ....25<br>Self ....26<br>Nobody..27<br>Other (specify)...77<br>Do Not Know/ Do Not Remember 88
Y5_23	What was the weight of the baby (check card if possible)?			Weight in kg<br><br>Do Not Know/ Do Not Remember 88

Household Number: | | | | |

Module Y7: NNP Services Usage by Children under 2 years of age (Female)

To be asked for all children under the age of 24 months

Serial no.	Question	Child of < 24 months		Response Code
		Child – 1	Child – 2	
Child's Member ID	Copy the child's Name and ID from module B	Name:_______________ ____ Mem. ID	Name:_______________ ____ Mem. ID	Name and Member ID
Mother's Member ID	Copy the child's mother's Name and ID from module B	Name: ___________________ Member ID		Name and Member ID<br>If not member of HH, then record 55
Father's Member ID	Copy the child's father's Name and ID from module B	Name: _____________________ Member ID		Name and Member ID<br>If not member of HH, then record 55
Y7_ 01	Does your child [NAME] have a growth card?	weight in kg<br>age in months	weight in kg<br>age in months	Yes (if so check card and note weight and age in months at which last growth monitoring was done)<br>have card but weight and age not written.........88<br>No 99
Y7_ 02	Has NAME's weight ever been taken at a clinic or anywhere else?			Yes...1<br>No .... 2>> Y7_ 04
Y7_ 03	Where was NAME's weight taken?			Government hospital...................................... 1<br>Upazila Health Complex............................... 2<br>Private clinic... 3<br>Pharmacy .... 4<br>MCWC .... 5<br>Community Clinic .... 6<br>Satellite clinic/EPI Center............................. 7<br>Smiling Sun Clinic... 8<br>Union Health and Family Welfare Center ....9<br>NGO facility: (specify) .... 10<br>Doctor's Chamber .... 11<br>Private doctor (MBBS) .... 12<br>Village doctor .... 13<br>Home .... 14<br>Other (specify)... 77<br>Don't know/cannot recall............................... 88<br>Not applicable.............................99

Household Number: | | | | |

Serial no.	Question	Child of < 24 months		Response Code
		Child – 1	Child – 2	
Y7_ 04	Has NAME's height ever been taken at a clinic or anywhere else?			Yes .... 1<br>No .... 2>> Y7_ 06
Y7_ 05	Where was NAME's height taken?			Government hospital .... 1<br>Upazila Health Complex .... 2<br>Private clinic .... 3<br>Pharmacy .... 4<br>MCWC .... 5<br>Community Clinic .... 6<br>Satellite clinic/EPI Center .... 7<br>Smiling Sun Clinic .... 8<br>Union Health and Family Welfare Center9<br>NGO facility: (specify) .... 10<br>Doctor's Chamber .... 11<br>Private doctor (MBBS) .... 12<br>Village doctor .... 13<br>Home .... 14<br>Other (specify) .... 77<br>Don't know/cannot recall .... 88<br>Not applicable 99
Y7_ 06	Has NAME's arm circumference ever been taken at a clinic or anywhere else? Arm circumference is where the upper arm is measured using a tape			Yes .... 1<br>No .... 2>> NEXT MODULE

Household Number: | | | | |

Serial no.	Question	Child of < 24 months		Response Code
		Child – 1	Child – 2	
Y7_ 07	Where was NAME's arm circumference taken?<br><br>[if never attended put not applicable=99]			Government hospital .... 1<br>Upazila Health Complex .... 2<br>Private clinic .... 3<br>Pharmacy .... 4<br>MCWC .... 5<br>Community Clinic .... 6<br>Satellite clinic/EPI Center .... 7<br>Smiling Sun Clinic.. 8<br>Union Health and Family<br>Welfare Center... 9<br>NGO facility: (specify) .... 10<br>Doctor's Chamber .... 11<br>Private doctor (MBBS) .... 12<br>Village doctor .... 13<br>Home .... 14<br>Other (specify) .... 77<br>Don't know/cannot recall............................... 88<br>Not applicable.............................99
Y7_ 08	What was done after NAME's arm circumference was measured?			Nothing .... 1<br>I was given advise about food........................ 2<br>Child was given special foods to take home ... 3<br>Child was referred to treatment center .... 4<br>OTHER (specify).......................... 7

Household Number: | | | | |

Module Y8: Exposure to Nutrition Information from Health Workers and Media (Female)

Question Number	Questions	Response	Response Code
**Y8_01**	Have you been visited at home by <u>any health worker in the last SIX months?</u>		Yes........1<br>No........2>> Y8_06<br>Don't know........88>> Y8_06
**Y8_02**	<u>In the last SIX months</u>, did any health worker from anywhere or any organization visit you at home?		Government Health Worker (FWA/HA) ....1<br>SMC/Other Health Worker........2<br>BRAC Worker (SK/SS/PK etc.) ....3<br>Other NGO worker........4<br>Other (Specify) 5
**Y8_03**	How many times in the last SIX months were you visited at home by a health worker?		Number of times<br>Don't remember 88
**Y8_04**	During the last visit, did you receive any advice from the health worker on feeding your child?		Yes........1<br>No........2
**Y8_01a**	Have you been visited at home by <u>any health worker in the last THREE months?</u>		Yes........1<br>No........2>> Y8_06<br>Don't know........8>> Y8_06
**Y8_02a**	In the last THREE months, did any health worker from anywhere or any organization visit you at home?		Government Health Worker (FWA/HA) ....1<br>SMC/Other Health Worker........2<br>BRAC Worker (SK/SS/PK etc.) ....3<br>Other NGO worker........4<br>Other (Specify) 5
**Y8_03a**	How many times in the last THREE months were you visited at home by a health worker?		Number of times<br>Don't remember 88
**Y8_04a**	During the last visit (within the last THREE months), did you receive any advice from the health worker on feeding your child?		Yes........1<br>No........2>> Y8_06
**Y8_05**	What advice did you receive from the health worker on feeding your child?		Putting baby to breast immediately after birth........1<br>Giving only colostrum........2<br>No pre or post lacteals (honey/mustard oil/glucose water) ....3<br>Feed only breast milk up to six Months........4<br>Positioning........5<br>Attachment ....6<br>Feeding mashed family food after 6 months ....7<br>Feeding animal source foods ....8<br>Cooking/adding with oil ....9<br>Adding Sprinkles........10<br>Washing hands with water and soap before prep/feeding child........11<br>Feeding during illness/extra after illness ....12<br>Other (specify)........13<br>Respondent did not mention any of the above on her own 99

Household Number: | | | | |

**Y8_06**	Have you ever attended any group meetings/discussions where health or nutrition issues were discussed?		Yes....1<br>No....2>> Y8_11
**Y8_07**	Last time you attended a group discussion about health/nutrition, what was discussed?		Family planning....1<br>Water and sanitation....2<br>Immunization....3<br>Maternal nutrition....4<br>TB....5<br>Breastfeeding....6<br>Complementary feeding ....7<br>Sprinkles....8<br>Washing hands with water and soap before prep/ feeding child....9<br>Encouraging child to eat enough ....10<br>Feeding during illness/extra after illness ....11<br>Other (specify)....12<br>Respondent did not mention any of the above on her own 99

Module Y6a: Access to Community Clinics and Use of Community Clinic (Female)

Question Number	Question	Response			Response Code
	Access to Community Clinic	a	b	c	
**Y6a_ 01**	Have you heard about the Community Clinic?				Yes....1<br>No....2 >> Next Module
**Y6a_ 02**	How long does it take you to walk to the Community Clinic in your area?				MINUTES<br><br>Not walkable....999
**Y6a_ 03**	Is there anyone in this household who has used the Community Clinic and obtains/obtained services there?				Yes....1<br>No....2 >> Next Module
**Y6a _04**	Who in your household has used the Community Clinic?				Put member ID from roster
**Y6a_ 05**	What types of services at Community Clinic were used for this person?<br><br>(multiple responses allowed)				Sick child care ....1<br>Antenatal care....2<br>Delivery care....3<br>Postnatal care....4<br>Family planning services ....5<br>Received IFA tablets during pregnancy...6<br>Received vitamin A for children....7<br>Child was weighed....9<br>Sick care (for adult) ....10<br>Vaccination....11

Module End Time: Hour | | | Minute | | |

Household Number: | | | | |

Module Z: Women's Status (Female)

Module start time: Hour | | | Minu | | | Respondent ID: | | Consent : | | Yes ...1 No....2

Sl no	Question	Response	Response Code
**Z_1**	Member ID of Primary Female Household Member		Member ID
**Z_2**	Marital Status of Primary Female Household Member		Never Married ..... 1<br>Married, lives with husband..... 2<br>Married, does not live with husband..... 3<br>Widow ..... 4<br>Divorced ..... 5<br>Separated/Deserted 6

Module Z1: Work Earnings and Expenses (Female)

Question Number	Question	Response	Response Code
**Z1_01**	Are you now doing any work or business that brings in cash, additional food, or allows you to accumulate assets for your household? (***Note: if the woman says no, check she does not engage in agriculture, petty trade, money lending, etc.)***		Yes ..... 1 >>Z1_05<br>No..... 2
**Z1_02**	If not, why not?		Husband / in-laws won't allow ..... 1<br>Society doesn't like it ..... 2<br>Take care of children/household work ..... 3>>Z1_12<br>I don't want to ..... 4>>Z1_12<br>I don't need to..... 5>>Z1_12<br>Am unable to work ..... 6>>Z1_12<br>Lack of demand for the work that I have skill to do..... 7>>Z1_12<br>Other (specify)..... 8>>Z1_12<br>Don't know..... 88>>Z1_12
**Z1_03**	Why won't your husband or your in-laws, or society let you work?		Believes women should not work to earn income..... 1<br>Household has enough income ..... 2<br>Wants me to look after household work..... 3<br>Doesn't want me to mingle with other men ..... 4<br>Other (specify)..... 5<br>Don't know/ Don't remember..... 88
**Z1_04**	Would your husband or in-laws allow you to engage in a business or earn additional money from working inside your home?		Yes ..... 1<br>No..... 2>>Z1_12
**Z1_05**	Where do you work to earn income?		Inside the home..... 1<br>Outside ..... 2<br>Both..... 3

Household Number: ☐☐☐☐

Question Number	Question	Response	Response Code
**Z1_06**	Whose decision was it to work to earn income?		Yourself ..... 1<br>Your husband ..... 2<br>Self and husband ..... 3<br>Someone else (specify) 4
**Z1_07**	At first did your husband or other household member want to prevent you from working to earn money?		Yes ..... 1<br>No ..... 2>>Z1_09
**Z1_08**	If yes, why so?		Believes women should not work ..... 1>>Z1_10<br>Household has enough income ..... 2>> Z1_10<br>Wants me to look after household work ..... 3>> Z1_10<br>Doesn't want me to mingle with other men ..... 4>> Z1_10<br>Other (specify) ..... 5>> Z1_10<br>Don't know/ Don't remember ..... 6 >>Z1_10
**Z1_09**	If no, why not?		Husband/other household members unable to work ..... 1<br>Money/food was short ..... 2<br>Other (specify 3
**Z1_10**	What do you do with the money you earn?		Give it all to my husband / other family member ..... 1<br>Give some to husband / other member ..... 2<br>Keep all 3
**Z1_11**	Who usually decides how to spend the money you earn?		Yourself ..... 1<br>Your husband ..... 2<br>Self and husband ..... 3<br>Someone else (specify) 4
**Z1_12**	Have you ever taken any loans from an NGO?		Yes ..... 1<br>No 2>>Z1_15
**Z1_13**	Whose decision was it to take the loan from the NGO?		Yourself ..... 1<br>Your husband ..... 2<br>Self and husband ..... 3<br>Someone else (specify) 4
**Z1_14**	Who usually decides how to spend the money from the loan?		Yourself ..... 1<br>Your husband ..... 2<br>Self and husband ..... 3<br>Someone else (specify) 4
**Z1_15**	*Who decides how to spend money on the following items?*		

Household Number: | | | | |

Question Number	Question	Response	Response Code
**Z1_15a**	Food		Yourself .... 1<br>Your husband .... 2<br>Self and husband .... 3<br>Someone else (specify) .... 4<br>Not applicable 9
**Z1_15b**	Housing		
**Z1_15c**	Health Care		
**Z1_15d**	Education		
**Z1_15e**	Clothing		
**Z1_16**	*Do you yourself control the money needed to buy the following?*		
**Z1_16a**	Food from the market		Yes .... 1<br>No 2
**Z1_16b**	Clothes for yourself		
**Z1_16c**	Medicine for yourself		
**Z1_16d**	Toiletries/cosmetics for yourself		

Household Number: | | | | |

Module Z2: Freedom of Mobility (Female)

Place code	Places	Who decides whether you can go by yourself to the following places?<br><br>Yourself. .... 1<br>Your husband .... 2<br>Self and husband .... 3<br>Others (specify).......................... 4<br>Not applicable 5	If your husband or other household member objects, in what circumstances would they allow you to go?<br><br>If I have company (relatives, children) .... 1<br>If I can arrange my own expenses............................ 2<br>If I follow purdah / dress acceptably........................ 3<br>Other (specify) .... 4<br>Not applicable/would not object if I go alone 5
		Code ↑	Code ↑
**Z2_01**	Outside the community to visit friends or relatives		
**Z2_02**	Haat/Bazaar		
**Z2_03**	Hospital/Clinic/Doctor		
**Z2_04**	Cinema/Fair/Theatre		
**Z2_05**	Training for NGO/Programs		

Module End Time: | Hour | | | Minute | | |

Household Number: | | | |

Module Z3: Reproductive Decisions (Female)

To be asked to all the married females between the ages of 10 and 49 years

Module start time: Hour | | | Minu | | | Respondent ID: | | Consent : | | Yes ...1 No.....2

Question Number	Question	Response					Response Code
		**MID**	**MID**	**MID**	**MID**	**MID**	
Respondent Consent	Do you agree to answer the following question about your reproductive decisions?						Yes .... 1<br>No........ 2>>Next Module
**Z3_01**	Have you ever used birth control (methods to delay or avoid pregnancy?)						Yes .... 1<br>No........ 2>>Z3_03
**Z3_01a**	Which method did you use?						Birth control pill .... 1<br>IUD (e.g. Coper T) .... 2<br>Implant/Norplant .... 3<br>Injection/Somaject (to delay pregnancy by a few months) .... 4<br>Injection/DepoProvera(to delay pregnancy by a few months) ...... 5<br>Ligation/Tubectomy .... 6<br>Safe days/ Temperature Control .... 7<br>Others .... 8<br>Do not want to respond……….88
**Z3_02**	Who made this decision?<br>(After filling this question's response skip to Z3_04)						Yourself........1 >> Z3_04<br>Your husband ....2>> Z3_04<br>Self and husband........3>> Z3_04<br>Someone else (specify) ....4>> Z3_04
**Z3_03**	Why have you not used birth control?						Husband didn't allow........ 1<br>Makes me feel weak / ill........ 2<br>Didn't feel the need to .... 3<br>Other (specify) 4
**Z3_04**	Has your husband ever used any birth control method?						Yes .... 1<br>No........2 >> Z3_05<br>Not applicable........3 >> Z3_05
**Z3_04a**	Which method did your husband use?						Condom .... 1<br>Vasectomy........ 2<br>Others(specify) 3
**Z3_05**	If Z3_01=2 and Z3_04=2 or 3, please confirm if the man was sterilized?						Yes .... 1<br>No........ 2
**Z3_06**	If Z3_01=2 and Z3_04=2 or 3, please confirm if the woman was sterilized?						Yes .... 1<br>No........ 2

Household Number: | | | | |

Module Z4: Domestic Violence, Abuse and Threats (Female)

To be asked to all the married females aged between 10 and 49 years

Question Number	Question	Response					Response Code
		**Member 1**	**Member 2**	**Member 3**	**Member 4**	**Member 5**	
		**Member Name**<br><br>**MID**	**Member Name**<br><br>**MID**	**Member Name**<br><br>**MID**	**Member Name**<br><br>**MID**	**Member Name**<br><br>**MID**	
**Respondent Consent**	Do you agree to answer the following question about domestic violence?						Yes....................1<br>No....................2>>Next Module
**Z4_01**	Has any of the following happened to you in the past year?						
**Z4_01a**	Your husband threatened you with divorce?						Yes, often.................... 1<br>Yes, sometimes.................... 2<br>Has threatened and has divorced/remarried .... 3<br>No.................... 4<br>Not applicable.................... 9<br>Do not want to respond………………….88
**Z4_01b**	Your husband threatened you with taking another wife?						
**Z4_01c1**	Your husband verbally abused you?						Yes, often.................... 1<br>Yes, sometimes.................... 2<br>Has threatened and has divorced/remarried .... 3<br>No.................... 4<br>Not applicable.................... 9<br>Do not want to respond………………….88
**Z4_01c2**	Any other adult male of your household verbally abused you?						
**Z4_01c3**	Any adult female of your household verbally abused you?						
**Z4_01d1**	Your husband physically abused you?						Yes, often.................... 1<br>Yes, sometimes.................... 2<br>Has threatened and has divorced/remarried .... 3<br>No.................... 4<br>Not applicable.................... 9<br>Do not want to respond………………….88

Household Number: | | | |

Question Number	Question	Response					Response Code
**Z4_01e1**	If Z4_01d1 if 1 or 2, injuries from physical assault?<br><br>(multiple responses allowed)						Cuts=1<br>Bruises=2<br>Ache=3<br>Eye injury=4<br>Sprain=5<br>Dislocation=6<br>Burn=7<br>Deep wound=8<br>Broken bones=9<br>Broken teeth=10<br>Any other serious injury=11
**Z4_01d2**	Any other adult male of your household physically abused you?						Yes, often... 1<br>Yes, sometimes... 2<br>Has threatened and divorced/remarried.................. 3<br>No.. 4<br>Not applicable....................9<br>Do not want to respond………………….88
**Z4_01e2**	If Z4_01d2 if 1 or 2, injuries from physical assault?<br><br>(multiple responses allowed)						Cuts=1<br>Bruises=2<br>Ache=3<br>Eye injury=4<br>Sprain=5<br>Dislocation=6<br>Burn=7<br>Deep wound=8<br>Broken bones=9<br>Broken teeth=10<br>Any other serious injury=11
**Z4_01d3**	Any adult female of your household physically abused you?						Yes, often... 1<br>Yes, sometimes... 2<br>Has threatened and divorced/remarried.................. 3<br>No.. 4<br>Not applicable....................9<br>Do not want to respond………………….88
**Z4_01e3**	If Z4_01d3 if 1 or 2, injuries from physical assault?						Cuts=1<br>Bruises=2<br>Ache=3<br>Eye injury=4<br>Sprain=5<br>Dislocation=6<br>Burn=7<br>Deep wound=8<br>Broken bones=9<br>Broken teeth=10<br>Any other serious injury=11
**Z4_05**	Were you ever threatened to leave the house?						Yes... 1<br>No.. 2

Household Number: | | | | |

Question Number	Question	Response					Response Code
**Z4_06**	Were you ever forcefully sent out of the house?						Yes.. 1<br>No... 2
**Z4_07**	If yes, who brought you back?						Husband….1<br>Other adult male member of hh……2<br>Other adult female hh member…..3<br>Came back myself……4<br>Did not come back……5<br>Do not want to respond=88
**Z4_08**	If the answer to Z4_07 is 4, why did you come back yourself?						He was angry and didn't mean it .... 1<br>My husband and I came to an agreement............... 2<br>I could not support myself financially.................... 3<br>My parents could not support me financially......... 4<br>I came back for my children .... 5<br>Because of social pressure ..6<br>Do not want to respond=88
If response to question Z4_01a to Z4_01d3 is either 4 or 9, then skip to Z4_09							
**Z4_02**	If any answer to Z4_1a to Z4_1d3 is 1, 2 or 3, did you want to leave?						Yes.. 1<br>No..2 >>Z4_09
**Z4_03**	Did you leave?						Yes, permanently...1>>Z4_09<br>Yes, but I came back... 2<br>No...3
**Z4_04**	If you did not leave permanently, why not?						He was angry and didn't mean it .... 1<br>My husband and I came to an agreement............... 2<br>I could not support myself financially.................... 3<br>My parents could not support me financially......... 4<br>I came back for my children .... 5<br>Because of social pressure .... 6<br>Do not want to respond....................................... 88
**Z4_09**	Does your husband drink alcohol?						Yes .... 1<br>No .... 2<br>Not applicable (husband is deceased) .... 3<br>Do not want to respond 88
**Z4_10**	Does your husband consume drugs?						Yes .... 1<br>No .... 2<br>Not applicable (husband is deceased) .... 3<br>Do not want to respond 88
**Z4_09a**	If your husband is not alive, did he drink alcohol when he was alive?						Yes .... 1<br>No .... 2<br>Do not want to respond 88
**Z4_10a**	If your husband is not alive, did he consume drugs when he was alive?						Yes .... 1<br>No .... 2<br>Do not want to respond 88

Module End Time: | Hour | | | Minute | | |

Household Number: | | | | |

Module Z5: Wife's Assets that had been brought to Marriage (Female)

Module start time: | Hour | | | Minu | | | **Respondent ID:** | | Consent : | | Yes ...1 No.....2

Question Number	Question	Response	Response Code
Z5_01	When you married, did you bring any assets from your parent's home?		Yes..........................1>>go to next question<br>No...........................2→ End module

Name of asset brought at the time of marriage	Asset (see Asset Code below)	Value of asset at time of marriage (Tk)
**Name**	**Z5_02**	**Z5_03**

Asset Codes

Consumption Assets (for household use)
Homestead (excluding land)............................1
Large tree 2
Trunk/Suitcase 3
Bucket/Pots 4
Stove/Gas burner 5
Metal cooking pots..6
Bed/Khat/Chowki..7
Armoire/Cabinet/Alna 8
Table/chair 9
Fans/Iron 10
Radio/Cassette player 11
Wall clock/Watch...12
TV/VCR/CD 13
Refrigerator 14
Jewelry (gold/silver).......................................15
Sewing machine 16
Bicycle 17
Rickshaw..18
Motorcycle 19
Other motor vehicle..20
Mobile telephone / phone...............................21
Hand tube well 22
Livestock (for own consumption)...................23
Poultry (for own consumption) 24
Cash 25
Mattress..26
Quilt 27
Pillow 28
Other assets (list)..29

Productive Assets (for earning income)
Sewing machine 51
Rickshaw / van 52
Mobile phone / phone.....................................53
Fishing net..54
Iron...55
Hand tube well 56
Irrigation equipment, including pump............57
Boat..58
Livestock..59
Poultry..60
Other agricultural equipment..........................61
Other vehicles 62
Charcoal maker 63
Other production assets (list)..........................64

Module End Time: | Hour | | | Minute | | |

Household Number: | | | | |

Module WE: Women's Empowerment in Agriculture (WEAI) Index (Male and Female)

Bangladesh: Individual Level Questionnaire
International Food Policy Research Institute (IFPRI) & Data Analysis and Technical Assistance Limited (DATA)

INSTRUCTIONS ON ADMINISTRATION:

Enumerator: This questionnaire should be administered to individuals identified in the household roster (Module B) of the household level questionnaire as the primary male and primary female respondents.
You should complete this coversheet for each individual identified in the "selection section" even if the individual is not available to be interviewed for reporting purposes. Please double check to ensure:

- You have completed the household questionnaire, at least the first 2 modules;
- You have identified the correct individual;
- You have noted the household ID and individual ID correctly for the person you are about to interview;
- You have gained informed consent for the individual in the household questionnaire;
- You have sought to interview the individual in private or where other members of the household cannot overhear or contribute answers.

Module start time: Hour | | | Minu | | | Respondent ID: | | Consent : | | Yes ...1 No.....2

Module WEA: Individual Identification (Male and Female)

Household Identification	Code
WA01. Household Identification:	
WA02. Census number:	
WA03. Name of primary respondent (code from roster in Module B): Last, First:	
WA04. Name of respondent (code from roster in Module B of household questionnaire): Last, First:	
WA05. Sex of respondent: Male.........1 Female 2	
WA06. Type of household (enter code from Code 1↓):	

Interview details	Code
WA07. Start time of interview (hh:mm => write in 24 hr time format)	:
WA08. End time of interview (hh:mm => write in 24 hr time format)	:
WA09. Name/code of enumerator: …………………………………………………………………..	
WA10. Sex of enumerator: Male .... 1 Female..... 2	
WA11. Outcome of interview (enter code from Code 2↓):	
WA12. Ability to be interviewed alone (enter code from Code 3↓):	

Household Number: | | | | |

Code list for Module WEA:

Code 1 (WA06): Type of Household:	Code2 (WA11): Outcome of interview	Code 3 (WA12): Ability to be interviewed alone
Male and Female adult .... 1<br>Female, no Male adult................................. 2<br>Male, no Female adult................................. 3<br>No adult 4	Completed...1<br>Incomplete ....2<br>Absent..3<br>Refused...4<br>Could not locate...5	Alone ....1<br>With other adult females present ....2<br>With other adult males present ....3<br>With other adults mixed sex present ....4<br>With children present....................................5

Module WE2: (Dimension 1): Role in household decision-making around production and income generation (Male and Female)

Enumerator: The purpose of this module is to get an idea about men's and women's relative roles in decision making around income-generating activities. Do not attempt to ensure that responses are the same between the male and female respondent. It is okay for them to be different.

Code 1: Input into decision making
No input/No opinion at all....................1
Very little input...................................2
Input into some decisions.....................3
Input into most decisions/ Input into all decisions 4
Decision not made/not applicable 5

Activity		Did you (singular) participate in [ACTIVITY] in the past 12 months?<br>Yes.........1<br>No ....2 >> next activity	How much input did you have in making decisions about [ACTIVITY]?<br>**CODE 1↑**	How much input did you have in decisions on the use of income generated from [ACTIVITY]<br>**CODE 1↑**
Activity Code	Activity Description	**WE201**	**WE202**	**WE203**
**A**	Food crop farming: crops that are grown primarily for household food consumption			
**B**	Cash crop farming: crops that are grown primary for sale in the market			
**C1**	Large livestock (cow, oxen, cattle)			
**C2**	Small livestock (goats, pigs, sheep)			
**C3**	Poultry (chicken, duck, pigeons)			

Household Number: | | | |

D	Non-farm economic activities: small business, self-employment, buy-and-sell			
E	Wage and salary employment: in-kind or monetary work both agriculture and other			
F	Fishing or fish culture			

Module WE3a: (Dimension 2) Access to productive capital (Male and Female)

Now we would like to know about your household's ownership/use of productive assets.

Enumerator: The purpose of this module is to get an idea about men's and women's access to capital or assets and their ability to control use of the resource. Again, do not attempt to ensure that responses are the same between the male and female respondent. It is okay for them to be different.

**If the number of the shared in land is higher, the person who uses or who have the opportunity to use land should be treated as owner.

Productive Capital		Does anyone in your household currently have this [ITEM]?<br><br>Yes ...1<br>No.......2 >> next item	How many of [ITEM] does your household currently have?<br><br>[number]	Who would you say owns most of the [ITEM]?<br><br>CODE 1↓<br><br>(For joint decisions there could be multiple responses)			Who would you say can decide whether to sell [ITEM] most of the time?<br><br>CODE 1↓<br><br>(For joint decisions there could be multiple responses)			Who would you say can decide whether to give away [ITEM] most of the time?<br><br>CODE 1↓<br><br>(For joint decisions there could be multiple responses)			Who would you say can decide to mortgage or rent out [ITEM] most of the time?<br>CODE 1↓<br>(For joint decisions there could be multiple responses)			Who contributes most to decisions regarding a new purchase of [ITEM]?<br><br>CODE 1↓<br>(For joint decisions there could be multiple responses)			Do you own any of the item?<br><br>Yes, Solely…1<br>Yes, Jointly…2<br>Yes, Solely & Jointly…3<br>No…4<br><br>(multiple responses possible)
**Productive Capital**		**WE3a_01a**	**WE 3a_01b**	**WE 3a_02a**	**WE 3a_02 b**	**WE 3a_02 c**	**WE 3a_03a**	**WE 3a_03b**	**WE 3a_03c**	**WE 3a_04a**	**WE 3a_04 b**	**WE 3a_04 c**	**WE 3a_05a**	**WE 3a_05 b**	**WE 3a_05 c**	**WE3a_ 06a**	**WE3 a_06b**	**WE3 a_06c**	**WE3a_07**
**A**	Agricultural land (pieces/polts)**																		
**B**	Large livestock (oxen, buffalo)																		
**C**	Small livestock (goats, sheep)																		

Household Number: |__|__|__|__|

**D**	Chickens, Ducks, Turkeys, Pigeons																		
**E**	Fish pond or fishing equipment																		
**F**	Farm equipment (non-mechanized)																		
**G**	Farm equipment (mechanized)																		
**H**	Nonfarm business equipment																		
**I**	House (and other structures)																		
**J**	Large consumer durables (fridge, TV, sofa)																		
**K**	Small consumer durables (radio, cookware)																		
**L**	Cell phone																		
**M**	Other land not used for agricultural purposes (residential /commercial land)																		
**N**	Means of transportation (bicycle, rickshaw, motorcycle, car)																		

**CODE 1**		
Self..1<br>Spouse..2<br>Other household member.....................3<br>Member outside household..................4		Not applicable......................................98

Module WE3B: Agricultural Extension (Male and Female)

Q. No.	Question	Response	Response options
WE3B_07	Where do you typically get information on farming or livestock related topics such as new seeds, technology, crop rotation or animal health?		Government agency or outlet ....1<br>NGO or NGO outlet...............................2<br>Private shop/suppliers............................3<br>Community members or cooperative .....4<br>Family member......................................5<br>Media (radio/TV/newspaper) ....6<br>Not applicable/do not get advice............7
WE3B_08	Have you (yourself) ever met with an agricultural extension worker or livestock/fisheries extension worker in the past 12 months?		Yes..1<br>No .... 2 >> Next section

Household Number: | | | | |

WE3B_09	How many times did you meet with the agricultural extension worker or livestock/fisheries worker in the past 12 months?		[Enter number of visits]
WE3B_10	The last time you met with an extension worker, were they a male or female?		Male..1<br>Female ....2<br>Both male and female 3

Module WE3C: (Dimension 3) Income (Male and Female)

Enumerator: The purpose of this module is to get an idea about how the surplus of household, men's and women's incomes, after food needs are met, is allocated among other expenditure categories: Do not attempt to ensure that responses are the same between male and female respondent. It is okay for them to be different.

Q. No.	Question	Response	Response options/Instructions
WE3C_11	Do you alone have any money you can decide what to spend on?		Yes...1<br>No 2
WE3C_12	In comparison to your partner, do you; [READ RESPONSES]:<br><br>Enumerator: Skip this question if the respondent has no partner.		Earn more money than him/her.......................1<br>Earn less money than him/her ....2<br>Earn about the same money as him/her...........3<br>Partner does not earn money ....4<br>I do not earn money ....5<br>Both partner and I do not earn money.............6<br>Do not know how much partner earns.............7<br>Not Applicable...98
	Question: Household level		
WE3C_13	In the last 12 months, after providing food for the family, what did your household spend your remaining money on?<br><br>Enumerator: List up to 5 in order of importance<br>[DO NOT PROMPT]. If answer to WE3C_13 is from 1-20, go to WE3C_15	If answer to WE3C_13 is from 1-20, go to WE3C_15<br><br>A B C D E	Clothes/shoes for myself ....1<br>Clothes/shoes for my spouse...........................2<br>Clothes/shoes for my children.........................3<br>School fees of school supplies for children.....4<br>Consumer durables ....5<br>Fix or improve house ....6<br>Buy asset for farming ....7<br>Buy asset for business ....8<br>Buy jewelry..9<br>Services (beauty/hair/etc).............................10<br>Lend money to friends and relatives ....11<br>Make religious/ charitable donations ....12<br>Travel to visit friends/relatives......................13<br>Save for future ....14<br>Medicines or health needs.............................15<br>Communication (airtime)..............................16

			Buy consumables (toiletries, paraffin, etc.)...17<br>Social events (weddings/funerals/sports) ......18<br>Drinking alcohol and smoking......................19<br>Unknown how the money was spent ......20<br>Household or myself has no money .... 98 >>WE3C_14
WE3C_14	In the last 12 months, if the household did not have any remaining money, what is the reason?<br><br>Enumerator: List up to 3 in order of importance. Do not prompt.	A B C	Bad harvest ....1<br>Irregular income/business is slow ....2<br>Other negative shock (illness).........................3<br>Unexpected expenditures ....4<br>No control over extra money...........................5
	Question: Individual level		
WE3C_15	In the last 12 months, after providing food for the family, what did you (singular) spend your remaining money on?<br><br>Enumerator: List up to 5 in order of importance<br>Do not prompt.	If answer to WE3C_15 is from 1-20, go to Module WE3D<br><br>A B C D E	Clothes/shoes for myself ....1<br>Clothes/shoes for my spouse...........................2<br>Clothes/shoes for my children.........................3<br>School fees of school supplies for children .....4<br>Consumer durables ....5<br>Fix or improve house ....6<br>Buy asset for farming ....7<br>Buy asset for business ....8<br>Buy jewelry..9<br>Services (beauty/hair/etc).............................10<br>Lend money to friends and relatives ....11<br>Make religious/ charitable donations ....12<br>Travel to visit friends/relatives......................13<br>Save for future ....14<br>Medicines or health needs............................15<br>Communication (airtime).............................16<br>Buy consumables (toiletries, paraffin, etc.)...17<br>Social events (weddings/funerals/sports) ......18<br>Drinking alcohol and smoking......................19<br>Unknown how the money was spent.............20<br>Household or myself has no money….98>> WE3C_16
WE3C_16	In the last 12 months, if you (singular) did not have any remaining money, what is the reason?<br><br>Enumerator: List up to 3 in order of importance. Do not prompt	A B C	Bad harvest ....1<br>Irregular income/business is slow ....2<br>Other negative shock (illness).........................3<br>Unexpected expenditures ....4<br>No control over extra money...........................5

Household Number: | | | | |

Module WE3D: Access to loans (Male and Female)

Now I will ask you about any loans taken for the household in the last 12 months.

Lending sources		Has anyone in your household taken any loans or borrowed cash/in-kind from [SOURCE] in the past 12 months?<br><br>Yes, cash .... 1<br>Yes, in-kind............ 2<br>Yes, cash and in-kind .... 3<br>No ....4 >> next source<br>Don't know ....97 >> next source	Who made the decision to borrow from [SOURCE]?<br><br>Self ....1<br>Spouse ....2<br>Other household member............3<br>Other member outside the household.....4<br>Not applicable............98<br><br>For joint decisions, multiple responses are possible			Who makes the decision about what to do with the money/ item borrow from [SOURCE]?<br><br>Self............ 1<br>Spouse............ 2<br>Other household member .... 3<br>Other member outside the household .... 4<br>Not applicable .... 98<br><br>For joint decisions, multiple responses are possible1		
Lending source names		WE3d_17	WE3d _18a	WE3d _18b	WE3d _18c	WE3d _19a	WE3d _19b	WE3d _19c
A	Non-governmental organization (NGO)							
B	Informal lender							
C	Formal lender (bank/financial institution)							
D	Friends or relatives							
E	Non-institutional lending group, such as ROSCA (savings/credit group), merry-go-rounds, tontines etc.							

Household Number: | | | | |

Module WE4: Individual Leadership and Influence in the Community (Male and Female)

Enumerator: The purpose of this module is to get an idea about men's and women's potential for leadership and influence in the communities where they live. Again, do not attempt to ensure that responses are the same between the male and female respondent. It is okay for them to be different.

Now I have a few questions about how comfortable you feel speaking up in public when the community needs to make important decisions.

Q. No.	Question	Response
WE4.01	Do you feel comfortable speaking up in public to help decide on infrastructure (like small wells, roads, water supplies) to be built in your community?	No, not at all comfortable ....1<br>Yes, but with difficulty ....2<br>Yes, comfortably ....3<br>NOT APPLICABLE 98
WE4.02	Do you feel comfortable speaking up in public to ensure proper payment of wages for public works or other similar programs (such as EGPP, FFW)?	No, not at all comfortable ....1<br>Yes, but with difficulty ....2<br>Yes, comfortably ....3<br>NOT APPLICABLE 98
WE4.03	Do you feel comfortable speaking up in public to protest the misbehavior of authorities or elected officials?	No, not at all comfortable ....1<br>Yes, but with difficulty ....2<br>Yes, comfortably ....3<br>NOT APPLICABLE 98

Q. No.	Question	Response	Response options/Instructions
WE4.04	Do you feel that a [man / woman] like yourself can generally change things in the community where you live if s/he wants to?		No, not at all ....1<br>Yes, but with a great deal of difficulty...2<br>Yes, but with a little difficulty ....3<br>Yes, fairly easily ....4<br>Yes, very easily 5
WE4.05	In the last 12 months, have you		
WE4.05A	Contributed money or time to building small wells or maintenance of irrigation facilities in your community?		Yes... 1<br>No 2
WE4.05B	Contributed money or time to building or maintaining roads in your community?		Yes... 1<br>No 2
WE4.05C	Contributed money or time to town development projects or public works projects in your community?		Yes... 1<br>No 2
WE4.05D	Contributed money or time to building or maintaining your local mosque/church/temple?		Yes... 1<br>No 2
WE4.05E	In the last year, did you give money to any other family because someone in their family was sick?		Yes... 1<br>No 2
WE4.05F	In the last year, did you help another family out with agricultural labor?		Yes... 1<br>No 2
WE4.05G	In the last year, did you help another family out when they needed help with child care?		Yes... 1<br>No 2
WE4.06	Please imagine a nine-step ladder, where on the bottom, the first step, stand people who have NO influence on the community, and step 9, the highest step, stand those who have influence in the community. On which step are you?		[Enter step 1 – 9]<br>Enumerator show the ladder in the next page

Household Number: | | | | |

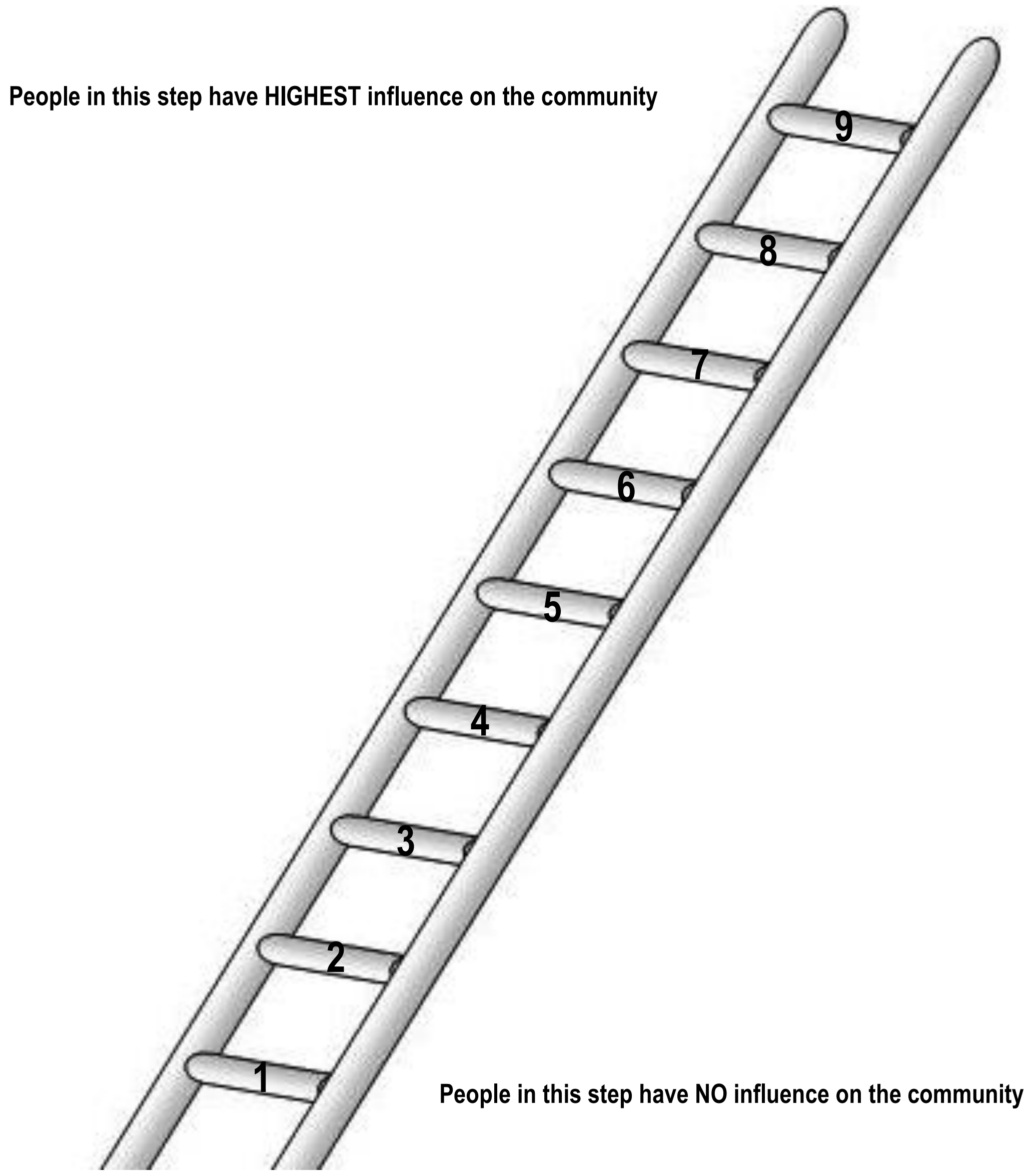

Household Number: | | | |

Module WE4 continued: Group Membership (Male and Female)

Now I'm going to ask you about groups in the community. These can be either formal or informal and customary groups.

Group membership		Is there a [GROUP] in your community?<br><br>Yes................ 1<br>No.................2>> next row<br>Don't know...97>>next row	Are you an active member of any [GROUP]?<br><br>Yes............1<br>No 2	Do you have a leadership position in this [GROUP]?<br><br>Yes... 1 >> WE4_11<br>No ...2	Have you ever had a leadership position in this [GROUP]?<br><br>Yes .... 1<br>No............. 2	Is this a single-sex group?<br><br>Yes........... 1<br>No 2	How often does the [GROUP] meet?<br>More than once a week .. 1<br>Once a week..................2<br>Once every two weeks....3<br>Once a month ....4<br>Less than once a month .. 5	Out of the last 5 meetings, how many did you attend?<br><br>[Number of meetings]
	Group Names	WE4.07	WE4.08	WE4.09	WE4.10	WE4.11	WE4.12	WE4.13
A	Agricultural / livestock/ fisheries producer's group (including marketing groups)							
B	Water users' group							
C	Forest users' group							
D	Credit or microfinance group (incl. ROSCAs/merry-go-rounds)							
E	Mutual help or insurance group (including burial societies)							
F	Trade and business association							
G	Civic groups (improving community) or charitable group (helping others)							
H	Local government							
I	Religious group							
J	Other women's group (only if it does not fit into one of the other categories)							
K	Other (specify)							

Household Number: | | | | |

Module WE5a: Decision Making (Male and Female)

I will now ask you some questions about decision making pertaining to the household

Enumerator: The purpose of this module is to get additional information about decision making within households.

Again, do not attempt to ensure that responses are the same between the male and female respondent. It is okay for them to be different.

Serial no		When decisions are made regarding the following aspects of household life, who is it that normally takes the decision?<br><br>Self.............1 >>skip to the next activity<br>Husband/ wife....................................... 2<br>Someone else in the household............. 3<br>Someone outside the household/other... 4<br>Not applicable………..98 >> skip to the next activity<br><br>In case of joint decisions, there could be multiple responses			To what extent do you feel you can make your own personal decisions regarding these aspects of household life if you want(ed) to?<br><br>Not at all ....1<br>Small extent.................................2<br>Medium extent.............................3<br>To a high extent...........................4
CODE	ACTIVITY	WE5a_01a	WE5a_01b	WE5a_01c	WE5a_02
A	What inputs to buy for agricultural production?				
B	What types of crops to grow for agricultural production?				
C	When or who would take crops to the market?				
D	Livestock raising?				
E	Your own wage or salary employment?				
F	Major household expenditures?				
G	Minor household expenditures?				

Household Number: | | | |

Module WE5b: Motivation for decision-making (Male and Female)

I will tell you a few stories about the agricultural practices of a few farmers. These questions are a little different from the rest. So feel free to take your time. After each story I will ask you with whom you identify the most and least. I will also want to know whether you are very different from them, very similar to them or somewhere in between. There are no right or wrong answers to these.

Enumerator: Read out each story. Read out the next question and their codes loudly. Circle one response.

			STORY	QUESTION 1	QUESTION 2	QUESTION 3
A	The types of crops to grow for consumption and sale in market	**WE5ba_01**	*"Anzira (Abdullah) can't grow other types of crops here for consumption and sale in market. Beans, sweet potato and maize are the only crops that grow here."*	Are you like this person?<br><br>Yes .... 1<br>No .... 2 → Question 3<br>[ ]	Are you completely the same or somewhat the same?<br><br>Completely the same .... 1<br>Somewhat the same .... 2<br>[ ]	Are you completely different or somewhat different?<br><br>Completely different .... 1<br>Somewhat different .... 2<br>[ ]
		**WE5ba_02**	*"[PERSON'S NAME] is a farmer and grows beans, sweet potato, and maize because her spouse, or another person or group in her community tells her she must grow these crops. She does what they tell her to do."*	Are you like this person?<br><br>Yes .... 1<br>No .... 2 → Question 3<br>[ ]	Are you completely the same or somewhat the same?<br><br>Completely the same .... 1<br>Somewhat the same .... 2<br>[ ]	Are you completely different or somewhat different?<br><br>Completely different .... 1<br>Somewhat different .... 2<br>[ ]
		**WE5ba_03**	*"[PERSON'S NAME] grows the crops for agricultural production that her family or community expect. She wants them to approve of her as a good farmer."*	Are you like this person?<br><br>Yes .... 1<br>No .... 2 → Question 3<br>[ ]	Are you completely the same or somewhat the same?<br><br>Completely the same .... 1<br>Somewhat the same .... 2<br>[ ]	Are you completely different or somewhat different?<br><br>Completely different .... 1<br>Somewhat different .... 2<br>[ ]
		**WE5ba_04**	*"[PERSON'S NAME] chooses the crops that she personally wants to grow for consumption and sale in market and thinks are best for her family and business. She values growing these crops. If she changed her mind, she could act differently."*	Are you like this person?<br><br>Yes .... 1<br>No .... 2 → Question 3<br>[ ]	Are you completely the same or somewhat the same?<br><br>Completely the same .... 1<br>Somewhat the same .... 2<br>[ ]	Are you completely different or somewhat different?<br><br>Completely different .... 1<br>Somewhat different .... 2<br>[ ]

Household Number: [ ][ ][ ][ ]

B	Taking crops to the market (or not)	**WE5bb_01**	*"There is no alternative to how much or how little of her crops [PERSON'S NAME] can take to the market. She is taking the only possible amount."*	Are you like this person?<br><br>Yes ....1<br>No ....2 → Question 3<br>[ ]	Are you completely the same or somewhat the same?<br><br>Completely the same .... 1<br>Somewhat the same .... 2<br>[ ]	Are you completely different or somewhat different?<br><br>Completely different ....1<br>Somewhat different ....2<br>[ ]
		**WE5bb_02**	*"[PERSON'S NAME] takes crops to the market because her spouse, or another person or group in her community tells her she must sell them there. She does what they tell her to do."*	Are you like this person?<br><br>Yes ....1<br>No ....2 → Question 3<br>[ ]	Are you completely the same or somewhat the same?<br><br>Completely the same .... 1<br>Somewhat the same .... 2<br>[ ]	Are you completely different or somewhat different?<br><br>Completely different ....1<br>Somewhat different ....2<br>[ ]
		**WE5bb_03**	*"[PERSON'S NAME] takes the crops to the market that her family or community expect. She wants them to approve of her as a good business woman."*	Are you like this person?<br><br>Yes ....1<br>No ....2 → Question 3<br>[ ]	Are you completely the same or somewhat the same?<br><br>Completely the same .... 1<br>Somewhat the same .... 2<br>[ ]	Are you completely different or somewhat different?<br><br>Completely different ....1<br>Somewhat different ....2<br>[ ]
		**WE5bb_04**	*"[PERSON'S NAME] chooses to take the crops to market that she personally wants to sell there, and thinks is best for her family and business. She values this approach to sales. If she changed her mind, she could act differently."*	Are you like this person?<br><br>Yes ....1<br>No ....2 → Question 3<br>[ ]	Are you completely the same or somewhat the same?<br><br>Completely the same .... 1<br>Somewhat the same .... 2<br>[ ]	Are you completely different or somewhat different?<br><br>Completely different ....1<br>Somewhat different ....2<br>[ ]

Household Number: | | | | |

C	Livestock raising	**WE5bc_01**	*"[PERSON'S NAME] can't raise any livestock other than what she has. These are all that's available."*	Are you like this person?<br><br>Yes ....1<br>No ....2 → Question 3<br>[ ]	Are you completely the same or somewhat the same?<br><br>Completely the same .... 1<br>Somewhat the same .... 2<br>[ ]	Are you completely different or somewhat different?<br><br>Completely different ....1<br>Somewhat different ....2<br>[ ]
		**WE5bc_02**	*"[PERSON'S NAME] raises the types of livestock she does because her spouse, or another person or group in her community tell her she must use these breeds. She does what they tell her to do."*	Are you like this person?<br><br>Yes ....1<br>No ....2 → Question 3<br>[ ]	Are you completely the same or somewhat the same?<br><br>Completely the same .... 1<br>Somewhat the same .... 2<br>[ ]	Are you completely different or somewhat different?<br><br>Completely different ....1<br>Somewhat different ....2<br>[ ]
		**WE5bc_03**	*"[PERSON'S NAME] buys the kinds of livestock that her family or community expect. She wants them to approve of her as a good livestock raiser."*	Are you like this person?<br><br>Yes ....1<br>No ....2 → Question 3<br>[ ]	Are you completely the same or somewhat the same?<br><br>Completely the same .... 1<br>Somewhat the same .... 2<br>[ ]	Are you completely different or somewhat different?<br><br>Completely different ....1<br>Somewhat different ....2<br>[ ]
		**WE5bc_04**	*"[PERSON'S NAME] chooses the types of livestock that she personally wants to raise and thinks are good for her family and business. She values raising these types. If she changed her mind, she could act differently."*	Are you like this person?<br><br>Yes ....1<br>No ....2 → Question 3<br>[ ]	Are you completely the same or somewhat the same?<br><br>Completely the same .... 1<br>Somewhat the same .... 2<br>[ ]	Are you completely different or somewhat different?<br><br>Completely different ....1<br>Somewhat different ....2<br>[ ]

Household Number: | | | | |

Module WE5c: Decision making (Male and Female)

Serial no.	*ENUMERATOR:* This is the last set of questions and it is very important. I am going to give you some reasons for why you might undertake activities in the domains I just mentioned. You might have several reasons for doing each one and there is no right or wrong answer. Please tell me how true it would be to say:	Your actions with respect to [DOMAIN] are motivated by a desire to avoid punishment or gain reward?<br>Can you tell me whether it is entirely true, somewhat true, not very true or never true?<br>[READ OPTIONS]<br>**CODE 1↓**	Your actions with respect to [DOMAIN] are motivated by a desire to avoid blame or so that other people speak well of you?<br>Can you tell me whether it is entirely true, somewhat true, not very true or never true?<br>[READ OPTIONS]<br>**CODE 1↓**	Your actions with respect to [DOMAIN] are motivated by and reflect your own values and/or interests?<br>Can you tell me whether it is entirely true, somewhat true, not very true or never true?<br>[READ OPTIONS]<br>**CODE 1↓**	How satisfied were you with the decisions made in [DOMAIN]?<br>[READ OPTIONS]<br>**CODE 2↓**
		**WE5c_03**	**WE5c_04**	**WE5c_05**	**WE5c_06**
**A**	Nonfarm business activity?				
**B**	Your own wage or salary employment?				
**C**	Minor household expenditures?				
**D**	What to do if you have a serious health problem?				
**E**	How to protect yourself from violence?				
**F**	Whether and how to express religious faith?				
**G**	What kind of tasks you will do on a particular day?				
**H**	Whether or not to use family planning to space or limit births?				

**CODE 1: Motivation for activity**	**CODE 2: Extent of satisfaction with activity**
Always true ....1<br>Somewhat true ....2<br>Not very true ....3<br>Never true ....4<br>Decision not made................................98	Very satisfied...................................... 1<br>Somewhat satisfied............................. 2<br>Neither satisfied nor dissatisfied.......... 3<br>Somewhat dissatisfied .... 4<br>Very dissatisfied.................................. 5<br>Decision not made 98

Module WE6a: Time allocation (Male and Female)

Enumerator: The purpose of this module is to get an idea about men's and women's time spent in both work and leisure activities and their satisfaction with their time use.

WE6.01a: Please record a log of the activities for the individual in the last complete 24 hours (starting yesterday morning at 4 am, finishing 3.59 am of the current day). The time intervals are marked in 15 min intervals. Now we will ask you how you spent the last 24 hours. We will start from yesterday morning. This is an account of the entire time period. We are interested in knowing about all your activities (such as resting, eating, personal care, house and outside house work, child care, cooking, shopping, socializing, etc.), even those activities which do not take up a lot of time.

		If simultaneous: Primary ….1 Secondary 2	Night								Morning				Day																																			
	Activity↓		4				5				6				7				8				9				10				11				12				13				14				15			
A	Sleeping and resting																																																	
B	Eating and drinking																																																	
C	Personal care																																																	
D	School (also homework)																																																	
E	Work as employed																																																	
F	Own business work																																																	
G	Farming/Fishing																																																	
J	Shopping/getting service																																																	
K	Weaving, sewing, textile care																																																	
L	Cooking																																																	
M	Domestic work																																																	
N	Care for [illegible]																																																	
O	Commuting																																																	
P	Travelling																																																	
Q	Watching TV/listening to [illegible]																																																	
R	Reading																																																	
S	Sitting with family																																																	
T	Exercising																																																	
U	Social activities																																																	
V	Practicing hobbies																																																	
W	Religious activities																																																	
X	Other, specify…																																																	

Household Number: | | | |

WE6a: Continued

	Activity↓	If simultaneous: Primary ....1 Secondary 2	Night		Morning	Day								
			16	17	18	19	20	21	22	23	24	1	2	3
A	Sleeping and resting													
B	Eating and drinking													
C	Personal care													
D	School (also homework)													
E	Work as employed													
F	Own business work													
G	Farming/Fishing													
J	Shopping/getting service													
K	Weaving, sewing, textile care													
L	Cooking													
M	Domestic work													
N	Care for [illegible]													
O	Commuting													
P	Travelling													
Q	Watching TV/listening to [illegible]													
R	Reading													
S	Sitting with family													
T	Exercising													
U	Social activities													
V	Practicing hobbies													
W	Religious activities													
X	Other, specify…													

Household Number: | | | | |

Module WE6b: Satisfaction with Time Allocation (Male and Female)

Q. NO.	QUESTION	RESPONSE	RESPONSE CODE
**WE6.01b**	In the last 24 hours, did you work (at home or outside of the home)		More than usual...............................1<br>About the same as usual.................2<br>Less than usual................................3

Q. No.	Question	Response	Response options/Instructions
**WE6_02**	Was yesterday a holiday or nonworking day?		Yes...1<br>No 2
**WE6_03**	Regarding the amount of sleep you got last night, was that [READ RESPONSES]::		Less than average ....1<br>Average ....2<br>More than average 3
**WE6_04**	*READ:* I am going to ask you a series of questions and I want you to tell me how you would rate your satisfaction on a scale of 1 to 10, where 1 means you are not satisfied and 10 means you are very satisfied. If you are neither satisfied or dissatisfied this would be in the middle or 5 on the scale.<br>How would you rate your satisfaction with:		
**WE6_04A**	The distribution of work duties within your household?		**Please mark on a scale from 1 – 10**<br>Not satisfied ☹...1<br>⇩<br>Neither satisfied nor dissatisfied 😐..............5<br>⇩<br>Very satisfied ☺...10
**WE6_04B**	Your available time for leisure activities like watching TV, listening to radio, seeing movies or doing sports?		
**WE6_04C**	Your contacts with friends or relatives?		
**WE6_04D**	Your possibilities of going to other places outside your village?		
**WE6_04E**	Your power to make important decisions that change the course of your life?		
**WE6_04F**	Your satisfaction with your life overall?		
**WE6_05**	During the last four weeks, how many days of your primary daily activities did you miss because of poor health?		Enter number of days [1-28]
**WE6_06**	Were the last four weeks typical or average?		Worse than average ....1<br>Average ....2<br>Better than average 3
**WE6_07**	Were you unable to complete normal activities in the last 24 hours?		Yes...1<br>No 2

Household Number: | | | | |

Q. No.	Question	Response	Response options/Instructions
**WE6_08**	Do you suffer from a chronic disability?		Yes....1<br>No ....2 >> WE6_10
**WE6_09**	If yes, what kind?<br><br>[ALLOW UP TO 3 RESPONSES]	**WE6_09_a** | **WE6_09_b** | **WE6_09_c**	Deaf or hearing disability....1<br>Mobility or missing limbs....2<br>Speech or language disability....3<br>Learning or mental impairment....4<br>Blind or visual disability....5<br>Chronic health or disease....6<br>Psychological or emotional ....7<br>Other, specify....8
	**ENUMERATOR: If male, stop and proceed to Module WE6_12; If female, continue from question WE6_10:**		
**WE6_10**	Are you currently pregnant?		Yes....1<br>No 2
**WE6_11**	Are you currently breastfeeding?		Yes....1<br>No 2

Module WE6b continued: Satisfaction with Time Allocation

Q. NO.	QUESTION	RESPONSE OPTIONS/INSTRUCTIONS
**WE6_12**	Next, I am going to ask you a question about how satisfied you are with the time you have to yourself to do things you enjoy. Please give your opinion on a scale of 1 to 10. 1 means you are not satisfied and 10 means you are very satisfied. If you are neither satisfied nor dissatisfied, this would be in the middle, or 5, on the scale.<br><br>NOTE: LEISURE EXAMPLES SHOULD BE MODIFIED FOR LOCAL CONTEXT.<br><br>How satisfied are you with your available time for leisure activities like visiting neighbors, watching TV, listening to the radio, seeing movies or doing sports?	SATISFACTION RATING: | | |

Household Number: | | | | |

Module WE7b: Parent's information (Male and Female)

SL	Questions	Answer	Answer code	
**WE7b _1**	What's your (respondent's) mother's highest level of education?	☐	Never attended school .... 99<br>Studying in Class I...................... 0<br>Completed Class I...................... 1<br>Completed Class II .... 2<br>Completed Class III.................... 3<br>Completed Class IV.................... 4<br>Completed Class V .... 5<br>Completed Class VI.................... 6<br>Completed Class VII .... 7<br>Completed Class VIII .... 8<br>Completed Class IX.................... 9<br>SSC/Dakhil................................ 10<br>HSC/Alim.................................. 12	BA/BSC pass/fazil ....14<br>BA/BSC honorary/fazil ....15<br>MA/MSC and above/kamil ....16<br>SSC candidate ....22<br>HSC candidate............................33<br>Any class below Class I ....66<br>Mosque related primary school ..67<br>Medical/MBBS ....71<br>Nursing ....72<br>Engineer.....................................73<br>Diploma Engineer ....74<br>Vocational Education ....75<br>Others...76
**WE7b _2**	What's your (respondent) father's highest level of education?	☐		
**WE7b _3**	Number of alive sisters (including respondent)	☐	Number of sisters	
**WE7b _4**	Number of alive brothers (including respondent)	☐	Number of brothers	
**WE7b _5**	Where were you born?	☐	I am living where I was born ....1<br>In this village/ward ....2<br>In another village of this union/ward ....3<br>In another union of this station ....4<br>In another station of this district ....5<br>In another district of this division ....6<br>In another division (specify 7	
**WE7b _6**	How often do you read newspapers/magazines?	☐	Everyday...1<br>Every week at least once...2<br>Every 2 weeks at least once ....3<br>Every month at least once ....4<br>Irregularly...5<br>Hardly/almost never ....6>>WE7b_8	
**WE7b _7**	Do you read any national or regional newspaper or magazine?	☐	National ....1<br>Regional ....2<br>Both kinds 3	
**WE7b _8**	How often do you listen to the radio?	☐	Everyday...1<br>Every week at least once...2<br>Every 2 weeks at least once ....3<br>Every month at least once ....4<br>Irregularly...5<br>Hardly/almost never 6	

Household Number: [ ][ ][ ][ ]

SL	Questions	Answer	Answer code
**WE7b _9**	How often do you watch television?	[ ]	Everyday.......1<br>Every week at least once.......2<br>Every 2 weeks at least once ....3<br>Every month at least once ....4<br>Irregularly.......5<br>Hardly/almost never ....6>>WE7b_11
**WE7b _10**	Which channels do you watch often?	[ ]	BTV.......1<br>BTV World.......2<br>Other Local Chanel/Bangladeshi channel ....3<br>Foreign Bangla Channels.......4<br>Foreign other channels.......5
**WE7b _11**	Do you have your own mobile phone?	[ ]	Yes ....1<br>No.......2
**WE7b _12**	Do you use a mobile phone? (mobile phone could be his/her own or someone else's)	[ ]	Yes ....1<br>No.......2
**WE7b _13**	If yes, for what purpose do you use it?<br><br>(Mention the 3 main reasons)	[ ]<br>[ ]<br>[ ]	Talking to friends and family.......1<br>Financial Transactions (such as Bkash) ....2<br>Business purposes (such as contacting agriculture extension services or learning about market prices ....3<br>To access other kinds of information (such as health care, agriculture extension, services, other social services).......4<br>Entertainment (music, videos) ....5<br>To listen to FM radio 6
**WE7b _14**	Which operator do you use?<br><br>(Mention 2 main ones)	[ ]<br>[ ]	Grameen ....1<br>Rabi ....2<br>Banglalink ....3<br>Airtel ....4<br>Teletalk.......5<br>Citycell ....6<br>Others (mention name) 7
**WE7b _15**	If you are married, than who chose your partner?	[ ]	We chose each other.......1<br>I chose him/her and he/she agreed ....2<br>He/she chose me and I agreed ....3<br>He/she chose me but I did not agree ....4<br>The family chose and I agreed.......5<br>The family chose but I did not agree.......6<br>Others (mention here).......96

Household Number: | | | | |

Module WE7c: Parda information (Male and Female)

SL	Questions	Answer	Answer code
**WE7c_1**	What is done to protect the women (both young and old) in your family?		Providing a companion when they go out..........................1<br>Not allowing them to interact with close male relatives of similar age ....2<br>Not allowing them to go out in the evening.......................3<br>Cover them with a burkha when going out. ....4<br>Not allowing them to roam around here and there............5<br>Others (please mention here) 96
**WE7c _2**	Are the women (both young and old) required to cover the head when going out?		Yes ....1<br>No..2>> End of questionnaire. Fill out question WA11 from module WA
**WE7c_3**	What type of covering is used?		Burkha ....1<br>Scarf ....2<br>Cover the head with the saree ....3<br>Hijab/Nikab ....4<br>The veiling of the mind is enough 5
**WE7c_4**	What is the reason for covering up?<br><br>(Multiple responses possible)		Safety ....1<br>Religious reasons...2<br>Societal reasons ....3<br>Instructions from the family ....4<br>Personal choice...5<br>Others (mention here)...96<br>Don't know...98
**WE7c_5**	From what age are the women in the household required to cover their head?		Age (complete years)
**WE7c _6**	Do you cover your head when you go out of the house?		Yes ....1<br>No..2

Module End Time: | Hour | | | Minute | | |
