# Supplementary material for: Validity of Food insecurity experience scale (FIES) for use in rural Bangladesh and prevalence and determinants of household food insecurity: An analysis of data from Bangladesh integrated household survey (BIHS) 2018-2019
Source: Heliyon. 2023 Jun 17;9(6):e17378. doi: 10.1016/j.heliyon.2023.e17378 (PMC10329118; doi:10.1016/j.heliyon.2023.e17378)

**Supplementary tables and figures**

Table S1: Food groups and standard weight used in FCS calculation

| Food item | Food group | Weight |
| --- | --- | --- |
| Rice, wheat, potato, bread, or other locally available carbohydrate rich foods | Main staples | 2 |
| Bean, peas, lentil etc. | Pulse | 3 |
| Onion, tomato, brinjal, carrot or any kinds of vegetables including leafy vegetables | Vegetables | 1 |
| Guava, mango, banana, apple etc | Fruits | 1 |
| Any kind of dried or fresh meat and fish | Meat and fish | 4 |
| Milk, yogurt or other dairy products | Milk | 4 |
| Sugar or sugar products, honey | Sugar | 0.5 |
| Butter, ghee or oils added during food preparation | Fats and oils | 0.5 |
| Spices or beverages | Condiments | 0 |

Table S2: Questions for measuring hunger in BIHS survey

| Qs no | Question | Code | Code |
| --- | --- | --- | --- |
| 1 | In the past 4 weeks was there ever no food to eat of any kind in your house because of lack of resources to get food? | Yes… 1  No… 2 >> qs_3 |  |
| 2 | How often did this happen in the past 4 weeks? | Rarely (1-2 times) … 1  Sometimes (3-10 times) … 2  Often (> 10 times) …. 3 |  |
| 3 | In the past 4 weeks did you or any household member go to sleep at night hungry because there was not enough food? | Yes… 1  No… 2 >> qs_5 |  |
| 4 | How often did this happen in the past 4 weeks? | Rarely (1-2 times) … 1  Sometimes (3-10 times) … 2  Often (> 10 times) …. 3 |  |
| 5 | In the past 4 weeks did you or any household member go a whole day and night without eating anything at all because there was not enough food? | Yes… 1  No… 2 |  |
| 6 | How often did this happen in the past 4 weeks? | Rarely (1-2 times) … 1  Sometimes (3-10 times) … 2  Often (> 10 times) …. 3 |  |

Table S3: Sociodemographic profile of study participants (N = 5603), Bangladesgh Integrated Household Survey 2018-2019

| Characteristics | n (weighted percentage) |
| --- | --- |
| **Total** | 5603 |
| **Division**  Barisal  Chittagong  Dhaka  Khulna  Rajshahi  Rangpur  Sylhet | 412 (5.7)  974 (17.0)  1724 (31.0)  553 (12.2)  616 (14.8)  572 (13.2)  752 (6.0) |
| **Gender**  Male  Female | 6372 (45.5)  7930 (54.5) |
| **Age category**  18-34  35-49  50+ | 5934 (43.5)  4156 (28.4)  4221 (28.2) |
| **Ownership of household**  Owned  Rented | 5415 (96.8)  188 (3.2) |
| **Primary adult decision maker**  Male  Female | 4472 (80.5)  1131 (19.5) |
| **Access to electricity**  Yes  No | 4805 (86.0)  798 (14.0) |
| **Access to sanitation**  Improved  Not improved | 2765 (49.0)  2838 (51.0) |
| **Livestock ownership**  Yes  No | 2727 (51.4)  2876 (48.6) |
| **Monthly per capita food expenditure**  ≤ 25 USD  ≥ 26 USD | 2802 (51.6)  2801 (48.4) |
| **Family size**  ≤ 4  5+ | 3556 (65.6)  2047 (34.4) |
| **Education level of respondent**  Never attend school  Primary or below  Secondary or higher | 2423 (43.4)  1499 (26.1)  1681 (30.5) |

Table S4: Residual correlation between FIES items

|  | Healthy | Few food | Skipped | Ate less | Runout | Hungry | Whole day |
| --- | --- | --- | --- | --- | --- | --- | --- |
| Worried | -0.17 | -0.03 | -0.02 | 0.02 | 0.01 | 0.01 | 0.0 |
| Healthy |  | 0.28 | 0.01 | -0.14 | -0.07 | -0.2 | -0.02 |
| Fewfood |  |  | 0.04 | 0.06 | 0.01 | -0.01 | 0.0 |
| Skipped |  |  |  | 0.13 | 0.06 | -0.19 | 0.02 |
| Ateless |  |  |  |  | 0.14 | 0.22 | 0.01 |
| Runout |  |  |  |  |  | 0.11 | 0.09 |
| Hungry |  |  |  |  |  |  | -0.01 |

Table S5: INFIT statistics by region

| Item | Barisal | Chittagong | Dhaka | Khulna | Rajshahi | Rangpur | Sylhet |
| --- | --- | --- | --- | --- | --- | --- | --- |
| Worried | 1.35 | 1.39 | 1.01 | 1.22 | 1.47 | 1.0 | 1.04 |
| Healthy | 0.86 | 0.79 | 1.13 | 0.77 | 0.64 | 1.7 | 0.96 |
| Fewfood | 0.97 | 0.94 | 0.73 | 0.69 | 0.99 | 0.61 | 0.43 |
| Skipped | 1.07 | 0.87 | 0.8 | 1.12 | 1.27 | 0.87 | 1.16 |
| Ateless | 0.93 | 0.87 | 0.76 | 0.97 | 0.67 | 0.59 | 0.94 |
| Runout | 0.72 | 0.78 | 0.81 | 0.33 | 0.76 | 0.78 | 0.42 |
| Hungry | 1.03 | 1.1 | 0.78 | 0.49 | 1.06 | 1.29 | 1.15 |
| Whlday | 0.0 | 0.97 | 0.99 | 0.0 | 0.0 | 0.0 | 0.0 |

Table S6: Probability of food insecurity based on FIES raw score

| Raw score | Probability of moderate or severe FI | Probability of severe FI |
| --- | --- | --- |
| 0 | 0.0 | 0.0 |
| 1 | 0.004 | 0.0 |
| 2 | 0.042 | 0.0 |
| 3 | 0.32 | 0.0 |
| 4 | 0.77 | 0.0 |
| 5 | 0.97 | 0.0 |
| 6 | 0.99 | 0.0 |
| 7 | 0.99 | 0.26 |
| 8 | 1 | 0.63 |

Adjusted thresholds of food insecurity on the latent trait: Moderate or severe FI (-1.07), Severe FI (7.48)

Figure S1: Equating Plot


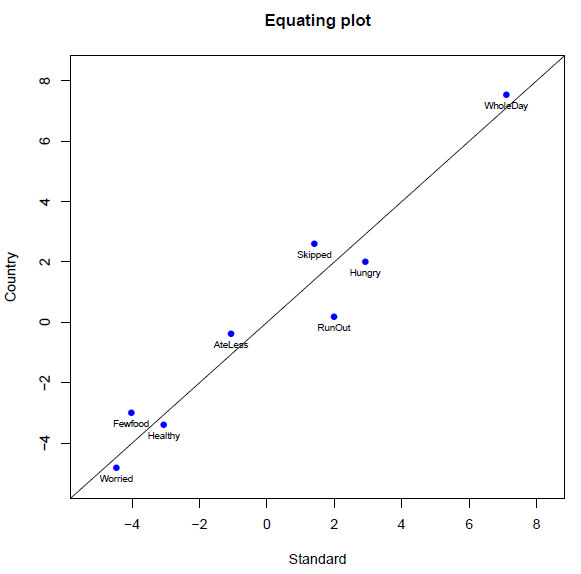

Supplement: Supplementary tables and figures [file mmc2.docx]
